# Comprehensive mapping of O‐glycosylation in flagellin from *Campylobacter jejuni* 11168: A multienzyme differential ion mobility mass spectrometry approach

**DOI:** 10.1002/pmic.201400533

**Published:** 2015-06-15

**Authors:** Gloria N. Ulasi, Andrew J. Creese, Sam Xin Hui, Charles W. Penn, Helen J. Cooper

**Affiliations:** ^1^ School of BiosciencesUniversity of BirminghamEdgbastonBirminghamUK

**Keywords:** *Campylobacter jejuni* 11168, Flagellin A, Glycoproteomics, Glycosylation, LC FAIMS MS/MS */* Proteinase K, Trypsin

## Abstract

Glycosylation of flagellin is essential for the virulence of *Campylobacter jejuni*, a leading cause of bacterial gastroenteritis. Here, we demonstrate comprehensive mapping of the O‐glycosylation of flagellin from *Campylobacter jejuni* 11168 by use of a bottom‐up proteomics approach that incorporates differential ion mobility spectrometry (also known as high field asymmetric waveform ion mobility spectrometry or FAIMS) together with proteolysis with proteinase K. Proteinase K provides complementary sequence coverage to that achieved following trypsin proteolysis. The use of FAIMS increased the number of glycopeptides identified. Novel glycans for this strain were identified (pseudaminic acid and either acetamidino pseudaminic acid or legionaminic acid), as were novel glycosylation sites: Thr208, Ser343, Ser348, Ser349, Ser395, Ser398, Ser423, Ser433, Ser436, Ser445, Ser448, Ser451, Ser452, Ser454, Ser457 and Thr465. Multiply glycosylated peptides were observed, as well as variation at individual residues in the nature of the glycan and its presence or absence. Such extreme heterogeneity in the pattern of glycosylation has not been reported previously, and suggests a novel dimension in molecular variation within a bacterial population that may be significant in persistence of the organism in its natural environment. These results demonstrate the usefulness of differential ion mobility in proteomics investigations of PTMs.

**Abbreviations**
AGC: automatic gain control
FAIMS: field asymmetric waveform ion mobility spectrometry

## 1 Introduction

*Campylobacter jejuni* is the most prevalent foodborne bacterial agent of diarrhoeal disease in humans worldwide 1. This zoonosis enters the human diet from poultry and farm animal sources in which the organism causes little pathology 2. Disease in humans is usually self‐limiting and relatively short‐lived although symptoms can be severe, such that healthcare and economic impact are considerable 3. A minority of cases lead to more serious sequelae such as Guillain–Barré motor neuropathy 4, the most common cause of general paralysis in developed countries since the eradication of poliomyelitis.

Virulence factors are not fully understood but it is clear that flagellar motility is crucial in the colonisation by the organism of digestive tracts in birds 5, 6, hence is also likely to be required for infection in humans, a conclusion supported by experimental infection in human subjects 7. The flagellum is not only responsible for motility of campylobacters, but is also responsible for secretion of important colonisation and virulence factors, reviewed in 8. The flagellar filament protein, flagellin, is both a dominant antigen important as a target of immune reactivity 9, and a receptor for bacteriophage attack 10. A recent report demonstrated the involvement of flagellin glycan residues in phage receptor activity 11. Hence there are strong selective pressures that may drive molecular variation in the flagellin molecule, with important implications in the control of infection.

Flagellins FlaA (major, ca. 90% of total) and FlaB, the major structural protein monomers of the flagellar filament, are proteins each of 572 amino acid residues totalling approximately 59 kDa, although apparent M_r_ determined by SDS‐PAGE is about 10% higher, due to the presence of numerous glycan residues: for an overview see 12. The two proteins are 94% identical in amino acid sequence, with very few differences in the glycosylated non‐terminal portion of the molecule that is known to be exposed on the surface of the assembled structure. We have therefore taken the sequence of FlaA as the basis for our observations and refer to it throughout as ‘flagellin’. Although it has been shown in strains 81–176 13, 14 and NCTC 11168 15 that a number of specific amino acid residues of flagellin may be O‐glycosylated, it is unclear whether these modifications are always present or whether there is variation within the population of molecules in the nature of the glycan or the extent of glycosylation. Flagellin is rich especially in serine (approx. 11% of residues in strain 11168 and the most commonly modified residue) and also in threonine (approx. 6%) but many of these residues have not been shown to be targets for modification. It is not known how specific residues are selected for glycosylation or whether this process is tightly controlled or stochastic in nature.

MS was first applied to the analysis of glycosylation of flagellins from *Campylobacter jejuni* (81–176, 11168 and OH4384) and *Campylobacter coli* (VC167 T2) by Thibault et al. 16. The major glycans identified were pseudaminic acid and derivatives thereof. Further work on *C. coli* (VC167) revealed the presence of legionaminic acid and derivatives 17. Previously in our laboratory 18 we applied LC electron capture dissociation MS/MS (LC ECD MS/MS) to the analysis of glycosylation in flagellin from *Campylobacter jejuni* 11168, revealing that the protein was modified by dimethyl glyceric acid derivatives of pseudaminic acid and acetamidino pseudaminic acid at Ser181, Ser207 and either Thr 464 or Thr465. That work was in agreement with the work of Logan et al. who first identified these novel glycans 19. Although the LC ECD MS/MS approach was successful in identifying the presence and site of glycosylation in flagellin from *C. jejuni* 11168, there was an extensive region of the protein (residues 387–463) for which no coverage was achieved. This observation is particularly salient as the homologous region in flagellin from *C. jejuni* 81–176 is heavily glycosylated 16. Moreover, the region contains numerous potential sites of O‐glycosylation, 21 serine residues and four threonine residues, affording the opportunity for differential glycosylation within the region. Any (non‐tryptic) proteolysis within the region is therefore likely to result in a complex mixture comprising multiple glycopeptide isomers and isobars.

In order to address the limited flagellin coverage obtained with trypsin, we have applied an alternative protease for the analysis of flagellin, that is, proteinase K. Proteinase K has a broad specificity and cleaves peptide bonds that are C‐terminal to aliphatic and aromatic residues 20. It has been previously successfully applied to the analysis of glycoproteins 21, 22. Given the broad specificity of proteinase K and the likelihood of extensive glycosylation in the region of interest, we also incorporated gas‐phase fractionation in the form of high field asymmetric waveform ion mobility spectrometry (FAIMS), in the workflow. FAIMS separates ions at atmospheric pressure on the basis of differences in their differential ion mobility. We, and others, have shown that FAIMS is useful in extending proteome coverage 23, 24, 25. We have also shown that FAIMS is capable of separating glycopeptide isomers 26. Here, we demonstrate comprehensive mapping of O‐glycosylation in flagellin from *Campylobacter jejuni* 11168 by use of this multienzyme differential ion mobility approach.

## 2 Materials and methods

### 2.1 Preparation of flagellin A

*Campylobacter jejuni* (NCTC 11168) cultures were grown on Mueller Hinton agar (Oxoid Ltd., Basingstoke, UK) and incubated in microaerophilic conditions (90% N_2_, 4% O_2_, and 6% CO_2_ at 37°C) for 30 h. Cells were harvested from 13 separate agar plates and suspended in 5.7 mL of Luria‐Bertani Broth. The suspension was homogenised at high speed (20 500 rpm) using an Ultra Turrax T‐25 homogeniser, for 2 min, with 30 s rest, for four cycles in order to shear the flagellar filament from the bacterial wall. The homogenate was centrifuged at 7500 rpm for 20 min at 4°C to remove cells and cell debris. The supernatant was collected and treated with 1% Triton X (Bio‐Rad), incubated at 37°C for 45 min, and subjected to ultracentrifugation (TL‐100 Ultracentrifuge, TLA‐100.3 fixed angle rotor, maximum RCF 541000 × g, Beckman, USA) at 50 000 rpm for 1 h at 4°C. The supernatant was removed immediately, tubes inverted on filter paper to remove residual fluid, and the resulting pellets were re‐suspended in 100 μL distilled water. A 5 μL aliquot of the sample was analysed by SDS‐PAGE, and visualised using Coomassie stain R250 (Bio‐Rad) to check flagellar protein purity, see Supporting Information Fig. 1. A further 5 μL was subjected to a Bradford protein assay to measure the protein concentration. The remaining sample was stored at –20°C until further analysis. Two biological replicates were considered.

### 2.2 Trypsin digestion

For LC MS/MS analysis, 10 μL of sample (∼7 μg of the extracted flagellar protein) was made up to 100 μL in 100 mM of ammonium bicarbonate (Fluka Analytical, UK)) and denatured by incubating at 95°C for 5 min. Trypsin (Sigma Aldrich, Dorset, UK) (1 μg/μL in 50 mM acetic acid (Sigma Aldrich, Dorset, UK)) was added to the mixture to give 1:50 (replicate#1) or 1:7 (replicate#2) ratio of enzyme to protein, and incubated overnight at 37°C with mild shaking. Proteolysis was quenched by freezing at –20°C and the sample stored until further analysis. For LC FAIMS MS/MS analysis, 50 μg of protein were digested using the digestion procedure as above.

### 2.3 Proteinase K digestion

Approximately 84 μL of the sample (70 μg of the extracted flagellar protein) was made up to 100 μL in 100 mM of ammonium bicarbonate (Fluka Analytical, UK) and denatured at 95°C for 5 min. Proteinase K (Sigma Aldrich, Dorset, UK) (1 μg/μL in 50 mM Tris‐HCl (Fisher Scientific, Loughborough, UK), 5 mM CaCl_2_ (BHD laboratory, England)) was added to the mixture to give a 1:1 (replicate#1) or 1:100 (replicate#2) ratio of enzyme to protein, and incubated for 1 h (replicate#1) or 2 h (replicate#2) at 37°C with mild shaking. Proteolysis was quenched by freezing at –20°C and the sample stored until further analysis.

Both trypsin‐ and proteinase K‐treated samples were desalted using C_18_ column zip tips (Merck Millipore Ltd., Germany) (according to manufacturers’ instructions). Briefly, the tips were wetted using 100% acetonitrile (J.T., Baker, Holland), and then equilibrated using 0.1% trifluoroacetic acid (Fisher Scientific Loughborough, UK). The samples were aspirated and dispensed for 10 cycles, and then washed three times using 0.1% TFA and eluted using 70:30 acetonitrile and 0.1% TFA. The desalted samples were dried and re‐suspended in 10 μL of 0.1% formic acid prior to MS analysis.

### 2.4 LC‐MS/MS

Peptides were separated using online reversed phase LC (Dionex Ultimate 3000) using a binary solvent system consisting of mobile phase A (water (J.T., Baker, Holland)/0.1% formic acid (Fisher Scientific, Loughborough, UK)) and mobile phase B (acetonitrile, (J.T., Baker, Holland)/0.1% formic acid (Fisher Scientific Loughborough, UK)). Six microliters of the desalted samples were loaded onto a 150 mm Acclaim® Pepmap 100 C_18_ column and separated by a 30 min gradient from 3.2 to 44% of mobile phase B, followed by 10 min at 90% of mobile phase B and 16 min with 3.2% mobile phase B to re‐equilibrate the system. The LC was coupled to an Advion Triversa Nanomate (Advion, Ithaca, USA) that infused the peptides at a spray voltage of 1.7 kV. Peptides were infused directly into the Thermo Orbitrap Velos ETD mass spectrometer (Thermo Fisher Scientific) at a flow rate of 0.35 μL/min.

The mass spectrometer alternated between a full FT‐MS scan (*m/z* 380–1600) and subsequent CID and ETD of the four most abundant precursor ions. Survey scans were acquired in the Orbitrap with a resolution of 60 000 at *m/z* 400. Only multiply charged precursor ions were selected for MS/MS. The dynamic exclusion was used with a repeat count of 1 for 30 s. Automatic gain control (AGC) was used to accumulate sufficient precursor ions. AGC target value for FT‐MS was 1 × 10^6^ charges. CID was performed in the linear ion trap using helium at normalised collision energy of 35%. Width of the precursor isolation window was 2 Th. AGC target was 5 × 10^4^ charges with maximum injection time of 100 ms. Charge state‐dependant ETD was performed in the linear ion trap with activation time 100 ms. Isolation window was 3 Th. AGC target of ETD was performed with fluoranthene ions. AGC target was 5 × 10^4^ charges with maximum injection time of 100 ms. Supplemental activation was enabled with activation energy of 25%. Data acquisition was controlled by Xcalibur 2.1 (Thermo Fisher Scientific).

### 2.5 FAIMS

The LC was coupled to the FAIMS device (Thermo Fisher Scientific), though a modified nanospray HESI II heated electrospray source, similar to that used by Swearingen et al. 25, incorporating a Picotip emitter needle (New Objective, Woburn, USA). The spray voltage was 3 kV. For FAIMS the dispersion voltage was –5000 V, inner and outer electrode temperatures were 70°C and 90°C, respectively. The gas flow was 2.9 L/min, consisting of 50:50 helium and nitrogen. Separate LC‐FAIMS‐MS/MS analyses were performed at the following compensation voltages: –20 V, –25 V, –30 V, –35 V, –40 V, –45 V, –50 V and –55 V (trypsin replicate #2; proteinase K replicate #2); –20 V, –25 V, –30 V, –35 V, –40 V, –45 V and –50 V ((trypsin replicate #1); –15 V, –20 V, –25 V, –30 V, –35 V, –40 V and –45 V (proteinase K replicate #1). LC and MS/MS method was the same as described above.

### 2.6 Data analysis

Raw data were loaded into Proteome Discoverer (version 1.4). Precursors with a mass of less than 350 Da and greater than 5000 Da were excluded and MS/MS spectra were required to have at least 1 peak. The CID and ETD spectra were separated and searched with the same parameters except for the fragment ions (*b* and *y* ions for CID and *c*, *y* and *z* ions for ETD). Data were searched against a manually created *Campylobacter* flagellin database (886 sequences) or a *Campylobacter* flagellin 11168 database (59 sequences) with sequences from the NCBI nr database. The data were searched using both the SEQUEST and Mascot algorithms (controlled through Proteome Discover version 1.4, Mascot version 2.4). In both SEQUEST and Mascot searches, the following parameters were used: precursor ion *m/z* tolerance 10 ppm; fragment ion tolerance 0.5 Da; enzyme—either trypsin or nonspecific enzyme (proteinase K); maximum number of missed cleavages = 2; no fixed modifications; dynamic modifications as described below. Multiple searches of the data were performed with different combinations of dynamic modifications (maximum number of dynamic modifications per search = 6). The glycans considered were as described in 18. The following combinations of dynamic modifications were considered: (A) oxidation +15.995 Da (M), C_13_H_20_N_2_O_7_, + 316.127 Da, (S/T), C_16_H_27_N_3_O_8_ + 389.180 Da (S/T), C_13_H_21_N_3_O_6_ + 315.143 Da (S/T) and C_16_H_26_N_2_O_9_ + 390.164 (S/T); (B) oxidation +15.995 Da (M), C_16_H_27_N_3_O_8_ + 389.180 Da (S/T), C_16_H_26_N_2_O_9_ + 390.164 (S/T), C_15_H_24_N_2_O_11_ + 408.138 Da (S/T), C_20_H_31_N_5_O_9,_ +485.212 Da (S/T), and C_15_H_22_N_2_O_8,_ + 358.137 Da (S/T); (C) oxidation +15.995 Da (M), C_16_H_27_N_3_O_8_ + 389.180 Da (S/T), C_16_H_26_N_2_O_9_ + 390.164 (S/T), C_13_H_20_N_2_O_7_ + 316.127 Da (S/T), C_20_H_30_N_4_O_10_ + 486.196 Da (S/T) and C_14_H_23_N_3_O_6_ + 329.1587 (S/T); (D) oxidation +15.995 Da (M), C_13_H_20_N_2_O_7_ +316.127 Da (S/T), C_13_H_21_N_3_O_6_ + 315.143 Da (S/T), C_15_H_24_N_2_O_11_ + 408.138 Da (S/T), C_20_H_31_N_5_O_9,_ +485.212 Da (S/T), and C_15_H_22_N_2_O_8,_ + 358.137 Da (S/T); (E) oxidation +15.995 Da (M), C_13_H_20_N_2_O_7_ +316.127 Da (S/T), C_13_H_21_N_3_O_6_ + 315.143 Da (S/T), C_20_H_30_N_4_O_10_ + 486.196 Da (S/T) and C_14_H_23_N_3_O_6_ +329.1587 (S/T) and C_16_H_26_N_2_O_9_ + 390.164 (S/T); (F) oxidation +15.995 Da (M), C_16_H_27_N_3_O_8_ + 389.180 Da (S/T), C_16_H_26_N_2_O_9_ + 390.164 (S/T), C_13_H_20_N_2_O_7_ +316.127 Da (S/T), C_13_H_21_N_3_O_6_ + 315.143 Da (S/T), and C_15_H_24_N_2_O_11_ + 408.138 Da (S/T). Glycopeptide identities were confirmed by manual analysis using the MS product package in Protein Prospector (http://prospector.ucsf.edu/prospector/mshome.htm).

## 3 Results and discussion

### 3.1 Trypsin proteolysis and LC MS/MS

**Scheme 1:**
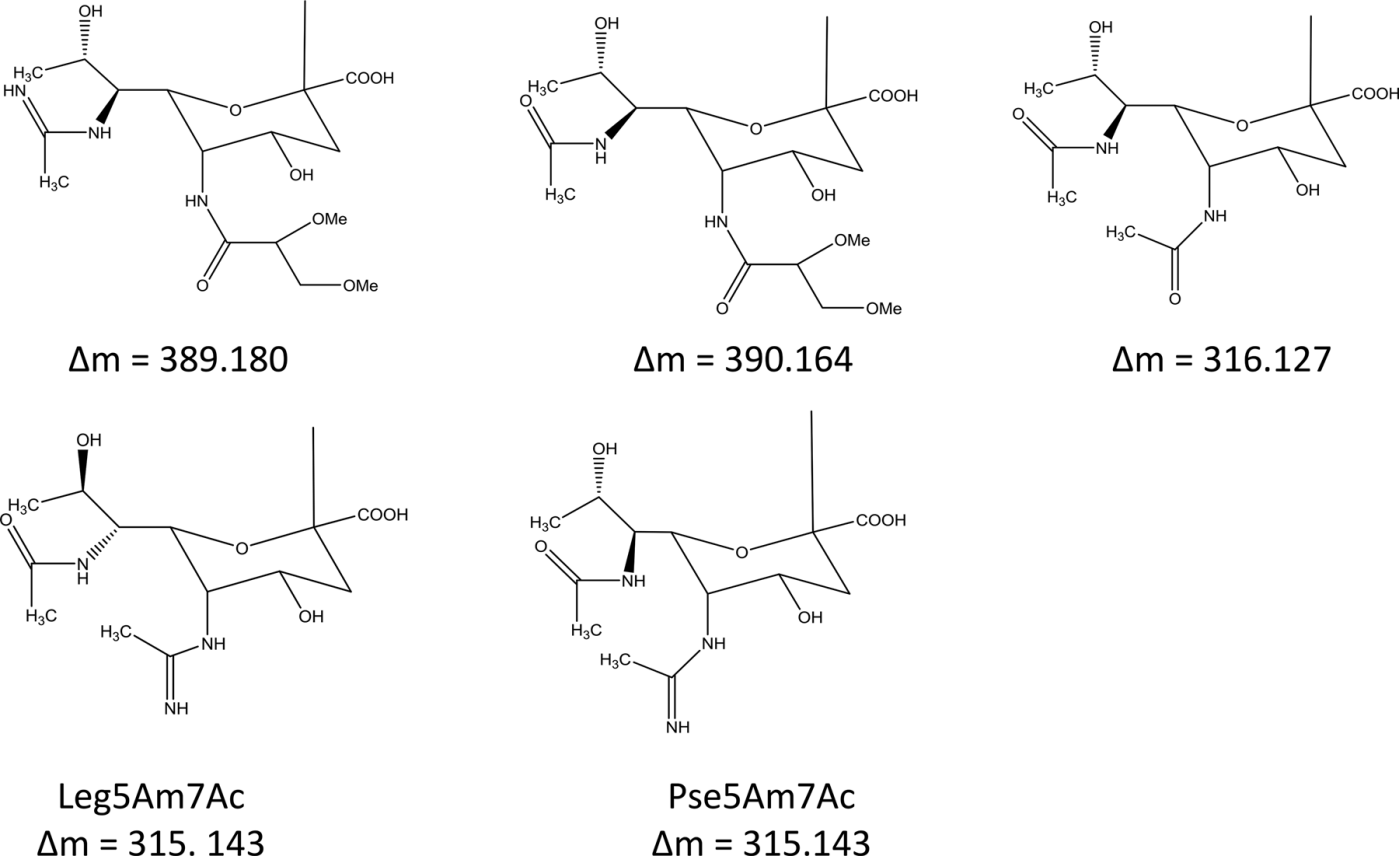
Structures of glycans identified in the protein database searches (based on findings in 19, 16, and 17).

In our previous paper 18, we used a combination of trypsin proteolysis and LC ECD MS/MS. Here, we have used a CID‐ETD method where each precursor ion is sequentially fragmented with CID and ETD. For comparison purposes, we first analysed the flagellin digest by LC CID ETD MS/MS without FAIMS. The data were searched against *Campylobacter* protein databases, using the Mascot and SEQUEST algorithms. A range of glycans were considered in the database searches as described in the experimental section. The glycans identified were dimethylglyceric acid derivative of pseudaminic acid (C_16_H_26_N_2_O_9_, Δm 390.164); dimethylglyceric acid derivative of 7‐acetamidino pseudaminic acid (C_16_H_27_N_3_O_8_, Δm 389.180), pseudaminic acid (C_13_H_20_N_2_O_7_, Δm 316.127), acetamidino pseudaminic acid/legionaminic acid (C_13_H_21_N_3_O_6_, Δm 315.143). The structures of these glycans are shown in Scheme 1. For convenience, the glycans will hereafter be referred to by their mass shift (Δm390, Δm389, Δm316, Δm315).

**Figure 1:**
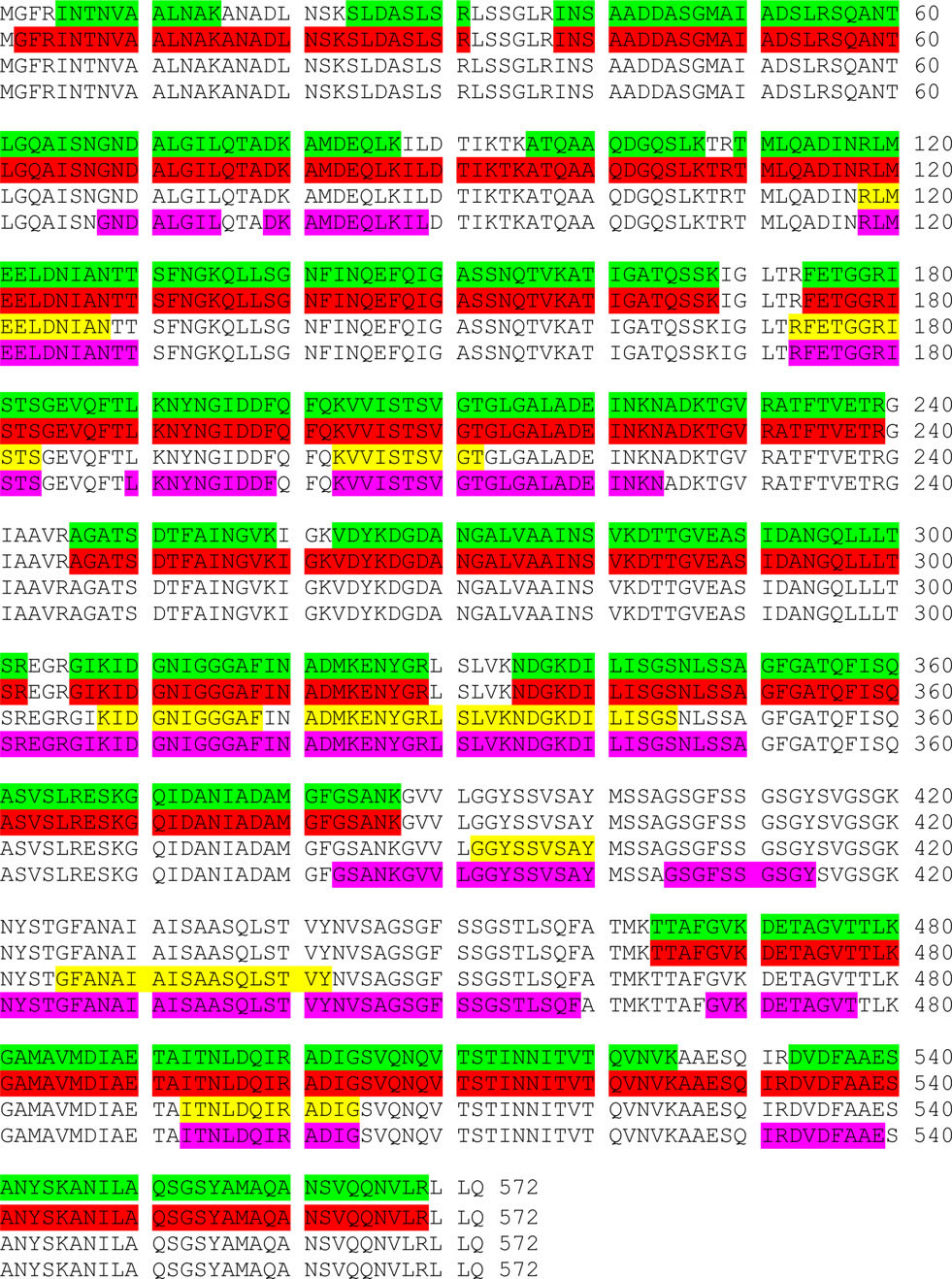
Sequence coverage obtained from ETD MS/MS for tryptic digest (no FAIMS) (green), tryptic digest (with FAIMS) (red); proteinase K digest (no FAIMS) (yellow); proteinase K (with FAIMS) (pink).

The combined protein sequence coverage obtained from the two replicates with CID was 76.2%. Eighteen glycopeptides were identified in the two database searches, however manual analysis of the mass spectra revealed that in each case fragmentation was poor and the peptide sequence coverage insufficient to assign sites of glycosylation. The combined protein sequence coverage obtained for ETD was 76.2%, see Fig. 1. Nine glycopeptides were assigned in the database search of the ETD data, but in this case, the fragmentation spectra were of sufficient quality to assign the modification sites in all but one case. Table 1 summarises the glycopeptides identified. (Unmodified peptides are summarised in Supporting Information Table 1). The ETD MS/MS spectra for the glycopeptides are shown in Supporting Information Fig. 2. Although the ETD mass spectra are better suited for localisation of sites of glycosylation, the CID mass spectra are useful in confirming the nature of the glycan: Each CID mass spectrum of a glycopeptide contains peak(s) corresponding to glycan oxonium ions, see below.

**Table 1:** Glycopeptides identified from tryptic digest of flagellin following ETD MS/MS (with and without FAIMS)

Peptide sequence	Glycan	Site	Charge state	*m/z*_meas_	*m/z*_calc_	Δ ppm	CV	#1	#2
*Trypsin proteolysis and LC MS/MS*									
I**S**TSGEVQFTLK	Δm389	Ser181	3+	566.9626	566.9647	–3.7	n/a	✓	✓
I**S**TSGEVQFTLK	Δm390	Ser181	3+	567.2933	567.2928	0.9	n/a		✓
I**S**TSGEVQFTLK	Δm315^a^	Ser181	3+	542.2829	542.2858	–5.3	n/a	✓	✓
VVI**S**TSVGTGLGALADEINK	Δm389	Ser207	3+	778.4171	778.4197	–3.3	n/a	✓	✓
VVI**S**TSVGTGLGALADEINK	Δm390	Ser207	3+	778.7489	778.4197	1.5	n/a		✓
VVI**S**TSVGTGLGALADEINK	Δm315	Ser207	3+	753.7415	753.7408	0.9	n/a		✓
DILISG**S**NL**S**SAGFGATQFISQASVSLR	Δm389, Δm390	Ser345^a^, Ser349^a^	3+	1202.6097	1202.6072	2.1	n/a		✓
DILISG**S**NL**S**SAGFGATQFISQASVSLR	Δm390, Δm390	Ser345^a^, Ser349^a^	3+	1202.9425	1202.9352	6.1	n/a		✓
**TT**AFGVKDETAGVTTLK	Δm390	Thr464 or Thr465	3+	710.3671	710.3669	0.3	n/a		✓
*Trypsin proteolysis and LC FAIMS MS/MS*									
FETGGRI**S**TSGEVQFTLK	Δm390	Ser181	3+	783.0623	783.0603	2.6	–45	✓	
FETGGRI**S**TSGEVQFTLK	Δm389	Ser181	3+	782.7332	782.7323	1.1	–40 (#1), –45 (#2)	✓	✓
FETGGRI**S**TSGEVQFTLK	Δm315	Ser181^a^	3+	758.0537	758.0534	0.4	–50	✓	
FETGGRI**S**TSGEVQFTLK	Δm316	Ser181^a^	3+	758.3839	758.3814	3.3	–40	✓	
I**S**TSGEVQFTLK	Δm389	Ser181	3+	566.9653	566.9647	1.1	–50 (#1, #2)	✓	✓
I**S**TSGEVQFTLK	Δm390	Ser181	2+	850.4339	850.4355	–1.9	–20 (#1, #2)	✓	✓
I**S**TSGEVQFTLK	Δm315	Ser181	3+	542.2846	542.2858	–2.4	–50		✓
VVI**S**TSVGTGLGALADEINK	Δm389	Ser207	3+	778.4181	778.4198	–2.1	–35		✓
VVI**S**TSVGTGLGALADEINK	Δm390	Ser207	4+,3+	584.3129	584.3126	0.5	–35 (#1, #2)	✓	✓
VVI**S**TSVGTGLGALADEINK	Δm315	Ser207^a^	3+	753.7407	753.7408	–0.1	–30	✓	
VVI**S**TSVGTGLGALADEINKNADK	Δm390	Ser207	3+	921.4810	921.4817	–0.8	–25	✓	
VVI**S**TSVGTGLGALADEINKNADK	Δm389	Ser207	3+	921.1532	921.1537	–0.5	–25	✓	
**TT**AFGVKDETAGVTTLK	Δm390	Thr 464 or Thr465	3+	710.3674	710.3668	0.8	–45 (#1), –40 (#2)	✓	✓
**TT**AFGVKDETAGVTTLK	Δm389	Thr464 or Thr465^a^	3+	710.0390	710.0388	0.3	–40 (#1), –45 (#2)	✓	✓
**TT**AFGVKDETAGVTTLK	Δm315	Thr464 or Thr465^a^	3+	685.3596	685.3599	–0.4	–40 (#1), –50 (#2)	✓	✓

a Indicates novel glycan and/or glycosylation site. Note that where peptides were identified from both replicates, *m/z*_meas_ values are given for replicate#1. Glycosylated residues are underlined and in bold.

Seven of the glycopeptides identified were modified by Δm389 or by Δm390, five of which were at Ser181, Ser207 and Thr 464 or Thr465. These combinations of glycan and modification site were seen in our previous work. Two of the glycopeptides were glycosylated by these glycans at Ser345 and 349. These sites of glycosylation have not been observed previously. Two of the glycopeptides are modified by Δm315, one at Ser181 and one at Ser207. Modification of flagellin A from *Campylobacter jejuni* 11168 by this glycan has not been observed previously.

As observed previously, no sequence coverage was observed between amino acid residues 387 and 463. This region of the protein has a high concentration of serine and threonine residues and may be heavily glycosylated, as observed in *C. jejuni* 81–168 16. The region is also characterised by a scarcity of lysine and arginine residues. Only two large peptides are predicted to result from trypsin proteolysis, one containing 11 and one containing 14 potential O‐glycosylation sites.

### 3.2 Tryspin proteolysis and LC FAIMS MS/MS

Trypsin digests of flagellin were analysed by use of LC FAIMS MS/MS with the top‐4 MS method. An ‘external CV stepping’ FAIMS method 23 was applied in which multiple LC MS/MS analyses are performed at different compensation voltages. The total combined protein sequence coverage obtained for ETD was 81.6%, see Fig. 1. Nineteen glycopeptides were assigned in the protein database search of the ETD data. Manual analysis of the data revealed the presence of 15 glycopeptides, summarised in Table 1. MS/MS spectra are shown in Supporting Information Fig. 3. (Unmodified peptides are summarised in Supporting Information Table 1).

Seven glycopeptides containing Ser181 were identified, four of which were modified by Δm389 or Δm390, as observed previously 18. The missed‐cleavage peptide FETGGRISTSGEVQFTLK modified with Δm315 was identified in the analysis conducted at CV = –50 V. Glycosylation by Δm315 of the same site in the fully tryptic peptide was also observed in this analysis (CV = –50 V) and in the analysis without FAIMS described above. The missed‐cleavage peptide FETGGRISTSGEVQFTLK was also identified as modified by Δm316 in the analysis at CV = –40 V. This modification of flagellin has not been observed previously in *C. jejuni* 11186.

Five glycopeptides containing Ser207 were identified, four of which were modified by Δ389 or Δm390. The remaining peptide was modified by Δm315 at Ser 207. This combination of glycan/glycosylation site has not been observed previously. Finally, three peptides were identified which contained Thr464 and Thr465, modified by Δm389, Δm390 and Δm315. In all cases, although the ETD fragmentation was extensive, no cleavage between the two threonine residues was observed precluding unambiguous site localisation. As seen above, glycosylation of these site(s) by Δm389 and Δm315 are novel observations.

The introduction of FAIMS into the workflow resulted in observation of novel glycans and combinations of glycan/glycosylation sites. Nevertheless, protein coverage between amino acid residues 388 and 463 was not achieved, presumably as a result of the use of trypsin as the protease.

### 3.3 Proteinase K proteolysis and LC MS/MS

To address the problem of the ‘missing region’, i.e. amino acid residues 388–463, an alternative protease—proteinase K —was considered. Two biological replicates were investigated. For the first, proteolysis conditions were enzyme/protein ratio 1:1, 1 h incubation (replicate #1). Subsequent experiments to determine the optimum conditions (to achieve maximum coverage) for proteolysis by proteinase K were performed (data not shown), and conditions found to be enzyme/protein ratio 1:100, 2 h incubation (replicate #2). The proteinase K digest of flagellin was first analysed by the ‘top‐4’ LC MS/MS method without FAIMS. The combined protein sequence coverage obtained for ETD was 18.4%, see Fig. 1. Note that proteinase K has provided access to the ‘missing region’: the sequence coverage in the region [388–463] is 36.6%.

Twenty glycopeptides were assigned in the database search of the ETD data, however manual analysis of the ETD spectra, with cross‐validation against the corresponding CID spectra, revealed 17 true assignments, see Table 2. (Unmodified peptides are summarised in Supporting Information Table 2. Note that no unmodified peptides were identified in replicate #1). ETD MS/MS spectra are shown in Supporting Information Fig. 4. One of the glycopeptides contained Ser181 modified by Δm390. Three contained Ser207 glycosylated with either Δm389 or Δm390. These observations support our earlier work 18. One of the glycopeptides contained Thr465 modified by Δm390. In our earlier work and with trypsin and FAIMS—see above, we showed that either Thr464 or Thr465 was modified, but we were unable to unambiguously assign the glycosylation site. Six of the glycopeptides identified contained the novel glycosylation site Ser343 and were glycosylated with either Δm389 or Δm390. Two of the peptides contained site Ser343 glycosylated with Δm315. The remaining peptides derived from the region inaccessible via trypsin. Peptide [NYSTGFAN] was shown to be modified by Δm389 at Ser423. Peptide [GGYSSVSAY] was multiply modified by Δm315 and Δm390 or Δm389 at sites Ser395 and 298, respectively. Peptide [GSGFSSGSGYSVG] was multiply modified by Δm316, Δm315, Δm389 and Δm389 at sites Ser409, Ser410, Ser412 and Ser415, respectively.

**Table 2:** Glycopeptides identified from proteinase K digest of flagellin following ETD MS/MS (without FAIMS)

Peptide sequence	Glycans	Site	Charge state	*m/z*_meas_	*m/z*_calc_	Δ ppm	CV	#1	#2
*Proteinase K proteolysis and LC MS/MS*									
RFETGGRI**S**TS	Δm390	Ser181	3+	534.2614	534.2653	–7.3	n/a		✓
KVVI**S**TSVGTGL	Δm389	Ser207	3+	517.2893	517.2943	–9.7	n/a		✓
KVVI**S**TSVGT	Δm390	Ser207	3+	460.9164	460.9205	–8.9	n/a		✓
KVVI**S**TSVGT	Δm389	Ser207	3+	460.5884	460.5925	–8.9	n/a		✓
SLVKNDGKDILI**S**GS	Δm389	Ser343^a^	3+	645.6748	645.6809	–9.4	n/a		✓
SLVKNDGKDILI**S**GS	Δm390	Ser343^a^	3+	646.0026	646.0089	–9.6	n/a		✓
DGKDILI**S**GS	Δm389	Ser343^a^	2+	697.3502	697.3565	–9.0	n/a	✓	✓
LVKNDGKDILI**S**GS	Δm389	Ser343^a^	3+	616.6645	616.6702	–9.2	n/a		✓
DGKDILI**S**GS	Δm390	Ser343^a^	2+	697.8419	697.8485	–9.4	n/a	✓	✓
DGKDILI**S**GS	Δm315	Ser343^a^	3+	440.5617	440.5612	1.2	n/a	✓	
NDGKDILI**S**GS	Δm389	Ser343^a^	3+	503.2496	503.2544	–9.5	n/a	✓	✓
NDGKDILI**S**GS	Δm315	Ser343^a^	3+	478.5761	478.5755	1.3	n/a	✓	
GGY**S**SV**S**AY	Δm315, Δm390	Ser395 Ser398^a^	3+	532.5648	532.5702	–9.8	n/a	✓	✓
GGY**S**SV**S**AY	Δm315, Δm389	Ser395 Ser398^a^	3+	532.2426	532.2422	0.8	n/a	✓	
GSGF**SS**G**S**GY**S**VG	Δm316, Δm315, Δm389	Ser409, Ser410, Ser412 Ser415	3+	853.3694	853.3766	–8.4	n/a		✓
NY**S**TGFAN	Δm389	Ser423^a^	2+	631.7742	631.7804	–9.7	n/a		✓
KT**T**AFGVKDETAGVT	Δm390	Thr465^a^	3+	638.9835	638.9896	–9.5	n/a		✓

a Indicates novel glycan and/or glycosylation site. Note that where peptides were identified from both replicates, *m/z*_meas_ values are given for replicate#2. Glycosylated residues are underlined and in bold.

### 3.4 Proteinase K proteolysis and LC FAIMS MS/MS

**Figure 2:**
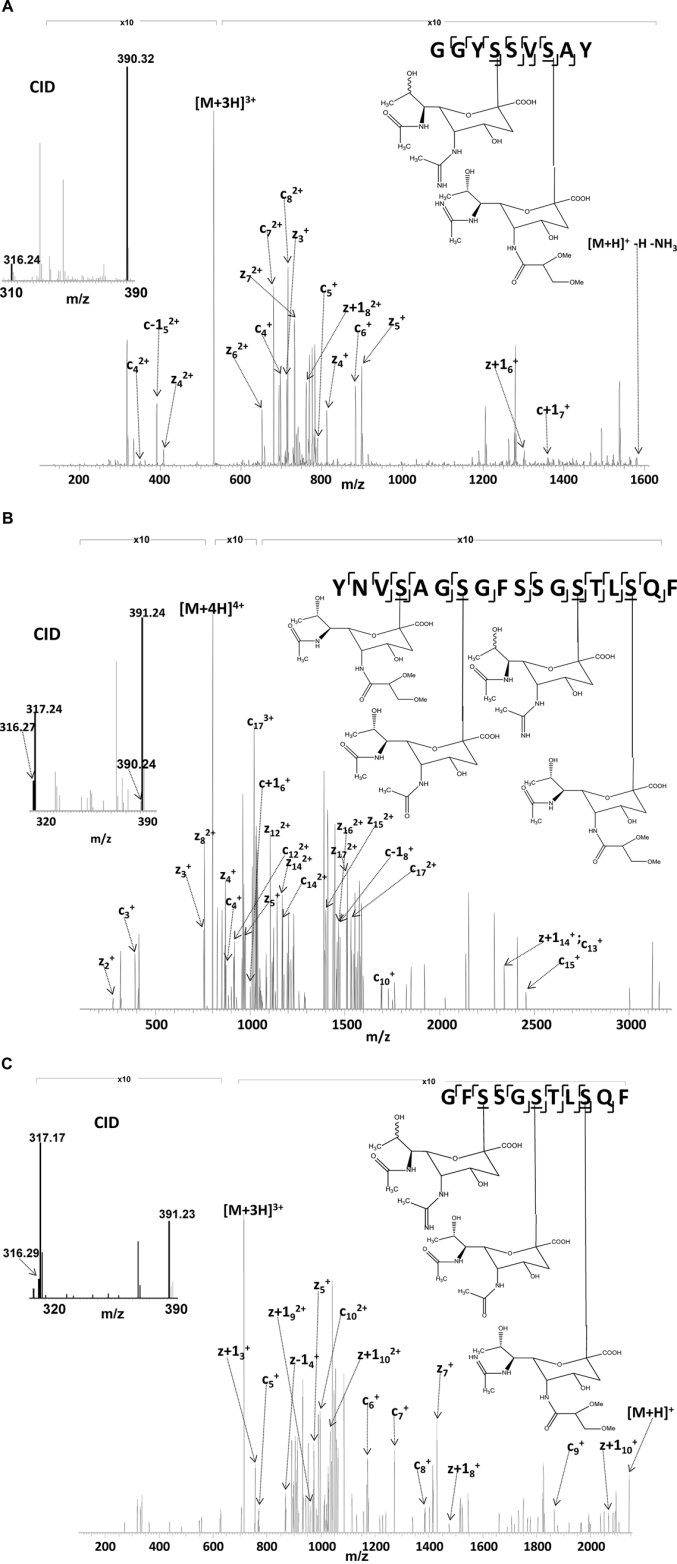
(A) ETD mass spectrum of [M+3H]^3+^ ions of glycopeptide GGYSSVSAY modified at Ser395 and Ser398 with glycans Δm315 and Δm389, respectively. Retention time = 12.88 min. CV = –45 V. Inset: CID mass spectrum showing the oxonium ions for glycans Δm315 and Δm389 at *m/z* 316 and *m/z* 390. (B) ETD mass spectrum of [M+4H]^4+^ ions of glycopeptide YNVSAGSGFSSGSTLSQF modified at Ser445, Ser448, Ser454, and Ser457 modified with glycans Δm390, Δm316, Δm316, and Δm389, respectively (see text for discussion). Retention time = 19.75 min. CV = –40 V. Inset: CID mass spectrum showing the oxonium ions for glycans Δm315, Δm316, Δm389, and Δm390 at *m/z* 316, *m/z* 317, *m/z* 390, and *m/z* 391. (C) ETD mass spectrum of [M+3H]^3+^ ions of glycopeptide GFSSGSTLSQF modified at Ser 452, Ser454, and Ser457 with glycans Δm315, Δm316, and Δm390, respectively. Retention time = 19.82 min. CV = –40 V. Inset: CID mass spectrum showing the oxonium ions for glycans Δm315, Δm316, and Δm390 at *m/z* 316, *m/z* 317, and *m/z* 391.

Proteinase K digests were analysed by LC FAIMS MS/MS (‘external CV stepping’) with the top‐4 MS method. The combined protein sequence coverage obtained for ETD was 39.0%, see Fig. 1. Importantly, the sequence coverage in the ‘missing region’ was 81.5%. The combined coverage from the proteinase K and trypsin LC FAIMS MS/MS analyses was 94.1%. Forty‐four non‐redundant glycopeptides were assigned in the database search; however 37 non‐redundant glycopeptides were confirmed by manual analysis and cross‐validation against corresponding CID mass spectra. See Table 3. (Unmodified peptides are summarised in Supporting Information Table 3. Note that no unmodified peptides were identified in replicate #1). Previously unobserved glycans or glycosylation sites are indicated with an asterisk. Representative ETD MS/MS spectra are shown in Fig. 2 and the remainders are shown in Supporting Information Fig. 5. Figure 2A shows the ETD mass spectrum of triply charged ions of multiply‐glycosylated peptide GGYSSVSAY modified at Ser395 and Ser398 with glycans Δm315 and Δm389, respectively. This peptide is from the region of the protein inaccessible by trypsin digestion. The corresponding CID mass spectrum (shown inset) confirms the nature of the glycans. Oxonium ions are observed at *m/z* 316 and 390. This peptide was observed at compensation voltages –45 V and –50 V (as 3+ precursor). Differential glycosylation of this peptide was observed, i.e. modification at Ser395 and Ser398 with glycans Δm315 and Δm390, respectively. That peptide was observed at CV = –45 V (3+ precursor) and –25 V (2+ precursor).

**Table 3:** Glycopeptides identified from proteinase K digest of flagellin following ETD MS/MS (with FAIMS)

Peptide sequence	Glycans	Site	Charge state	*m/z*_meas_	*m/z*_calc_	Δ ppm	CV	#1	#2
*K proteolysis and LC FAIMS MS/MS*									
FETGGRI**S**TS	Δm390	Ser181	3+	482.2289	482.2316	–5.6	–55		✓
RFETGGRI**S**TS	Δm390	Ser181	2+	800.8937	800.8943	–0.7	–30		✓
RFETGGRI**S**TS	Δm390	Ser181	3+	534.2627	534.2653	–4.9	–40, –50, –55		✓
KVVI**S**TSVGT	Δm390	Ser207	2+	690.8729	690.8770	–6.1	–30		✓
KVVI**S**TSVGT	Δm390	Ser207	3+	460.9183	460.9205	–4.8	–45		✓
KVVI**S**TSVGT	Δm389	Ser207	3+	460.5901	460.5925	–5.2	–40		✓
KVVI**S**TSVGT	Δm315	Ser207^a^	2+	653.8559	653.8587	–4.3	–25		✓
KVVIS**T**SVGT	Δm390	Thr208^a^	3+	460.9181	460.9205	–5.2	–40		✓
KVVI**S**TSVGTGL	Δm390	Ser207	2+	775.9260	775.9298	–4.9	–20, –25		✓
KVVI**S**TSVGTGL	Δm390	Ser207	3+	517.6198	517.6223	–4.8	–40		✓
KVVI**S**TSVGTGL	Δm389	Ser207	3+	517.2915	517.2943	–5.4	–40		✓
GKDILI**S**GS	Δm389	Ser343^a^	2+	639.8397	639.8430	–5.2	–30(#1,#2 35(#1), 40(#1)	✓	✓
GKDILI**S**GS	Δm389	Ser343^a^	3+	426.898	426.898	0.5	–35	✓	
GKDILI**S**GS	Δm315	Ser343^a^	2+	602.8246	602.8246	–0.1	–30	✓	
DGKDILI**S**GS	Δm315	Ser343^a^	3+	440.5618	440.5612	1.4	–40	✓	
DGKDILI**S**GS	Δm390	Ser343^a^	2+	697.8455	697.8485	–4.3	–25(#1), –30(#2)	✓	✓
DGKDILI**S**GS	Δm390	Ser343^a^	3+	465.5656	465.5681	–5.3	–40	✓	✓
DGKDILI**S**GS	Δm389	Ser343^a^	2+	697.3527	697.3565	–5.4	–30	✓	✓
DGKDILI**S**GS	Δm389	Ser343^a^	3+	465.2376	465.2401	–5.4	–40		✓
NDGKDILI**S**GS	Δm390	Ser343^a^	2+	754.8671	754.8700	–3.8	–25		✓
NDGKDILI**S**GS	Δm389	Ser343^a^	3+	503.2518	503.2544	–5.0	–40		✓
VKNDGKDILI**S**GS	Δm390	Ser343^a^	3+	579.3001	579.3035	–5.9	–40, –45		✓
LVKNDGKDILI**S**GS	Δm390	Ser343^a^	2+	924.9881	924.9937	–6.1	–20, –25		✓
LVKNDGKDILI**S**GS	Δm390	Ser343^a^	3+	616.9946	616.9982	–5.8	–35, –40, –45		✓
LVKNDGKDILI**S**GS	Δm389	Ser343^a^	3+	616.6667	616.6702	–5.7	–40		✓
SLVKNDGKDILI**S**GS	Δm390	Ser343^a^	3+	646.0053	646.0089	–5.6	–40, –45		✓
SLVKNDGKDILI**S**GS	Δm389	Ser343^a^	3+	645.6771	645.6809	–5.9	–40, –45		✓
DGKDILI**S**GSNL**SS**A	Δm389, Δm390	Ser343, Ser348^a^	3+	752.7002	752.7041	–5.2	–40		✓
DGKDILI**S**GSNL**S**SA	Δm389	Ser343, Ser348/349^a^	3+	752.3716	752.3761	6.0	–35		✓
DGKDILI**S**GSNL**S**SA	Δm390	Ser343, S348^a^	3+	753.0285	753.0321	–4.7	–40		✓
NDGKDILI**S**GSNL**S**SA	Δm390	Ser343, Ser348^a^	3+	791.0425	791.0464	–4.9	–35		✓
NDGKDILI**S**GSNL**S**SA	Δm390, Δm389	Ser343, Ser348^a^	3+	790.7147	790.7184	–4.6	–30, –35		✓
LVKNDGKDILI**S**GSNLS**S**A	Δm389	Ser343, Ser349^a^	3+	903.8013	903.8062	–5.4	–30		✓
NY**S**TGFAN	Δm389	Ser423^a^	2+	631.7775	631.7804	–4.6	–25		✓
NY**S**TGFAN	Δm390	Ser423^a^	2+	632.2691	632.2724	–5.2	–25		✓
AIAI**S**AA**S**QL	Δm390, Δm315	Ser433, Ser436^a^	3+	550.6179	550.6208	–5.3	–40		✓
GGY**S**SV**S**AY	Δm315, Δm389	Ser395 Ser398^a^	3+	532.2392	532.2421	–5.4	–45(#1,2), –40(#2), –50(#2)	✓	✓
GGY**S**SV**S**AY	Δm315, Δm390	Ser395, Ser398^a^	2+	798.3481	798.3516	–4.4	–25		✓
GGY**S**SV**S**AY	Δm315, Δm390	Ser395, Ser398^a^	3+	532.5664	532.5702	–7.1	–45		✓
**S**AGSGF**SS**GSTL**S**QF	Δm390, Δm315, Δm316, Δm390	Ser445, Ser451, Ser452, Ser457^a^	3+	944.4135	944.4170	–3.7	–30		✓
YNV**S**AG**S**GFSSG**S**TL**S**QF	Δm390, (Δm316 or Δm315), (Δm315 or Δm316), Δm390	Ser445, Ser448, Ser454 Ser457^a^	4+	802.6043	802.6082	–4.9	–40		✓
GFS**S**G**S**TL**S**QF	Δm315, Δm316, Δm 390	Ser452, Ser454, Ser457^a^	3+	713.6513	713.6548	–4.9	–40		✓
GSGF**SS**GSGY	Δm316, Δm315 site undetermined	Ser409, Ser410	2+	768.8207	768.8205	0.3	–35	✓	
SGF**SS**GSTL**S**QF	Δm389, Δm316, Δm390	Ser451, Ser452 Ser457^a^	3+	767.3407	767.3444	–4.8	–40		✓
T**T**AFGVKDETAGVT	Δm390	Thr465	3+	596.2880	596.2913	–5.5	–40		✓

a Indicates novel glycan and/or glycosylation site. Note that where peptides were identified from both replicates, *m/z*_meas_ values are given for replicate#2. Glycosylated residues are underlined and in bold.

The ETD spectrum shown in Fig. 2B was obtained for quadruply charged multiply glycosylated YNVSAGSGFSSGSTLSQF. The precursor ion mass suggests glycan combinations (i) Δm316, Δm316, Δm389 and Δm390; or (ii) Δm315, Δm316, Δm390 and Δm390. Given the well‐established propensity for hydrogen transfer among ETD fragments (z, z+1, c, c‐1), there is scope for ambiguity in the ETD mass spectrum. The corresponding CID mass spectrum reveals oxonium ions at *m/z* 316, 317, 390 and 391 suggesting both species are present; however, the relative abundances of the oxonium ions suggests that (ii) is the predominant species. It is not possible to confirm unambiguously the sites of the Δm315 and Δm316 glycans within the glycopeptide because of the potential for hydrogen transfer between ETD fragments. The glycans are sited on Ser448 and Ser454 (or a combination of the two). Figure 2C shows a third example of a multiply glycosylated peptide from the previously inaccessible region.

**Figure 3:**
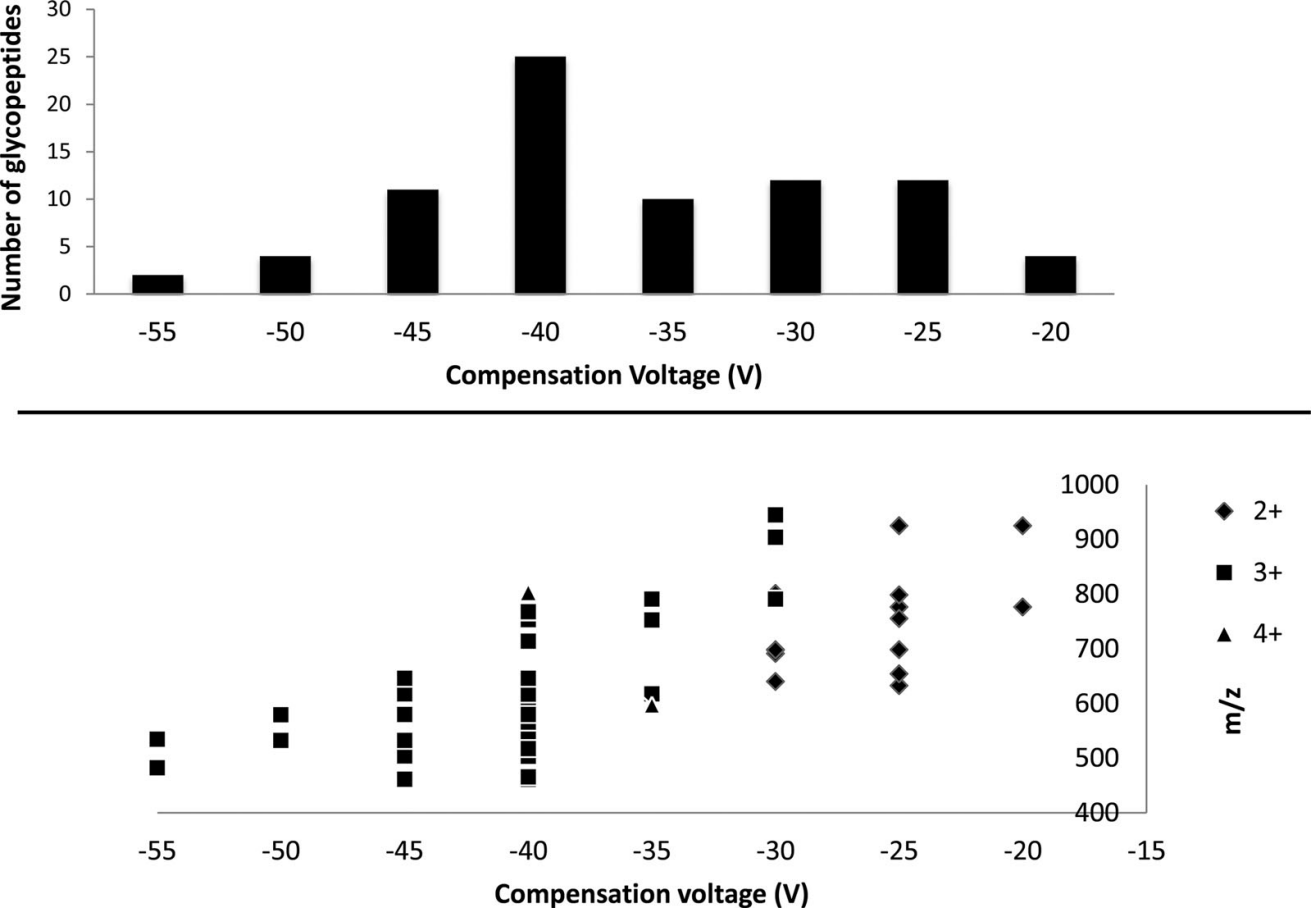
(Top) The number of glycopeptides identified at each of the compensation voltages. (Bottom) Distribution of glycopeptides identified according to charge state.

Figure 3 (top) shows the number of glycopeptides identified at each of the various compensation voltages in replicate #2. The greatest number of identifications was achieved at a compensation voltage of –40 V. The distribution of glycopeptides identified according to charge state is shown in Fig. 3 (bottom). Doubly charged glycopeptides ions were observed between CVs of –20 and –30 V; triply charged glycopeptides between –30 and –55 V. Two 4+ glycopeptides ions were observed at –35 and –40 V. The majority of the identifications were of triply charged precursors.

## 4 Concluding remarks

**Figure 4:**
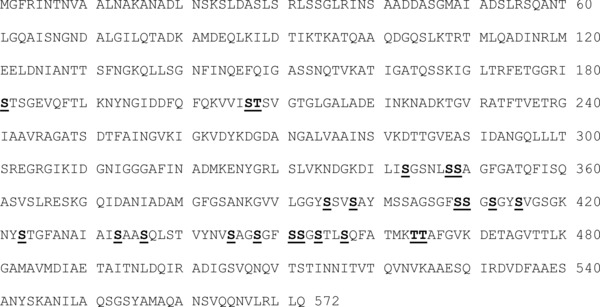
Summary of the sites of glycosylation observed following multienzyme differential ion mobility mass spectrometry of flagellin from *Campylobacter jejuni* 11168.

We have demonstrated that comprehensive mapping of the O‐glycosylation of flagellin from *Campylobacter jejuni* 11168 may be achieved by incorporating differential ion mobility spectrometry into the bottom‐up proteomics workflow, together with use of both trypsin and proteinase K for proteolysis. A summary of the sites of glycosylation observed is given in Fig. 4. Novel glycans for this strain have been identified (pseudaminic acid and either acetamidino pseudaminic acid or legionaminic acid), as have novel glycosylation sites: Thr208, Ser343, Ser348, Ser349, Ser395, Ser398, Ser423, Ser433, Ser436, Ser445, Ser448, Ser451, Ser452, Ser454, Ser457 and Thr465. Multiply and differentially glycosylated peptides were observed: the identity of the glycan at modified amino acid residues was variable, and both presence and absence of glycan at specific residues was also observed. The observed heterogeneity in glycosylation patterns thus appears to confer a combinatorial element of biological variation in the flagellin, potentially advantageous to selective survival of members of the population in the face of biological attack. These results further demonstrate the usefulness of differential ion mobility in proteomics investigations of PTMs.

*The authors have declared no conflict of interest*.

## Supporting information

As a service to our authors and readers, this journal provides supporting information supplied by the authors. Such materials are peer reviewed and may be re‐organized for online delivery, but are not copy‐edited or typeset. Technical support issues arising from supporting information (other than missing files) should be addressed to the authors.

Figure S1. SDS‐PAGE analysis of purified Campylobacter jejuni flagellin protein. 10% SDS‐PAGE gel, stained with Coomassie blue. Lane 1 – MW markers. Lane 2 – cell suspension from C. jejuni strain 11168 culture, Lane 3 – purified flagellin protein
Figure S2.
Figure S3.
Figure S4.
Figure S5.
**Supplemental Table 1**: Non‐glycopeptides identified from tryptic digest of flagellin following ETD MS/MS (with and without FAIMS). (Note that where peptides were identified from both replicates, m/z_meas_ values are given for replicate#1).
**Supplemental Table 2**: Non‐glycopeptides identified from proteinase K digest of flagellin following ETD MS/MS (without FAIMS). (Note that where peptides were identified from both replicates, m/z_meas_ values are given for replicate#2).
**Supplemental Table 3**: Non‐glycopeptides identified from proteinase K digest of flagellin following ETD MS/MS (with FAIMS). (Note that where peptides were identified from both replicates, m/z_meas_ values are given for replicate#2).
Comprehensive mapping of O‐glycosylation in flagellin from Campylobacter jejuni 11168: A multi‐enzyme differential ion mobility mass spectrometry approach
