# Supplementary material for: Comprehensive mapping of O‐glycosylation in flagellin from Campylobacter jejuni 11168: A multienzyme differential ion mobility mass spectrometry approach
Source: Proteomics. 2015 Jun 15;15(16):2733–45. doi: 10.1002/pmic.201400533 (PMC4975691; doi:10.1002/pmic.201400533)
Supplement: Supplementary file 1 — Figure S1. SDS‐PAGE analysis of purified Campylobacter jejuni flagellin protein. 10% SDS‐PAGE gel, stained with Coomassie blue. Lane 1 – MW markers. Lane 2 – cell suspension from C. jejuni strain 11168 culture, Lane 3 – purified flagellin protein Figure S2. Figure S3. Figure S4. Figure S5. Supplemental Table 1: Non‐glycopeptides identified from tryptic digest of flagellin following ETD MS/MS (with and without FAIMS). (Note that where peptides were identified from both replicates, m/zmeas values are given for replicate#1). Supplemental Table 2: Non‐glycopeptides identified from proteinase K digest of flagellin following ETD MS/MS (without FAIMS). (Note that where peptides were identified from both replicates, m/zmeas values are given for replicate#2). Supplemental Table 3: Non‐glycopeptides identified from proteinase K digest of flagellin following ETD MS/MS (with FAIMS). (Note that where peptides were identified from both replicates, m/zmeas values are given for replicate#2). Comprehensive mapping of O‐glycosylation in flagellin from Campylobacter jejuni 11168: A multi‐enzyme differential ion mobility mass spectrometry approach [file PMIC-15-2733-s001.zip › pmic12013-sup-0008-legend.docx]

**Comprehensive mapping of O-glycosylation in flagellin from *Campylobacter jejuni* 11168: A multi-enzyme differential ion mobility mass spectrometry approach**

Gloria N. Ulasi, Andrew J. Creese, Charles W. Penn and Helen J. Cooper*

School of Biosciences, University of Birmingham, Edgbaston, Birmingham B15 2TT, UK.

**Supplemental information**

**Supplemental Figures:**

**Supplemental figure 1:** SDS-PAGE analysis of purified *Campylobacter jejuni* flagellin protein. 10% SDS-PAGE gel, stained with Coomassie blue. Lane 1 – MW markers. Lane 2 – cell suspension from *C. jejuni* strain 11168 culture, Lane 3 – purified flagellin protein.

**Supplemental figure 2:** ETD mass spectra of non-redundant glycopeptides identified following treatment with trypsin (without FAIMS).

**Supplemental figure 3:** ETD mass spectra of non-redundant glycopeptides identified following treatment with trypsin (with FAIMS).

**Supplemental figure 4:** ETD mass spectra of non-redundant glycopeptides identified following treatment with proteinase K (without FAIMS).

**Supplemental figure 5:** ETD mass spectra of non-redundant glycopeptides identified following treatment with proteinase K (with FAIMS).

**Supplemental Tables:**

**Supplemental Table 1:** Non-glycopeptides identified from tryptic digest of flagellin following ETD MS/MS (with and without FAIMS). (Note that where peptides were identified from both replicates, m/z_meas_ values are given for replicate#1).

**Supplemental Table 2:** Non-glycopeptides identified from proteinase K digest of flagellin following ETD MS/MS (without FAIMS). (Note that where peptides were identified from both replicates, m/z_meas_ values are given for replicate#2).

**Supplemental** **Table 3:** Non-glycopeptides identified from proteinase K digest of flagellin following ETD MS/MS (with FAIMS). (Note that where peptides were identified from both replicates, m/z_meas_ values are given for replicate#2).
