# Supplementary material for: Comprehensive mapping of O‐glycosylation in flagellin from Campylobacter jejuni 11168: A multienzyme differential ion mobility mass spectrometry approach
Source: Proteomics. 2015 Jun 15;15(16):2733–45. doi: 10.1002/pmic.201400533 (PMC4975691; doi:10.1002/pmic.201400533)
Supplement: Supplementary file 1 — Figure S1. SDS‐PAGE analysis of purified Campylobacter jejuni flagellin protein. 10% SDS‐PAGE gel, stained with Coomassie blue. Lane 1 – MW markers. Lane 2 – cell suspension from C. jejuni strain 11168 culture, Lane 3 – purified flagellin protein Figure S2. Figure S3. Figure S4. Figure S5. Supplemental Table 1: Non‐glycopeptides identified from tryptic digest of flagellin following ETD MS/MS (with and without FAIMS). (Note that where peptides were identified from both replicates, m/zmeas values are given for replicate#1). Supplemental Table 2: Non‐glycopeptides identified from proteinase K digest of flagellin following ETD MS/MS (without FAIMS). (Note that where peptides were identified from both replicates, m/zmeas values are given for replicate#2). Supplemental Table 3: Non‐glycopeptides identified from proteinase K digest of flagellin following ETD MS/MS (with FAIMS). (Note that where peptides were identified from both replicates, m/zmeas values are given for replicate#2). Comprehensive mapping of O‐glycosylation in flagellin from Campylobacter jejuni 11168: A multi‐enzyme differential ion mobility mass spectrometry approach [file PMIC-15-2733-s001.zip › pmic201400533-sup-0001-figure 1.pptx]

## Slide 1
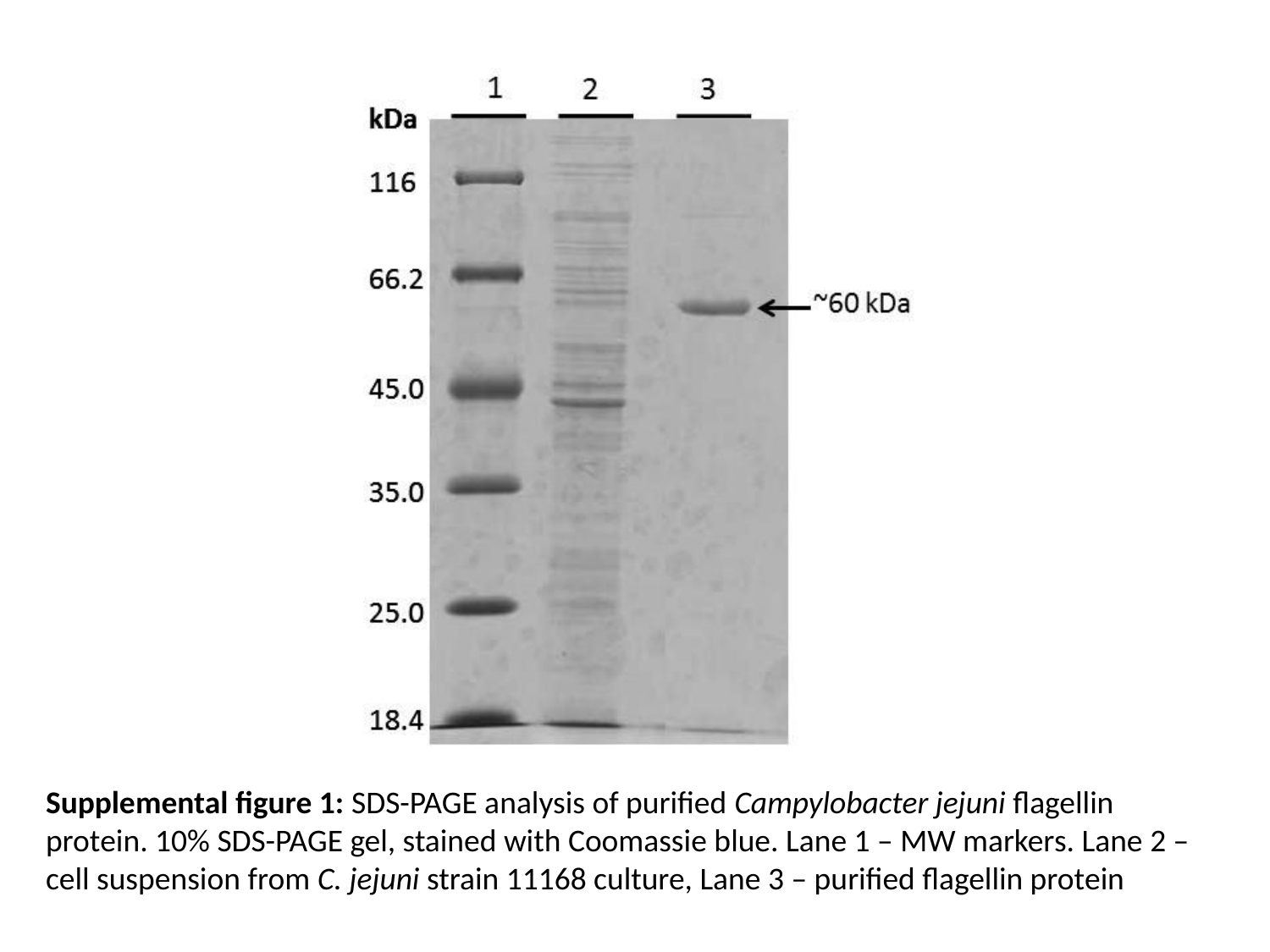

Supplemental figure 1: SDS-PAGE analysis of purified Campylobacter jejuni flagellin protein. 10% SDS-PAGE gel, stained with Coomassie blue. Lane 1 – MW markers. Lane 2 – cell suspension from C. jejuni strain 11168 culture, Lane 3 – purified flagellin protein
