# Supplementary material for: Comprehensive mapping of O‐glycosylation in flagellin from Campylobacter jejuni 11168: A multienzyme differential ion mobility mass spectrometry approach
Source: Proteomics. 2015 Jun 15;15(16):2733–45. doi: 10.1002/pmic.201400533 (PMC4975691; doi:10.1002/pmic.201400533)
Supplement: Supplementary file 1 — Figure S1. SDS‐PAGE analysis of purified Campylobacter jejuni flagellin protein. 10% SDS‐PAGE gel, stained with Coomassie blue. Lane 1 – MW markers. Lane 2 – cell suspension from C. jejuni strain 11168 culture, Lane 3 – purified flagellin protein Figure S2. Figure S3. Figure S4. Figure S5. Supplemental Table 1: Non‐glycopeptides identified from tryptic digest of flagellin following ETD MS/MS (with and without FAIMS). (Note that where peptides were identified from both replicates, m/zmeas values are given for replicate#1). Supplemental Table 2: Non‐glycopeptides identified from proteinase K digest of flagellin following ETD MS/MS (without FAIMS). (Note that where peptides were identified from both replicates, m/zmeas values are given for replicate#2). Supplemental Table 3: Non‐glycopeptides identified from proteinase K digest of flagellin following ETD MS/MS (with FAIMS). (Note that where peptides were identified from both replicates, m/zmeas values are given for replicate#2). Comprehensive mapping of O‐glycosylation in flagellin from Campylobacter jejuni 11168: A multi‐enzyme differential ion mobility mass spectrometry approach [file PMIC-15-2733-s001.zip › pmic201400533-sup-0002-figure 2.pptx]

## Slide 1
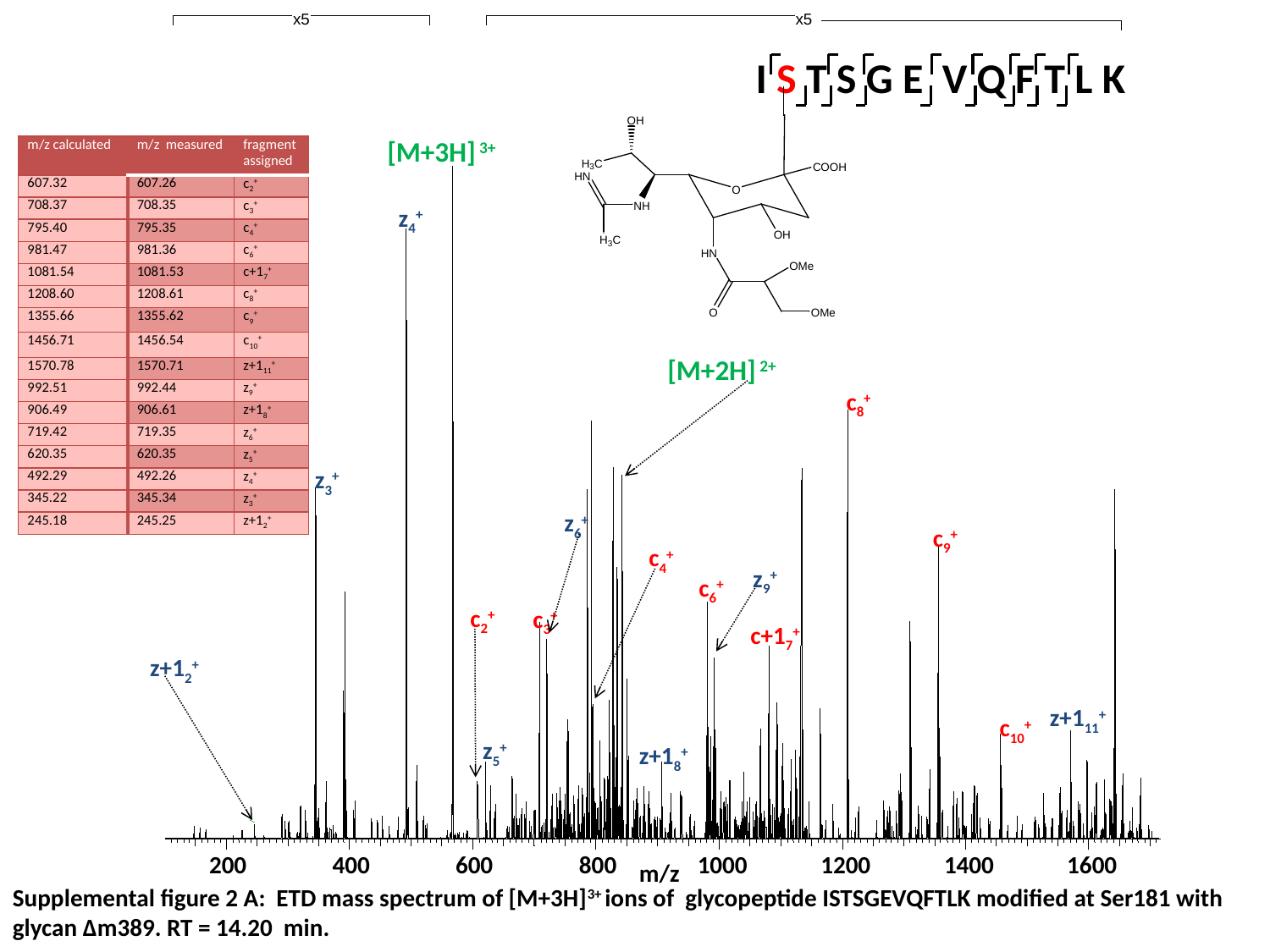

x5
x5
 I S T S G E V Q F T L K
[M+3H] 3+
| m/z calculated | m/z measured | fragment assigned |
| --- | --- | --- |
| 607.32 | 607.26 | c2+ |
| 708.37 | 708.35 | c3+ |
| 795.40 | 795.35 | c4+ |
| 981.47 | 981.36 | c6+ |
| 1081.54 | 1081.53 | c+17+ |
| 1208.60 | 1208.61 | c8+ |
| 1355.66 | 1355.62 | c9+ |
| 1456.71 | 1456.54 | c10+ |
| 1570.78 | 1570.71 | z+111+ |
| 992.51 | 992.44 | z9+ |
| 906.49 | 906.61 | z+18+ |
| 719.42 | 719.35 | z6+ |
| 620.35 | 620.35 | z5+ |
| 492.29 | 492.26 | z4+ |
| 345.22 | 345.34 | z3+ |
| 245.18 | 245.25 | z+12+ |
z4+
[M+2H] 2+
c8+
z3+
z6+
c9+
c4+
z9+
c6+
c2+
c3+
c+17+
z+12+
z+111+
c10+
z5+
z+18+
200
400
600
800
1000
1200
1400
1600
m/z
Supplemental figure 2 A: ETD mass spectrum of [M+3H]3+ ions of glycopeptide ISTSGEVQFTLK modified at Ser181 with glycan Δm389. RT = 14.20 min.

## Slide 2
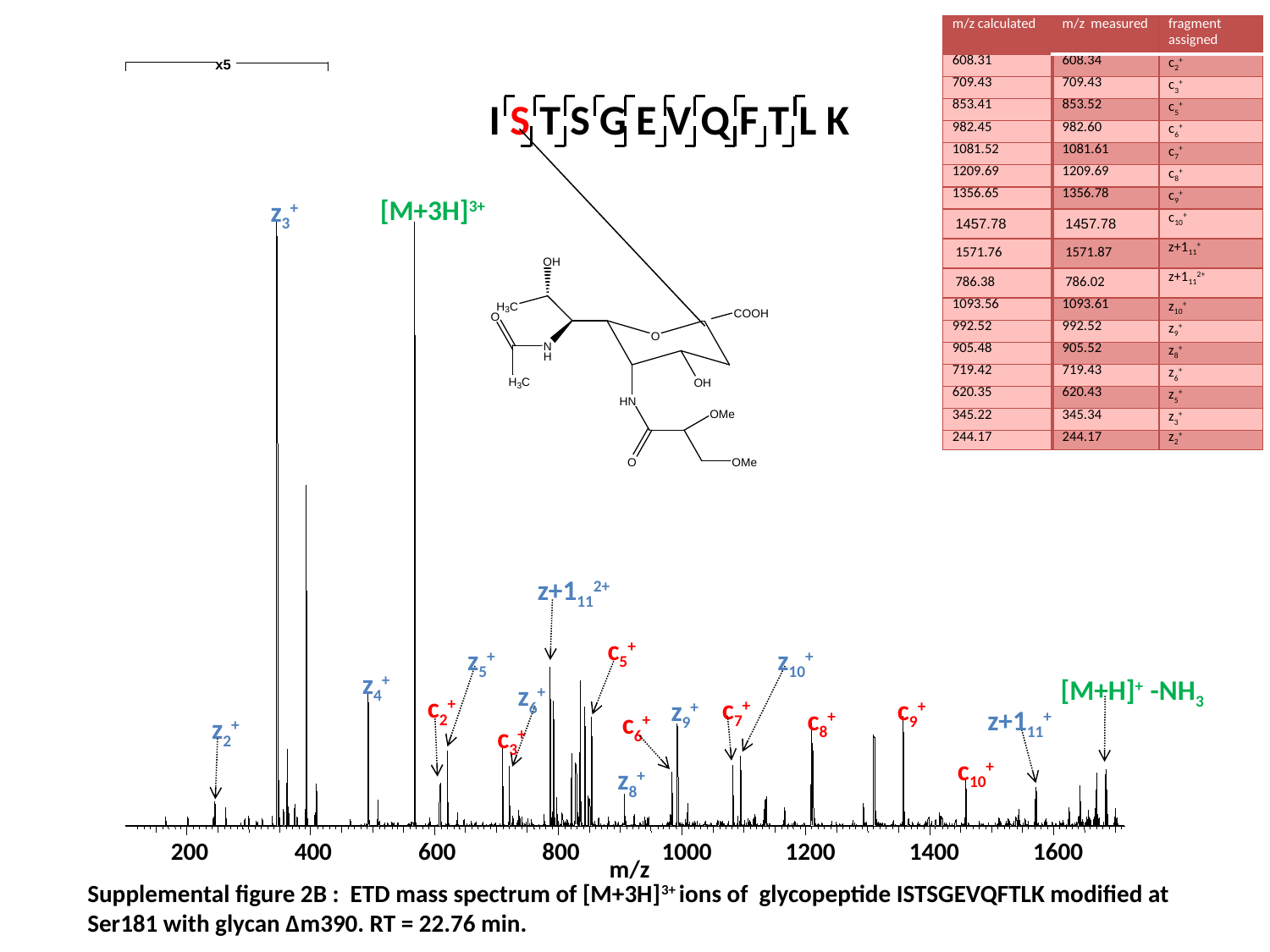

| m/z calculated | m/z measured | fragment assigned |
| --- | --- | --- |
| 608.31 | 608.34 | c2+ |
| 709.43 | 709.43 | c3+ |
| 853.41 | 853.52 | c5+ |
| 982.45 | 982.60 | c6+ |
| 1081.52 | 1081.61 | c7+ |
| 1209.69 | 1209.69 | c8+ |
| 1356.65 | 1356.78 | c9+ |
| 1457.78 | 1457.78 | c10+ |
| 1571.76 | 1571.87 | z+111+ |
| 786.38 | 786.02 | z+1112+ |
| 1093.56 | 1093.61 | z10+ |
| 992.52 | 992.52 | z9+ |
| 905.48 | 905.52 | z8+ |
| 719.42 | 719.43 | z6+ |
| 620.35 | 620.43 | z5+ |
| 345.22 | 345.34 | z3+ |
| 244.17 | 244.17 | z2+ |
x5
I S T S G E V Q F T L K
[M+3H]3+
z3+
z+1112+
c5+
z5+
z10+
z4+
[M+H]+ -NH3
z6+
c2+
c7+
c9+
z9+
c8+
z+111+
c6+
z2+
c3+
c10+
z8+
200
400
600
800
1000
1200
1400
1600
m/z
Supplemental figure 2B : ETD mass spectrum of [M+3H]3+ ions of glycopeptide ISTSGEVQFTLK modified at Ser181 with glycan Δm390. RT = 22.76 min.

## Slide 3
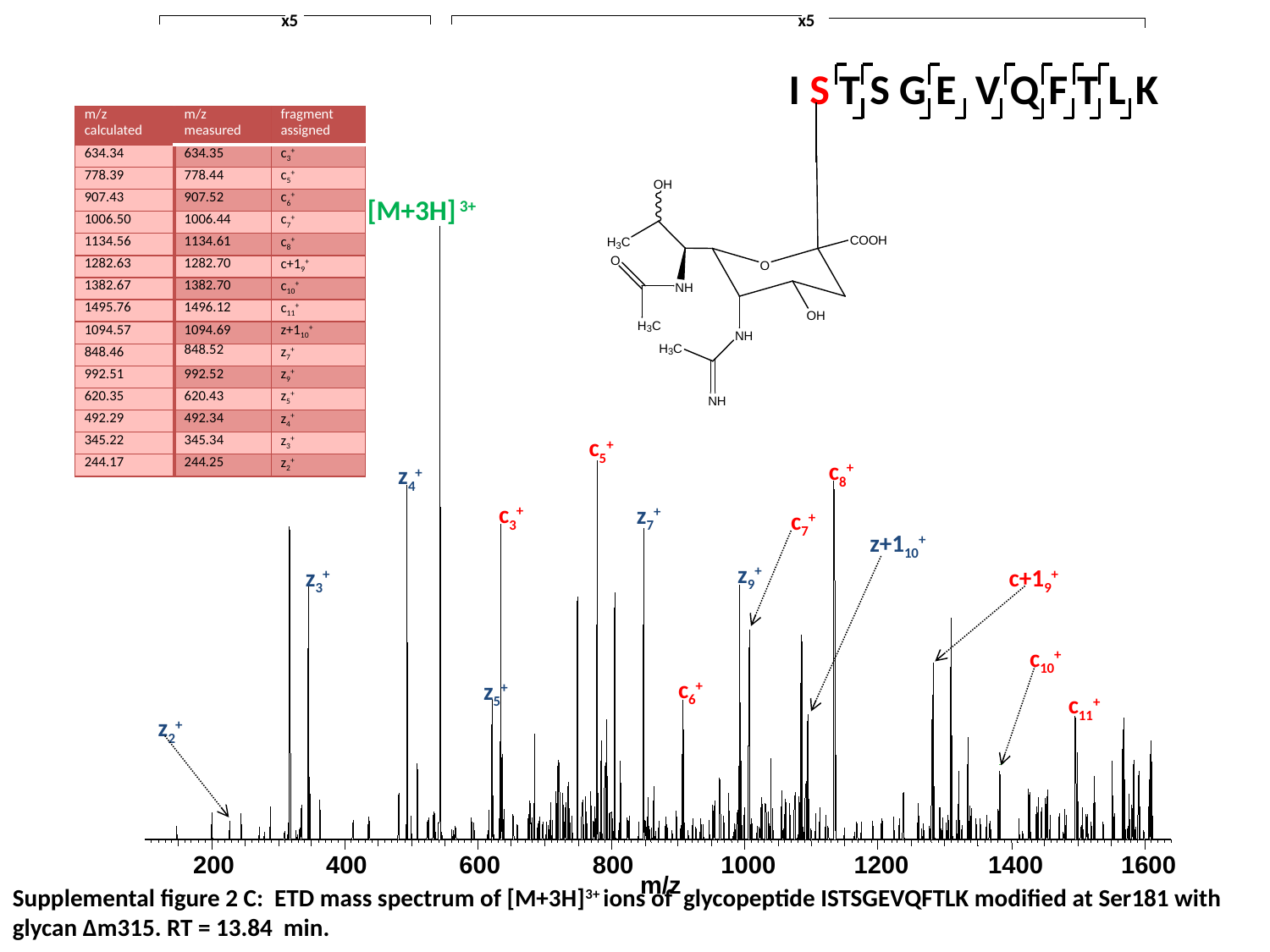

x5
x5
 I S T S G E V Q F T L K
| m/z calculated | m/z measured | fragment assigned |
| --- | --- | --- |
| 634.34 | 634.35 | c3+ |
| 778.39 | 778.44 | c5+ |
| 907.43 | 907.52 | c6+ |
| 1006.50 | 1006.44 | c7+ |
| 1134.56 | 1134.61 | c8+ |
| 1282.63 | 1282.70 | c+19+ |
| 1382.67 | 1382.70 | c10+ |
| 1495.76 | 1496.12 | c11+ |
| 1094.57 | 1094.69 | z+110+ |
| 848.46 | 848.52 | z7+ |
| 992.51 | 992.52 | z9+ |
| 620.35 | 620.43 | z5+ |
| 492.29 | 492.34 | z4+ |
| 345.22 | 345.34 | z3+ |
| 244.17 | 244.25 | z2+ |
[M+3H] 3+
c5+
c8+
z4+
c3+
z7+
c7+
z+110+
z9+
z3+
c+19+
c10+
c6+
z5+
c11+
z2+
200
400
600
800
1000
1200
1400
1600
m/z
Supplemental figure 2 C: ETD mass spectrum of [M+3H]3+ ions of glycopeptide ISTSGEVQFTLK modified at Ser181 with glycan Δm315. RT = 13.84 min.

## Slide 4
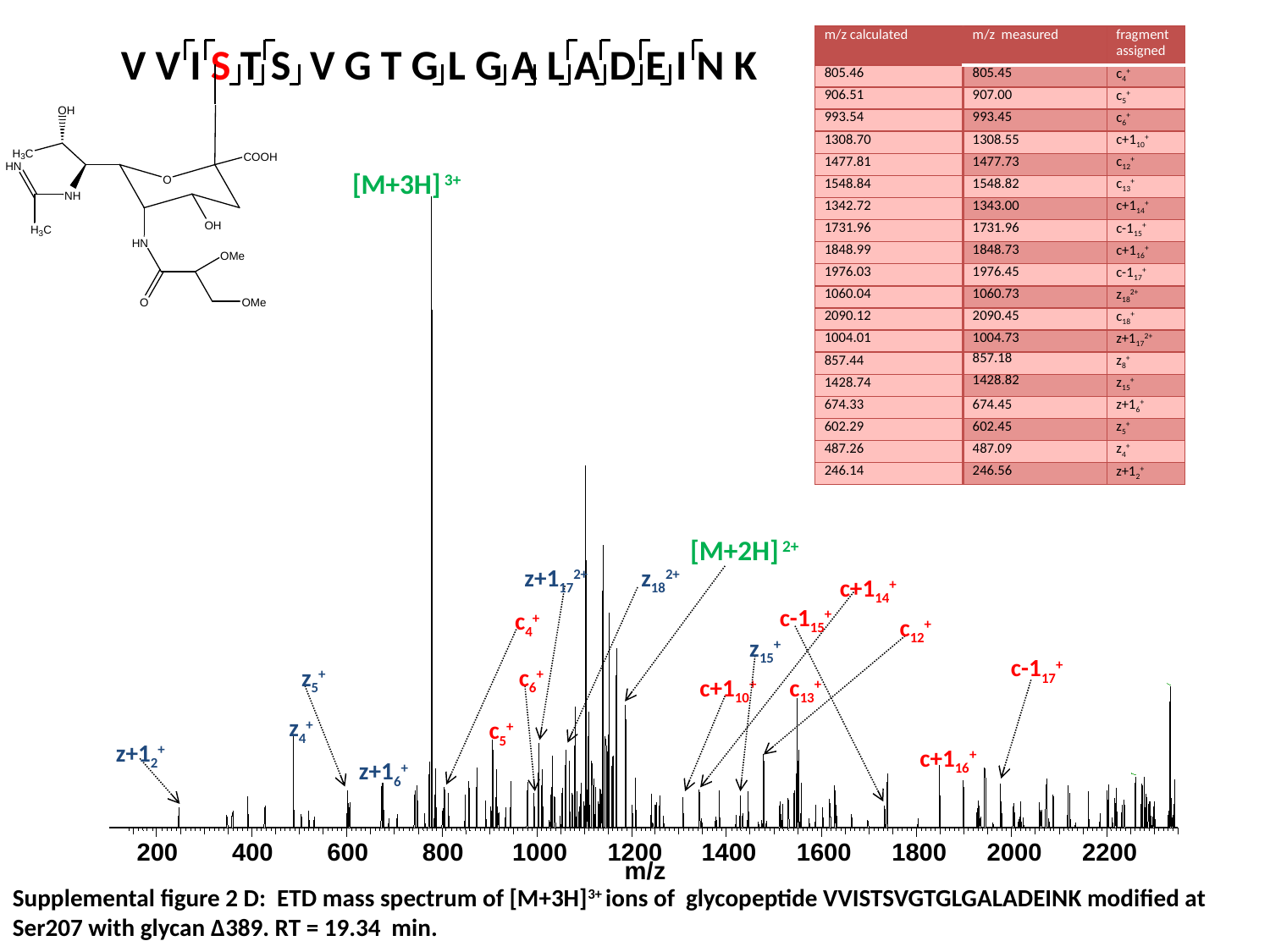

| m/z calculated | m/z measured | fragment assigned |
| --- | --- | --- |
| 805.46 | 805.45 | c4+ |
| 906.51 | 907.00 | c5+ |
| 993.54 | 993.45 | c6+ |
| 1308.70 | 1308.55 | c+110+ |
| 1477.81 | 1477.73 | c12+ |
| 1548.84 | 1548.82 | c13+ |
| 1342.72 | 1343.00 | c+114+ |
| 1731.96 | 1731.96 | c-115+ |
| 1848.99 | 1848.73 | c+116+ |
| 1976.03 | 1976.45 | c-117+ |
| 1060.04 | 1060.73 | z182+ |
| 2090.12 | 2090.45 | c18+ |
| 1004.01 | 1004.73 | z+1172+ |
| 857.44 | 857.18 | z8+ |
| 1428.74 | 1428.82 | z15+ |
| 674.33 | 674.45 | z+16+ |
| 602.29 | 602.45 | z5+ |
| 487.26 | 487.09 | z4+ |
| 246.14 | 246.56 | z+12+ |
V V I S T S V G T G L G A L A D E I N K
[M+3H] 3+
[M+2H] 2+
z+1172+
z182+
c+114+
c-115+
c4+
c12+
z15+
c-117+
z5+
c6+
c+110+
c13+
z4+
c5+
z+12+
c+116+
z+16+
200
400
600
800
1000
1200
1400
1600
1800
2000
2200
m/z
Supplemental figure 2 D: ETD mass spectrum of [M+3H]3+ ions of glycopeptide VVISTSVGTGLGALADEINK modified at Ser207 with glycan Δ389. RT = 19.34 min.

## Slide 5
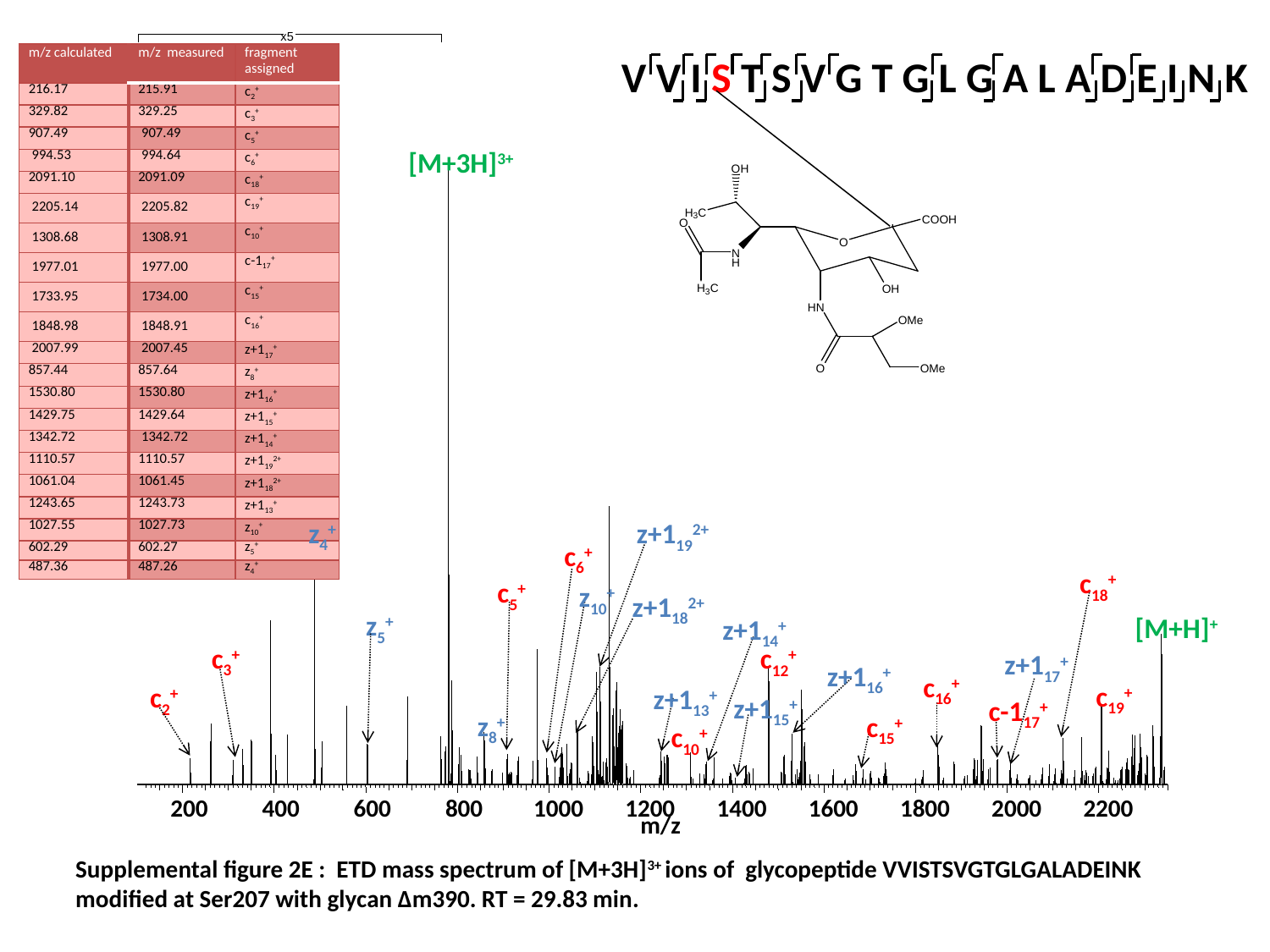

x5
| m/z calculated | m/z measured | fragment assigned |
| --- | --- | --- |
| 216.17 | 215.91 | c2+ |
| 329.82 | 329.25 | c3+ |
| 907.49 | 907.49 | c5+ |
| 994.53 | 994.64 | c6+ |
| 2091.10 | 2091.09 | c18+ |
| 2205.14 | 2205.82 | c19+ |
| 1308.68 | 1308.91 | c10+ |
| 1977.01 | 1977.00 | c-117+ |
| 1733.95 | 1734.00 | c15+ |
| 1848.98 | 1848.91 | c16+ |
| 2007.99 | 2007.45 | z+117+ |
| 857.44 | 857.64 | z8+ |
| 1530.80 | 1530.80 | z+116+ |
| 1429.75 | 1429.64 | z+115+ |
| 1342.72 | 1342.72 | z+114+ |
| 1110.57 | 1110.57 | z+1192+ |
| 1061.04 | 1061.45 | z+1182+ |
| 1243.65 | 1243.73 | z+113+ |
| 1027.55 | 1027.73 | z10+ |
| 602.29 | 602.27 | z5+ |
| 487.36 | 487.26 | z4+ |
V V I S T S V G T G L G A L A D E I N K
[M+3H]3+
z4+
z+1192+
c6+
c18+
c5+
z10+
z+1182+
z5+
[M+H]+
z+114+
c3+
c12+
z+117+
z+116+
c16+
c19+
c2+
z+113+
z+115+
c-117+
z8+
c15+
c10+
200
400
600
800
1000
1200
1400
1600
1800
2000
2200
m/z
Supplemental figure 2E : ETD mass spectrum of [M+3H]3+ ions of glycopeptide VVISTSVGTGLGALADEINK modified at Ser207 with glycan Δm390. RT = 29.83 min.

## Slide 6
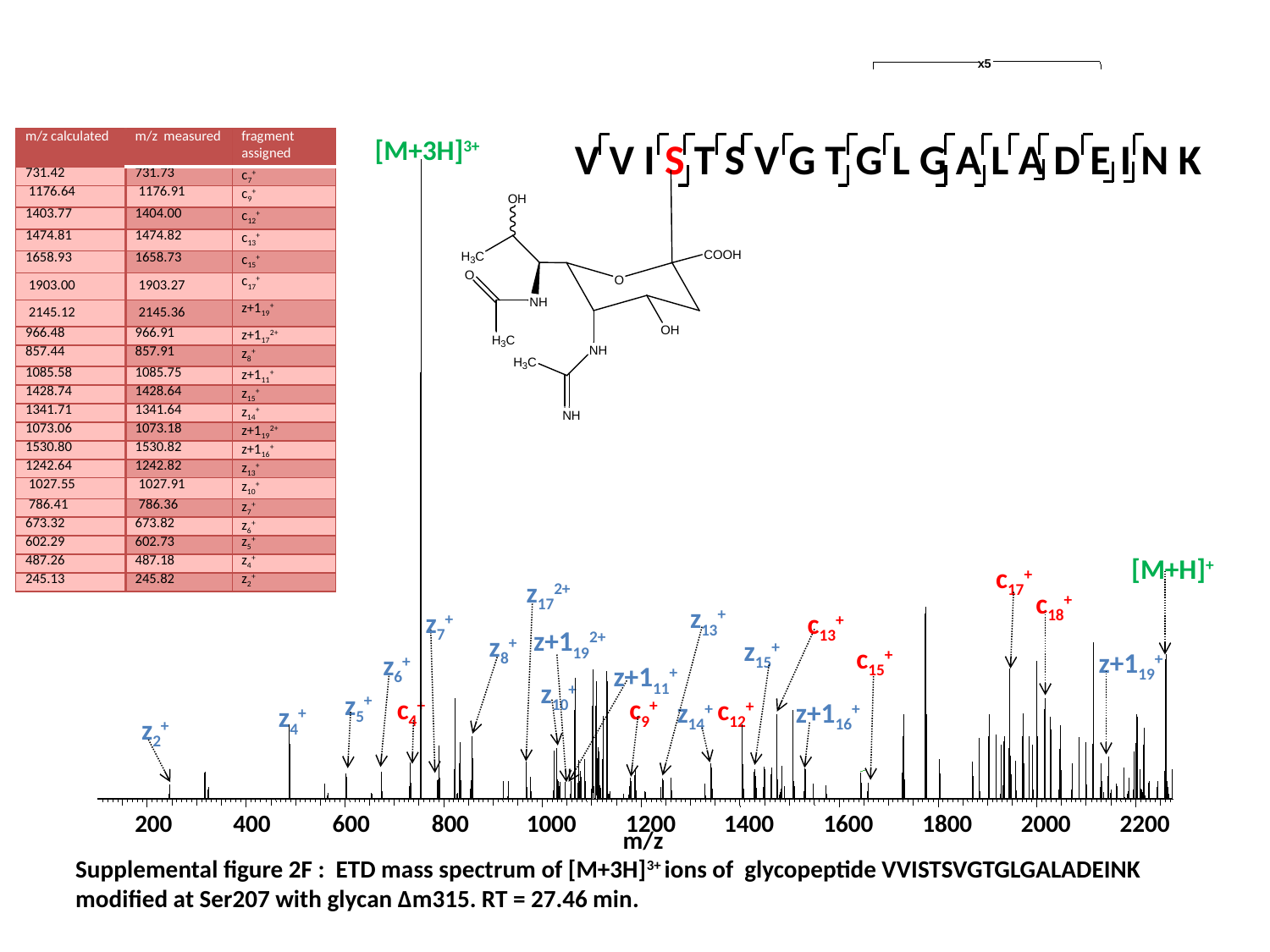

x5
[M+3H]3+
V V I S T S V G T G L G A L A D E I N K
| m/z calculated | m/z measured | fragment assigned |
| --- | --- | --- |
| 731.42 | 731.73 | c7+ |
| 1176.64 | 1176.91 | c9+ |
| 1403.77 | 1404.00 | c12+ |
| 1474.81 | 1474.82 | c13+ |
| 1658.93 | 1658.73 | c15+ |
| 1903.00 | 1903.27 | c17+ |
| 2145.12 | 2145.36 | z+119+ |
| 966.48 | 966.91 | z+1172+ |
| 857.44 | 857.91 | z8+ |
| 1085.58 | 1085.75 | z+111+ |
| 1428.74 | 1428.64 | z15+ |
| 1341.71 | 1341.64 | z14+ |
| 1073.06 | 1073.18 | z+1192+ |
| 1530.80 | 1530.82 | z+116+ |
| 1242.64 | 1242.82 | z13+ |
| 1027.55 | 1027.91 | z10+ |
| 786.41 | 786.36 | z7+ |
| 673.32 | 673.82 | z6+ |
| 602.29 | 602.73 | z5+ |
| 487.26 | 487.18 | z4+ |
| 245.13 | 245.82 | z2+ |
[M+H]+
c17+
z172+
c18+
z13+
z7+
c13+
z+1192+
z8+
z15+
c15+
z+119+
z6+
z+111+
z10+
z5+
c4+
c9+
c12+
z+116+
z14+
z4+
z2+
200
400
600
800
1000
1200
1400
1600
1800
2000
2200
m/z
Supplemental figure 2F : ETD mass spectrum of [M+3H]3+ ions of glycopeptide VVISTSVGTGLGALADEINK modified at Ser207 with glycan Δm315. RT = 27.46 min.

## Slide 7
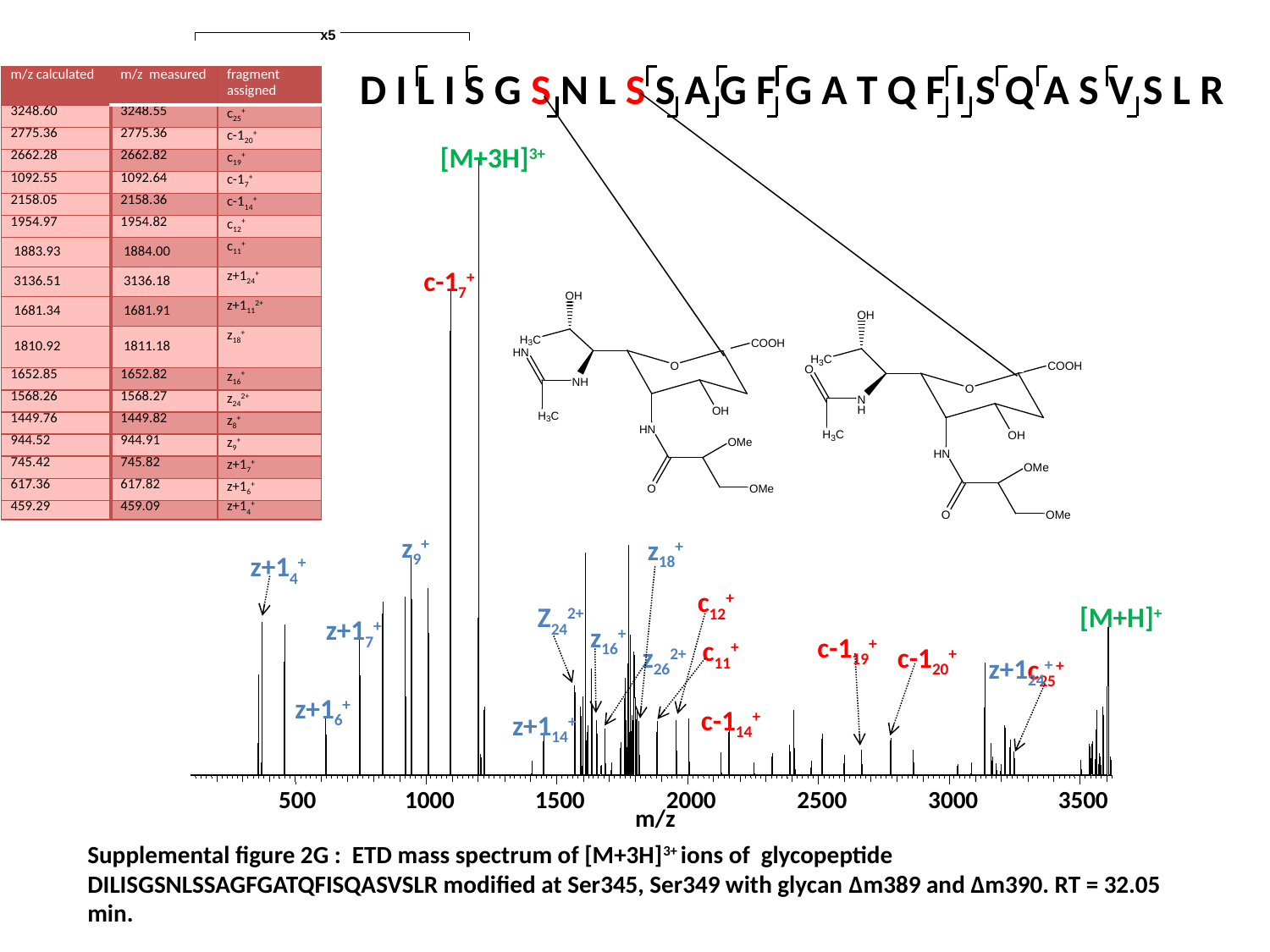

x5
D I L I S G S N L S S A G F G A T Q F I S Q A S V S L R
| m/z calculated | m/z measured | fragment assigned |
| --- | --- | --- |
| 3248.60 | 3248.55 | c25+ |
| 2775.36 | 2775.36 | c-120+ |
| 2662.28 | 2662.82 | c19+ |
| 1092.55 | 1092.64 | c-17+ |
| 2158.05 | 2158.36 | c-114+ |
| 1954.97 | 1954.82 | c12+ |
| 1883.93 | 1884.00 | c11+ |
| 3136.51 | 3136.18 | z+124+ |
| 1681.34 | 1681.91 | z+1112+ |
| 1810.92 | 1811.18 | z18+ |
| 1652.85 | 1652.82 | z16+ |
| 1568.26 | 1568.27 | z242+ |
| 1449.76 | 1449.82 | z8+ |
| 944.52 | 944.91 | z9+ |
| 745.42 | 745.82 | z+17+ |
| 617.36 | 617.82 | z+16+ |
| 459.29 | 459.09 | z+14+ |
[M+3H]3+
c-17+
z9+
z18+
z+14+
c12+
Z242+
[M+H]+
z+17+
z16+
c-119+
c11+
z262+
c-120+
z+124+
c25+
z+16+
c-114+
z+114+
500
1000
1500
2000
2500
3000
3500
m/z
Supplemental figure 2G : ETD mass spectrum of [M+3H]3+ ions of glycopeptide DILISGSNLSSAGFGATQFISQASVSLR modified at Ser345, Ser349 with glycan Δm389 and Δm390. RT = 32.05 min.

## Slide 8
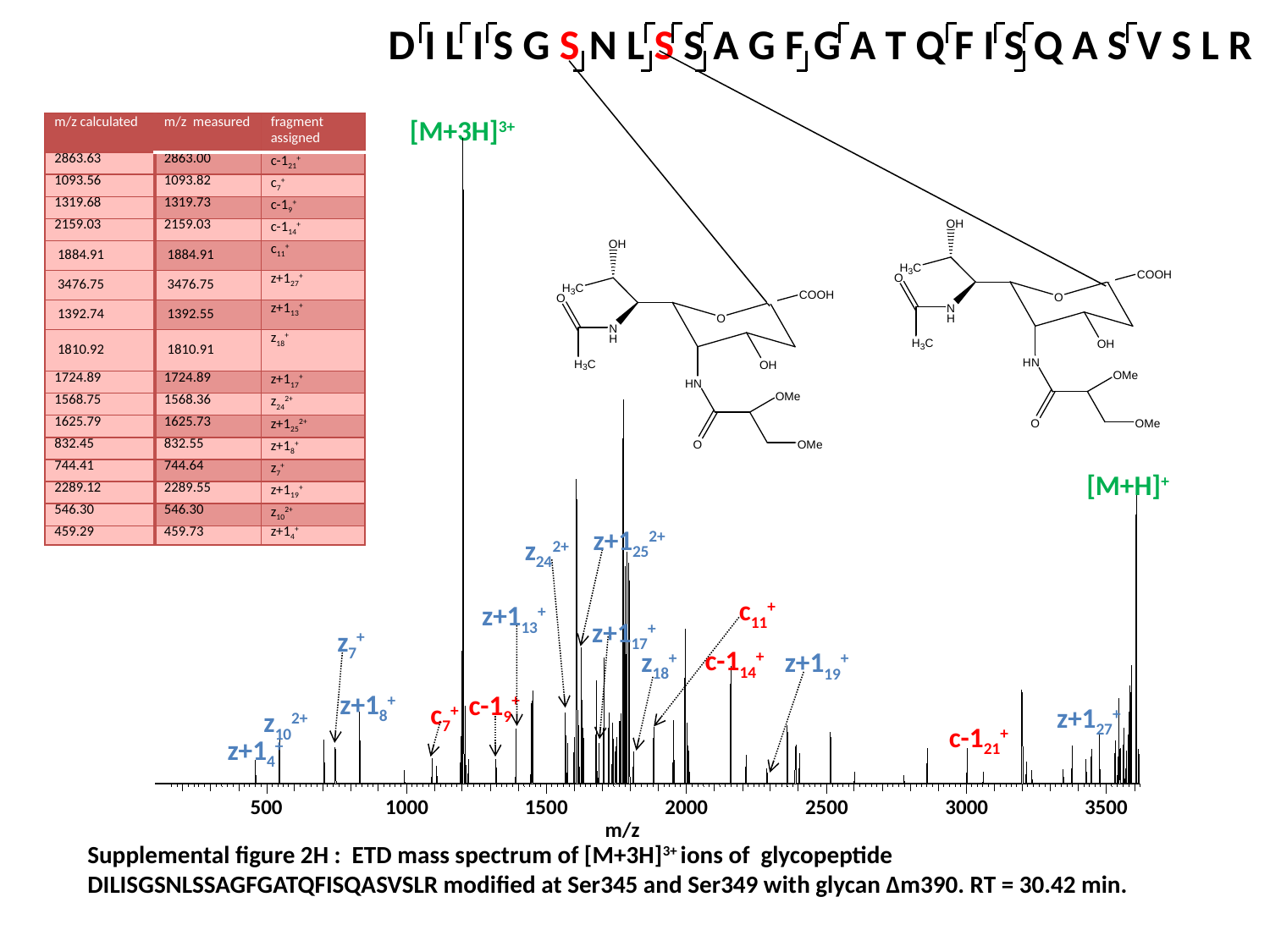

D I L I S G S N L S S A G F G A T Q F I S Q A S V S L R
[M+3H]3+
| m/z calculated | m/z measured | fragment assigned |
| --- | --- | --- |
| 2863.63 | 2863.00 | c-121+ |
| 1093.56 | 1093.82 | c7+ |
| 1319.68 | 1319.73 | c-19+ |
| 2159.03 | 2159.03 | c-114+ |
| 1884.91 | 1884.91 | c11+ |
| 3476.75 | 3476.75 | z+127+ |
| 1392.74 | 1392.55 | z+113+ |
| 1810.92 | 1810.91 | z18+ |
| 1724.89 | 1724.89 | z+117+ |
| 1568.75 | 1568.36 | z242+ |
| 1625.79 | 1625.73 | z+1252+ |
| 832.45 | 832.55 | z+18+ |
| 744.41 | 744.64 | z7+ |
| 2289.12 | 2289.55 | z+119+ |
| 546.30 | 546.30 | z102+ |
| 459.29 | 459.73 | z+14+ |
[M+H]+
z+1252+
z242+
c11+
z+113+
z+117+
z7+
c-114+
z18+
z+119+
z+18+
c-19+
c7+
z+127+
z102+
c-121+
z+14+
500
1000
1500
2000
2500
3000
3500
m/z
Supplemental figure 2H : ETD mass spectrum of [M+3H]3+ ions of glycopeptide DILISGSNLSSAGFGATQFISQASVSLR modified at Ser345 and Ser349 with glycan Δm390. RT = 30.42 min.

## Slide 9
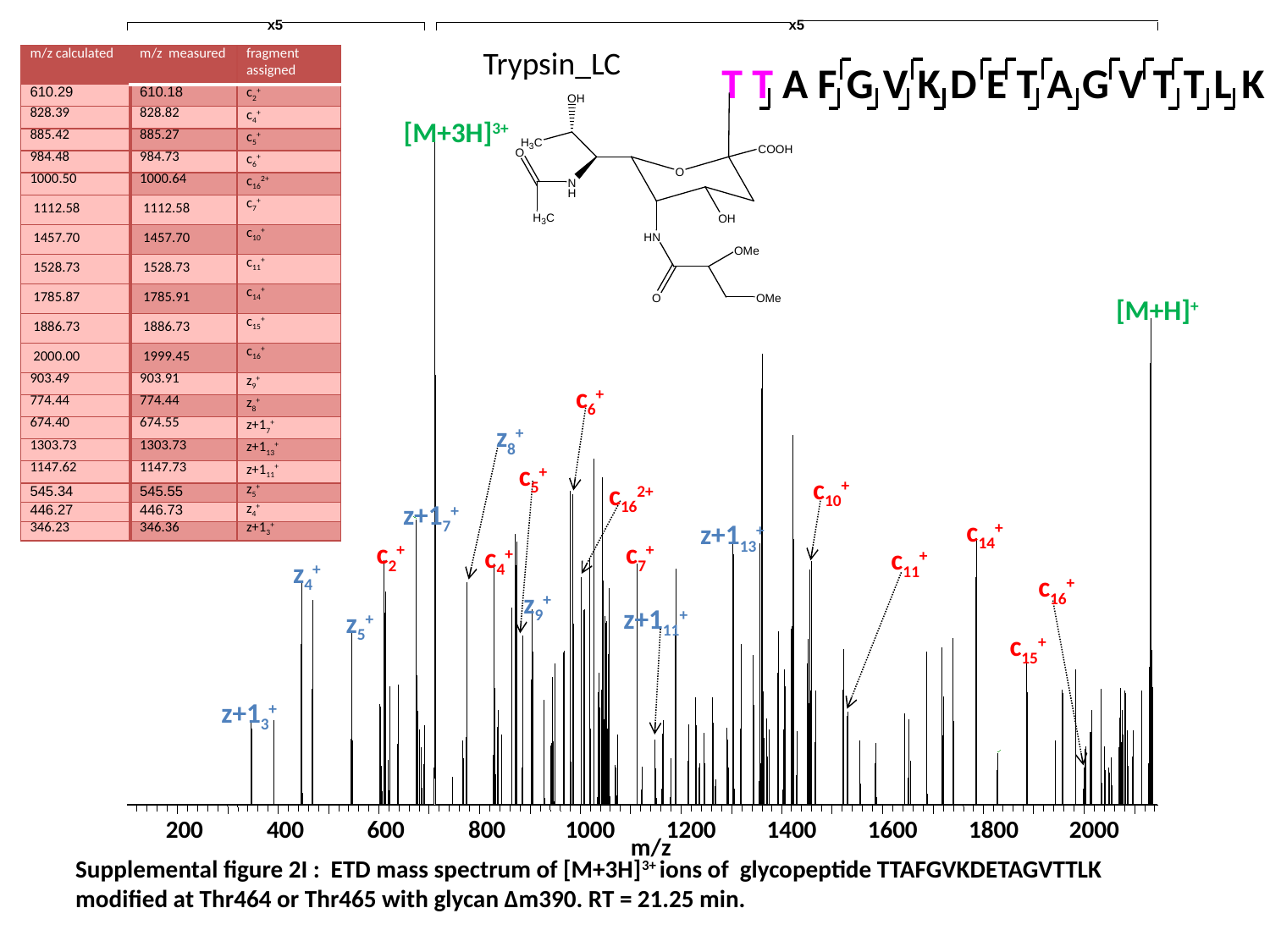

x5
x5
Trypsin_LC
| m/z calculated | m/z measured | fragment assigned |
| --- | --- | --- |
| 610.29 | 610.18 | c2+ |
| 828.39 | 828.82 | c4+ |
| 885.42 | 885.27 | c5+ |
| 984.48 | 984.73 | c6+ |
| 1000.50 | 1000.64 | c162+ |
| 1112.58 | 1112.58 | c7+ |
| 1457.70 | 1457.70 | c10+ |
| 1528.73 | 1528.73 | c11+ |
| 1785.87 | 1785.91 | c14+ |
| 1886.73 | 1886.73 | c15+ |
| 2000.00 | 1999.45 | c16+ |
| 903.49 | 903.91 | z9+ |
| 774.44 | 774.44 | z8+ |
| 674.40 | 674.55 | z+17+ |
| 1303.73 | 1303.73 | z+113+ |
| 1147.62 | 1147.73 | z+111+ |
| 545.34 | 545.55 | z5+ |
| 446.27 | 446.73 | z4+ |
| 346.23 | 346.36 | z+13+ |
T T A F G V K D E T A G V T T L K
[M+3H]3+
[M+H]+
c6+
z8+
c5+
c10+
c162+
z+17+
c14+
z+113+
c2+
c7+
c4+
c11+
z4+
c16+
z9+
z+111+
z5+
c15+
z+13+
200
400
600
800
1000
1200
1400
1600
1800
2000
m/z
Supplemental figure 2I : ETD mass spectrum of [M+3H]3+ ions of glycopeptide TTAFGVKDETAGVTTLK modified at Thr464 or Thr465 with glycan Δm390. RT = 21.25 min.
