# Supplementary material for: Comprehensive mapping of O‐glycosylation in flagellin from Campylobacter jejuni 11168: A multienzyme differential ion mobility mass spectrometry approach
Source: Proteomics. 2015 Jun 15;15(16):2733–45. doi: 10.1002/pmic.201400533 (PMC4975691; doi:10.1002/pmic.201400533)
Supplement: Supplementary file 1 — Figure S1. SDS‐PAGE analysis of purified Campylobacter jejuni flagellin protein. 10% SDS‐PAGE gel, stained with Coomassie blue. Lane 1 – MW markers. Lane 2 – cell suspension from C. jejuni strain 11168 culture, Lane 3 – purified flagellin protein Figure S2. Figure S3. Figure S4. Figure S5. Supplemental Table 1: Non‐glycopeptides identified from tryptic digest of flagellin following ETD MS/MS (with and without FAIMS). (Note that where peptides were identified from both replicates, m/zmeas values are given for replicate#1). Supplemental Table 2: Non‐glycopeptides identified from proteinase K digest of flagellin following ETD MS/MS (without FAIMS). (Note that where peptides were identified from both replicates, m/zmeas values are given for replicate#2). Supplemental Table 3: Non‐glycopeptides identified from proteinase K digest of flagellin following ETD MS/MS (with FAIMS). (Note that where peptides were identified from both replicates, m/zmeas values are given for replicate#2). Comprehensive mapping of O‐glycosylation in flagellin from Campylobacter jejuni 11168: A multi‐enzyme differential ion mobility mass spectrometry approach [file PMIC-15-2733-s001.zip › pmic201400533-sup-0003-figure 3.pptx]

## Slide 1
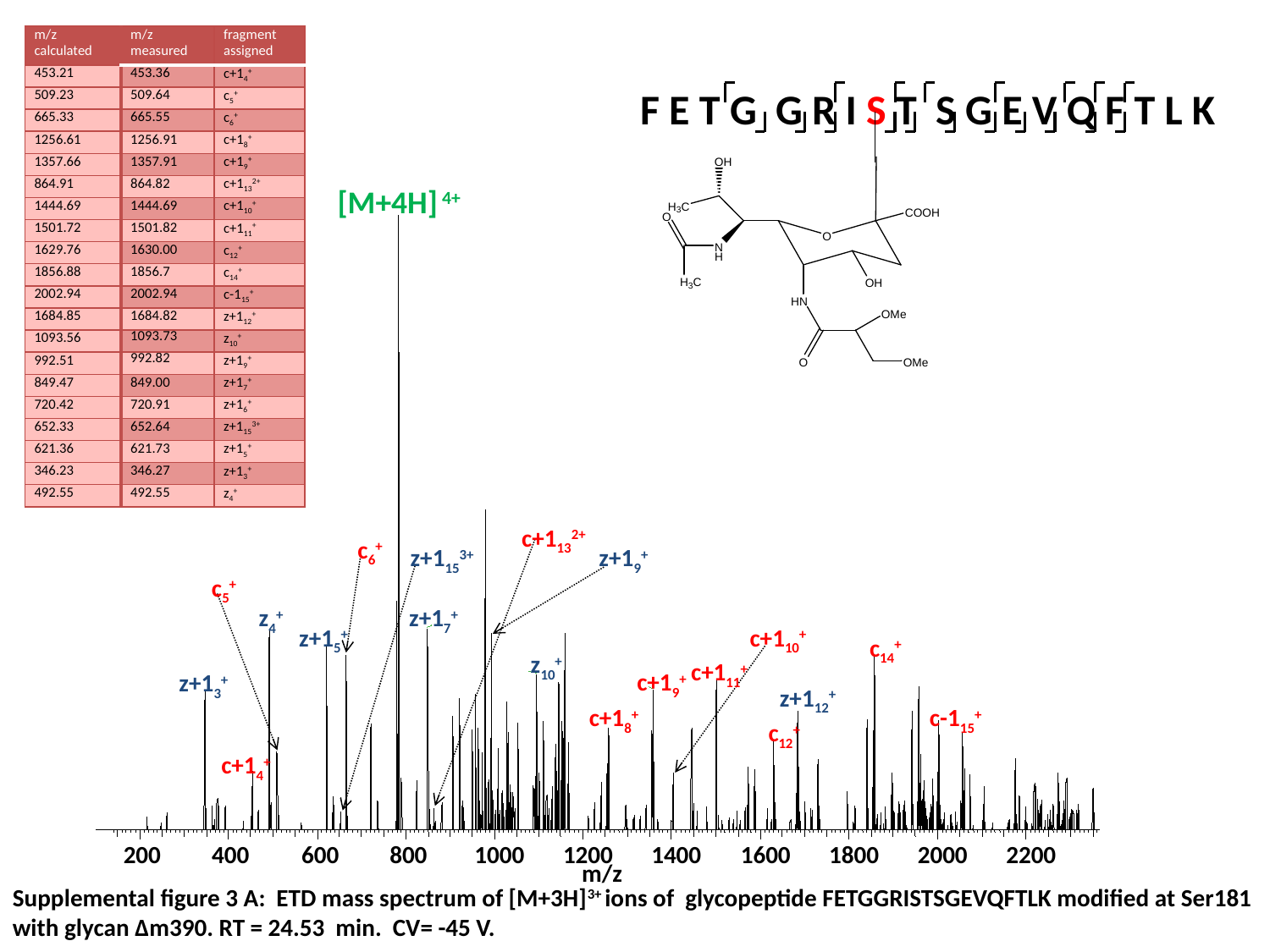

| m/z calculated | m/z measured | fragment assigned |
| --- | --- | --- |
| 453.21 | 453.36 | c+14+ |
| 509.23 | 509.64 | c5+ |
| 665.33 | 665.55 | c6+ |
| 1256.61 | 1256.91 | c+18+ |
| 1357.66 | 1357.91 | c+19+ |
| 864.91 | 864.82 | c+1132+ |
| 1444.69 | 1444.69 | c+110+ |
| 1501.72 | 1501.82 | c+111+ |
| 1629.76 | 1630.00 | c12+ |
| 1856.88 | 1856.7 | c14+ |
| 2002.94 | 2002.94 | c-115+ |
| 1684.85 | 1684.82 | z+112+ |
| 1093.56 | 1093.73 | z10+ |
| 992.51 | 992.82 | z+19+ |
| 849.47 | 849.00 | z+17+ |
| 720.42 | 720.91 | z+16+ |
| 652.33 | 652.64 | z+1153+ |
| 621.36 | 621.73 | z+15+ |
| 346.23 | 346.27 | z+13+ |
| 492.55 | 492.55 | z4+ |
F E T G G R I S T S G E V Q F T L K
[M+4H] 4+
c+1132+
c6+
z+1153+
z+19+
c5+
z4+
z+17+
z+15+
c+110+
c14+
z10+
c+111+
c+19+
z+13+
z+112+
c+18+
c-115+
c12+
c+14+
200
400
600
800
1000
1200
1400
1600
1800
2000
2200
m/z
Supplemental figure 3 A: ETD mass spectrum of [M+3H]3+ ions of glycopeptide FETGGRISTSGEVQFTLK modified at Ser181 with glycan Δm390. RT = 24.53 min. CV= -45 V.

## Slide 2
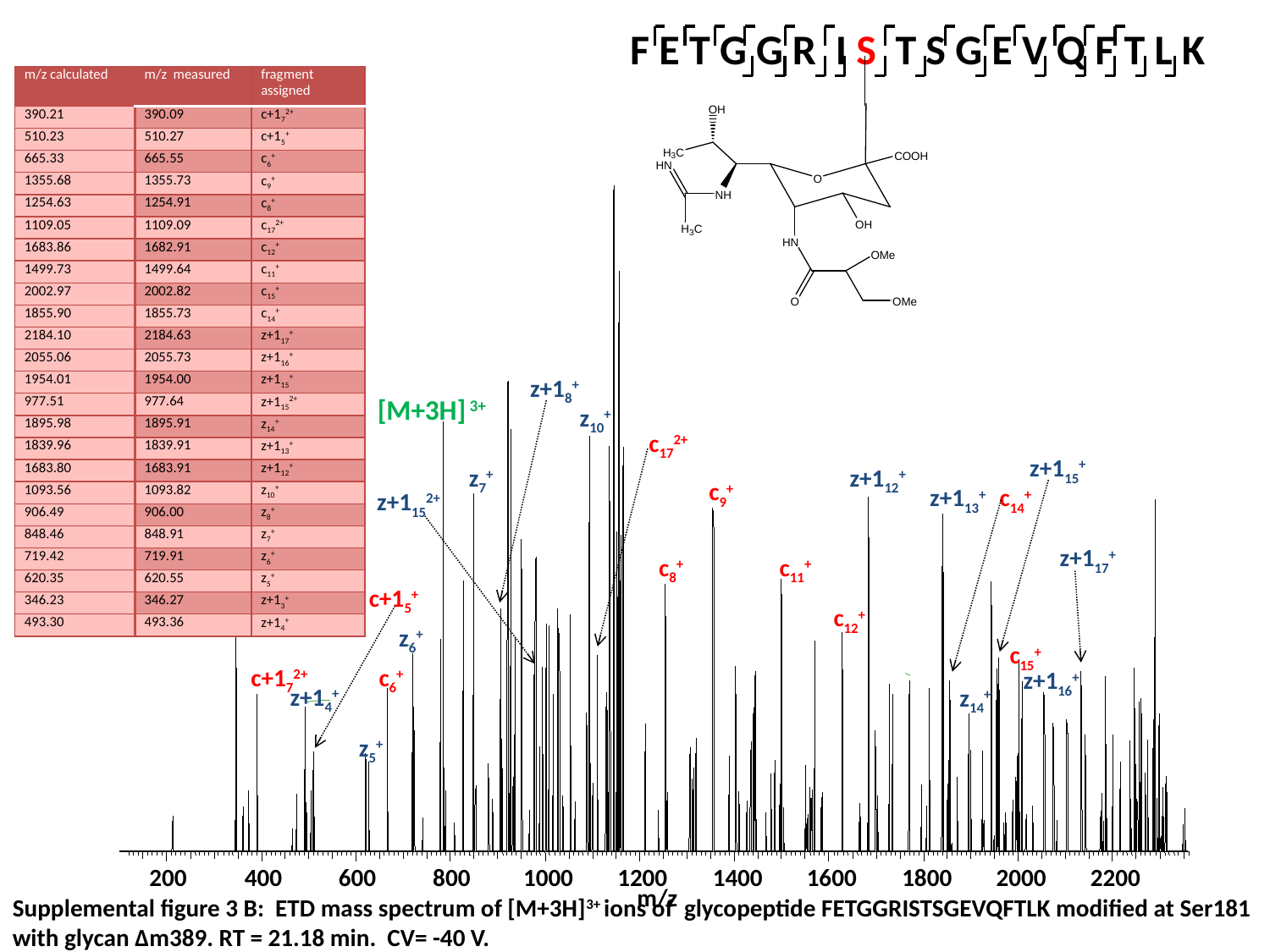

F E T G G R I S T S G E V Q F T L K
| m/z calculated | m/z measured | fragment assigned |
| --- | --- | --- |
| 390.21 | 390.09 | c+172+ |
| 510.23 | 510.27 | c+15+ |
| 665.33 | 665.55 | c6+ |
| 1355.68 | 1355.73 | c9+ |
| 1254.63 | 1254.91 | c8+ |
| 1109.05 | 1109.09 | c172+ |
| 1683.86 | 1682.91 | c12+ |
| 1499.73 | 1499.64 | c11+ |
| 2002.97 | 2002.82 | c15+ |
| 1855.90 | 1855.73 | c14+ |
| 2184.10 | 2184.63 | z+117+ |
| 2055.06 | 2055.73 | z+116+ |
| 1954.01 | 1954.00 | z+115+ |
| 977.51 | 977.64 | z+1152+ |
| 1895.98 | 1895.91 | z14+ |
| 1839.96 | 1839.91 | z+113+ |
| 1683.80 | 1683.91 | z+112+ |
| 1093.56 | 1093.82 | z10+ |
| 906.49 | 906.00 | z8+ |
| 848.46 | 848.91 | z7+ |
| 719.42 | 719.91 | z6+ |
| 620.35 | 620.55 | z5+ |
| 346.23 | 346.27 | z+13+ |
| 493.30 | 493.36 | z+14+ |
z+18+
[M+3H] 3+
z10+
c172+
z+115+
z7+
z+112+
c9+
z+113+
c14+
z+1152+
z+117+
c8+
c11+
z+13+
c+15+
c12+
z6+
c15+
c+172+
c6+
z+116+
z+14+
z14+
z5+
200
400
600
800
1000
1200
1400
1600
1800
2000
2200
m/z
Supplemental figure 3 B: ETD mass spectrum of [M+3H]3+ ions of glycopeptide FETGGRISTSGEVQFTLK modified at Ser181 with glycan Δm389. RT = 21.18 min. CV= -40 V.

## Slide 3
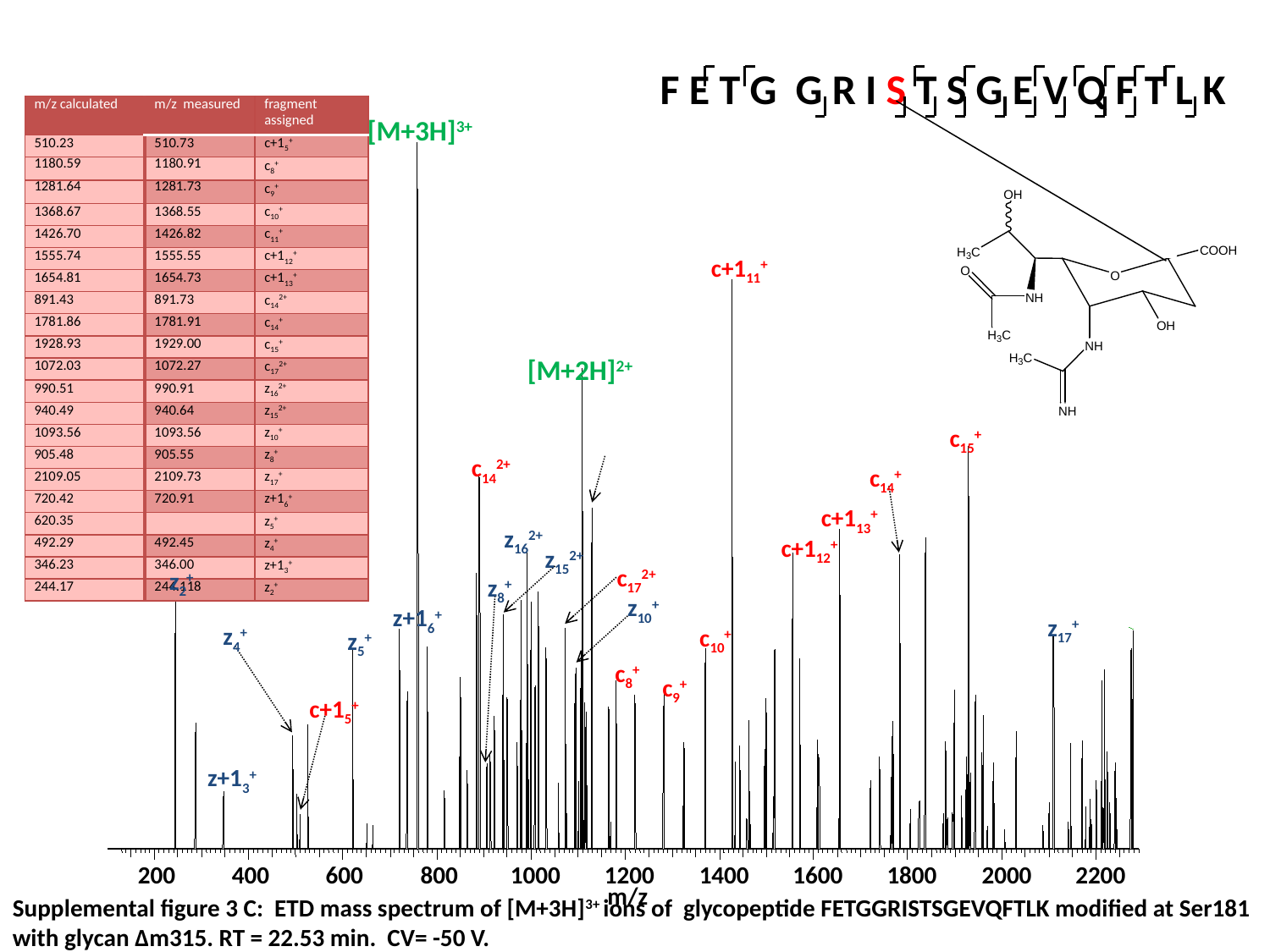

F E T G G R I S T S G E V Q F T L K
| m/z calculated | m/z measured | fragment assigned |
| --- | --- | --- |
| 510.23 | 510.73 | c+15+ |
| 1180.59 | 1180.91 | c8+ |
| 1281.64 | 1281.73 | c9+ |
| 1368.67 | 1368.55 | c10+ |
| 1426.70 | 1426.82 | c11+ |
| 1555.74 | 1555.55 | c+112+ |
| 1654.81 | 1654.73 | c+113+ |
| 891.43 | 891.73 | c142+ |
| 1781.86 | 1781.91 | c14+ |
| 1928.93 | 1929.00 | c15+ |
| 1072.03 | 1072.27 | c172+ |
| 990.51 | 990.91 | z162+ |
| 940.49 | 940.64 | z152+ |
| 1093.56 | 1093.56 | z10+ |
| 905.48 | 905.55 | z8+ |
| 2109.05 | 2109.73 | z17+ |
| 720.42 | 720.91 | z+16+ |
| 620.35 | | z5+ |
| 492.29 | 492.45 | z4+ |
| 346.23 | 346.00 | z+13+ |
| 244.17 | 244.118 | z2+ |
[M+3H]3+
c+111+
[M+2H]2+
c15+
c142+
c14+
c+113+
z162+
c+112+
z152+
c172+
z2+
z8+
z10+
z+16+
z17+
z4+
c10+
z5+
c8+
c9+
c+15+
z+13+
200
400
600
800
1000
1200
1400
1600
1800
2000
2200
m/z
Supplemental figure 3 C: ETD mass spectrum of [M+3H]3+ ions of glycopeptide FETGGRISTSGEVQFTLK modified at Ser181 with glycan Δm315. RT = 22.53 min. CV= -50 V.

## Slide 4
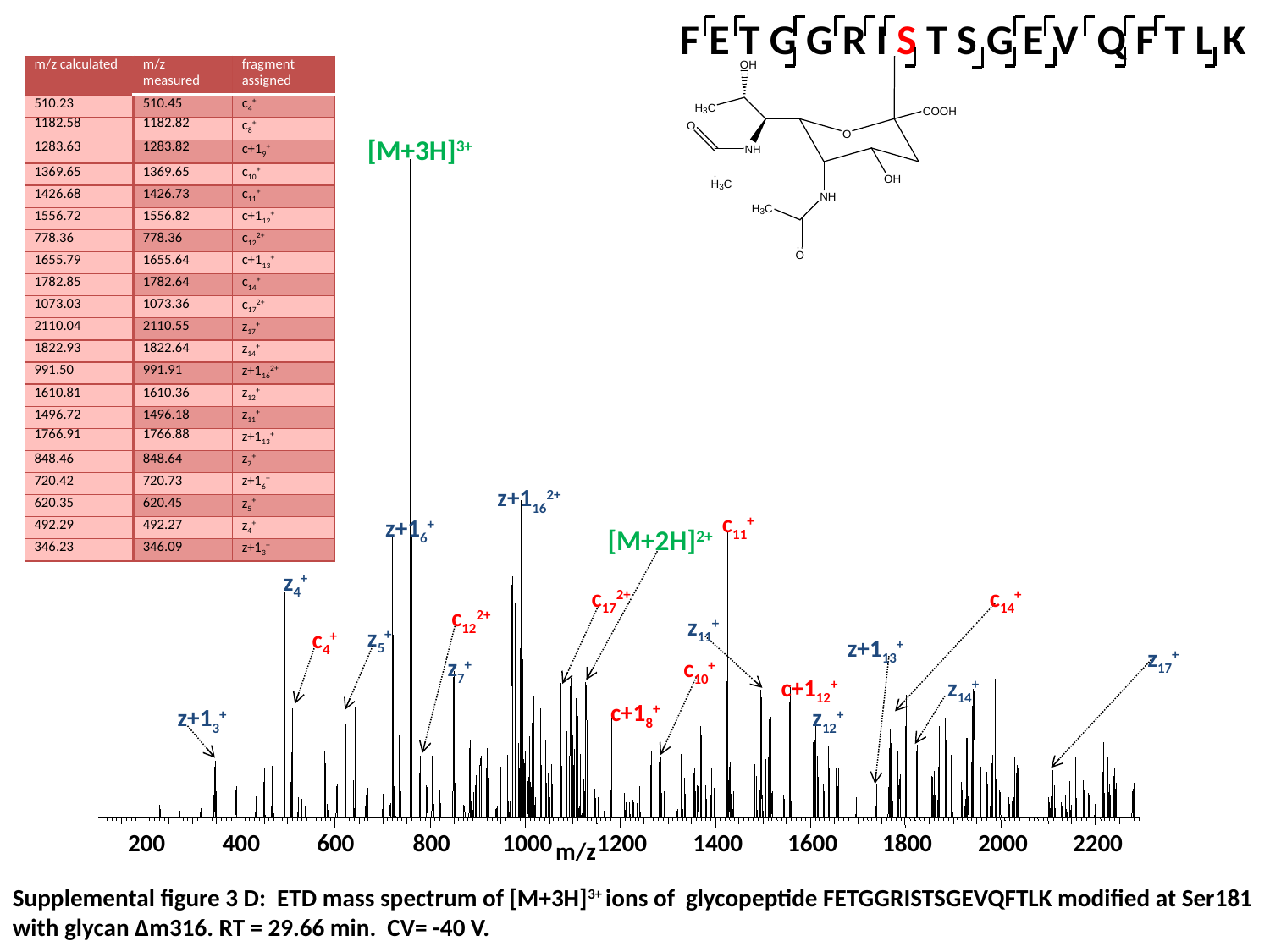

F E T G G R I S T S G E V Q F T L K
| m/z calculated | m/z measured | fragment assigned |
| --- | --- | --- |
| 510.23 | 510.45 | c4+ |
| 1182.58 | 1182.82 | c8+ |
| 1283.63 | 1283.82 | c+19+ |
| 1369.65 | 1369.65 | c10+ |
| 1426.68 | 1426.73 | c11+ |
| 1556.72 | 1556.82 | c+112+ |
| 778.36 | 778.36 | c122+ |
| 1655.79 | 1655.64 | c+113+ |
| 1782.85 | 1782.64 | c14+ |
| 1073.03 | 1073.36 | c172+ |
| 2110.04 | 2110.55 | z17+ |
| 1822.93 | 1822.64 | z14+ |
| 991.50 | 991.91 | z+1162+ |
| 1610.81 | 1610.36 | z12+ |
| 1496.72 | 1496.18 | z11+ |
| 1766.91 | 1766.88 | z+113+ |
| 848.46 | 848.64 | z7+ |
| 720.42 | 720.73 | z+16+ |
| 620.35 | 620.45 | z5+ |
| 492.29 | 492.27 | z4+ |
| 346.23 | 346.09 | z+13+ |
[M+3H]3+
z+1162+
c11+
z+16+
[M+2H]2+
z4+
c172+
c14+
c122+
z11+
z5+
c4+
z+113+
z17+
z7+
c10+
c+112+
z14+
c+18+
z+13+
z12+
200
400
600
800
1000
1200
1400
1600
1800
2000
2200
m/z
Supplemental figure 3 D: ETD mass spectrum of [M+3H]3+ ions of glycopeptide FETGGRISTSGEVQFTLK modified at Ser181 with glycan Δm316. RT = 29.66 min. CV= -40 V.

## Slide 5
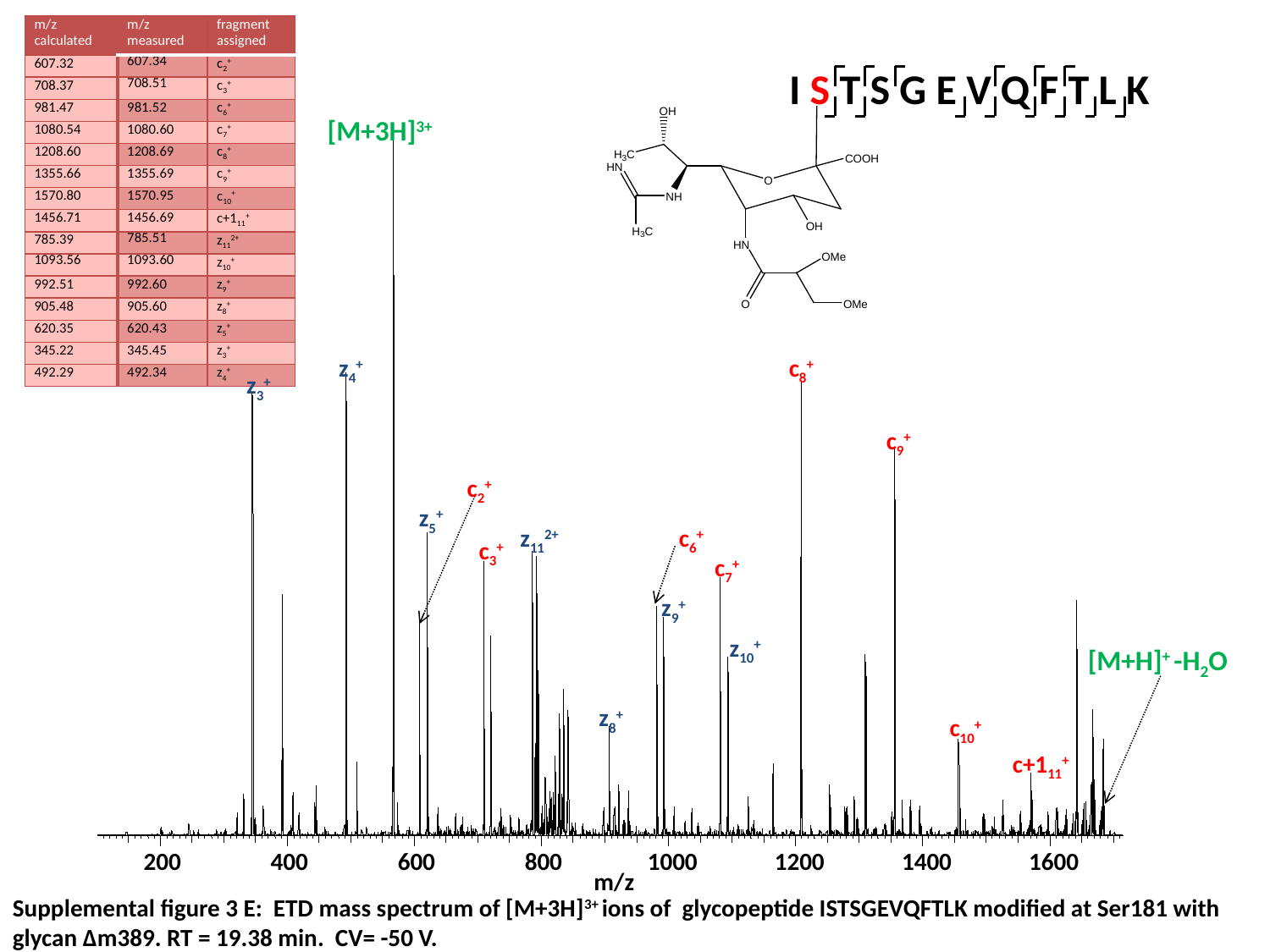

| m/z calculated | m/z measured | fragment assigned |
| --- | --- | --- |
| 607.32 | 607.34 | c2+ |
| 708.37 | 708.51 | c3+ |
| 981.47 | 981.52 | c6+ |
| 1080.54 | 1080.60 | c7+ |
| 1208.60 | 1208.69 | c8+ |
| 1355.66 | 1355.69 | c9+ |
| 1570.80 | 1570.95 | c10+ |
| 1456.71 | 1456.69 | c+111+ |
| 785.39 | 785.51 | z112+ |
| 1093.56 | 1093.60 | z10+ |
| 992.51 | 992.60 | z9+ |
| 905.48 | 905.60 | z8+ |
| 620.35 | 620.43 | z5+ |
| 345.22 | 345.45 | z3+ |
| 492.29 | 492.34 | z4+ |
I S T S G E V Q F T L K
[M+3H]3+
z4+
c8+
z3+
c9+
c2+
z5+
z112+
c6+
c3+
c7+
z9+
z10+
[M+H]+ -H2O
z8+
c10+
c+111+
200
400
600
800
1000
1200
1400
1600
m/z
Supplemental figure 3 E: ETD mass spectrum of [M+3H]3+ ions of glycopeptide ISTSGEVQFTLK modified at Ser181 with glycan Δm389. RT = 19.38 min. CV= -50 V.

## Slide 6
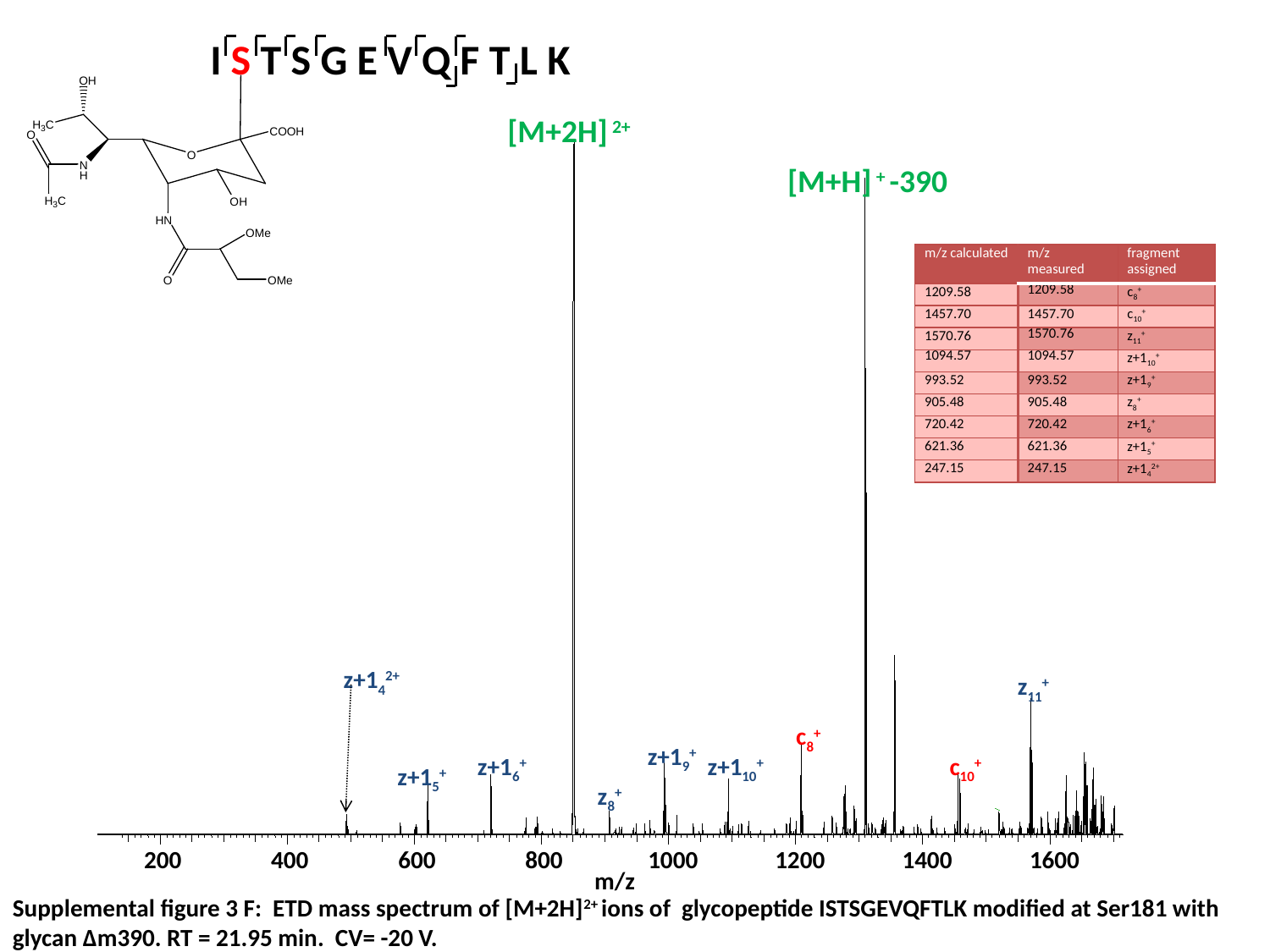

I S T S G E V Q F T L K
[M+2H] 2+
[M+H] + -390
| m/z calculated | m/z measured | fragment assigned |
| --- | --- | --- |
| 1209.58 | 1209.58 | c8+ |
| 1457.70 | 1457.70 | c10+ |
| 1570.76 | 1570.76 | z11+ |
| 1094.57 | 1094.57 | z+110+ |
| 993.52 | 993.52 | z+19+ |
| 905.48 | 905.48 | z8+ |
| 720.42 | 720.42 | z+16+ |
| 621.36 | 621.36 | z+15+ |
| 247.15 | 247.15 | z+142+ |
z+142+
z11+
c8+
z+19+
z+16+
z+110+
c10+
z+15+
z8+
200
400
600
800
1000
1200
1400
1600
m/z
Supplemental figure 3 F: ETD mass spectrum of [M+2H]2+ ions of glycopeptide ISTSGEVQFTLK modified at Ser181 with glycan Δm390. RT = 21.95 min. CV= -20 V.

## Slide 7
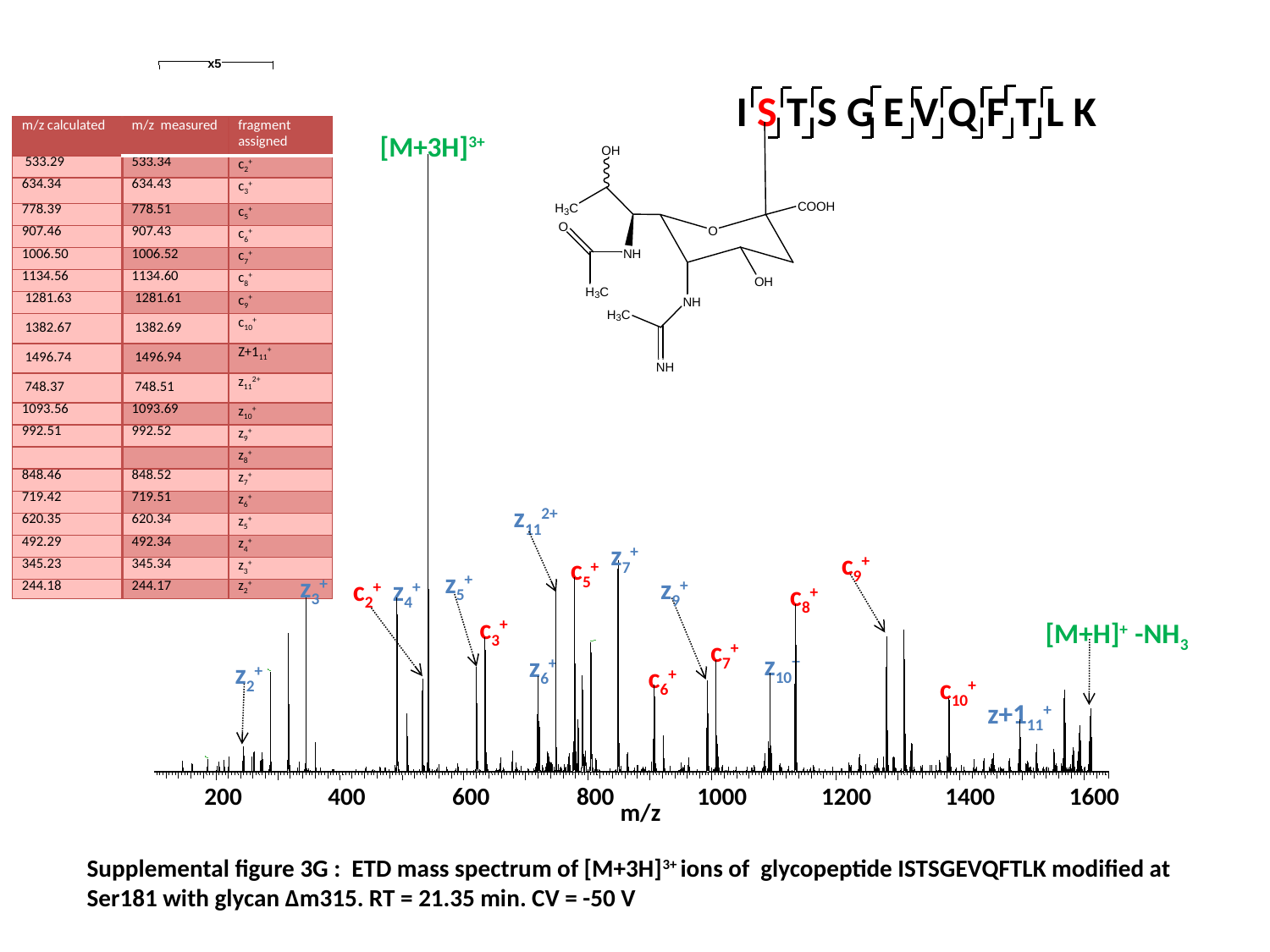

x5
I S T S G E V Q F T L K
| m/z calculated | m/z measured | fragment assigned |
| --- | --- | --- |
| 533.29 | 533.34 | c2+ |
| 634.34 | 634.43 | c3+ |
| 778.39 | 778.51 | c5+ |
| 907.46 | 907.43 | c6+ |
| 1006.50 | 1006.52 | c7+ |
| 1134.56 | 1134.60 | c8+ |
| 1281.63 | 1281.61 | c9+ |
| 1382.67 | 1382.69 | c10+ |
| 1496.74 | 1496.94 | Z+111+ |
| 748.37 | 748.51 | z112+ |
| 1093.56 | 1093.69 | z10+ |
| 992.51 | 992.52 | z9+ |
| | | z8+ |
| 848.46 | 848.52 | z7+ |
| 719.42 | 719.51 | z6+ |
| 620.35 | 620.34 | z5+ |
| 492.29 | 492.34 | z4+ |
| 345.23 | 345.34 | z3+ |
| 244.18 | 244.17 | z2+ |
[M+3H]3+
z112+
z7+
c9+
c5+
z5+
z3+
z9+
c2+
z4+
c8+
c3+
[M+H]+ -NH3
c7+
z10+
z6+
z2+
c6+
c10+
z+111+
200
400
600
800
1000
1200
1400
1600
m/z
Supplemental figure 3G : ETD mass spectrum of [M+3H]3+ ions of glycopeptide ISTSGEVQFTLK modified at Ser181 with glycan Δm315. RT = 21.35 min. CV = -50 V

## Slide 8
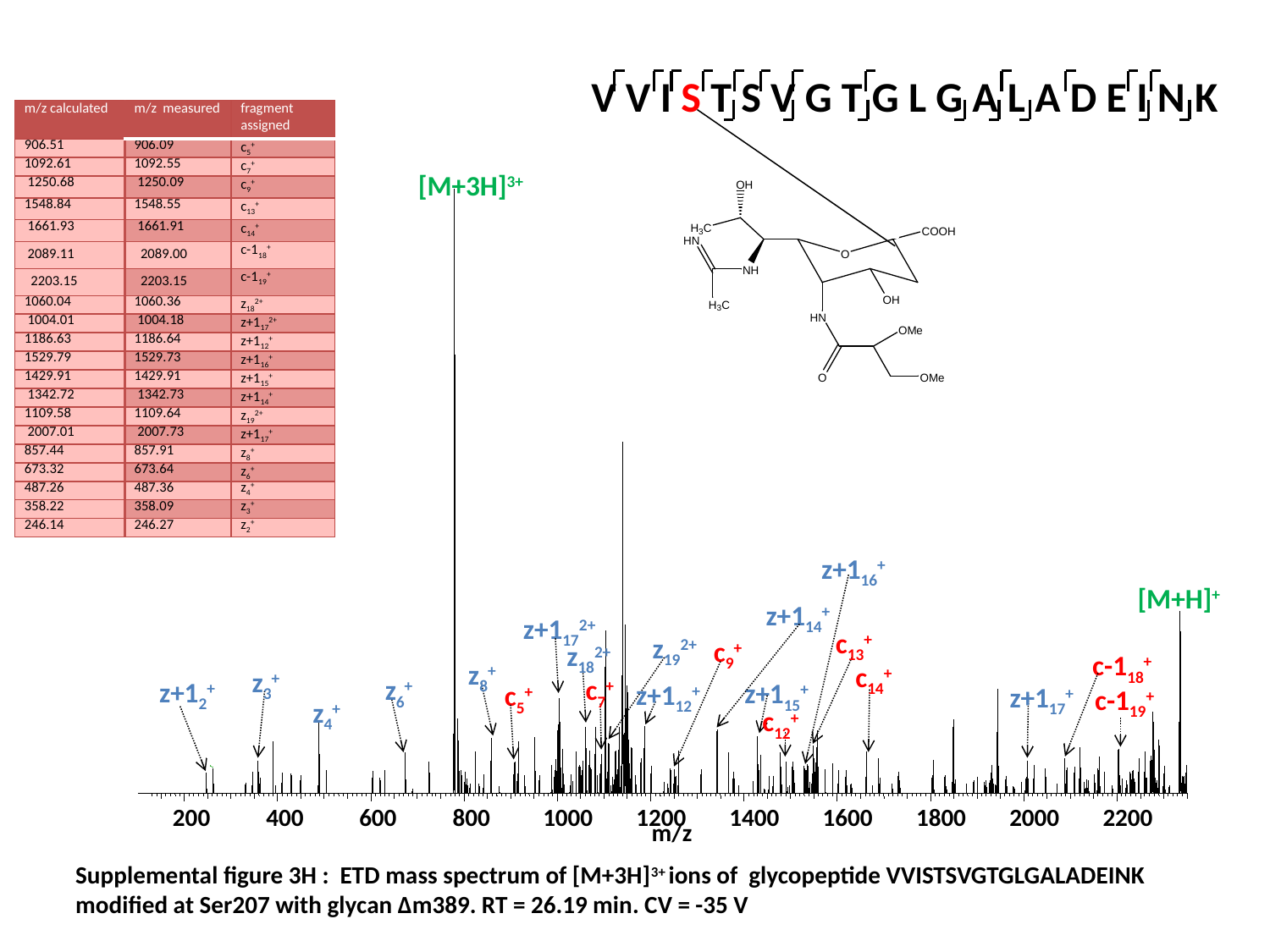

V V I S T S V G T G L G A L A D E I N K
| m/z calculated | m/z measured | fragment assigned |
| --- | --- | --- |
| 906.51 | 906.09 | c5+ |
| 1092.61 | 1092.55 | c7+ |
| 1250.68 | 1250.09 | c9+ |
| 1548.84 | 1548.55 | c13+ |
| 1661.93 | 1661.91 | c14+ |
| 2089.11 | 2089.00 | c-118+ |
| 2203.15 | 2203.15 | c-119+ |
| 1060.04 | 1060.36 | z182+ |
| 1004.01 | 1004.18 | z+1172+ |
| 1186.63 | 1186.64 | z+112+ |
| 1529.79 | 1529.73 | z+116+ |
| 1429.91 | 1429.91 | z+115+ |
| 1342.72 | 1342.73 | z+114+ |
| 1109.58 | 1109.64 | z192+ |
| 2007.01 | 2007.73 | z+117+ |
| 857.44 | 857.91 | z8+ |
| 673.32 | 673.64 | z6+ |
| 487.26 | 487.36 | z4+ |
| 358.22 | 358.09 | z3+ |
| 246.14 | 246.27 | z2+ |
[M+3H]3+
z+116+
[M+H]+
z+114+
z+1172+
c13+
z192+
c9+
z182+
c-118+
z8+
c14+
z3+
z6+
c7+
z+12+
z+115+
z+112+
c5+
z+117+
c-119+
z4+
c12+
200
400
600
800
1000
1200
1400
1600
1800
2000
2200
m/z
Supplemental figure 3H : ETD mass spectrum of [M+3H]3+ ions of glycopeptide VVISTSVGTGLGALADEINK modified at Ser207 with glycan Δm389. RT = 26.19 min. CV = -35 V

## Slide 9
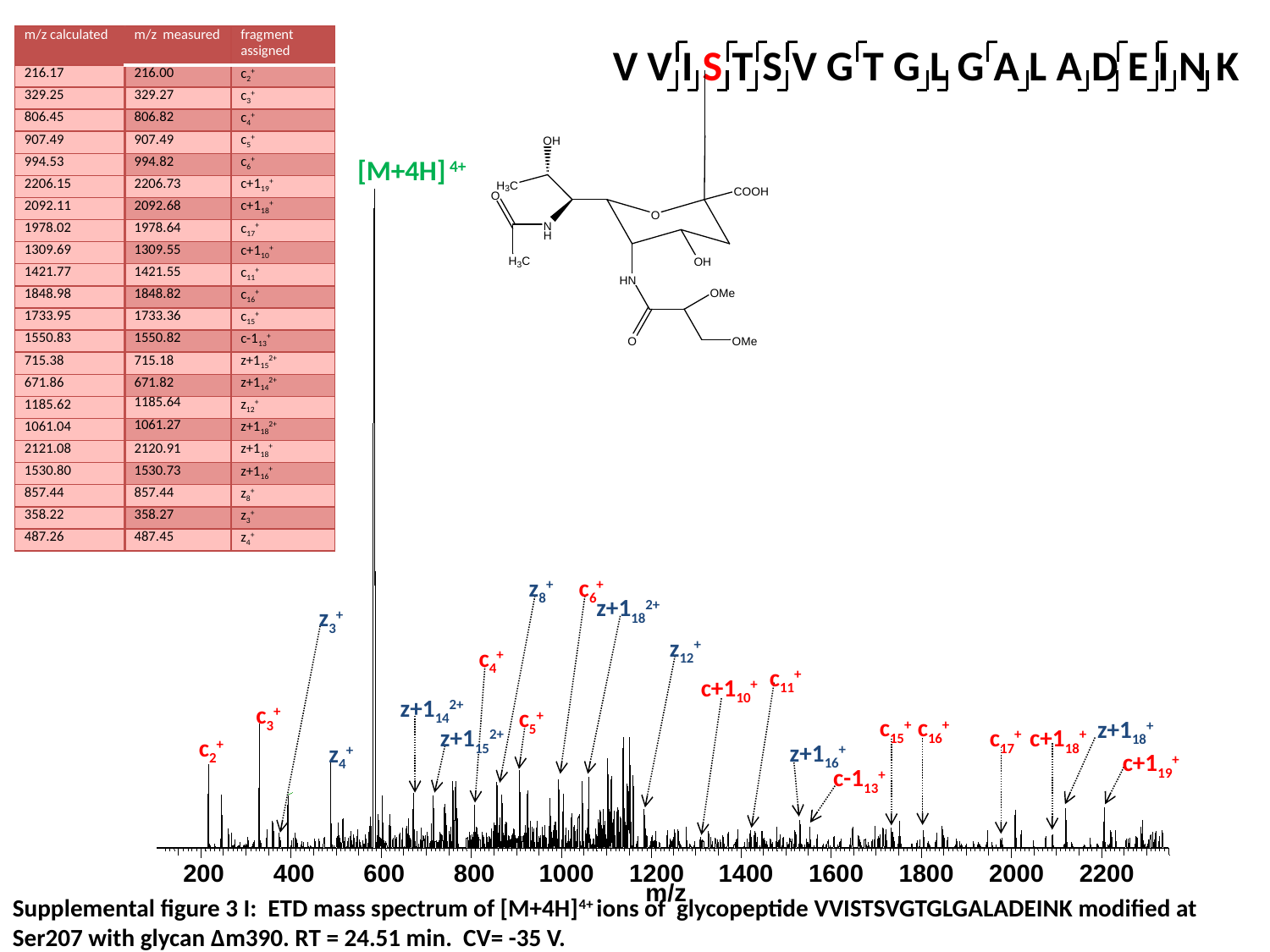

| m/z calculated | m/z measured | fragment assigned |
| --- | --- | --- |
| 216.17 | 216.00 | c2+ |
| 329.25 | 329.27 | c3+ |
| 806.45 | 806.82 | c4+ |
| 907.49 | 907.49 | c5+ |
| 994.53 | 994.82 | c6+ |
| 2206.15 | 2206.73 | c+119+ |
| 2092.11 | 2092.68 | c+118+ |
| 1978.02 | 1978.64 | c17+ |
| 1309.69 | 1309.55 | c+110+ |
| 1421.77 | 1421.55 | c11+ |
| 1848.98 | 1848.82 | c16+ |
| 1733.95 | 1733.36 | c15+ |
| 1550.83 | 1550.82 | c-113+ |
| 715.38 | 715.18 | z+1152+ |
| 671.86 | 671.82 | z+1142+ |
| 1185.62 | 1185.64 | z12+ |
| 1061.04 | 1061.27 | z+1182+ |
| 2121.08 | 2120.91 | z+118+ |
| 1530.80 | 1530.73 | z+116+ |
| 857.44 | 857.44 | z8+ |
| 358.22 | 358.27 | z3+ |
| 487.26 | 487.45 | z4+ |
V V I S T S V G T G L G A L A D E I N K
[M+4H] 4+
z8+
c6+
z+1182+
z3+
z12+
c4+
c11+
c+110+
z+1142+
c3+
c5+
c15+
c16+
z+118+
z+1152+
c17+
c+118+
c2+
z+116+
z4+
c+119+
c-113+
200
400
600
800
1000
1200
1400
1600
1800
2000
2200
m/z
Supplemental figure 3 I: ETD mass spectrum of [M+4H]4+ ions of glycopeptide VVISTSVGTGLGALADEINK modified at Ser207 with glycan Δm390. RT = 24.51 min. CV= -35 V.

## Slide 10
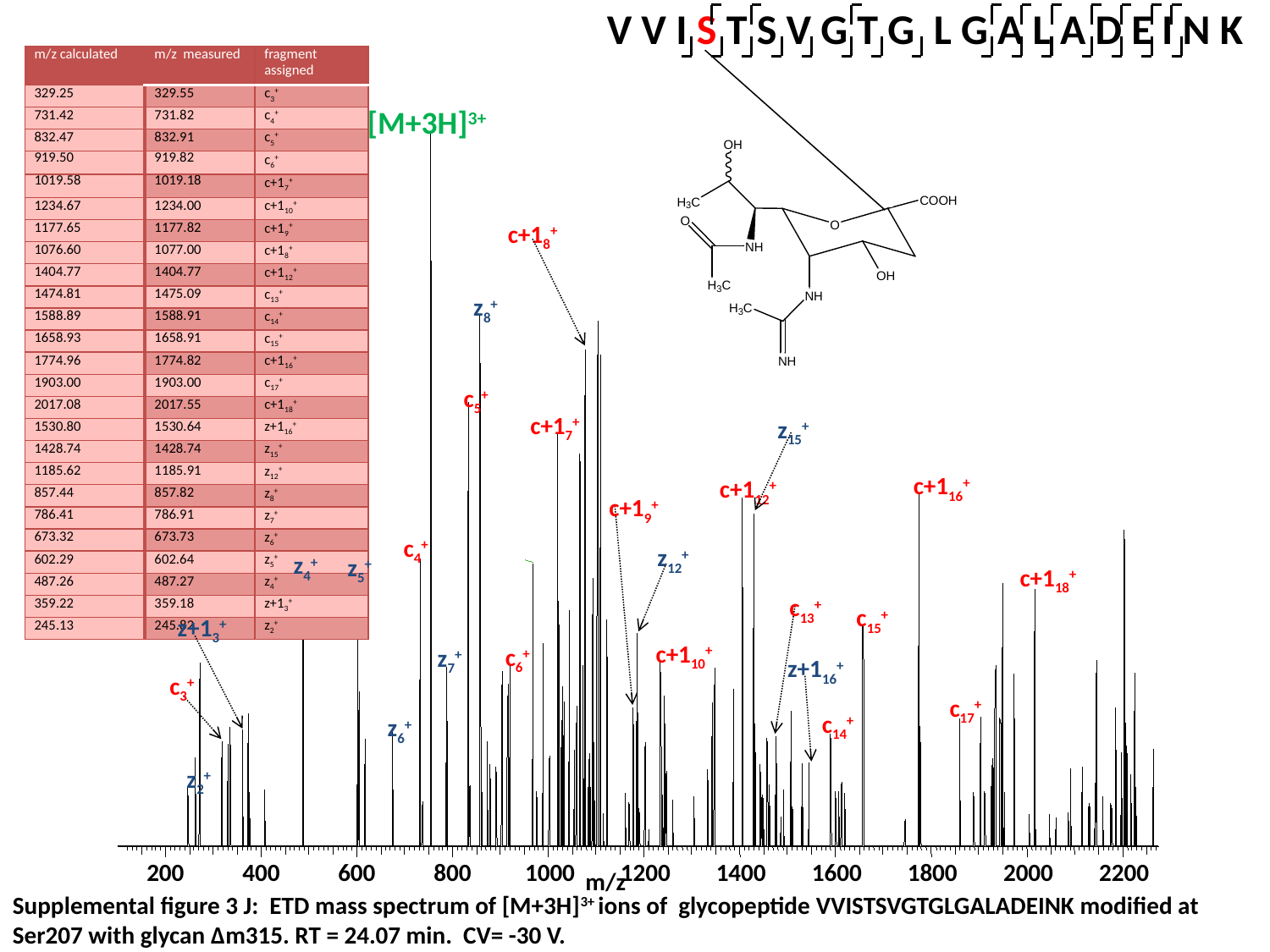

V V I S T S V G T G L G A L A D E I N K
| m/z calculated | m/z measured | fragment assigned |
| --- | --- | --- |
| 329.25 | 329.55 | c3+ |
| 731.42 | 731.82 | c4+ |
| 832.47 | 832.91 | c5+ |
| 919.50 | 919.82 | c6+ |
| 1019.58 | 1019.18 | c+17+ |
| 1234.67 | 1234.00 | c+110+ |
| 1177.65 | 1177.82 | c+19+ |
| 1076.60 | 1077.00 | c+18+ |
| 1404.77 | 1404.77 | c+112+ |
| 1474.81 | 1475.09 | c13+ |
| 1588.89 | 1588.91 | c14+ |
| 1658.93 | 1658.91 | c15+ |
| 1774.96 | 1774.82 | c+116+ |
| 1903.00 | 1903.00 | c17+ |
| 2017.08 | 2017.55 | c+118+ |
| 1530.80 | 1530.64 | z+116+ |
| 1428.74 | 1428.74 | z15+ |
| 1185.62 | 1185.91 | z12+ |
| 857.44 | 857.82 | z8+ |
| 786.41 | 786.91 | z7+ |
| 673.32 | 673.73 | z6+ |
| 602.29 | 602.64 | z5+ |
| 487.26 | 487.27 | z4+ |
| 359.22 | 359.18 | z+13+ |
| 245.13 | 245.82 | z2+ |
[M+3H]3+
c+18+
z8+
c5+
c+17+
z15+
c+116+
c+112+
c+19+
c4+
z12+
z4+
z5+
c+118+
c13+
c15+
z+13+
c+110+
c6+
z7+
z+116+
c3+
c17+
c14+
z6+
z2+
200
400
600
800
1000
1200
1400
1600
1800
2000
2200
m/z
Supplemental figure 3 J: ETD mass spectrum of [M+3H]3+ ions of glycopeptide VVISTSVGTGLGALADEINK modified at Ser207 with glycan Δm315. RT = 24.07 min. CV= -30 V.

## Slide 11
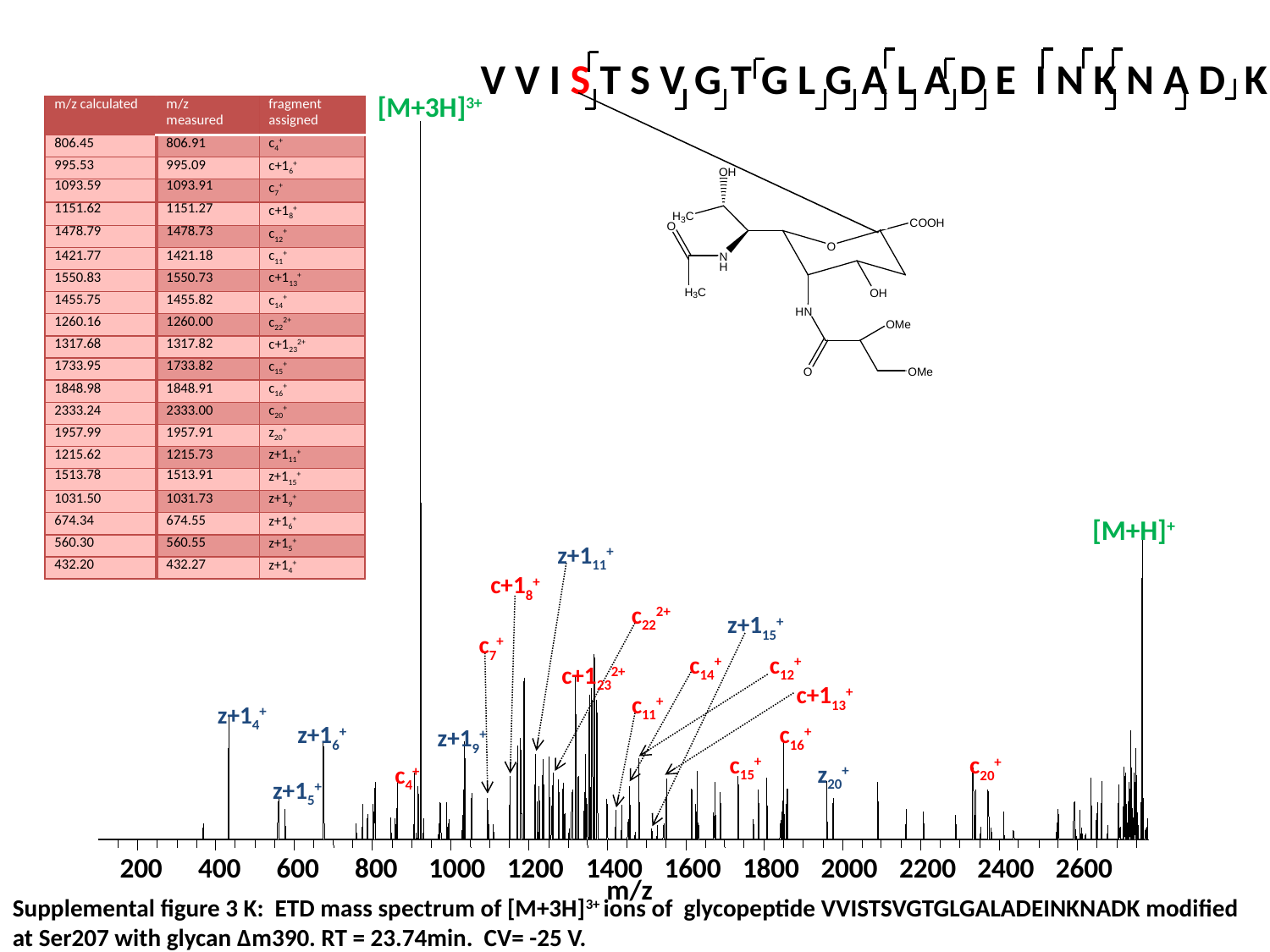

V V I S T S V G T G L G A L A D E I N K N A D K
[M+3H]3+
| m/z calculated | m/z measured | fragment assigned |
| --- | --- | --- |
| 806.45 | 806.91 | c4+ |
| 995.53 | 995.09 | c+16+ |
| 1093.59 | 1093.91 | c7+ |
| 1151.62 | 1151.27 | c+18+ |
| 1478.79 | 1478.73 | c12+ |
| 1421.77 | 1421.18 | c11+ |
| 1550.83 | 1550.73 | c+113+ |
| 1455.75 | 1455.82 | c14+ |
| 1260.16 | 1260.00 | c222+ |
| 1317.68 | 1317.82 | c+1232+ |
| 1733.95 | 1733.82 | c15+ |
| 1848.98 | 1848.91 | c16+ |
| 2333.24 | 2333.00 | c20+ |
| 1957.99 | 1957.91 | z20+ |
| 1215.62 | 1215.73 | z+111+ |
| 1513.78 | 1513.91 | z+115+ |
| 1031.50 | 1031.73 | z+19+ |
| 674.34 | 674.55 | z+16+ |
| 560.30 | 560.55 | z+15+ |
| 432.20 | 432.27 | z+14+ |
[M+H]+
z+111+
c+18+
c222+
z+115+
c7+
c14+
c12+
c+1232+
c+113+
c11+
z+14+
z+16+
c16+
z+19+
c15+
c20+
z20+
c4+
z+15+
200
400
600
800
1000
1200
1400
1600
1800
2000
2200
2400
2600
m/z
Supplemental figure 3 K: ETD mass spectrum of [M+3H]3+ ions of glycopeptide VVISTSVGTGLGALADEINKNADK modified at Ser207 with glycan Δm390. RT = 23.74min. CV= -25 V.

## Slide 12
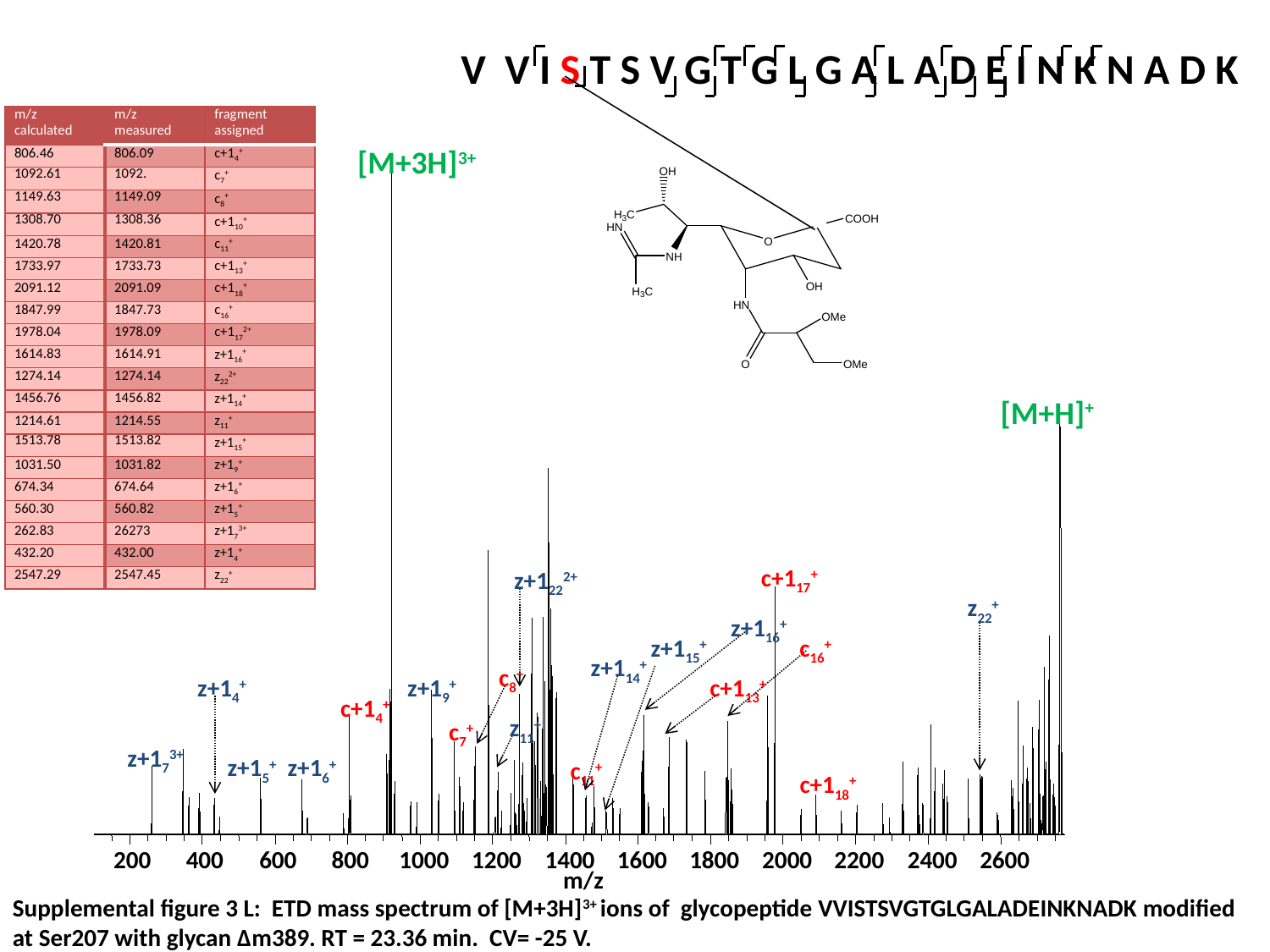

V V I S T S V G T G L G A L A D E I N K N A D K
| m/z calculated | m/z measured | fragment assigned |
| --- | --- | --- |
| 806.46 | 806.09 | c+14+ |
| 1092.61 | 1092. | c7+ |
| 1149.63 | 1149.09 | c8+ |
| 1308.70 | 1308.36 | c+110+ |
| 1420.78 | 1420.81 | c11+ |
| 1733.97 | 1733.73 | c+113+ |
| 2091.12 | 2091.09 | c+118+ |
| 1847.99 | 1847.73 | c16+ |
| 1978.04 | 1978.09 | c+1172+ |
| 1614.83 | 1614.91 | z+116+ |
| 1274.14 | 1274.14 | z222+ |
| 1456.76 | 1456.82 | z+114+ |
| 1214.61 | 1214.55 | z11+ |
| 1513.78 | 1513.82 | z+115+ |
| 1031.50 | 1031.82 | z+19+ |
| 674.34 | 674.64 | z+16+ |
| 560.30 | 560.82 | z+15+ |
| 262.83 | 26273 | z+173+ |
| 432.20 | 432.00 | z+14+ |
| 2547.29 | 2547.45 | z22+ |
[M+3H]3+
[M+H]+
c+117+
z+1222+
z22+
z+116+
z+115+
c16+
z+114+
c8+
z+14+
z+19+
c+113+
c+14+
z11+
c7+
z+173+
z+15+
z+16+
c11+
c+118+
200
400
600
800
1000
1200
1400
1600
1800
2000
2200
2400
2600
m/z
Supplemental figure 3 L: ETD mass spectrum of [M+3H]3+ ions of glycopeptide VVISTSVGTGLGALADEINKNADK modified at Ser207 with glycan Δm389. RT = 23.36 min. CV= -25 V.

## Slide 13
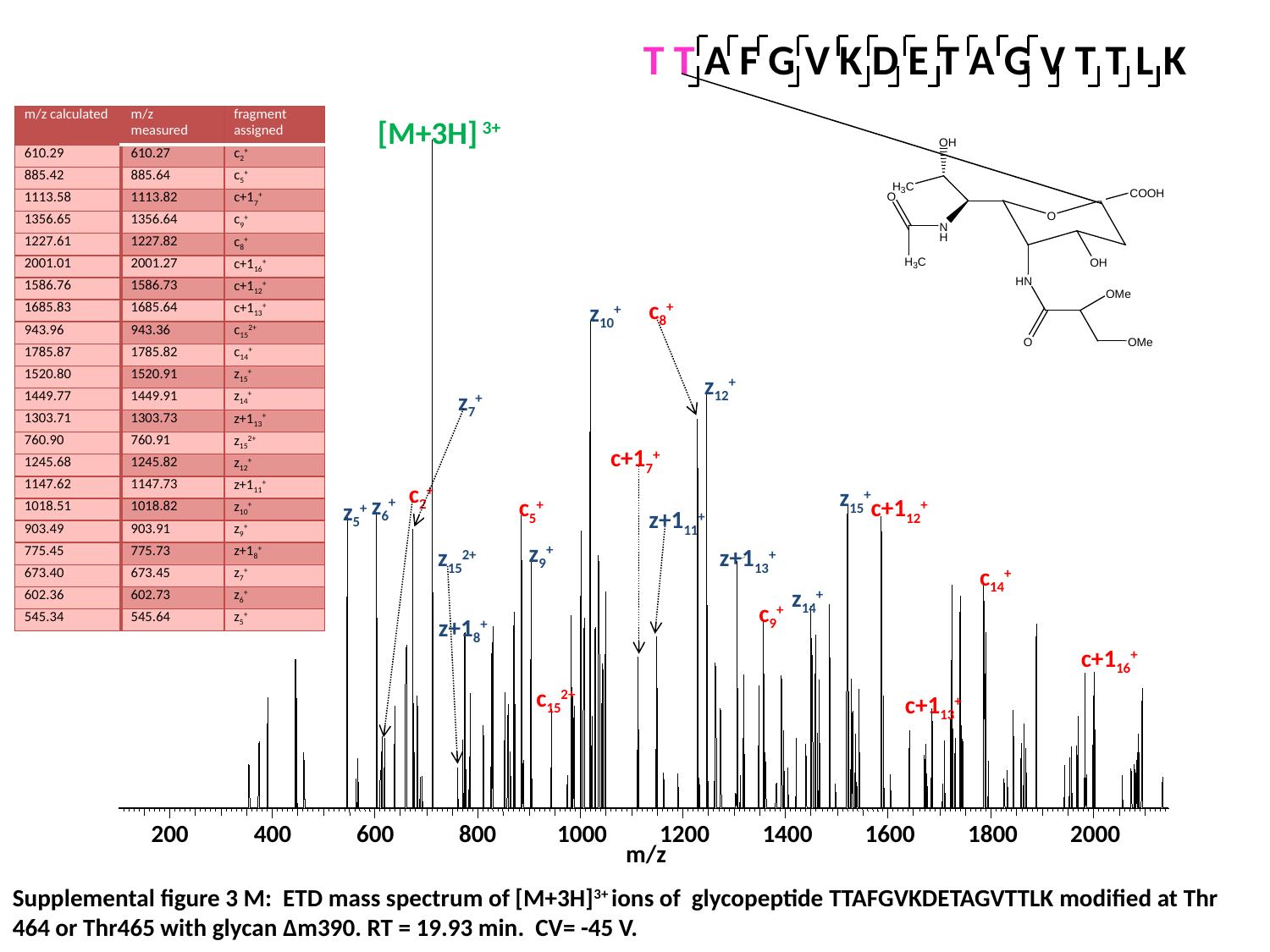

T T A F G V K D E T A G V T T L K
| m/z calculated | m/z measured | fragment assigned |
| --- | --- | --- |
| 610.29 | 610.27 | c2+ |
| 885.42 | 885.64 | c5+ |
| 1113.58 | 1113.82 | c+17+ |
| 1356.65 | 1356.64 | c9+ |
| 1227.61 | 1227.82 | c8+ |
| 2001.01 | 2001.27 | c+116+ |
| 1586.76 | 1586.73 | c+112+ |
| 1685.83 | 1685.64 | c+113+ |
| 943.96 | 943.36 | c152+ |
| 1785.87 | 1785.82 | c14+ |
| 1520.80 | 1520.91 | z15+ |
| 1449.77 | 1449.91 | z14+ |
| 1303.71 | 1303.73 | z+113+ |
| 760.90 | 760.91 | z152+ |
| 1245.68 | 1245.82 | z12+ |
| 1147.62 | 1147.73 | z+111+ |
| 1018.51 | 1018.82 | z10+ |
| 903.49 | 903.91 | z9+ |
| 775.45 | 775.73 | z+18+ |
| 673.40 | 673.45 | z7+ |
| 602.36 | 602.73 | z6+ |
| 545.34 | 545.64 | z5+ |
[M+3H] 3+
c8+
z10+
z12+
z7+
c+17+
c2+
z15+
z6+
c5+
c+112+
z5+
z+111+
z9+
z152+
z+113+
c14+
z14+
c9+
z+18+
c+116+
c152+
c+113+
200
400
600
800
1000
1200
1400
1600
1800
2000
m/z
Supplemental figure 3 M: ETD mass spectrum of [M+3H]3+ ions of glycopeptide TTAFGVKDETAGVTTLK modified at Thr 464 or Thr465 with glycan Δm390. RT = 19.93 min. CV= -45 V.

## Slide 14
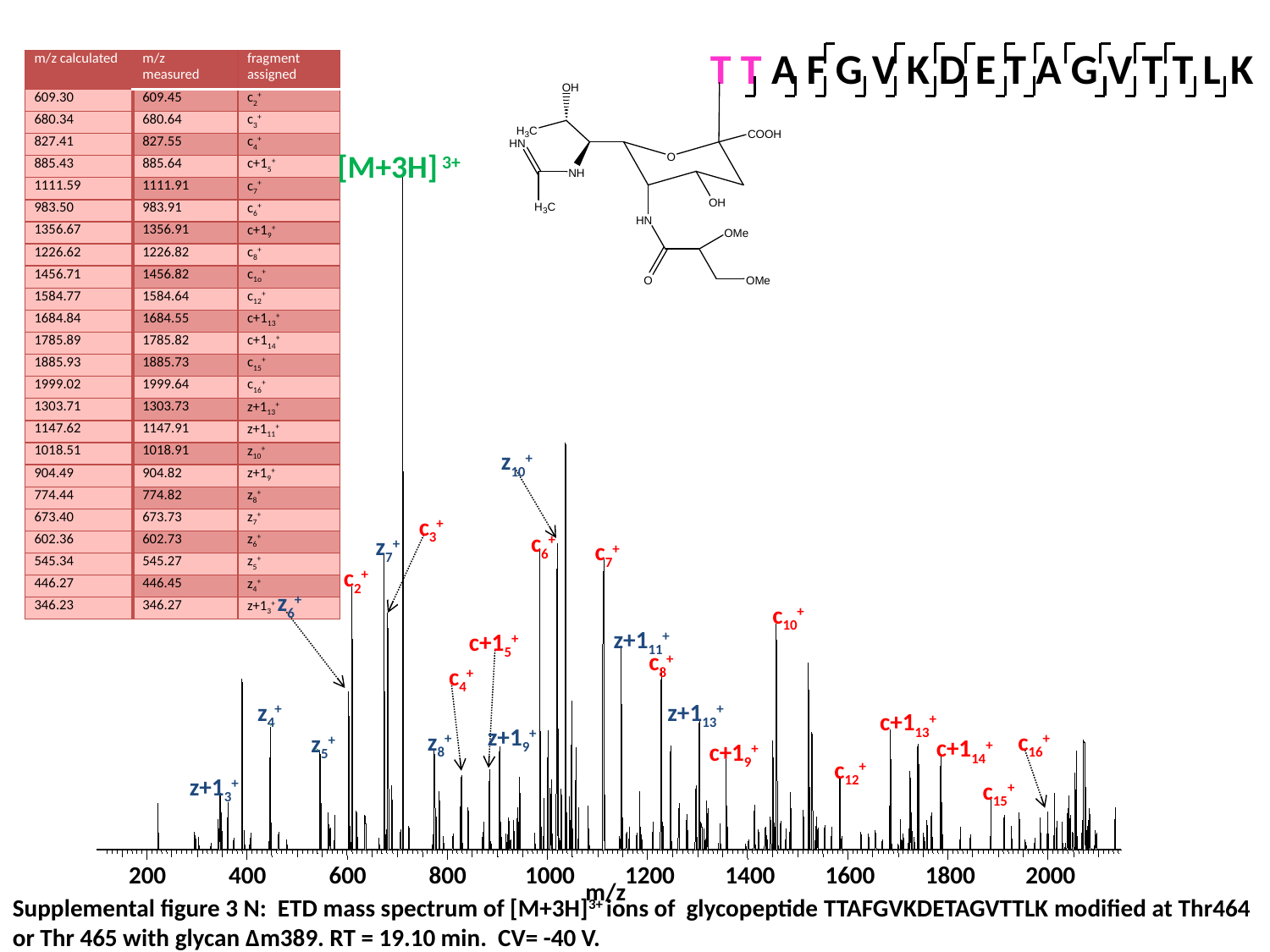

T T A F G V K D E T A G V T T L K
| m/z calculated | m/z measured | fragment assigned |
| --- | --- | --- |
| 609.30 | 609.45 | c2+ |
| 680.34 | 680.64 | c3+ |
| 827.41 | 827.55 | c4+ |
| 885.43 | 885.64 | c+15+ |
| 1111.59 | 1111.91 | c7+ |
| 983.50 | 983.91 | c6+ |
| 1356.67 | 1356.91 | c+19+ |
| 1226.62 | 1226.82 | c8+ |
| 1456.71 | 1456.82 | c1o+ |
| 1584.77 | 1584.64 | c12+ |
| 1684.84 | 1684.55 | c+113+ |
| 1785.89 | 1785.82 | c+114+ |
| 1885.93 | 1885.73 | c15+ |
| 1999.02 | 1999.64 | c16+ |
| 1303.71 | 1303.73 | z+113+ |
| 1147.62 | 1147.91 | z+111+ |
| 1018.51 | 1018.91 | z10+ |
| 904.49 | 904.82 | z+19+ |
| 774.44 | 774.82 | z8+ |
| 673.40 | 673.73 | z7+ |
| 602.36 | 602.73 | z6+ |
| 545.34 | 545.27 | z5+ |
| 446.27 | 446.45 | z4+ |
| 346.23 | 346.27 | z+13+ |
[M+3H] 3+
z10+
c3+
c6+
z7+
c7+
c2+
z6+
c10+
z+111+
c+15+
c8+
c4+
z4+
z+113+
c+113+
z+19+
z8+
c16+
z5+
c+114+
c+19+
c12+
z+13+
c15+
200
400
600
800
1000
1200
1400
1600
1800
2000
m/z
Supplemental figure 3 N: ETD mass spectrum of [M+3H]3+ ions of glycopeptide TTAFGVKDETAGVTTLK modified at Thr464 or Thr 465 with glycan Δm389. RT = 19.10 min. CV= -40 V.

## Slide 15
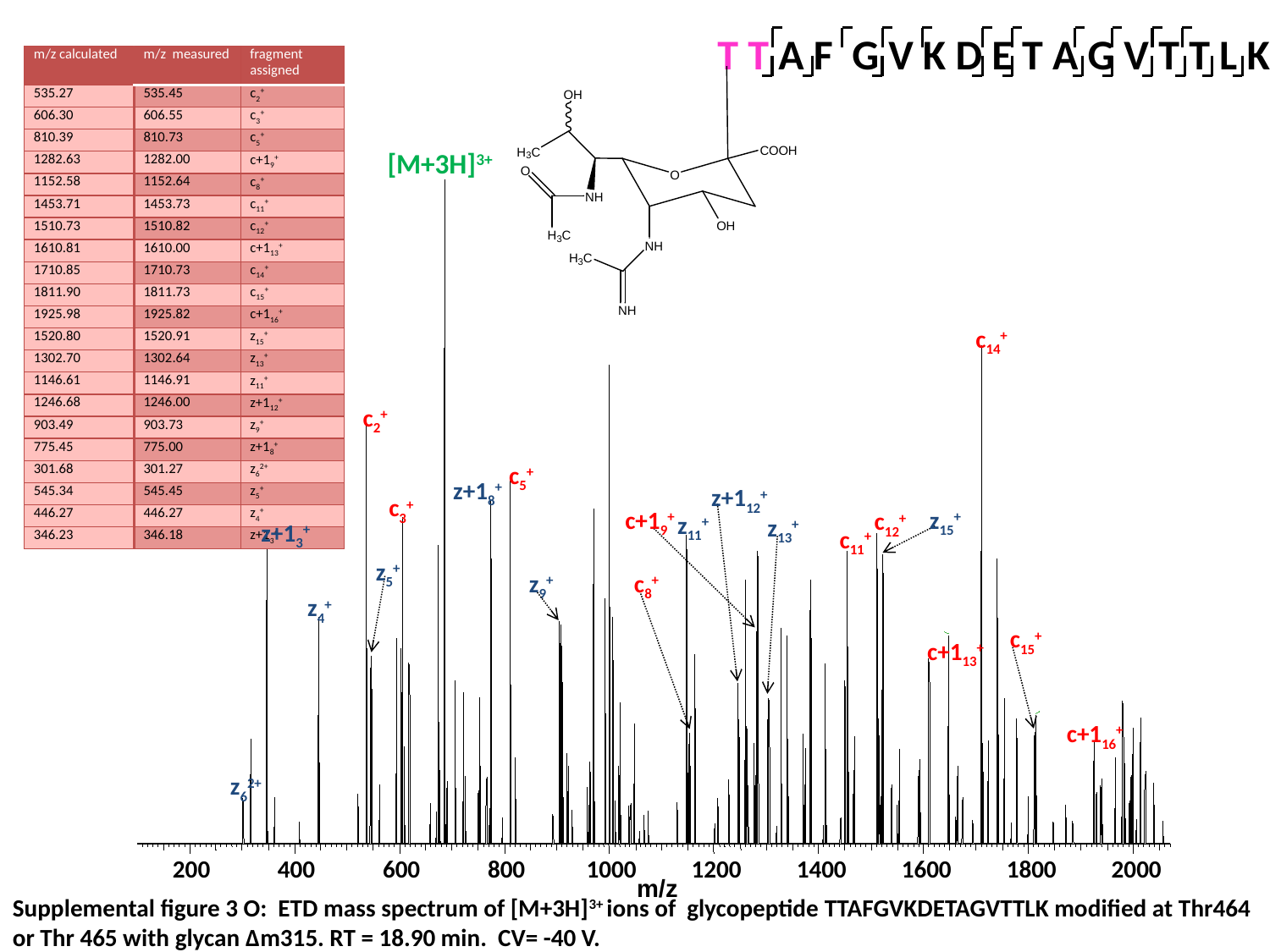

T T A F G V K D E T A G V T T L K
| m/z calculated | m/z measured | fragment assigned |
| --- | --- | --- |
| 535.27 | 535.45 | c2+ |
| 606.30 | 606.55 | c3+ |
| 810.39 | 810.73 | c5+ |
| 1282.63 | 1282.00 | c+19+ |
| 1152.58 | 1152.64 | c8+ |
| 1453.71 | 1453.73 | c11+ |
| 1510.73 | 1510.82 | c12+ |
| 1610.81 | 1610.00 | c+113+ |
| 1710.85 | 1710.73 | c14+ |
| 1811.90 | 1811.73 | c15+ |
| 1925.98 | 1925.82 | c+116+ |
| 1520.80 | 1520.91 | z15+ |
| 1302.70 | 1302.64 | z13+ |
| 1146.61 | 1146.91 | z11+ |
| 1246.68 | 1246.00 | z+112+ |
| 903.49 | 903.73 | z9+ |
| 775.45 | 775.00 | z+18+ |
| 301.68 | 301.27 | z62+ |
| 545.34 | 545.45 | z5+ |
| 446.27 | 446.27 | z4+ |
| 346.23 | 346.18 | z+13+ |
[M+3H]3+
c14+
c2+
c5+
z+18+
z+112+
c3+
z15+
c+19+
c12+
z11+
z13+
z+13+
c11+
z5+
z9+
c8+
z4+
c15+
c+113+
c+116+
z62+
200
400
600
800
1000
1200
1400
1600
1800
2000
m/z
Supplemental figure 3 O: ETD mass spectrum of [M+3H]3+ ions of glycopeptide TTAFGVKDETAGVTTLK modified at Thr464 or Thr 465 with glycan Δm315. RT = 18.90 min. CV= -40 V.
