# Supplementary material for: Comprehensive mapping of O‐glycosylation in flagellin from Campylobacter jejuni 11168: A multienzyme differential ion mobility mass spectrometry approach
Source: Proteomics. 2015 Jun 15;15(16):2733–45. doi: 10.1002/pmic.201400533 (PMC4975691; doi:10.1002/pmic.201400533)
Supplement: Supplementary file 1 — Figure S1. SDS‐PAGE analysis of purified Campylobacter jejuni flagellin protein. 10% SDS‐PAGE gel, stained with Coomassie blue. Lane 1 – MW markers. Lane 2 – cell suspension from C. jejuni strain 11168 culture, Lane 3 – purified flagellin protein Figure S2. Figure S3. Figure S4. Figure S5. Supplemental Table 1: Non‐glycopeptides identified from tryptic digest of flagellin following ETD MS/MS (with and without FAIMS). (Note that where peptides were identified from both replicates, m/zmeas values are given for replicate#1). Supplemental Table 2: Non‐glycopeptides identified from proteinase K digest of flagellin following ETD MS/MS (without FAIMS). (Note that where peptides were identified from both replicates, m/zmeas values are given for replicate#2). Supplemental Table 3: Non‐glycopeptides identified from proteinase K digest of flagellin following ETD MS/MS (with FAIMS). (Note that where peptides were identified from both replicates, m/zmeas values are given for replicate#2). Comprehensive mapping of O‐glycosylation in flagellin from Campylobacter jejuni 11168: A multi‐enzyme differential ion mobility mass spectrometry approach [file PMIC-15-2733-s001.zip › pmic201400533-sup-0004-figure 4.pptx]

## Slide 1
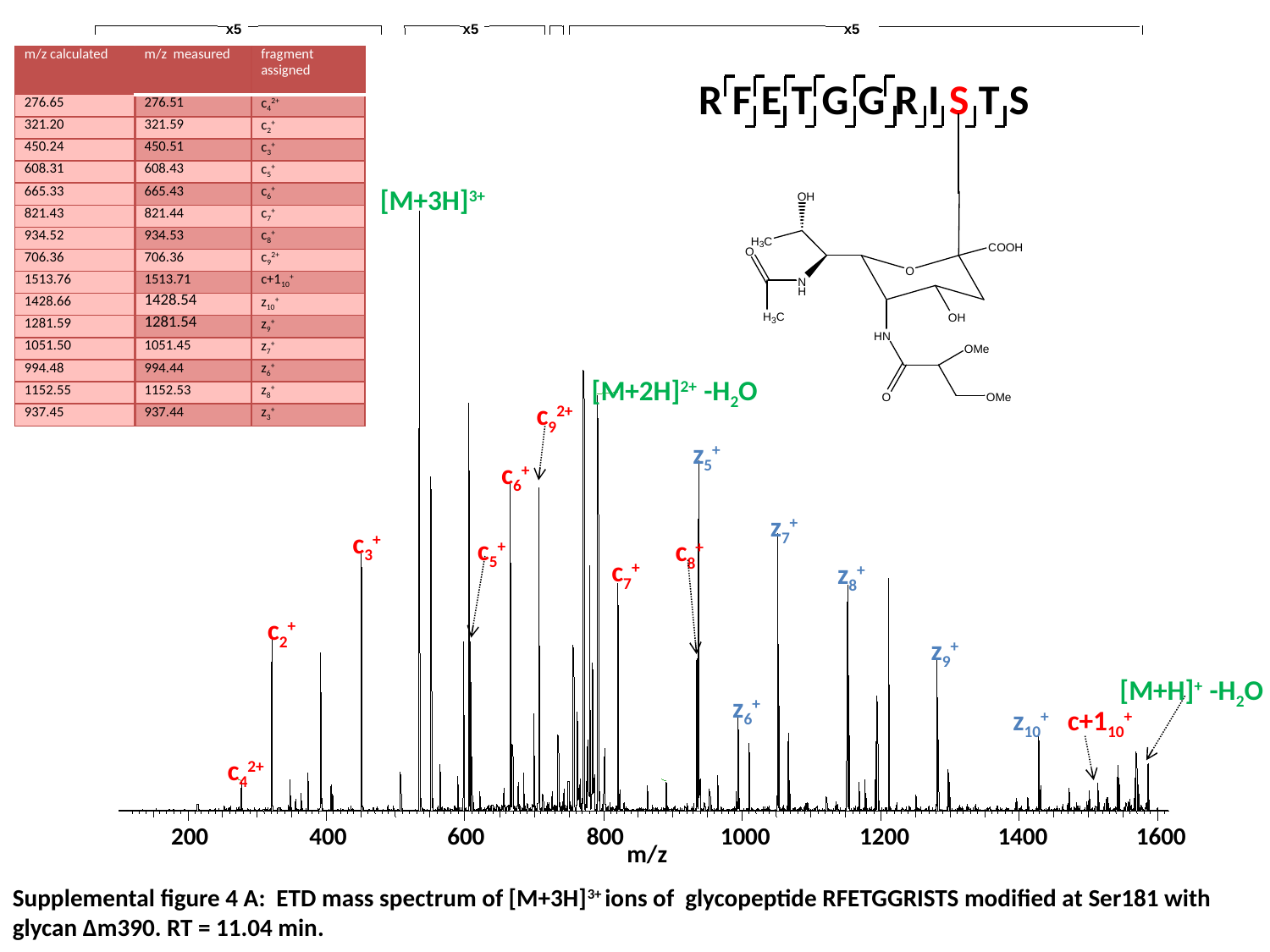

x5
x5
x5
| m/z calculated | m/z measured | fragment assigned |
| --- | --- | --- |
| 276.65 | 276.51 | c42+ |
| 321.20 | 321.59 | c2+ |
| 450.24 | 450.51 | c3+ |
| 608.31 | 608.43 | c5+ |
| 665.33 | 665.43 | c6+ |
| 821.43 | 821.44 | c7+ |
| 934.52 | 934.53 | c8+ |
| 706.36 | 706.36 | c92+ |
| 1513.76 | 1513.71 | c+110+ |
| 1428.66 | 1428.54 | z10+ |
| 1281.59 | 1281.54 | z9+ |
| 1051.50 | 1051.45 | z7+ |
| 994.48 | 994.44 | z6+ |
| 1152.55 | 1152.53 | z8+ |
| 937.45 | 937.44 | z3+ |
R F E T G G R I S T S
[M+3H]3+
[M+2H]2+ -H2O
c92+
z5+
c6+
z7+
c3+
c5+
c8+
c7+
z8+
c2+
z9+
[M+H]+ -H2O
z6+
z10+
c+110+
c42+
200
400
600
800
1000
1200
1400
1600
m/z
Supplemental figure 4 A: ETD mass spectrum of [M+3H]3+ ions of glycopeptide RFETGGRISTS modified at Ser181 with glycan Δm390. RT = 11.04 min.

## Slide 2
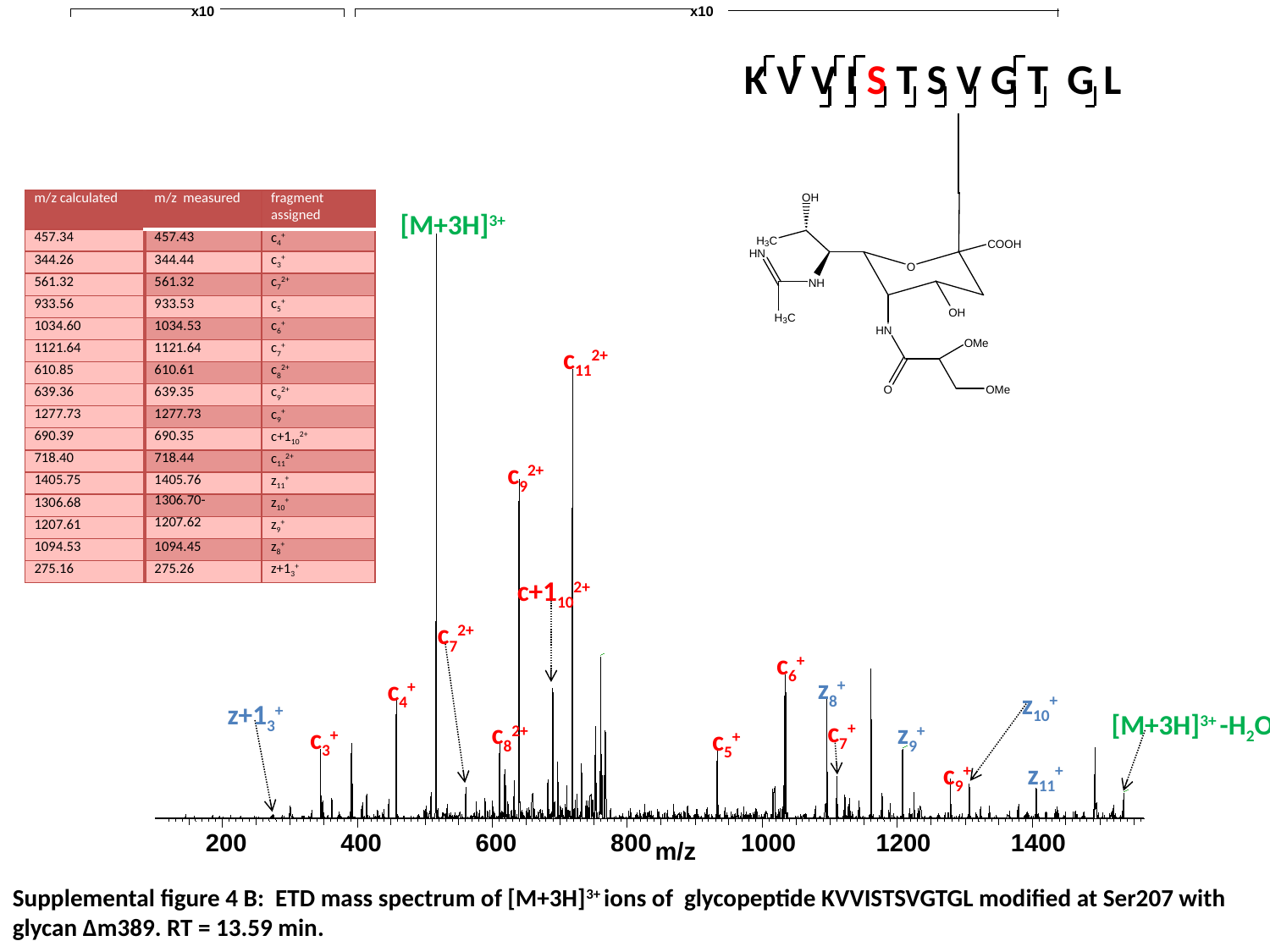

x10
x10
K V V I S T S V G T G L
| m/z calculated | m/z measured | fragment assigned |
| --- | --- | --- |
| 457.34 | 457.43 | c4+ |
| 344.26 | 344.44 | c3+ |
| 561.32 | 561.32 | c72+ |
| 933.56 | 933.53 | c5+ |
| 1034.60 | 1034.53 | c6+ |
| 1121.64 | 1121.64 | c7+ |
| 610.85 | 610.61 | c82+ |
| 639.36 | 639.35 | c92+ |
| 1277.73 | 1277.73 | c9+ |
| 690.39 | 690.35 | c+1102+ |
| 718.40 | 718.44 | c112+ |
| 1405.75 | 1405.76 | z11+ |
| 1306.68 | 1306.70- | z10+ |
| 1207.61 | 1207.62 | z9+ |
| 1094.53 | 1094.45 | z8+ |
| 275.16 | 275.26 | z+13+ |
[M+3H]3+
c112+
c92+
c+1102+
c72+
c6+
z8+
c4+
z10+
z+13+
[M+3H]3+ -H2O
c7+
c82+
z9+
c3+
c5+
c9+
z11+
200
400
600
800
1000
1200
1400
m/z
Supplemental figure 4 B: ETD mass spectrum of [M+3H]3+ ions of glycopeptide KVVISTSVGTGL modified at Ser207 with glycan Δm389. RT = 13.59 min.

## Slide 3
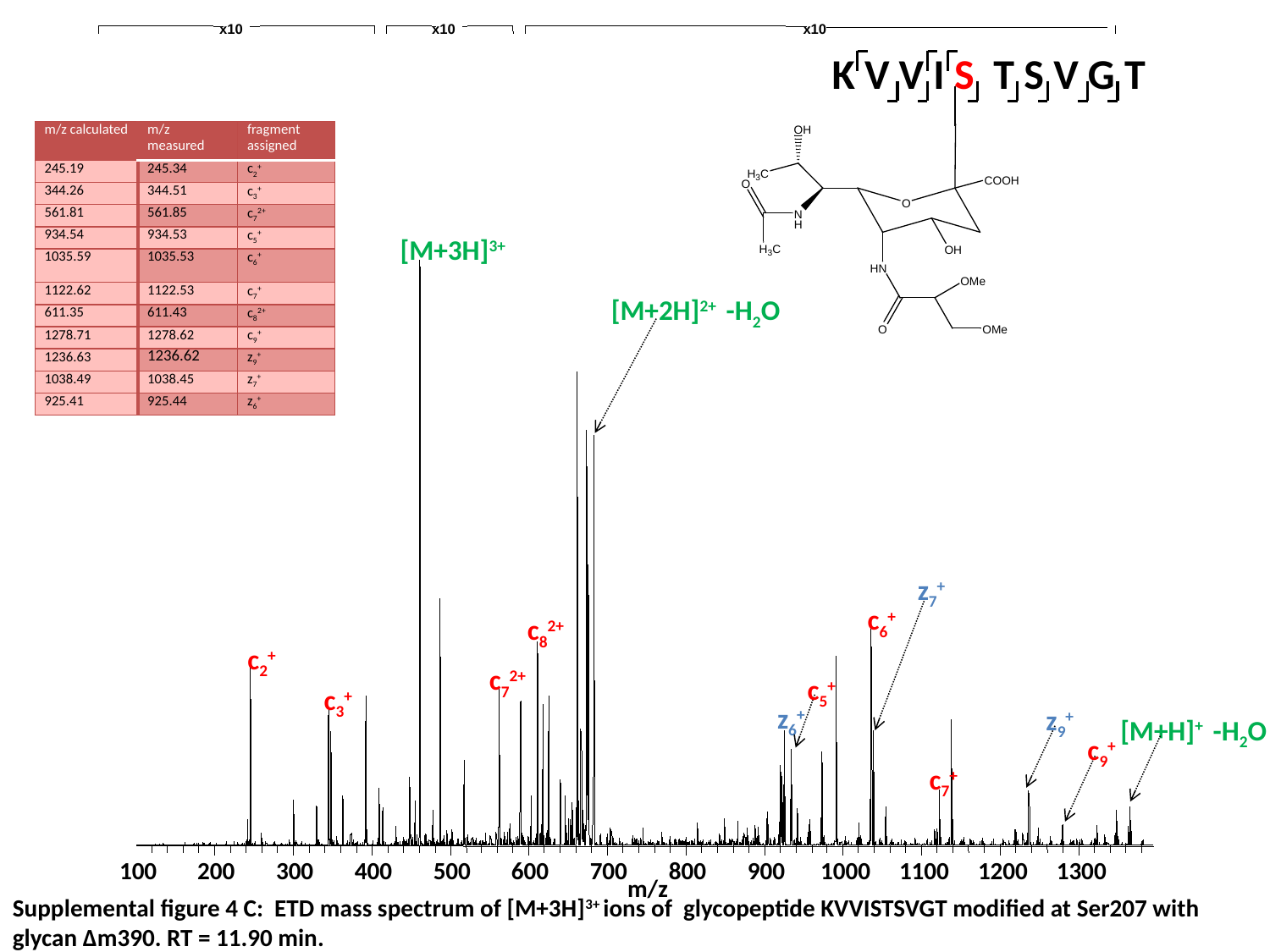

x10
x10
x10
K V V I S T S V G T
| m/z calculated | m/z measured | fragment assigned |
| --- | --- | --- |
| 245.19 | 245.34 | c2+ |
| 344.26 | 344.51 | c3+ |
| 561.81 | 561.85 | c72+ |
| 934.54 | 934.53 | c5+ |
| 1035.59 | 1035.53 | c6+ |
| 1122.62 | 1122.53 | c7+ |
| 611.35 | 611.43 | c82+ |
| 1278.71 | 1278.62 | c9+ |
| 1236.63 | 1236.62 | z9+ |
| 1038.49 | 1038.45 | z7+ |
| 925.41 | 925.44 | z6+ |
[M+3H]3+
[M+2H]2+ -H2O
z7+
c6+
c82+
c2+
c72+
c5+
c3+
z6+
z9+
[M+H]+ -H2O
c9+
c7+
100
200
300
400
500
600
700
800
900
1000
1100
1200
1300
m/z
Supplemental figure 4 C: ETD mass spectrum of [M+3H]3+ ions of glycopeptide KVVISTSVGT modified at Ser207 with glycan Δm390. RT = 11.90 min.

## Slide 4
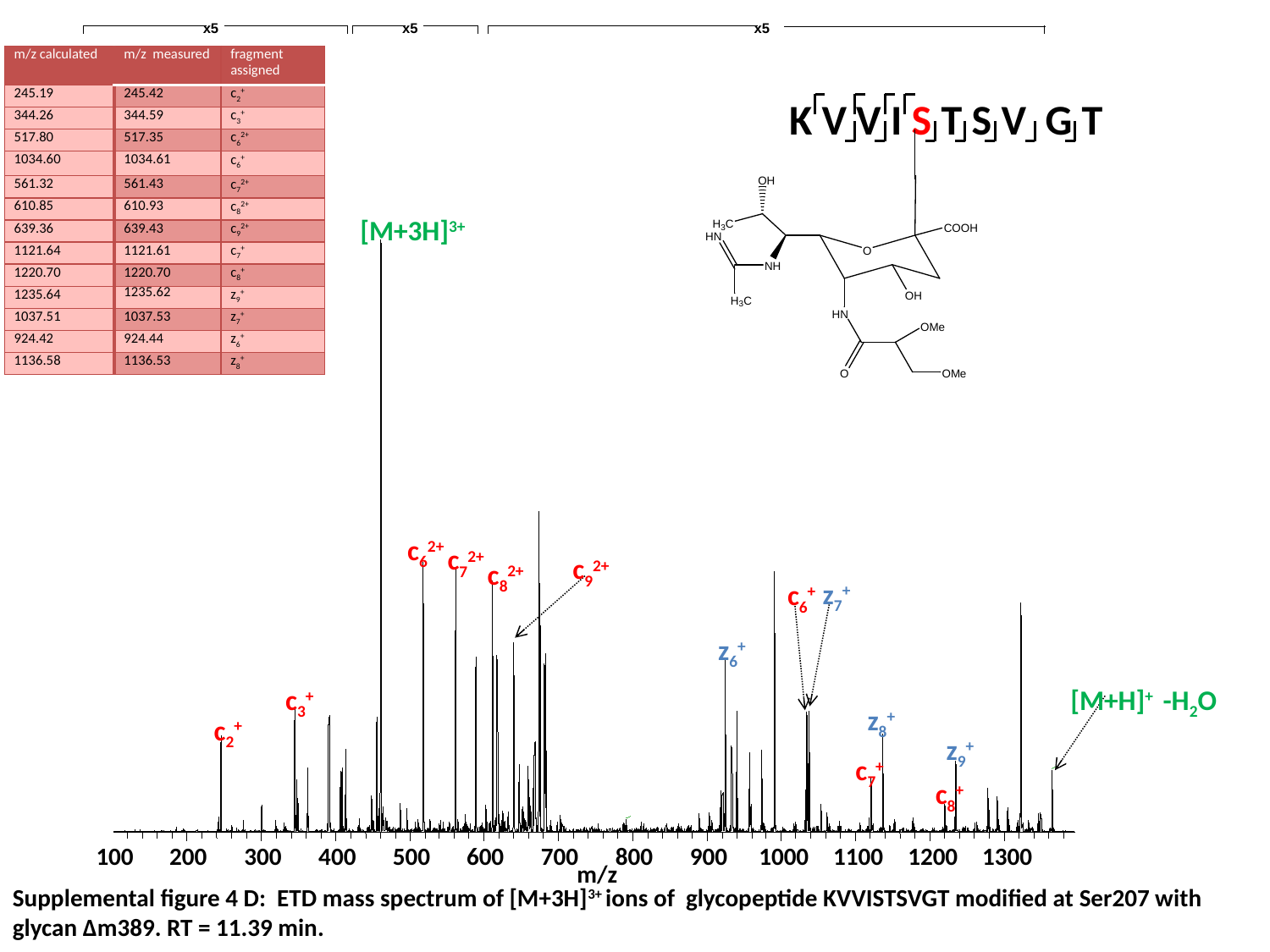

x5
x5
x5
| m/z calculated | m/z measured | fragment assigned |
| --- | --- | --- |
| 245.19 | 245.42 | c2+ |
| 344.26 | 344.59 | c3+ |
| 517.80 | 517.35 | c62+ |
| 1034.60 | 1034.61 | c6+ |
| 561.32 | 561.43 | c72+ |
| 610.85 | 610.93 | c82+ |
| 639.36 | 639.43 | c92+ |
| 1121.64 | 1121.61 | c7+ |
| 1220.70 | 1220.70 | c8+ |
| 1235.64 | 1235.62 | z9+ |
| 1037.51 | 1037.53 | z7+ |
| 924.42 | 924.44 | z6+ |
| 1136.58 | 1136.53 | z8+ |
K V V I S T S V G T
[M+3H]3+
c62+
c72+
c92+
c82+
z7+
c6+
z6+
c3+
[M+H]+ -H2O
z8+
c2+
z9+
c7+
c8+
100
200
300
400
500
600
700
800
900
1000
1100
1200
1300
m/z
Supplemental figure 4 D: ETD mass spectrum of [M+3H]3+ ions of glycopeptide KVVISTSVGT modified at Ser207 with glycan Δm389. RT = 11.39 min.

## Slide 5
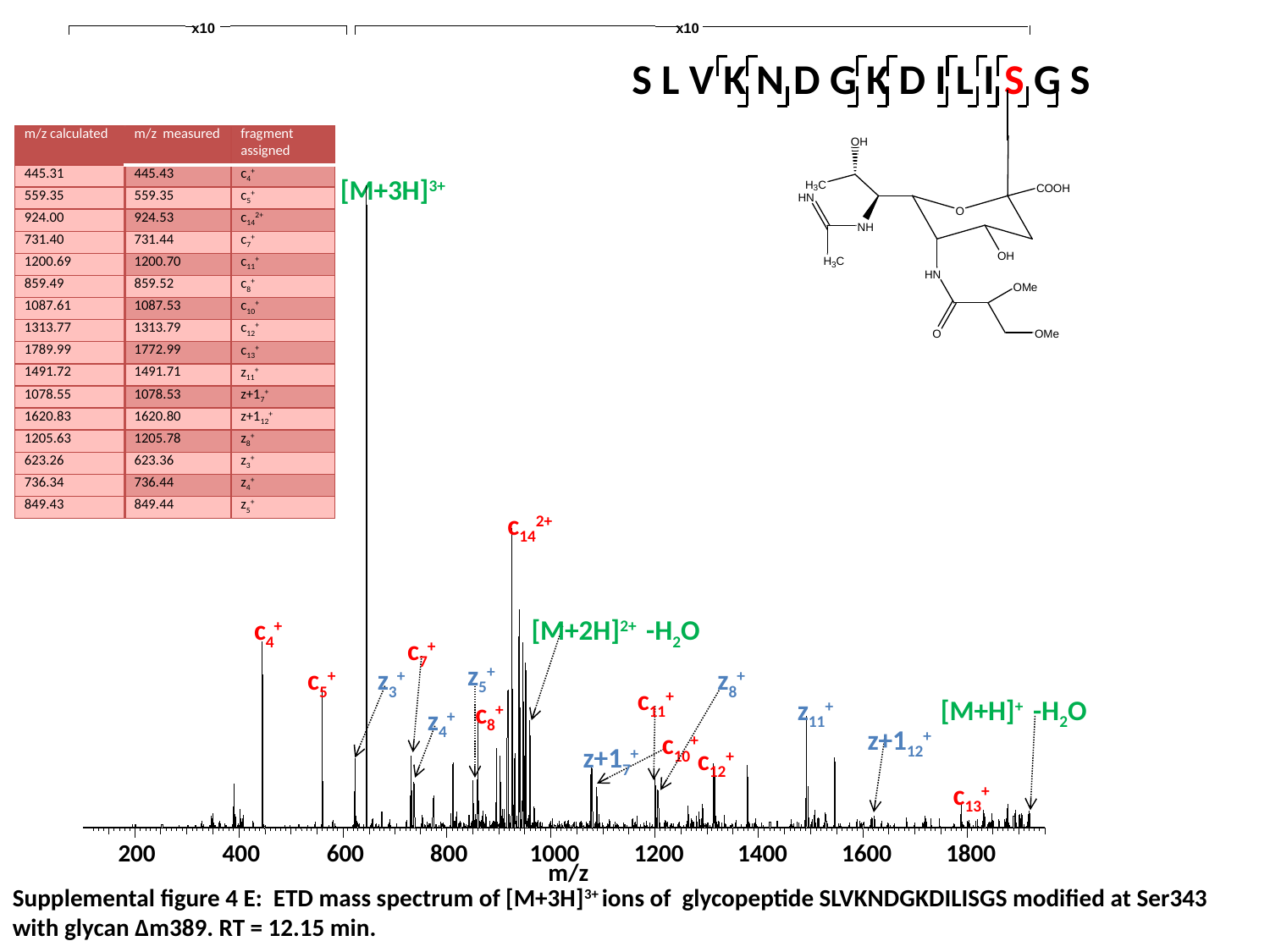

x10
x10
S L V K N D G K D I L I S G S
| m/z calculated | m/z measured | fragment assigned |
| --- | --- | --- |
| 445.31 | 445.43 | c4+ |
| 559.35 | 559.35 | c5+ |
| 924.00 | 924.53 | c142+ |
| 731.40 | 731.44 | c7+ |
| 1200.69 | 1200.70 | c11+ |
| 859.49 | 859.52 | c8+ |
| 1087.61 | 1087.53 | c10+ |
| 1313.77 | 1313.79 | c12+ |
| 1789.99 | 1772.99 | c13+ |
| 1491.72 | 1491.71 | z11+ |
| 1078.55 | 1078.53 | z+17+ |
| 1620.83 | 1620.80 | z+112+ |
| 1205.63 | 1205.78 | z8+ |
| 623.26 | 623.36 | z3+ |
| 736.34 | 736.44 | z4+ |
| 849.43 | 849.44 | z5+ |
[M+3H]3+
c142+
c4+
[M+2H]2+ -H2O
c7+
z5+
c5+
z3+
z8+
c11+
z11+
[M+H]+ -H2O
c8+
z4+
z+112+
c10+
z+17+
c12+
c13+
200
400
600
800
1000
1200
1400
1600
1800
m/z
Supplemental figure 4 E: ETD mass spectrum of [M+3H]3+ ions of glycopeptide SLVKNDGKDILISGS modified at Ser343 with glycan Δm389. RT = 12.15 min.

## Slide 6
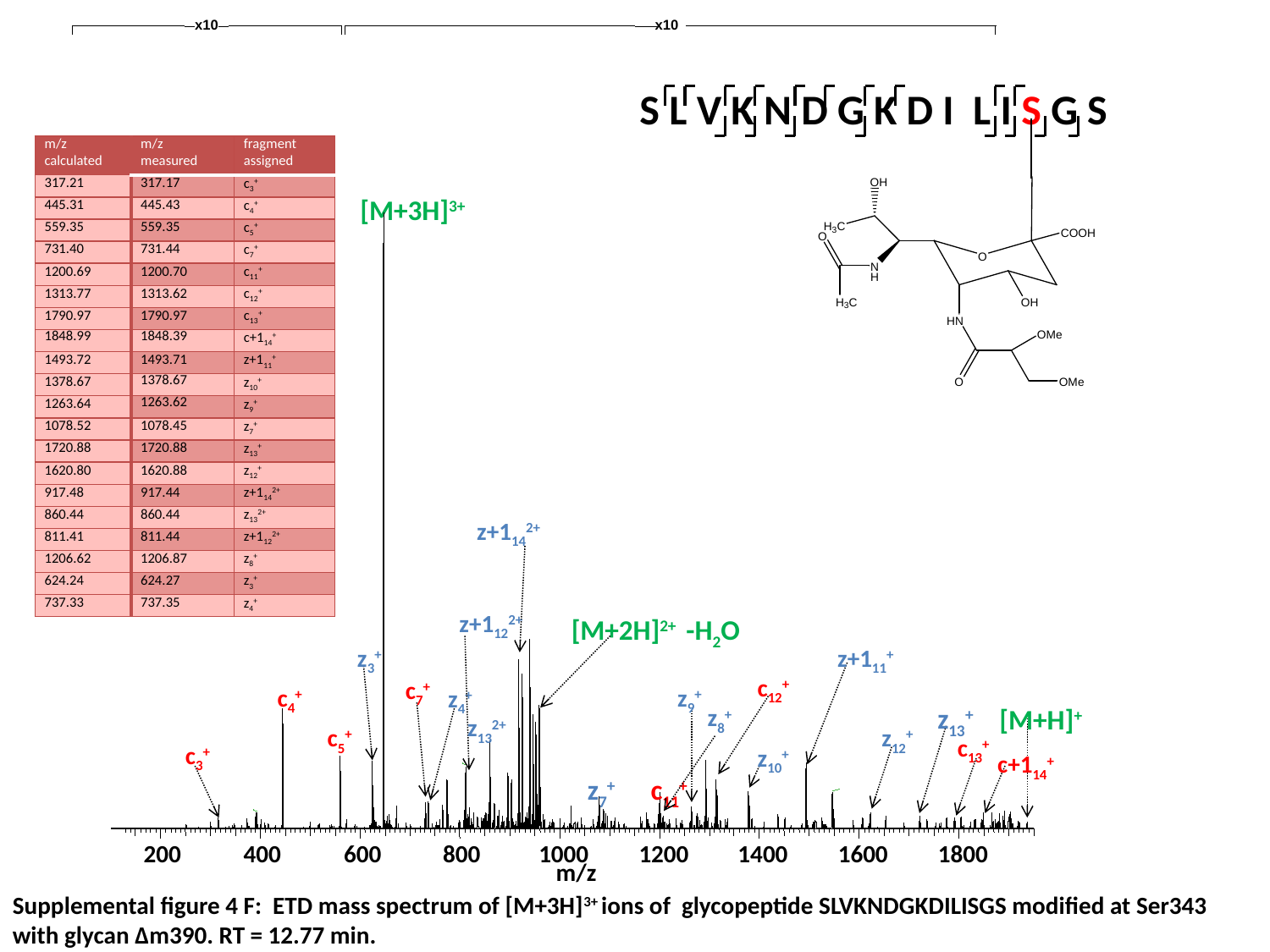

x10
x10
S L V K N D G K D I L I S G S
| m/z calculated | m/z measured | fragment assigned |
| --- | --- | --- |
| 317.21 | 317.17 | c3+ |
| 445.31 | 445.43 | c4+ |
| 559.35 | 559.35 | c5+ |
| 731.40 | 731.44 | c7+ |
| 1200.69 | 1200.70 | c11+ |
| 1313.77 | 1313.62 | c12+ |
| 1790.97 | 1790.97 | c13+ |
| 1848.99 | 1848.39 | c+114+ |
| 1493.72 | 1493.71 | z+111+ |
| 1378.67 | 1378.67 | z10+ |
| 1263.64 | 1263.62 | z9+ |
| 1078.52 | 1078.45 | z7+ |
| 1720.88 | 1720.88 | z13+ |
| 1620.80 | 1620.88 | z12+ |
| 917.48 | 917.44 | z+1142+ |
| 860.44 | 860.44 | z132+ |
| 811.41 | 811.44 | z+1122+ |
| 1206.62 | 1206.87 | z8+ |
| 624.24 | 624.27 | z3+ |
| 737.33 | 737.35 | z4+ |
[M+3H]3+
z+1142+
z+1122+
[M+2H]2+ -H2O
z3+
z+111+
c12+
c7+
c4+
z9+
z4+
z13+
z8+
[M+H]+
z132+
c5+
z12+
c13+
c3+
z10+
c+114+
z7+
c11+
1200
1400
1600
1800
200
400
600
800
1000
m/z
Supplemental figure 4 F: ETD mass spectrum of [M+3H]3+ ions of glycopeptide SLVKNDGKDILISGS modified at Ser343 with glycan Δm390. RT = 12.77 min.

## Slide 7
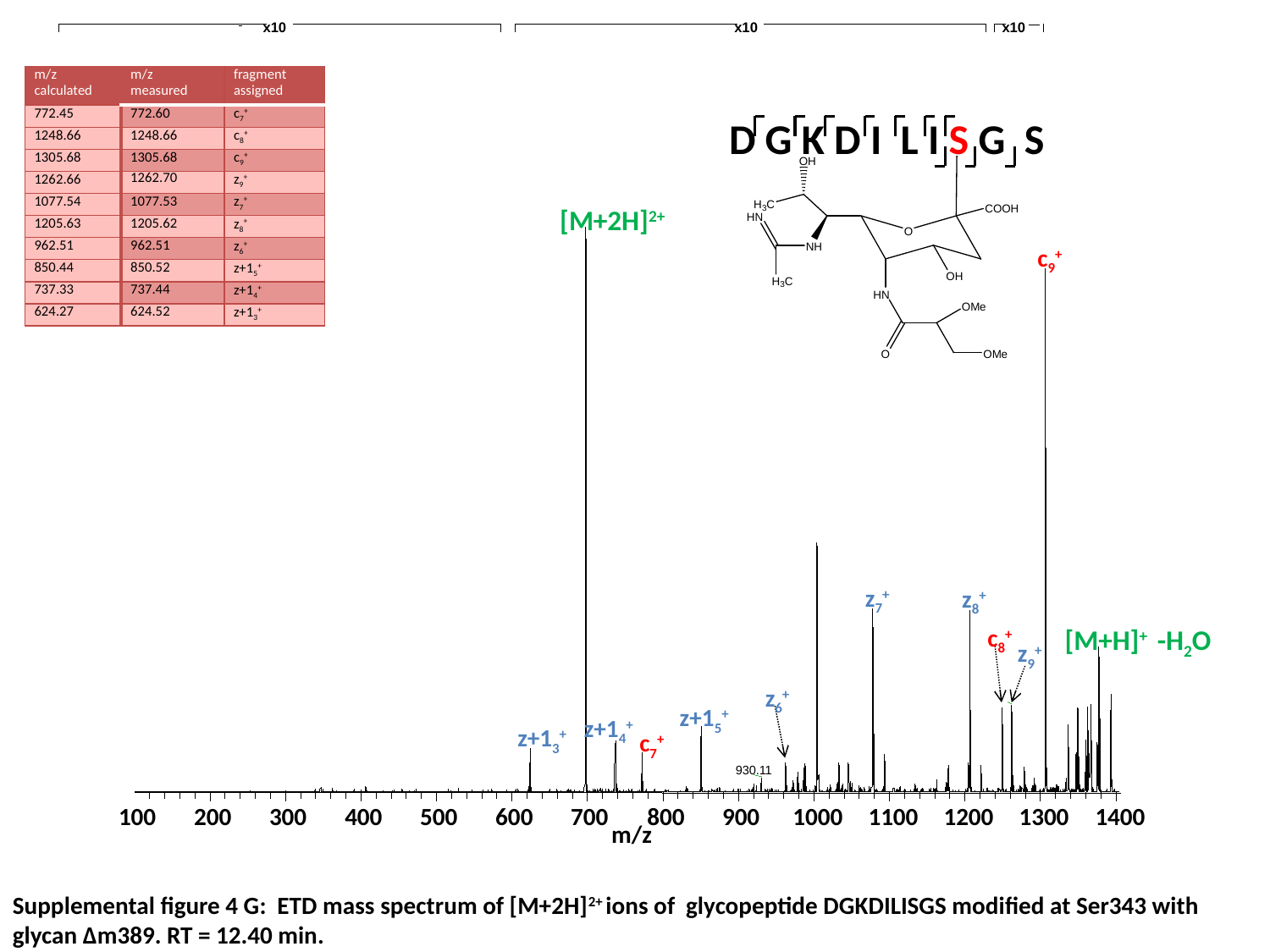

x10
x10
x10
| m/z calculated | m/z measured | fragment assigned |
| --- | --- | --- |
| 772.45 | 772.60 | c7+ |
| 1248.66 | 1248.66 | c8+ |
| 1305.68 | 1305.68 | c9+ |
| 1262.66 | 1262.70 | z9+ |
| 1077.54 | 1077.53 | z7+ |
| 1205.63 | 1205.62 | z8+ |
| 962.51 | 962.51 | z6+ |
| 850.44 | 850.52 | z+15+ |
| 737.33 | 737.44 | z+14+ |
| 624.27 | 624.52 | z+13+ |
D G K D I L I S G S
[M+2H]2+
c9+
z7+
z8+
c8+
[M+H]+ -H2O
z9+
z6+
z+15+
z+14+
z+13+
c7+
930.11
100
200
300
400
500
600
700
800
900
1000
1100
1200
1300
1400
m/z
Supplemental figure 4 G: ETD mass spectrum of [M+2H]2+ ions of glycopeptide DGKDILISGS modified at Ser343 with glycan Δm389. RT = 12.40 min.

## Slide 8
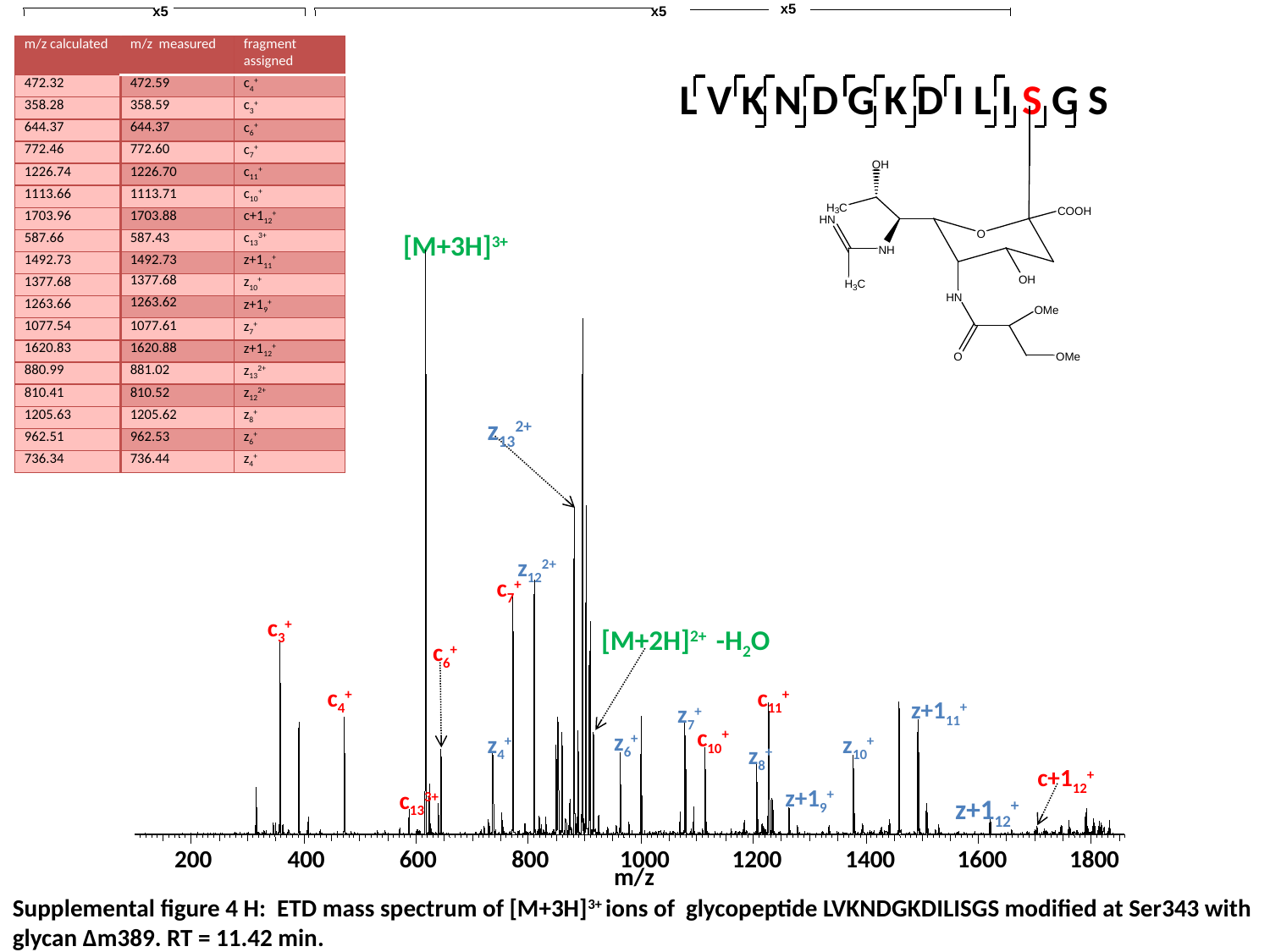

x5
x5
x5
| m/z calculated | m/z measured | fragment assigned |
| --- | --- | --- |
| 472.32 | 472.59 | c4+ |
| 358.28 | 358.59 | c3+ |
| 644.37 | 644.37 | c6+ |
| 772.46 | 772.60 | c7+ |
| 1226.74 | 1226.70 | c11+ |
| 1113.66 | 1113.71 | c10+ |
| 1703.96 | 1703.88 | c+112+ |
| 587.66 | 587.43 | c133+ |
| 1492.73 | 1492.73 | z+111+ |
| 1377.68 | 1377.68 | z10+ |
| 1263.66 | 1263.62 | z+19+ |
| 1077.54 | 1077.61 | z7+ |
| 1620.83 | 1620.88 | z+112+ |
| 880.99 | 881.02 | z132+ |
| 810.41 | 810.52 | z122+ |
| 1205.63 | 1205.62 | z8+ |
| 962.51 | 962.53 | z6+ |
| 736.34 | 736.44 | z4+ |
L V K N D G K D I L I S G S
[M+3H]3+
z132+
z122+
c7+
c3+
[M+2H]2+ -H2O
c6+
c4+
c11+
z+111+
z7+
c10+
z6+
z4+
z10+
z8+
c+112+
z+19+
c133+
z+112+
200
400
600
800
1000
1200
1400
1600
1800
m/z
Supplemental figure 4 H: ETD mass spectrum of [M+3H]3+ ions of glycopeptide LVKNDGKDILISGS modified at Ser343 with glycan Δm389. RT = 11.42 min.

## Slide 9
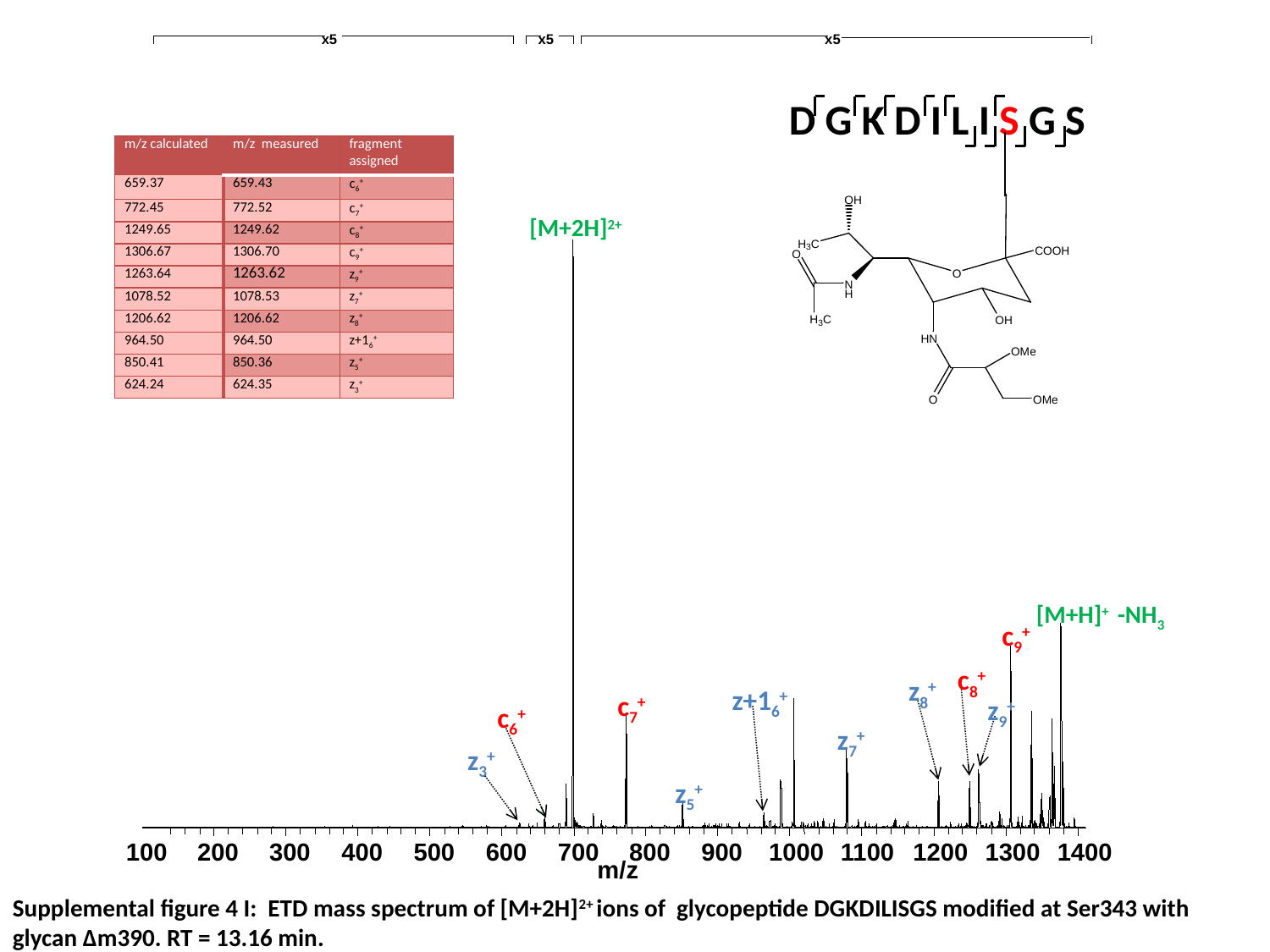

x5
x5
x5
D G K D I L I S G S
| m/z calculated | m/z measured | fragment assigned |
| --- | --- | --- |
| 659.37 | 659.43 | c6+ |
| 772.45 | 772.52 | c7+ |
| 1249.65 | 1249.62 | c8+ |
| 1306.67 | 1306.70 | c9+ |
| 1263.64 | 1263.62 | z9+ |
| 1078.52 | 1078.53 | z7+ |
| 1206.62 | 1206.62 | z8+ |
| 964.50 | 964.50 | z+16+ |
| 850.41 | 850.36 | z5+ |
| 624.24 | 624.35 | z3+ |
[M+2H]2+
[M+H]+ -NH3
c9+
c8+
z8+
z+16+
c7+
z9+
c6+
z7+
z3+
z5+
100
200
300
400
500
600
700
800
900
1000
1100
1200
1300
1400
m/z
Supplemental figure 4 I: ETD mass spectrum of [M+2H]2+ ions of glycopeptide DGKDILISGS modified at Ser343 with glycan Δm390. RT = 13.16 min.

## Slide 10
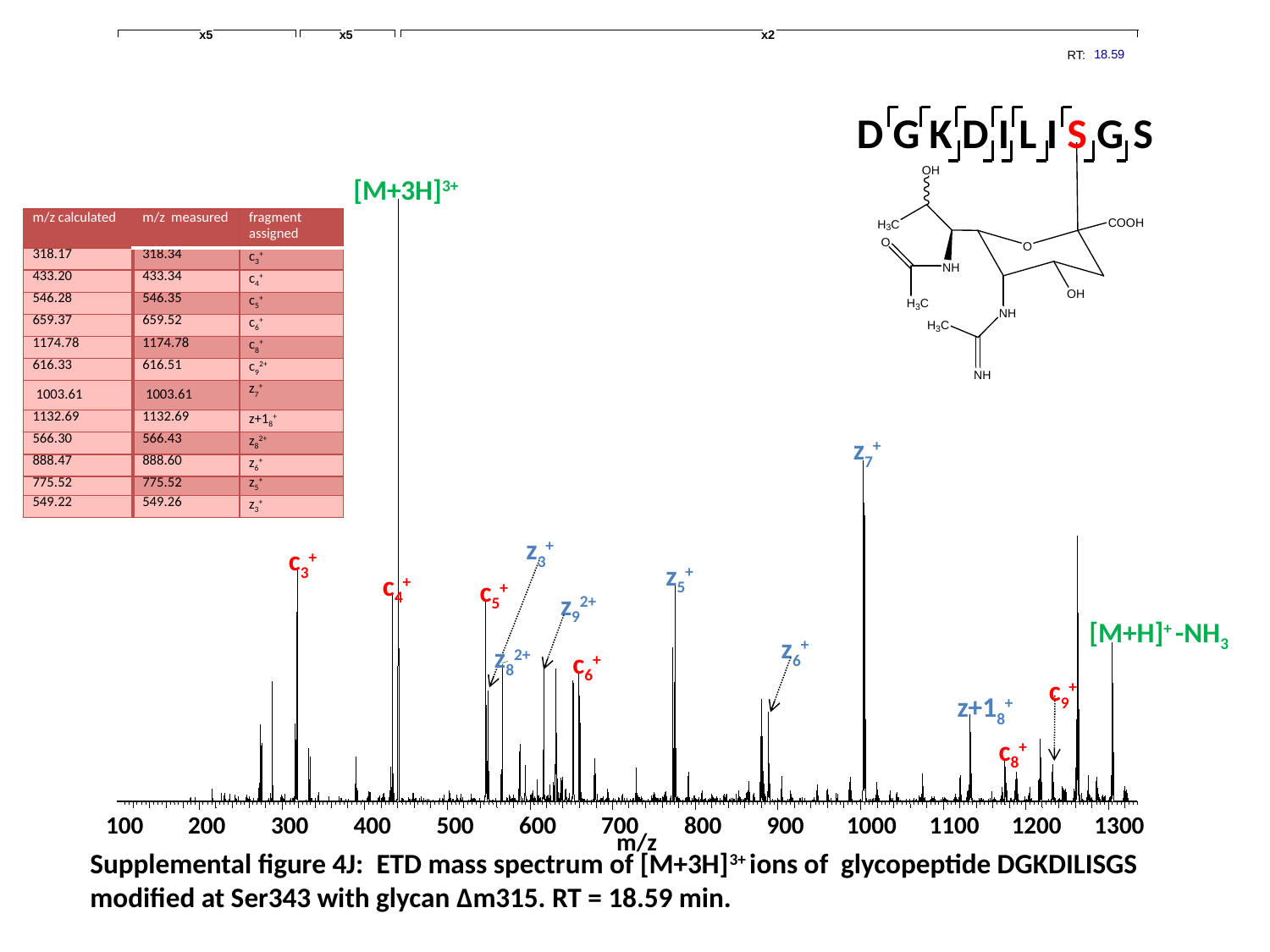

x5
x5
x2
18.59
RT:
D G K D I L I S G S
[M+3H]3+
| m/z calculated | m/z measured | fragment assigned |
| --- | --- | --- |
| 318.17 | 318.34 | c3+ |
| 433.20 | 433.34 | c4+ |
| 546.28 | 546.35 | c5+ |
| 659.37 | 659.52 | c6+ |
| 1174.78 | 1174.78 | c8+ |
| 616.33 | 616.51 | c92+ |
| 1003.61 | 1003.61 | z7+ |
| 1132.69 | 1132.69 | z+18+ |
| 566.30 | 566.43 | z82+ |
| 888.47 | 888.60 | z6+ |
| 775.52 | 775.52 | z5+ |
| 549.22 | 549.26 | z3+ |
z7+
z3+
c3+
z5+
c4+
c5+
z92+
[M+H]+ -NH3
z6+
z82+
c6+
c9+
z+18+
c8+
100
200
300
400
500
600
700
800
900
1000
1100
1200
1300
m/z
Supplemental figure 4J: ETD mass spectrum of [M+3H]3+ ions of glycopeptide DGKDILISGS modified at Ser343 with glycan Δm315. RT = 18.59 min.

## Slide 11
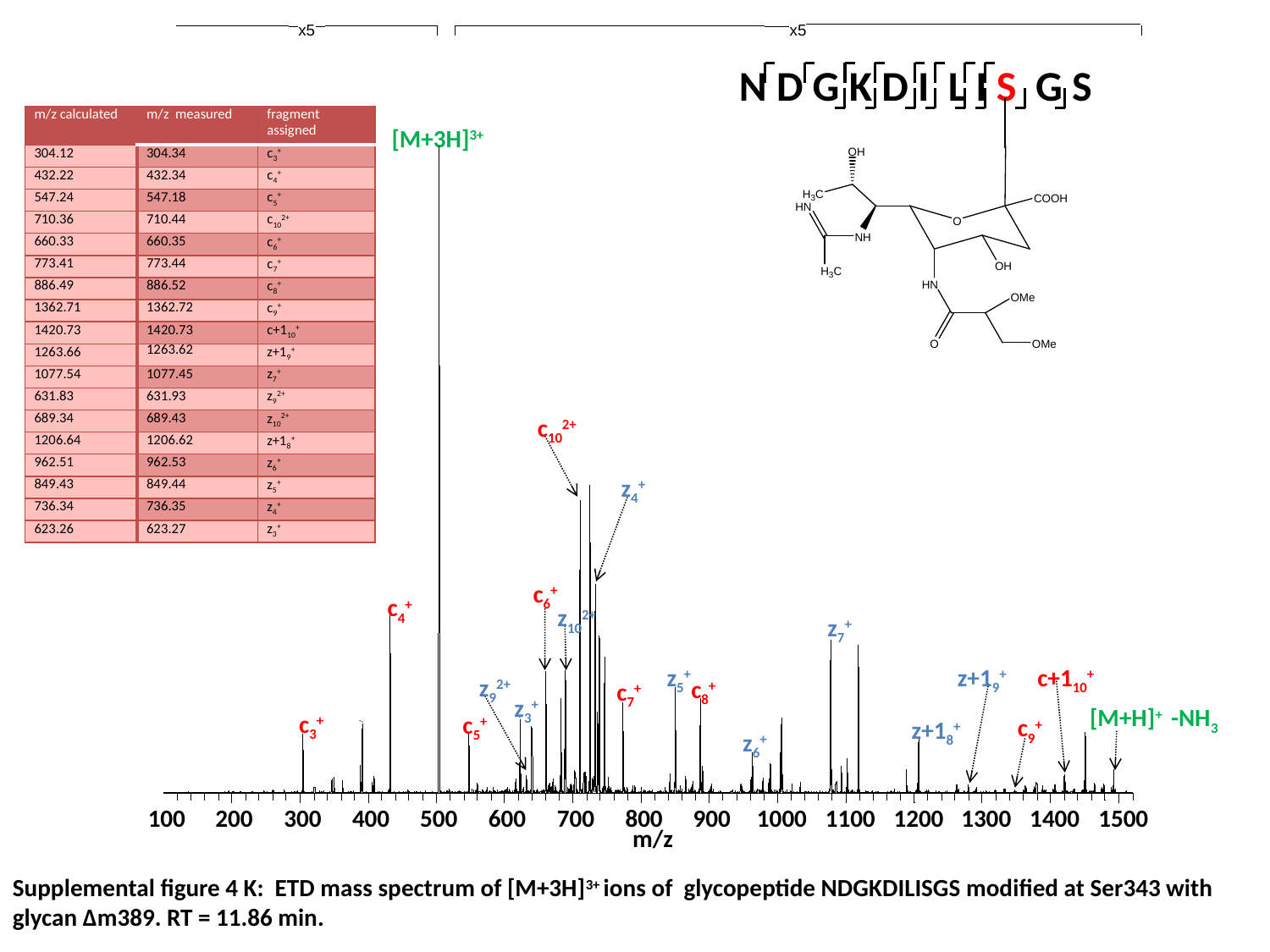

x5
x5
N D G K D I L I S G S
| m/z calculated | m/z measured | fragment assigned |
| --- | --- | --- |
| 304.12 | 304.34 | c3+ |
| 432.22 | 432.34 | c4+ |
| 547.24 | 547.18 | c5+ |
| 710.36 | 710.44 | c102+ |
| 660.33 | 660.35 | c6+ |
| 773.41 | 773.44 | c7+ |
| 886.49 | 886.52 | c8+ |
| 1362.71 | 1362.72 | c9+ |
| 1420.73 | 1420.73 | c+110+ |
| 1263.66 | 1263.62 | z+19+ |
| 1077.54 | 1077.45 | z7+ |
| 631.83 | 631.93 | z92+ |
| 689.34 | 689.43 | z102+ |
| 1206.64 | 1206.62 | z+18+ |
| 962.51 | 962.53 | z6+ |
| 849.43 | 849.44 | z5+ |
| 736.34 | 736.35 | z4+ |
| 623.26 | 623.27 | z3+ |
[M+3H]3+
c102+
z4+
c6+
c4+
z102+
z7+
z+19+
z5+
c+110+
z92+
c8+
c7+
z3+
[M+H]+ -NH3
c3+
c5+
c9+
z+18+
z6+
100
200
300
400
500
600
700
800
900
1000
1100
1200
1300
1400
1500
m/z
Supplemental figure 4 K: ETD mass spectrum of [M+3H]3+ ions of glycopeptide NDGKDILISGS modified at Ser343 with glycan Δm389. RT = 11.86 min.

## Slide 12
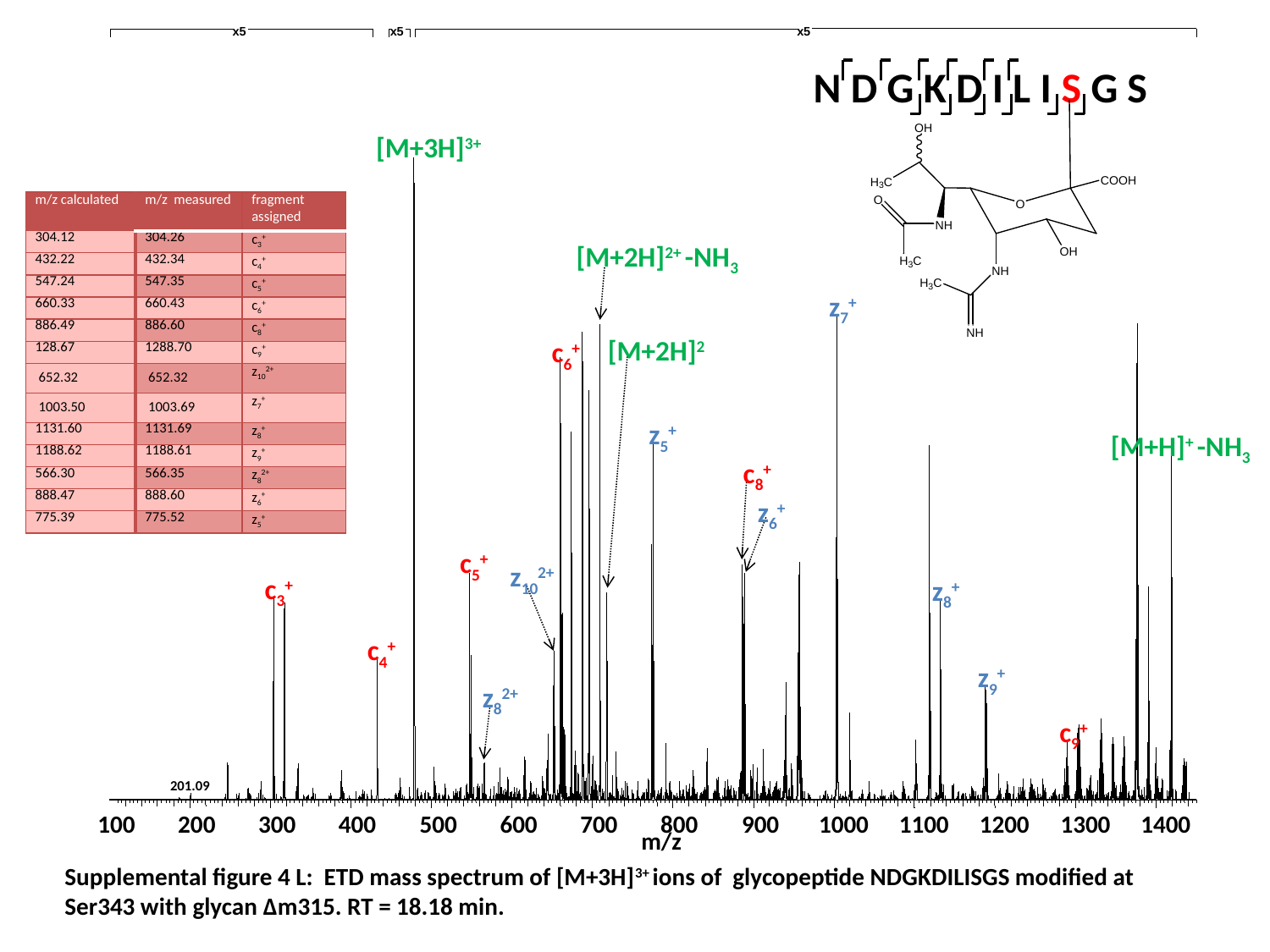

x5
x5
x5
N D G K D I L I S G S
[M+3H]3+
| m/z calculated | m/z measured | fragment assigned |
| --- | --- | --- |
| 304.12 | 304.26 | c3+ |
| 432.22 | 432.34 | c4+ |
| 547.24 | 547.35 | c5+ |
| 660.33 | 660.43 | c6+ |
| 886.49 | 886.60 | c8+ |
| 128.67 | 1288.70 | c9+ |
| 652.32 | 652.32 | z102+ |
| 1003.50 | 1003.69 | z7+ |
| 1131.60 | 1131.69 | z8+ |
| 1188.62 | 1188.61 | z9+ |
| 566.30 | 566.35 | z82+ |
| 888.47 | 888.60 | z6+ |
| 775.39 | 775.52 | z5+ |
[M+2H]2+ -NH3
z7+
[M+2H]2
c6+
z5+
[M+H]+ -NH3
c8+
z6+
c5+
z102+
c3+
z8+
c4+
z9+
z82+
c9+
201.09
100
200
300
400
500
600
700
800
900
1000
1100
1200
1300
1400
m/z
Supplemental figure 4 L: ETD mass spectrum of [M+3H]3+ ions of glycopeptide NDGKDILISGS modified at Ser343 with glycan Δm315. RT = 18.18 min.

## Slide 13
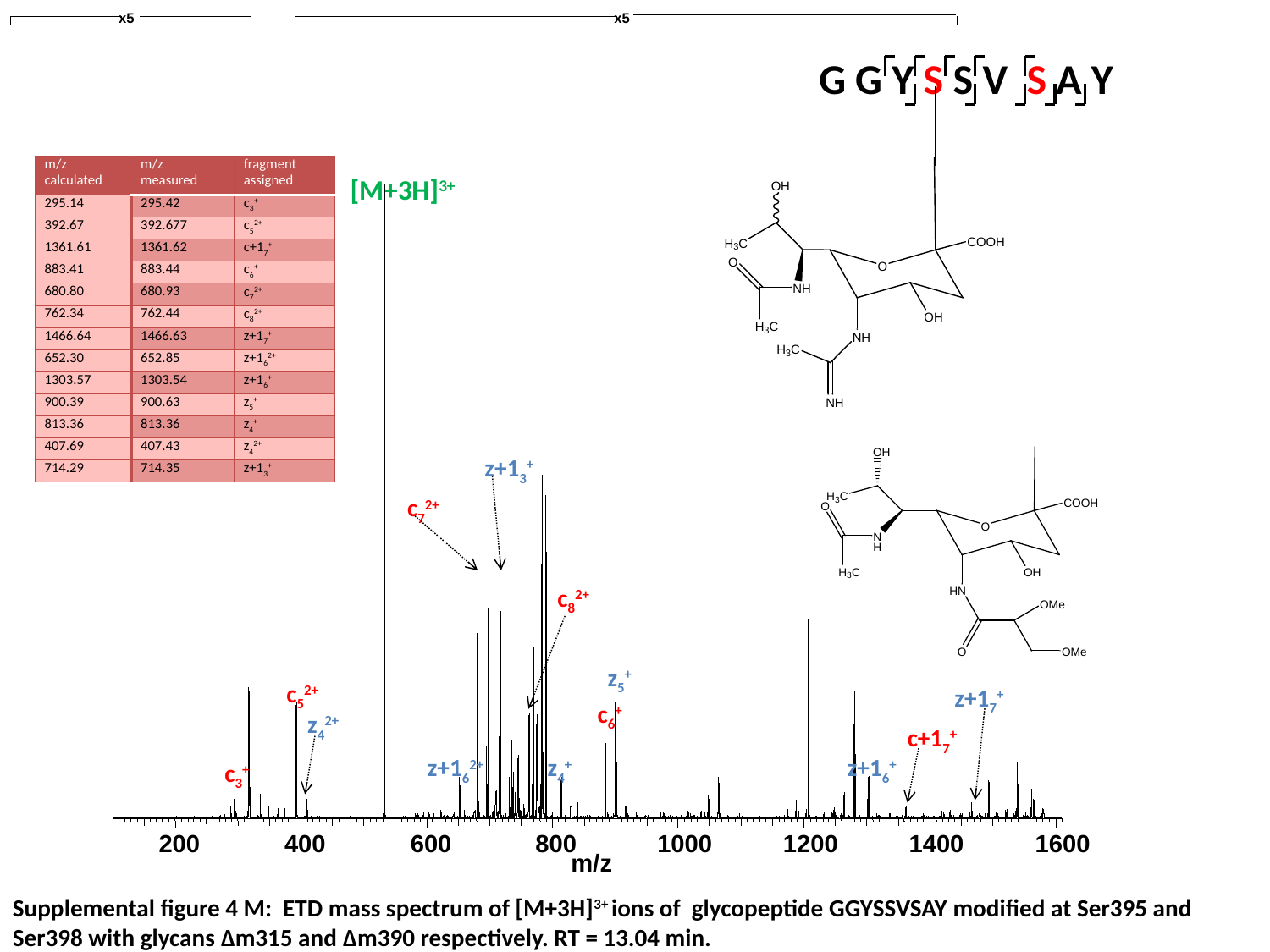

x5
x5
G G Y S S V S A Y
| m/z calculated | m/z measured | fragment assigned |
| --- | --- | --- |
| 295.14 | 295.42 | c3+ |
| 392.67 | 392.677 | c52+ |
| 1361.61 | 1361.62 | c+17+ |
| 883.41 | 883.44 | c6+ |
| 680.80 | 680.93 | c72+ |
| 762.34 | 762.44 | c82+ |
| 1466.64 | 1466.63 | z+17+ |
| 652.30 | 652.85 | z+162+ |
| 1303.57 | 1303.54 | z+16+ |
| 900.39 | 900.63 | z5+ |
| 813.36 | 813.36 | z4+ |
| 407.69 | 407.43 | z42+ |
| 714.29 | 714.35 | z+13+ |
[M+3H]3+
z+13+
c72+
c82+
z5+
c52+
z+17+
c6+
z42+
c+17+
z+162+
z4+
z+16+
c3+
200
400
600
800
1000
1200
1400
1600
m/z
Supplemental figure 4 M: ETD mass spectrum of [M+3H]3+ ions of glycopeptide GGYSSVSAY modified at Ser395 and Ser398 with glycans Δm315 and Δm390 respectively. RT = 13.04 min.

## Slide 14
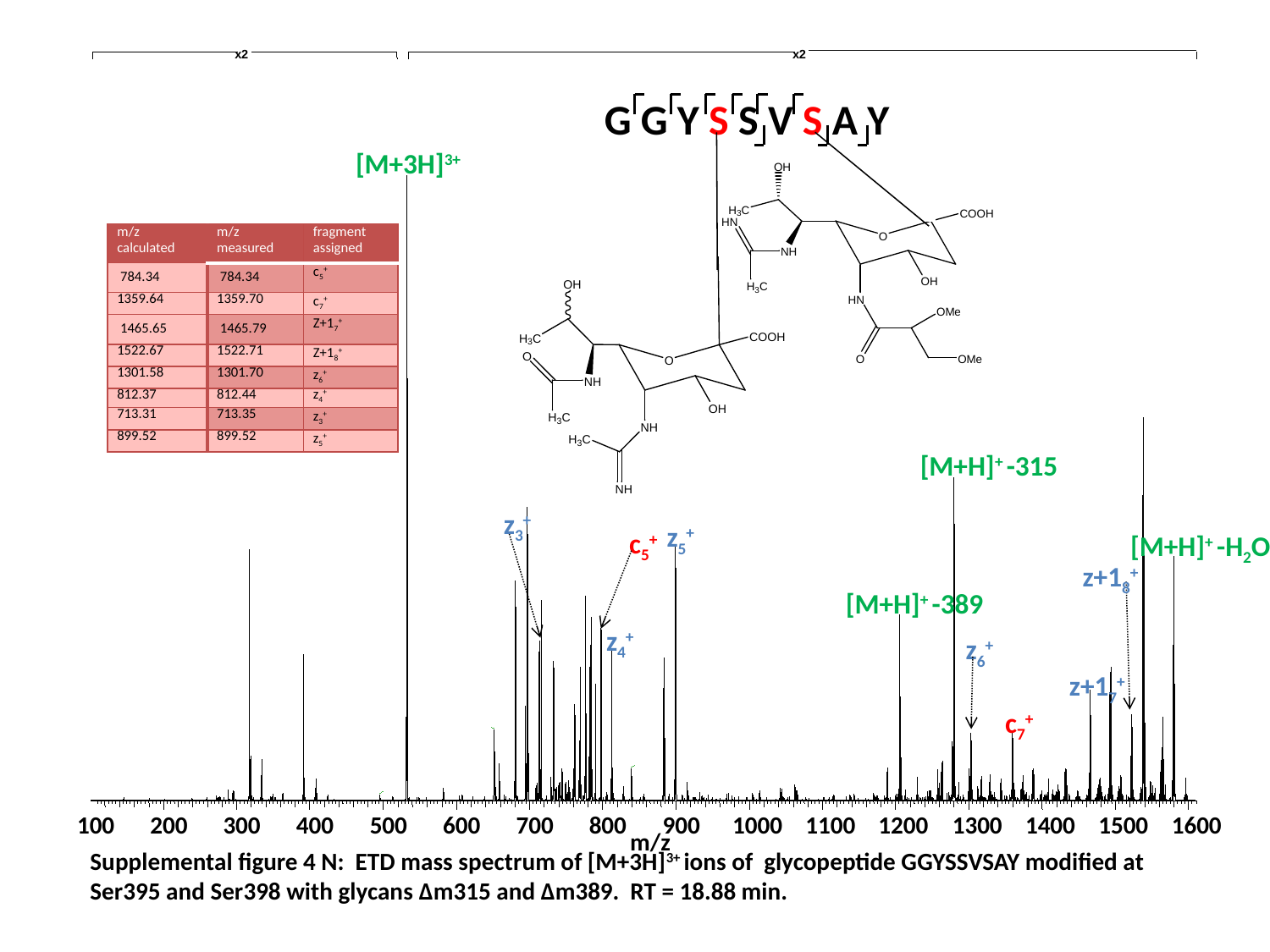

[M+3H]3+
x2
x2
G G Y S S V S A Y
[M+3H]3+
| m/z calculated | m/z measured | fragment assigned |
| --- | --- | --- |
| 784.34 | 784.34 | c5+ |
| 1359.64 | 1359.70 | c7+ |
| 1465.65 | 1465.79 | Z+17+ |
| 1522.67 | 1522.71 | Z+18+ |
| 1301.58 | 1301.70 | z6+ |
| 812.37 | 812.44 | z4+ |
| 713.31 | 713.35 | z3+ |
| 899.52 | 899.52 | z5+ |
[M+H]+ -315
z3+
z5+
c5+
[M+H]+ -H2O
z+18+
[M+H]+ -389
z4+
z6+
z+17+
c7+
100
200
300
400
500
600
700
800
900
1000
1100
1200
1300
1400
1500
1600
m/z
Supplemental figure 4 N: ETD mass spectrum of [M+3H]3+ ions of glycopeptide GGYSSVSAY modified at Ser395 and Ser398 with glycans Δm315 and Δm389. RT = 18.88 min.

## Slide 15
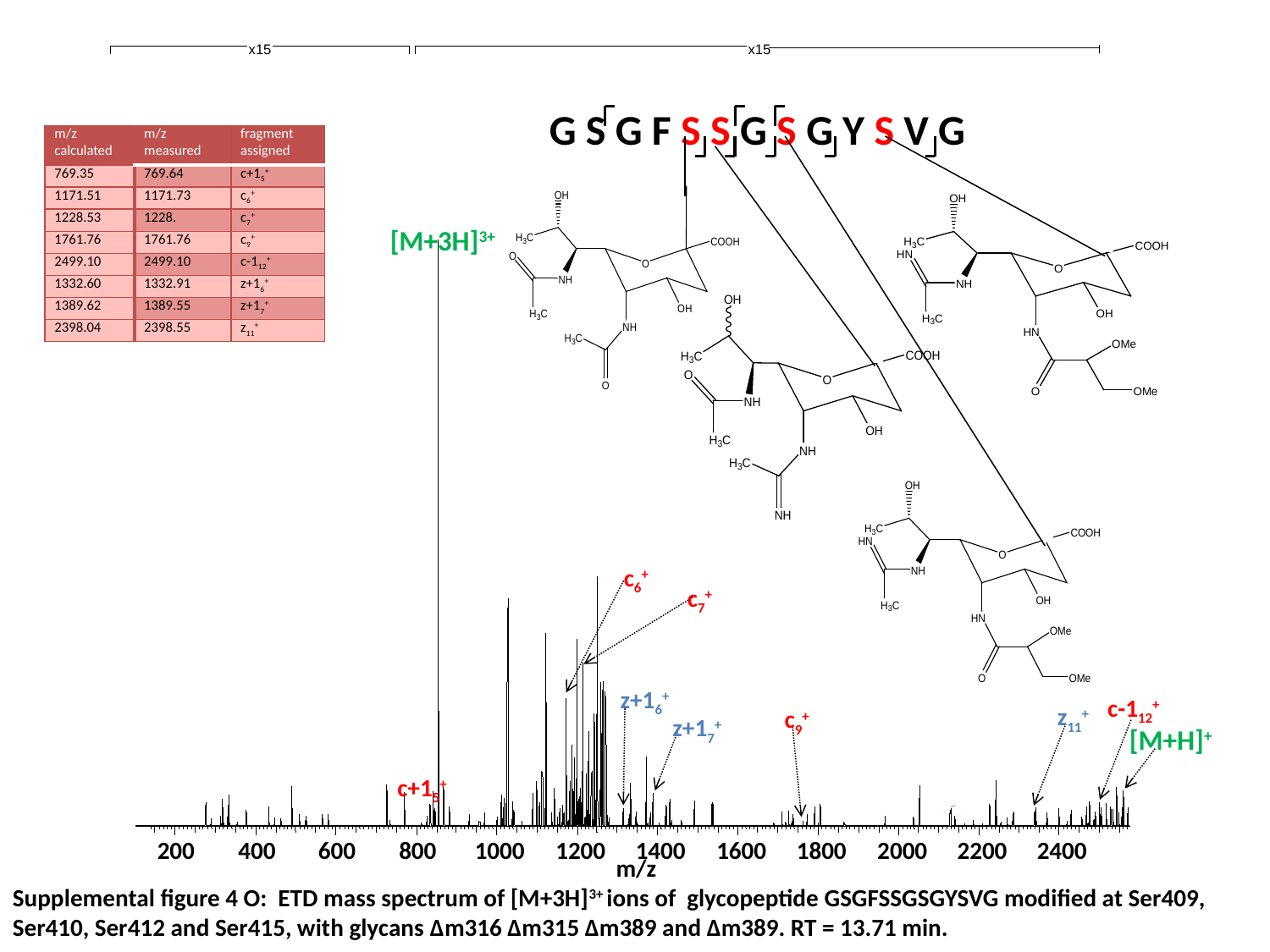

x15
x15
G S G F S S G S G Y S V G
| m/z calculated | m/z measured | fragment assigned |
| --- | --- | --- |
| 769.35 | 769.64 | c+15+ |
| 1171.51 | 1171.73 | c6+ |
| 1228.53 | 1228. | c7+ |
| 1761.76 | 1761.76 | c9+ |
| 2499.10 | 2499.10 | c-112+ |
| 1332.60 | 1332.91 | z+16+ |
| 1389.62 | 1389.55 | z+17+ |
| 2398.04 | 2398.55 | z11+ |
[M+3H]3+
c6+
c7+
z+16+
c-112+
z11+
c9+
z+17+
[M+H]+
c+15+
200
400
600
800
1000
1200
1400
1600
1800
2000
2200
2400
m/z
Supplemental figure 4 O: ETD mass spectrum of [M+3H]3+ ions of glycopeptide GSGFSSGSGYSVG modified at Ser409, Ser410, Ser412 and Ser415, with glycans Δm316 Δm315 Δm389 and Δm389. RT = 13.71 min.

## Slide 16
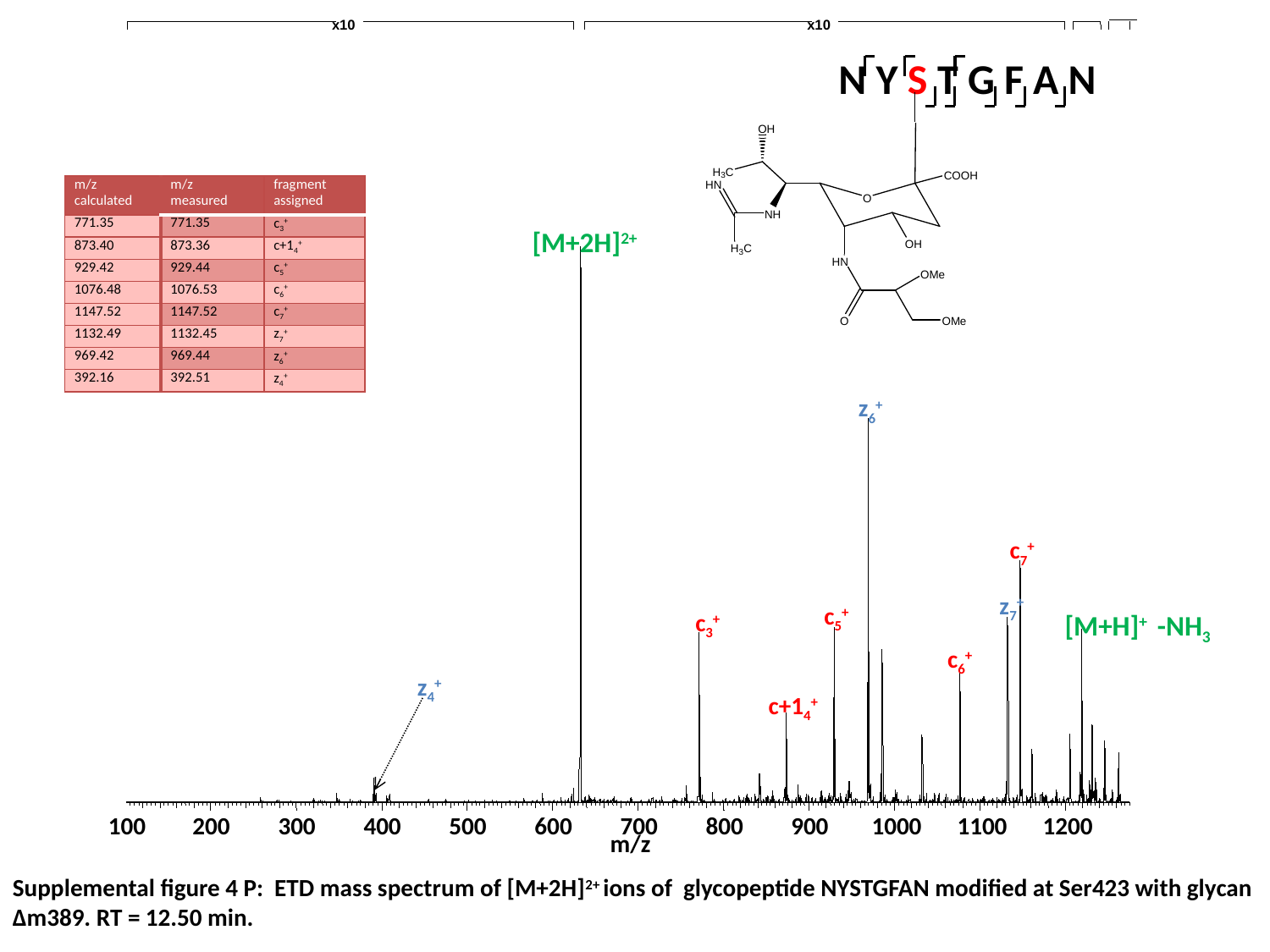

x10
x10
N Y S T G F A N
| m/z calculated | m/z measured | fragment assigned |
| --- | --- | --- |
| 771.35 | 771.35 | c3+ |
| 873.40 | 873.36 | c+14+ |
| 929.42 | 929.44 | c5+ |
| 1076.48 | 1076.53 | c6+ |
| 1147.52 | 1147.52 | c7+ |
| 1132.49 | 1132.45 | z7+ |
| 969.42 | 969.44 | z6+ |
| 392.16 | 392.51 | z4+ |
[M+2H]2+
z6+
c7+
z7+
c5+
c3+
[M+H]+ -NH3
c6+
z4+
c+14+
100
200
300
400
500
600
700
800
900
1000
1100
1200
m/z
Supplemental figure 4 P: ETD mass spectrum of [M+2H]2+ ions of glycopeptide NYSTGFAN modified at Ser423 with glycan Δm389. RT = 12.50 min.

## Slide 17
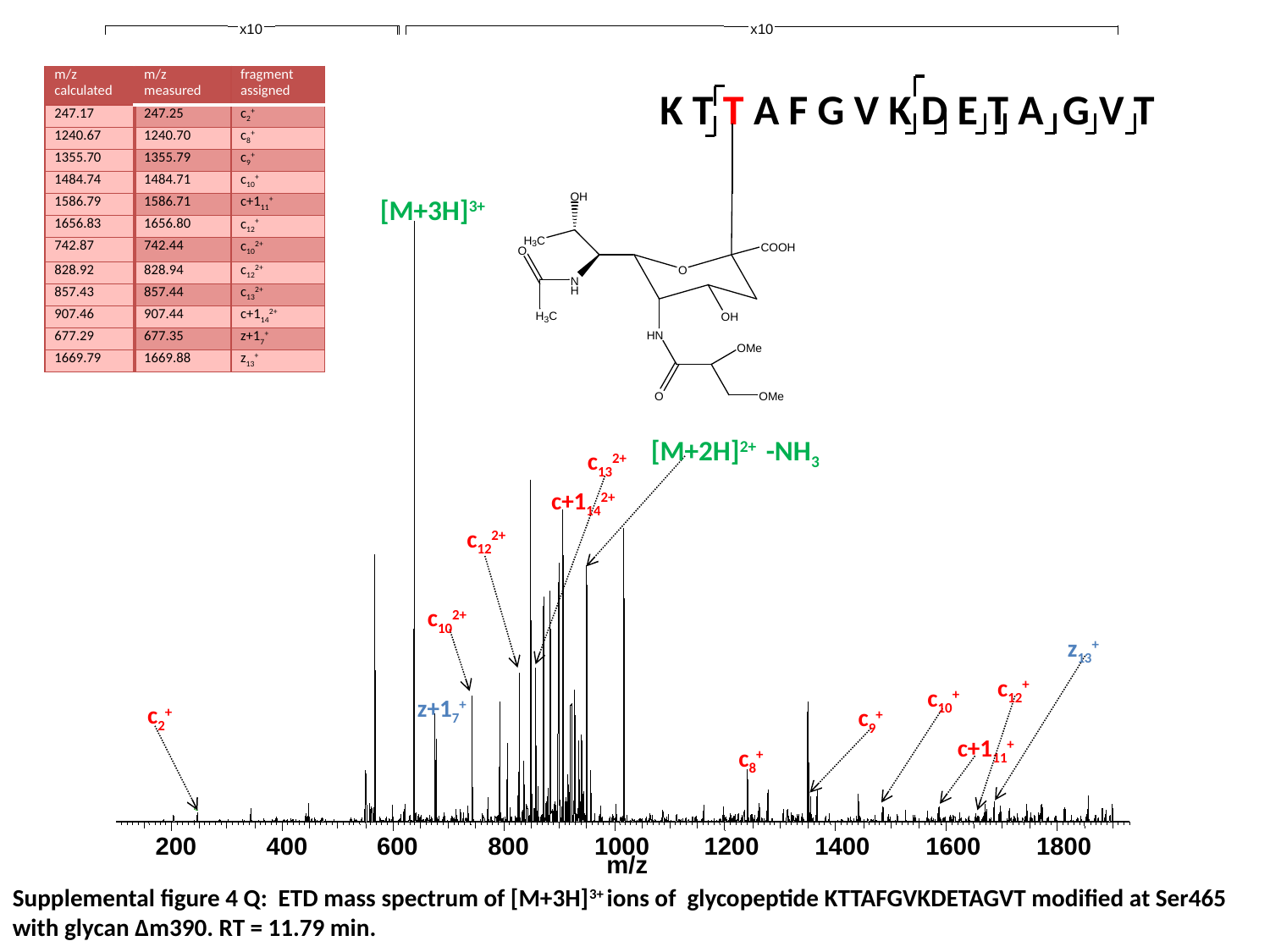

x10
x10
| m/z calculated | m/z measured | fragment assigned |
| --- | --- | --- |
| 247.17 | 247.25 | c2+ |
| 1240.67 | 1240.70 | c8+ |
| 1355.70 | 1355.79 | c9+ |
| 1484.74 | 1484.71 | c10+ |
| 1586.79 | 1586.71 | c+111+ |
| 1656.83 | 1656.80 | c12+ |
| 742.87 | 742.44 | c102+ |
| 828.92 | 828.94 | c122+ |
| 857.43 | 857.44 | c132+ |
| 907.46 | 907.44 | c+1142+ |
| 677.29 | 677.35 | z+17+ |
| 1669.79 | 1669.88 | z13+ |
K T T A F G V K D E T A G V T
[M+3H]3+
[M+2H]2+ -NH3
c132+
c+1142+
c122+
c102+
z13+
c12+
c10+
z+17+
c2+
c9+
c+111+
c8+
1400
1600
1800
200
400
600
800
1000
1200
m/z
Supplemental figure 4 Q: ETD mass spectrum of [M+3H]3+ ions of glycopeptide KTTAFGVKDETAGVT modified at Ser465 with glycan Δm390. RT = 11.79 min.
