# Supplementary material for: Comprehensive mapping of O‐glycosylation in flagellin from Campylobacter jejuni 11168: A multienzyme differential ion mobility mass spectrometry approach
Source: Proteomics. 2015 Jun 15;15(16):2733–45. doi: 10.1002/pmic.201400533 (PMC4975691; doi:10.1002/pmic.201400533)
Supplement: Supplementary file 1 — Figure S1. SDS‐PAGE analysis of purified Campylobacter jejuni flagellin protein. 10% SDS‐PAGE gel, stained with Coomassie blue. Lane 1 – MW markers. Lane 2 – cell suspension from C. jejuni strain 11168 culture, Lane 3 – purified flagellin protein Figure S2. Figure S3. Figure S4. Figure S5. Supplemental Table 1: Non‐glycopeptides identified from tryptic digest of flagellin following ETD MS/MS (with and without FAIMS). (Note that where peptides were identified from both replicates, m/zmeas values are given for replicate#1). Supplemental Table 2: Non‐glycopeptides identified from proteinase K digest of flagellin following ETD MS/MS (without FAIMS). (Note that where peptides were identified from both replicates, m/zmeas values are given for replicate#2). Supplemental Table 3: Non‐glycopeptides identified from proteinase K digest of flagellin following ETD MS/MS (with FAIMS). (Note that where peptides were identified from both replicates, m/zmeas values are given for replicate#2). Comprehensive mapping of O‐glycosylation in flagellin from Campylobacter jejuni 11168: A multi‐enzyme differential ion mobility mass spectrometry approach [file PMIC-15-2733-s001.zip › pmic201400533-sup-0005-figure 5.pptx]

## Slide 1
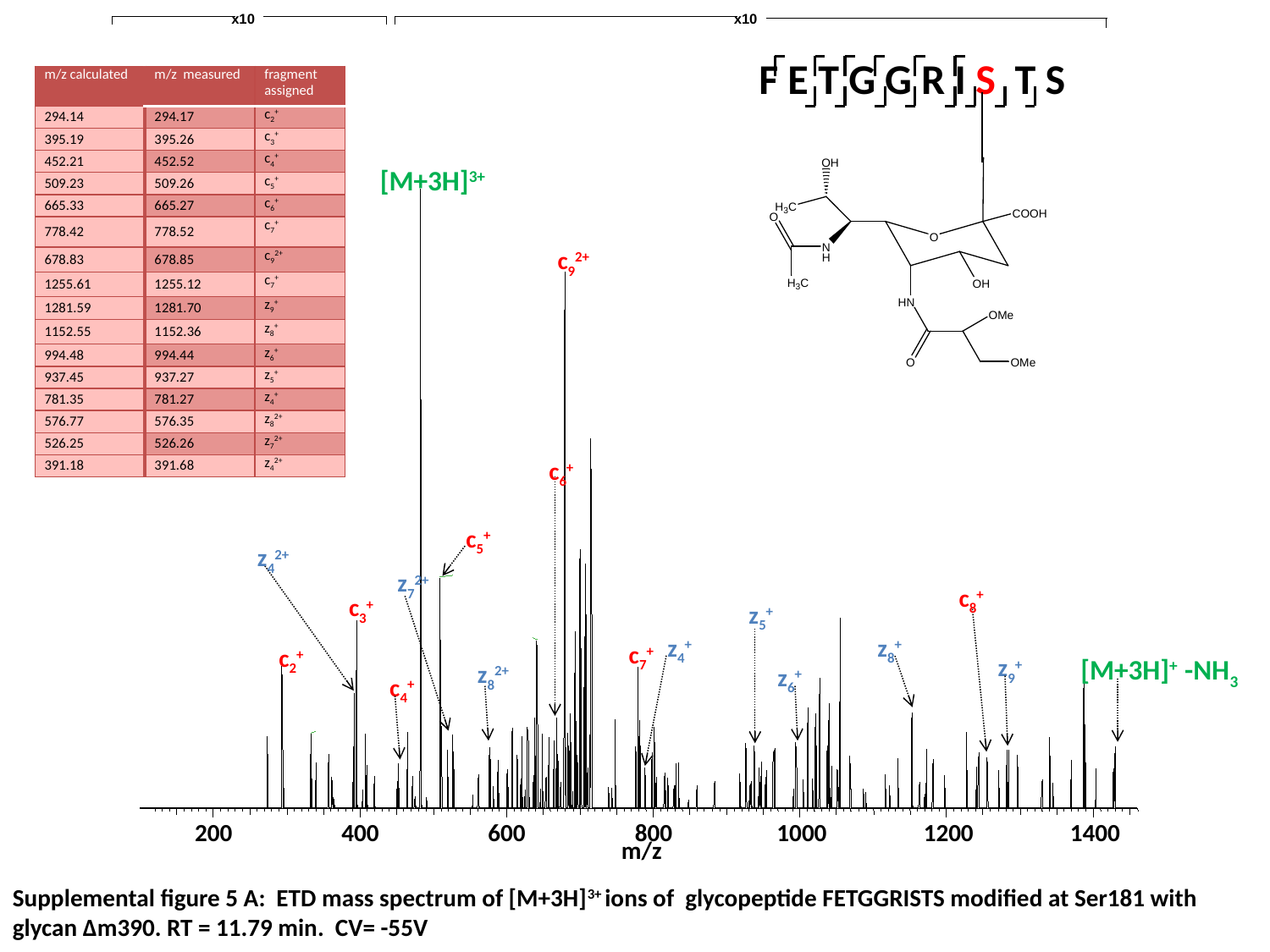

x10
x10
F E T G G R I S T S
| m/z calculated | m/z measured | fragment assigned |
| --- | --- | --- |
| 294.14 | 294.17 | c2+ |
| 395.19 | 395.26 | c3+ |
| 452.21 | 452.52 | c4+ |
| 509.23 | 509.26 | c5+ |
| 665.33 | 665.27 | c6+ |
| 778.42 | 778.52 | c7+ |
| 678.83 | 678.85 | c92+ |
| 1255.61 | 1255.12 | c7+ |
| 1281.59 | 1281.70 | z9+ |
| 1152.55 | 1152.36 | z8+ |
| 994.48 | 994.44 | z6+ |
| 937.45 | 937.27 | z5+ |
| 781.35 | 781.27 | z4+ |
| 576.77 | 576.35 | z82+ |
| 526.25 | 526.26 | z72+ |
| 391.18 | 391.68 | z42+ |
[M+3H]3+
c92+
c6+
c5+
z42+
z72+
c8+
c3+
z5+
z4+
z8+
c7+
c2+
z9+
[M+3H]+ -NH3
z82+
z6+
c4+
200
400
600
800
1000
1200
1400
m/z
Supplemental figure 5 A: ETD mass spectrum of [M+3H]3+ ions of glycopeptide FETGGRISTS modified at Ser181 with glycan Δm390. RT = 11.79 min. CV= -55V

## Slide 2
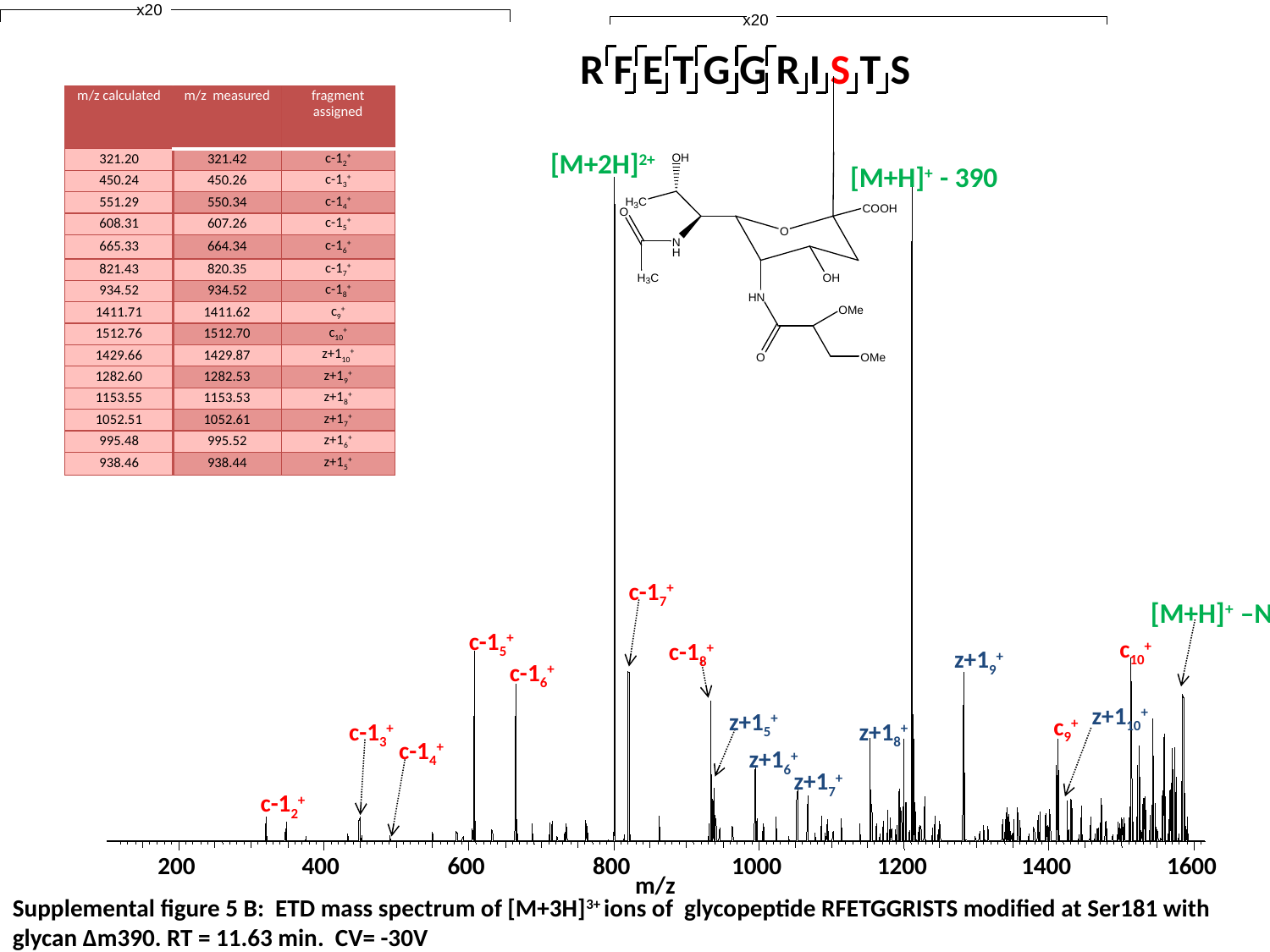

x20
x20
 R F E T G G R I S T S
| m/z calculated | m/z measured | fragment assigned |
| --- | --- | --- |
| 321.20 | 321.42 | c-12+ |
| 450.24 | 450.26 | c-13+ |
| 551.29 | 550.34 | c-14+ |
| 608.31 | 607.26 | c-15+ |
| 665.33 | 664.34 | c-16+ |
| 821.43 | 820.35 | c-17+ |
| 934.52 | 934.52 | c-18+ |
| 1411.71 | 1411.62 | c9+ |
| 1512.76 | 1512.70 | c10+ |
| 1429.66 | 1429.87 | z+110+ |
| 1282.60 | 1282.53 | z+19+ |
| 1153.55 | 1153.53 | z+18+ |
| 1052.51 | 1052.61 | z+17+ |
| 995.48 | 995.52 | z+16+ |
| 938.46 | 938.44 | z+15+ |
[M+2H]2+
[M+H]+ - 390
c-17+
[M+H]+ –NH3
c-15+
c10+
c-18+
z+19+
c-16+
z+110+
z+15+
c9+
c-13+
z+18+
c-14+
z+16+
z+17+
c-12+
200
400
600
800
1000
1200
1400
1600
m/z
Supplemental figure 5 B: ETD mass spectrum of [M+3H]3+ ions of glycopeptide RFETGGRISTS modified at Ser181 with glycan Δm390. RT = 11.63 min. CV= -30V

## Slide 3
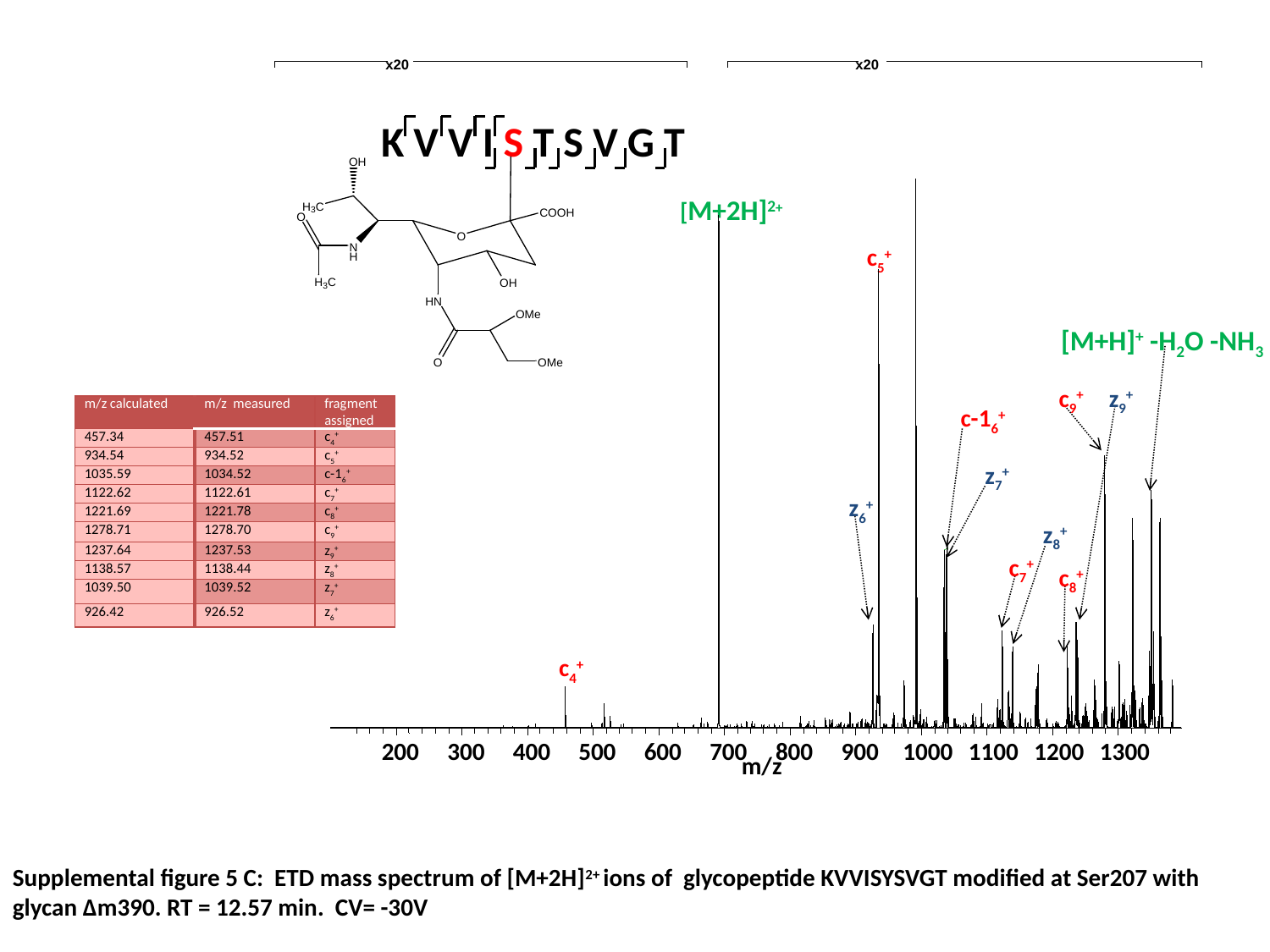

x20
x20
K V V I S T S V G T
[M+2H]2+
c5+
[M+H]+ -H2O -NH3
c9+
z9+
| m/z calculated | m/z measured | fragment assigned |
| --- | --- | --- |
| 457.34 | 457.51 | c4+ |
| 934.54 | 934.52 | c5+ |
| 1035.59 | 1034.52 | c-16+ |
| 1122.62 | 1122.61 | c7+ |
| 1221.69 | 1221.78 | c8+ |
| 1278.71 | 1278.70 | c9+ |
| 1237.64 | 1237.53 | z9+ |
| 1138.57 | 1138.44 | z8+ |
| 1039.50 | 1039.52 | z7+ |
| 926.42 | 926.52 | z6+ |
c-16+
z7+
z6+
z8+
c7+
c8+
c4+
200
300
400
500
600
700
800
900
1000
1100
1200
1300
m/z
Supplemental figure 5 C: ETD mass spectrum of [M+2H]2+ ions of glycopeptide KVVISYSVGT modified at Ser207 with glycan Δm390. RT = 12.57 min. CV= -30V

## Slide 4
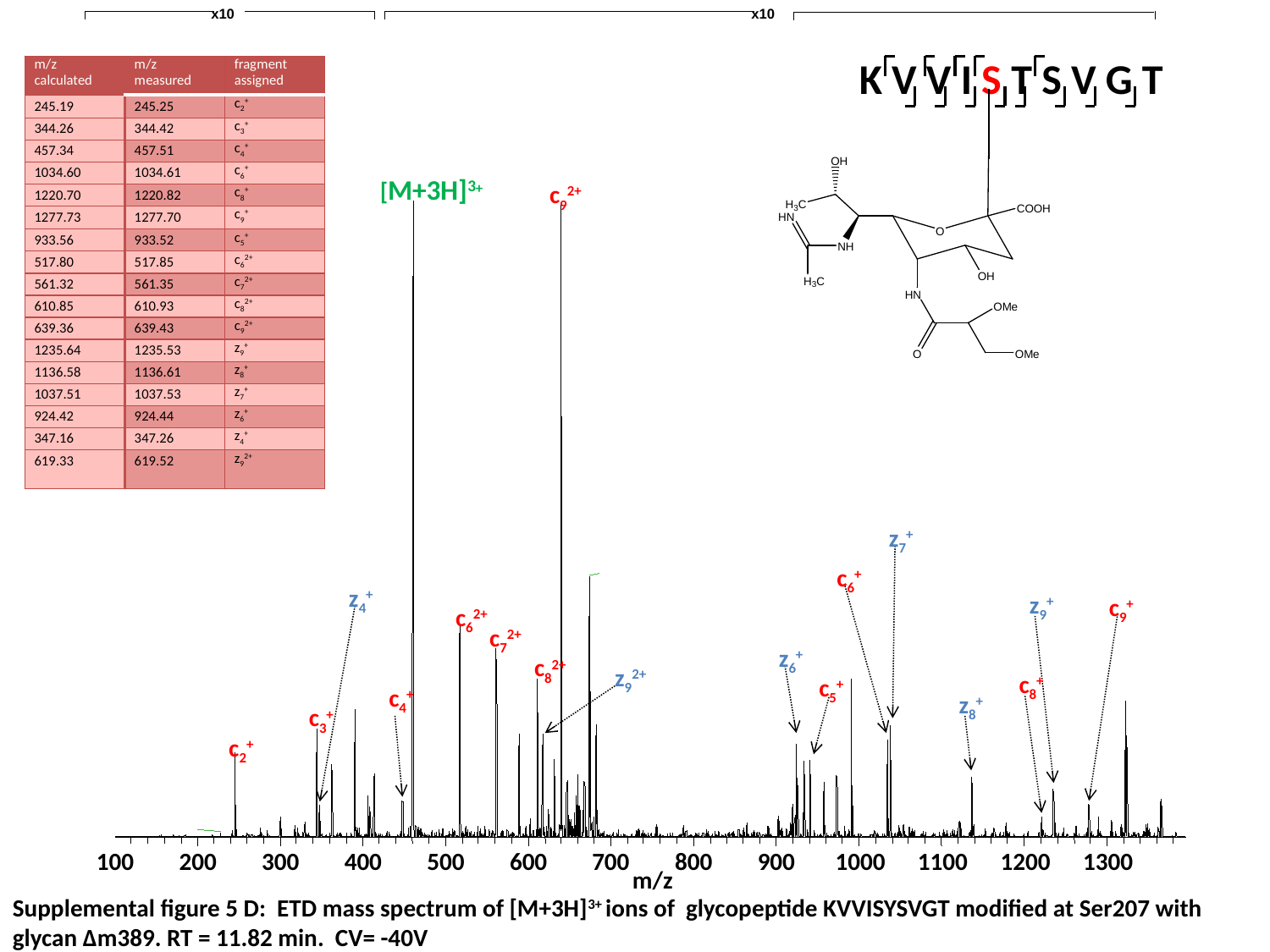

x10
x10
K V V I S T S V G T
| m/z calculated | m/z measured | fragment assigned |
| --- | --- | --- |
| 245.19 | 245.25 | c2+ |
| 344.26 | 344.42 | c3+ |
| 457.34 | 457.51 | c4+ |
| 1034.60 | 1034.61 | c6+ |
| 1220.70 | 1220.82 | c8+ |
| 1277.73 | 1277.70 | c9+ |
| 933.56 | 933.52 | c5+ |
| 517.80 | 517.85 | c62+ |
| 561.32 | 561.35 | c72+ |
| 610.85 | 610.93 | c82+ |
| 639.36 | 639.43 | c92+ |
| 1235.64 | 1235.53 | z9+ |
| 1136.58 | 1136.61 | z8+ |
| 1037.51 | 1037.53 | z7+ |
| 924.42 | 924.44 | z6+ |
| 347.16 | 347.26 | z4+ |
| 619.33 | 619.52 | z92+ |
[M+3H]3+
c92+
z7+
c6+
z4+
z9+
c9+
c62+
c72+
z6+
c82+
z92+
c8+
c5+
c4+
z8+
c3+
c2+
100
200
300
400
500
600
700
800
900
1000
1100
1200
1300
m/z
Supplemental figure 5 D: ETD mass spectrum of [M+3H]3+ ions of glycopeptide KVVISYSVGT modified at Ser207 with glycan Δm389. RT = 11.82 min. CV= -40V

## Slide 5
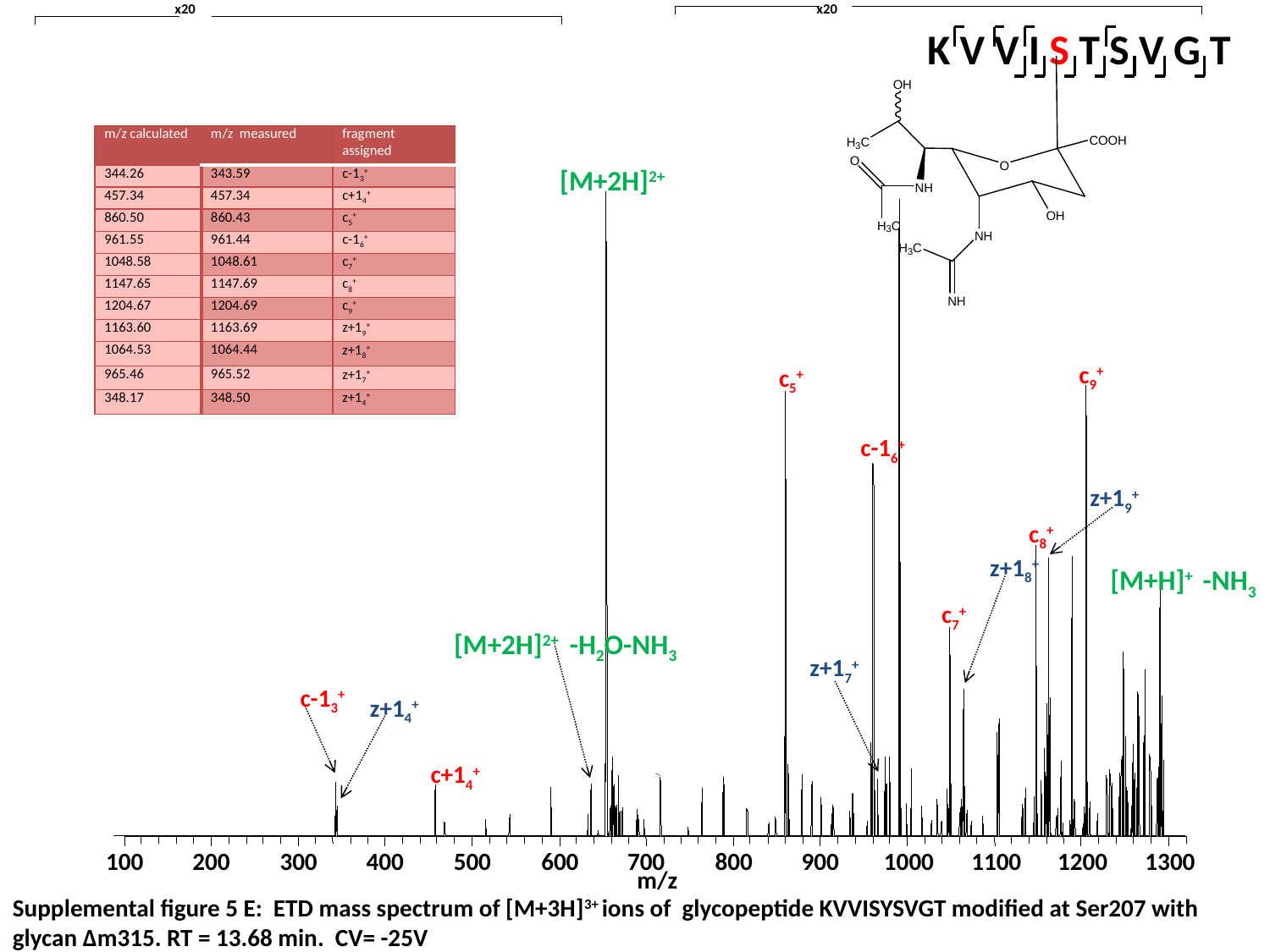

x20
x20
K V V I S T S V G T
| m/z calculated | m/z measured | fragment assigned |
| --- | --- | --- |
| 344.26 | 343.59 | c-13+ |
| 457.34 | 457.34 | c+14+ |
| 860.50 | 860.43 | c5+ |
| 961.55 | 961.44 | c-16+ |
| 1048.58 | 1048.61 | c7+ |
| 1147.65 | 1147.69 | c8+ |
| 1204.67 | 1204.69 | c9+ |
| 1163.60 | 1163.69 | z+19+ |
| 1064.53 | 1064.44 | z+18+ |
| 965.46 | 965.52 | z+17+ |
| 348.17 | 348.50 | z+14+ |
[M+2H]2+
c9+
c5+
c-16+
z+19+
c8+
z+18+
[M+H]+ -NH3
c7+
[M+2H]2+ -H2O-NH3
z+17+
c-13+
z+14+
c+14+
100
200
300
400
500
600
700
800
900
1000
1100
1200
1300
m/z
Supplemental figure 5 E: ETD mass spectrum of [M+3H]3+ ions of glycopeptide KVVISYSVGT modified at Ser207 with glycan Δm315. RT = 13.68 min. CV= -25V

## Slide 6
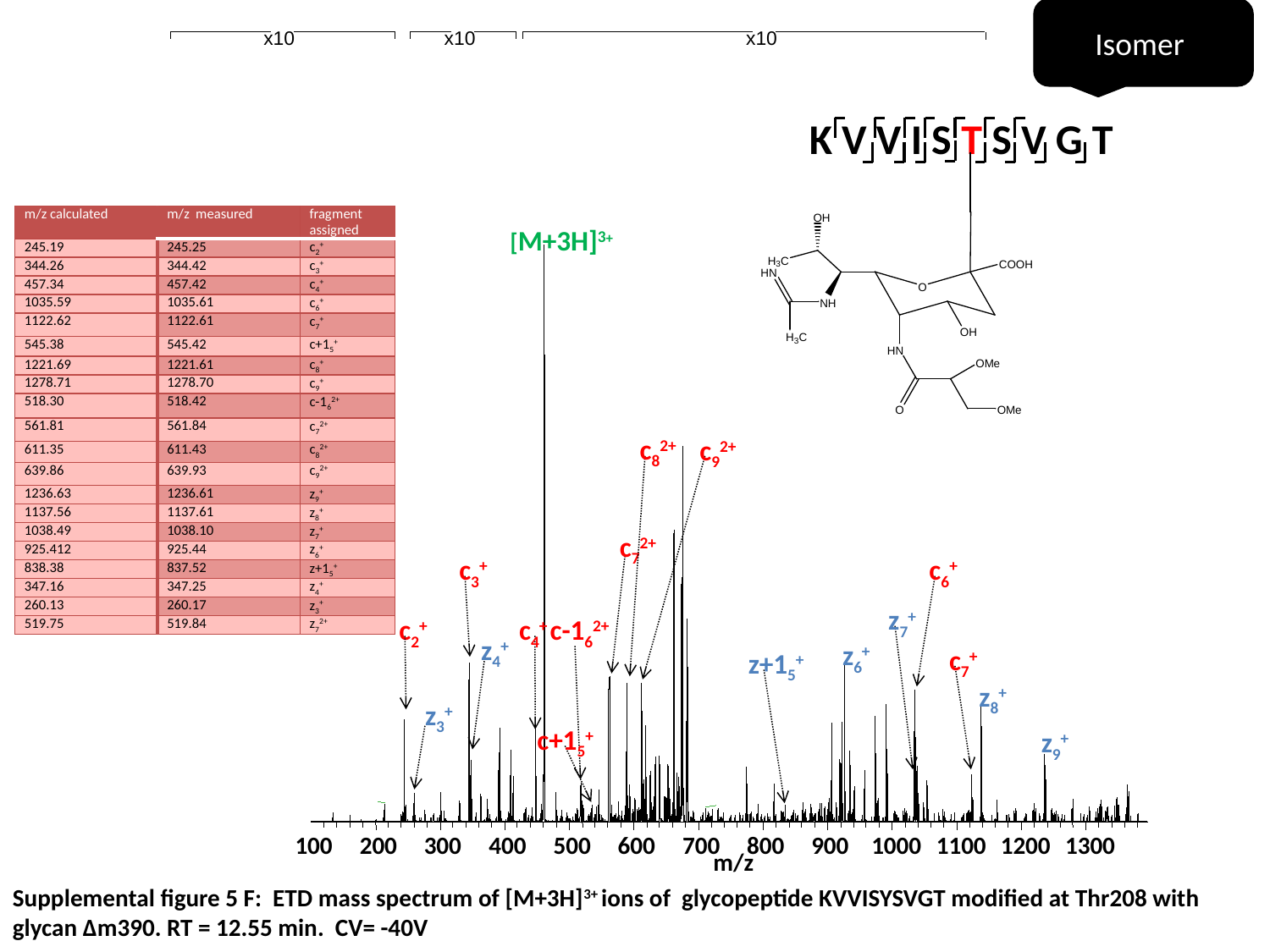

Isomer
x10
x10
x10
K V V I S T S V G T
| m/z calculated | m/z measured | fragment assigned |
| --- | --- | --- |
| 245.19 | 245.25 | c2+ |
| 344.26 | 344.42 | c3+ |
| 457.34 | 457.42 | c4+ |
| 1035.59 | 1035.61 | c6+ |
| 1122.62 | 1122.61 | c7+ |
| 545.38 | 545.42 | c+15+ |
| 1221.69 | 1221.61 | c8+ |
| 1278.71 | 1278.70 | c9+ |
| 518.30 | 518.42 | c-162+ |
| 561.81 | 561.84 | c72+ |
| 611.35 | 611.43 | c82+ |
| 639.86 | 639.93 | c92+ |
| 1236.63 | 1236.61 | z9+ |
| 1137.56 | 1137.61 | z8+ |
| 1038.49 | 1038.10 | z7+ |
| 925.412 | 925.44 | z6+ |
| 838.38 | 837.52 | z+15+ |
| 347.16 | 347.25 | z4+ |
| 260.13 | 260.17 | z3+ |
| 519.75 | 519.84 | z72+ |
[M+3H]3+
c82+
c92+
c72+
c3+
c6+
z7+
c2+
c4+
c-162+
z4+
z6+
c7+
z+15+
z8+
z3+
c+15+
z9+
100
200
300
400
500
600
700
800
900
1000
1100
1200
1300
m/z
Supplemental figure 5 F: ETD mass spectrum of [M+3H]3+ ions of glycopeptide KVVISYSVGT modified at Thr208 with glycan Δm390. RT = 12.55 min. CV= -40V

## Slide 7
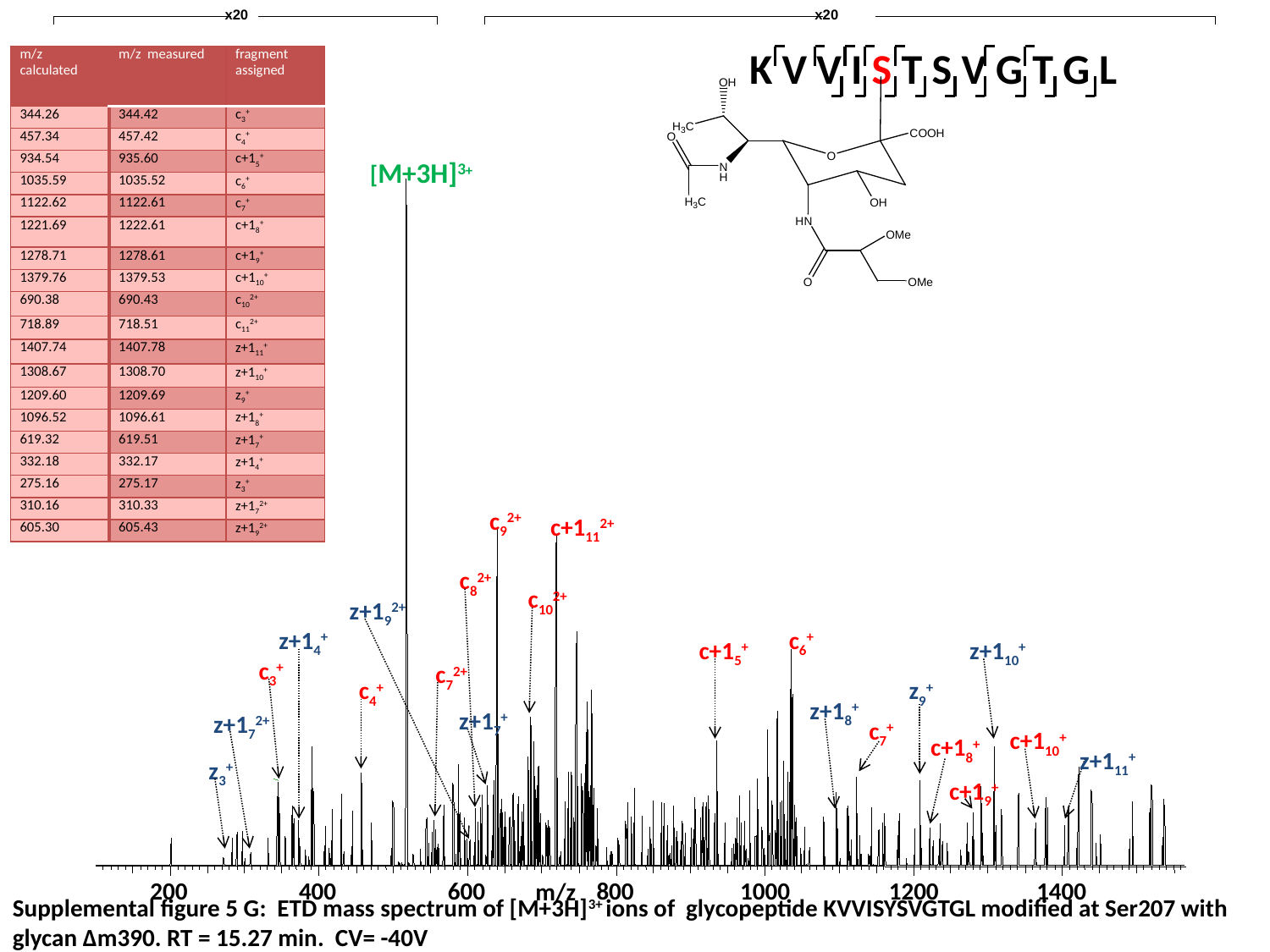

x20
x20
K V V I S T S V G T G L
| m/z calculated | m/z measured | fragment assigned |
| --- | --- | --- |
| 344.26 | 344.42 | c3+ |
| 457.34 | 457.42 | c4+ |
| 934.54 | 935.60 | c+15+ |
| 1035.59 | 1035.52 | c6+ |
| 1122.62 | 1122.61 | c7+ |
| 1221.69 | 1222.61 | c+18+ |
| 1278.71 | 1278.61 | c+19+ |
| 1379.76 | 1379.53 | c+110+ |
| 690.38 | 690.43 | c102+ |
| 718.89 | 718.51 | c112+ |
| 1407.74 | 1407.78 | z+111+ |
| 1308.67 | 1308.70 | z+110+ |
| 1209.60 | 1209.69 | z9+ |
| 1096.52 | 1096.61 | z+18+ |
| 619.32 | 619.51 | z+17+ |
| 332.18 | 332.17 | z+14+ |
| 275.16 | 275.17 | z3+ |
| 310.16 | 310.33 | z+172+ |
| 605.30 | 605.43 | z+192+ |
[M+3H]3+
c92+
c+1112+
c82+
c102+
z+192+
z+14+
c6+
c+15+
z+110+
c3+
c72+
c4+
z9+
z+18+
z+17+
z+172+
c7+
c+110+
c+18+
z+111+
z3+
c+19+
200
400
600
800
1000
1200
1400
m/z
Supplemental figure 5 G: ETD mass spectrum of [M+3H]3+ ions of glycopeptide KVVISYSVGTGL modified at Ser207 with glycan Δm390. RT = 15.27 min. CV= -40V

## Slide 8
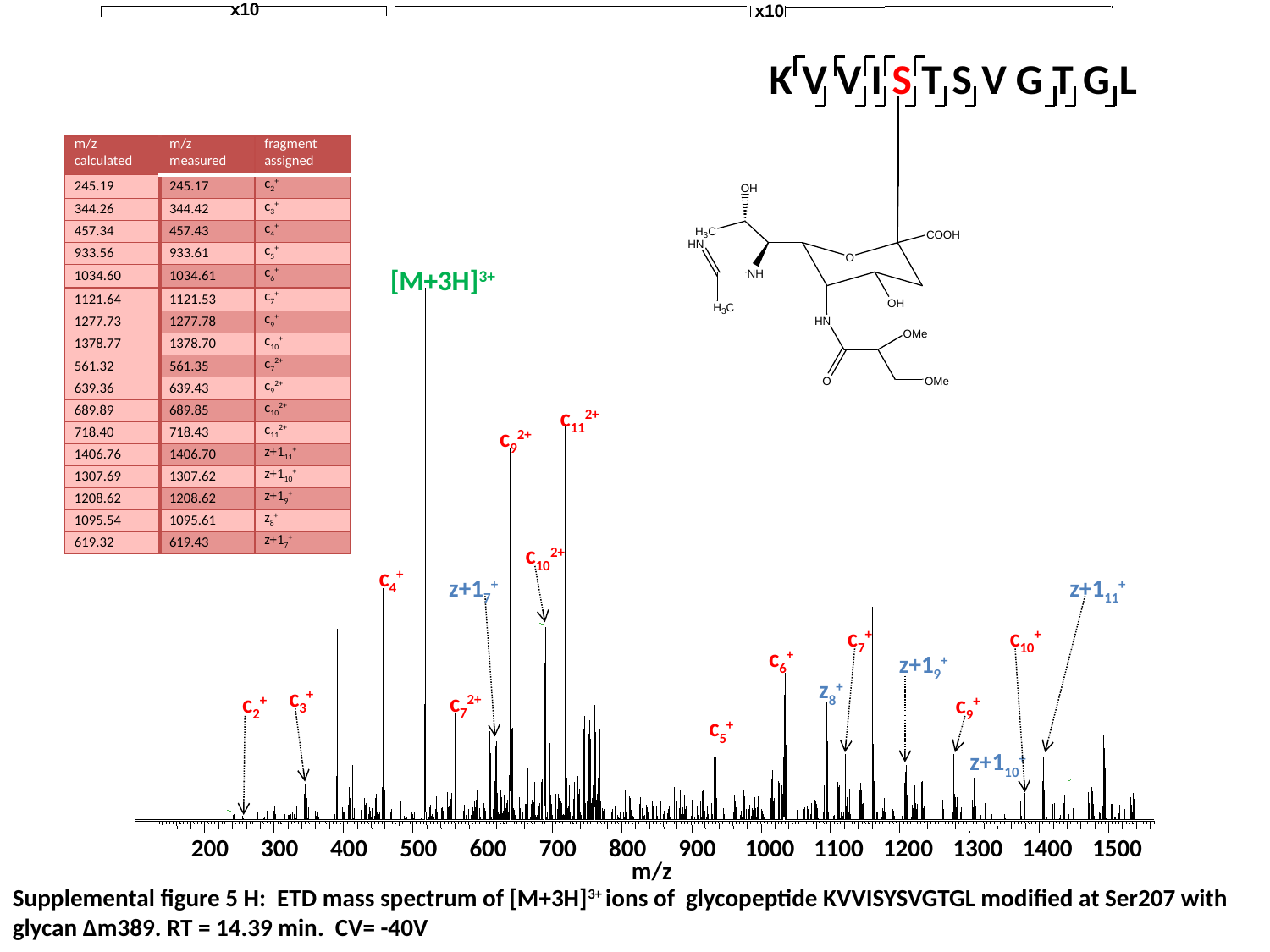

x10
x10
K V V I S T S V G T G L
| m/z calculated | m/z measured | fragment assigned |
| --- | --- | --- |
| 245.19 | 245.17 | c2+ |
| 344.26 | 344.42 | c3+ |
| 457.34 | 457.43 | c4+ |
| 933.56 | 933.61 | c5+ |
| 1034.60 | 1034.61 | c6+ |
| 1121.64 | 1121.53 | c7+ |
| 1277.73 | 1277.78 | c9+ |
| 1378.77 | 1378.70 | c10+ |
| 561.32 | 561.35 | c72+ |
| 639.36 | 639.43 | c92+ |
| 689.89 | 689.85 | c102+ |
| 718.40 | 718.43 | c112+ |
| 1406.76 | 1406.70 | z+111+ |
| 1307.69 | 1307.62 | z+110+ |
| 1208.62 | 1208.62 | z+19+ |
| 1095.54 | 1095.61 | z8+ |
| 619.32 | 619.43 | z+17+ |
[M+3H]3+
c112+
c92+
c102+
c4+
z+17+
z+111+
c7+
c10+
c6+
z+19+
z8+
c3+
c72+
c2+
c9+
c5+
z+110+
200
300
400
500
600
700
800
900
1000
1100
1200
1300
1400
1500
m/z
Supplemental figure 5 H: ETD mass spectrum of [M+3H]3+ ions of glycopeptide KVVISYSVGTGL modified at Ser207 with glycan Δm389. RT = 14.39 min. CV= -40V

## Slide 9
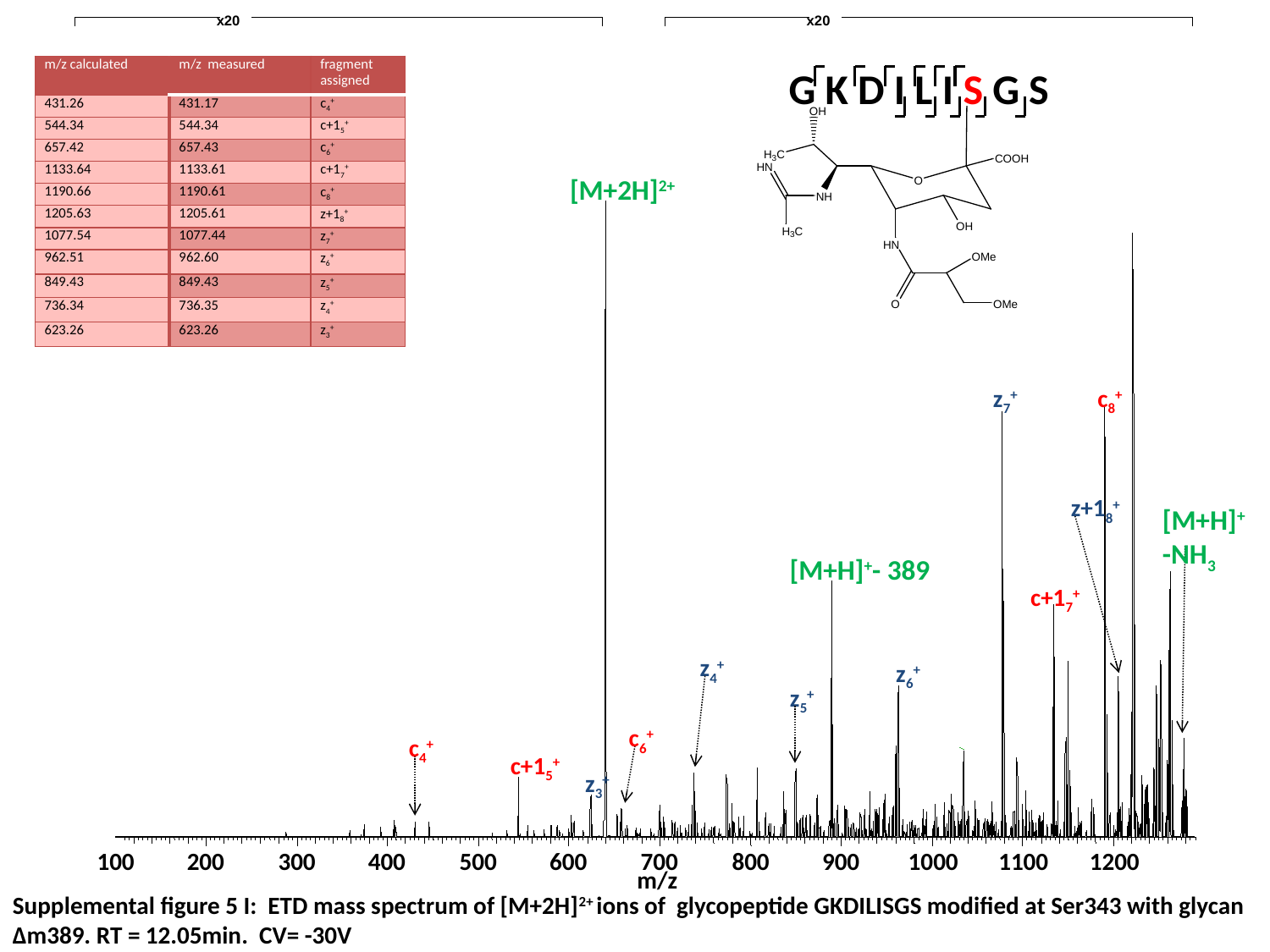

x20
x20
| m/z calculated | m/z measured | fragment assigned |
| --- | --- | --- |
| 431.26 | 431.17 | c4+ |
| 544.34 | 544.34 | c+15+ |
| 657.42 | 657.43 | c6+ |
| 1133.64 | 1133.61 | c+17+ |
| 1190.66 | 1190.61 | c8+ |
| 1205.63 | 1205.61 | z+18+ |
| 1077.54 | 1077.44 | z7+ |
| 962.51 | 962.60 | z6+ |
| 849.43 | 849.43 | z5+ |
| 736.34 | 736.35 | z4+ |
| 623.26 | 623.26 | z3+ |
G K D I L I S G S
[M+2H]2+
z7+
c8+
z+18+
[M+H]+ -NH3
[M+H]+- 389
c+17+
z4+
z6+
z5+
c6+
c4+
c+15+
z3+
100
200
300
400
500
600
700
800
900
1000
1100
1200
m/z
Supplemental figure 5 I: ETD mass spectrum of [M+2H]2+ ions of glycopeptide GKDILISGS modified at Ser343 with glycan Δm389. RT = 12.05min. CV= -30V

## Slide 10
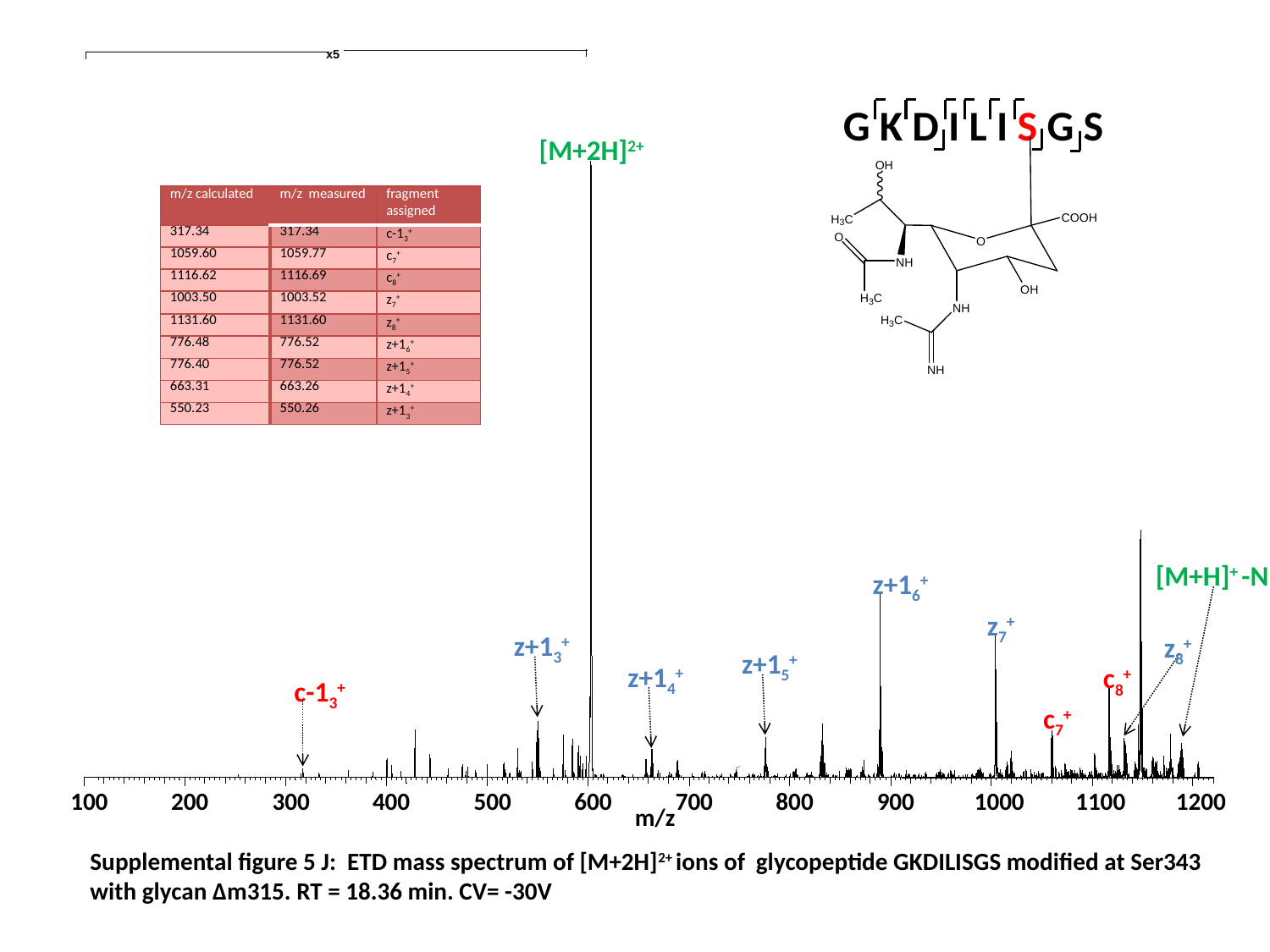

x5
 G K D I L I S G S
[M+2H]2+
| m/z calculated | m/z measured | fragment assigned |
| --- | --- | --- |
| 317.34 | 317.34 | c-13+ |
| 1059.60 | 1059.77 | c7+ |
| 1116.62 | 1116.69 | c8+ |
| 1003.50 | 1003.52 | z7+ |
| 1131.60 | 1131.60 | z8+ |
| 776.48 | 776.52 | z+16+ |
| 776.40 | 776.52 | z+15+ |
| 663.31 | 663.26 | z+14+ |
| 550.23 | 550.26 | z+13+ |
[M+H]+ -NH3
z+16+
z7+
z+13+
z8+
z+15+
z+14+
c8+
c-13+
c7+
100
200
300
400
500
600
700
800
900
1000
1100
1200
m/z
Supplemental figure 5 J: ETD mass spectrum of [M+2H]2+ ions of glycopeptide GKDILISGS modified at Ser343 with glycan Δm315. RT = 18.36 min. CV= -30V

## Slide 11
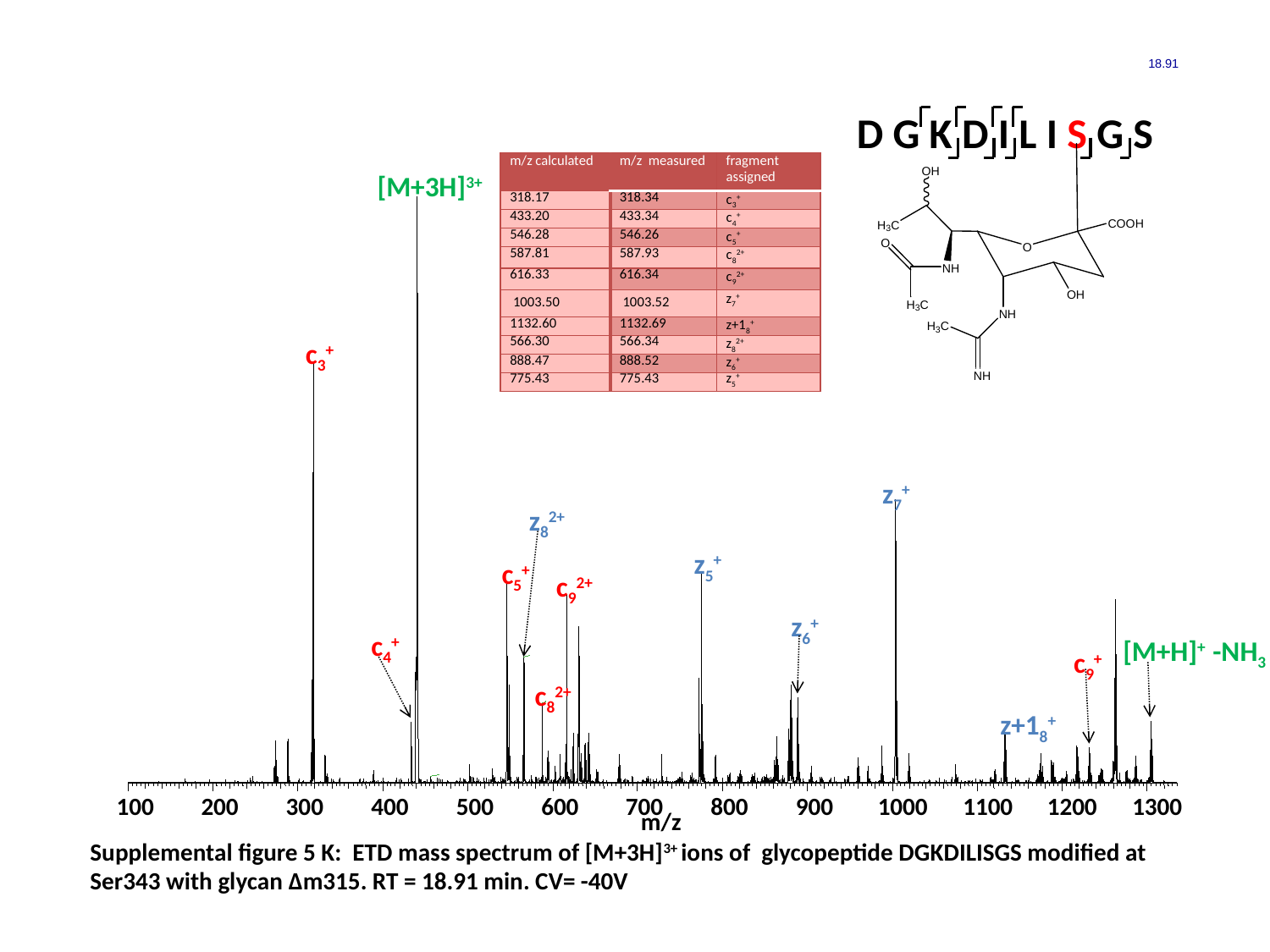

18.91
D G K D I L I S G S
| m/z calculated | m/z measured | fragment assigned |
| --- | --- | --- |
| 318.17 | 318.34 | c3+ |
| 433.20 | 433.34 | c4+ |
| 546.28 | 546.26 | c5+ |
| 587.81 | 587.93 | c82+ |
| 616.33 | 616.34 | c92+ |
| 1003.50 | 1003.52 | z7+ |
| 1132.60 | 1132.69 | z+18+ |
| 566.30 | 566.34 | z82+ |
| 888.47 | 888.52 | z6+ |
| 775.43 | 775.43 | z5+ |
[M+3H]3+
c3+
z7+
z82+
z5+
c5+
c92+
z6+
c4+
[M+H]+ -NH3
c9+
c82+
z+18+
100
200
300
400
500
600
700
800
900
1000
1100
1200
1300
m/z
Supplemental figure 5 K: ETD mass spectrum of [M+3H]3+ ions of glycopeptide DGKDILISGS modified at Ser343 with glycan Δm315. RT = 18.91 min. CV= -40V

## Slide 12
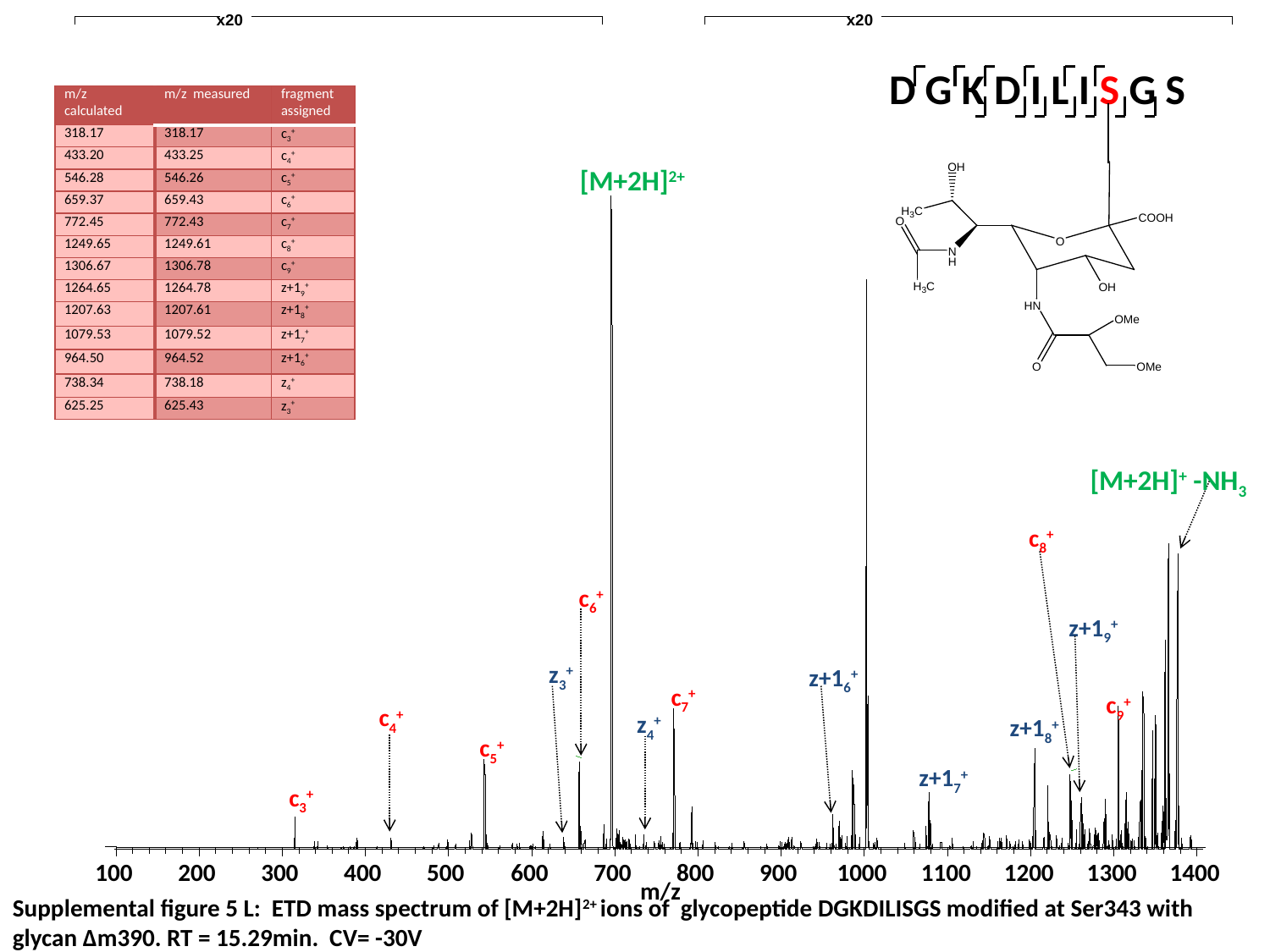

x20
x20
D G K D I L I S G S
| m/z calculated | m/z measured | fragment assigned |
| --- | --- | --- |
| 318.17 | 318.17 | c3+ |
| 433.20 | 433.25 | c4+ |
| 546.28 | 546.26 | c5+ |
| 659.37 | 659.43 | c6+ |
| 772.45 | 772.43 | c7+ |
| 1249.65 | 1249.61 | c8+ |
| 1306.67 | 1306.78 | c9+ |
| 1264.65 | 1264.78 | z+19+ |
| 1207.63 | 1207.61 | z+18+ |
| 1079.53 | 1079.52 | z+17+ |
| 964.50 | 964.52 | z+16+ |
| 738.34 | 738.18 | z4+ |
| 625.25 | 625.43 | z3+ |
[M+2H]2+
[M+2H]+ -NH3
c8+
c6+
z+19+
z3+
z+16+
c7+
c9+
c4+
z4+
z+18+
c5+
z+17+
c3+
100
200
300
400
500
600
700
800
900
1000
1100
1200
1300
1400
m/z
Supplemental figure 5 L: ETD mass spectrum of [M+2H]2+ ions of glycopeptide DGKDILISGS modified at Ser343 with glycan Δm390. RT = 15.29min. CV= -30V

## Slide 13
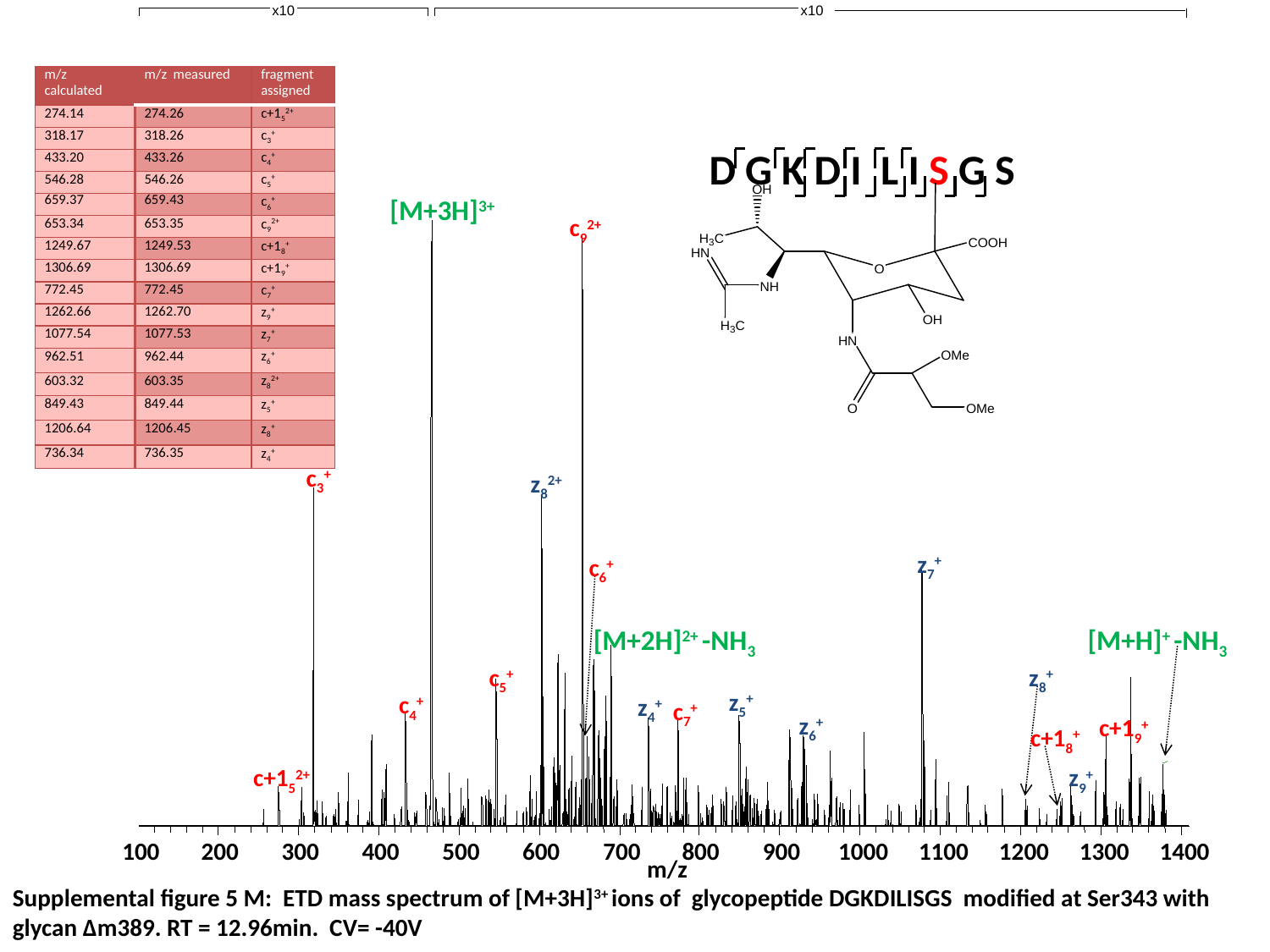

x10
x10
| m/z calculated | m/z measured | fragment assigned |
| --- | --- | --- |
| 274.14 | 274.26 | c+152+ |
| 318.17 | 318.26 | c3+ |
| 433.20 | 433.26 | c4+ |
| 546.28 | 546.26 | c5+ |
| 659.37 | 659.43 | c6+ |
| 653.34 | 653.35 | c92+ |
| 1249.67 | 1249.53 | c+18+ |
| 1306.69 | 1306.69 | c+19+ |
| 772.45 | 772.45 | c7+ |
| 1262.66 | 1262.70 | z9+ |
| 1077.54 | 1077.53 | z7+ |
| 962.51 | 962.44 | z6+ |
| 603.32 | 603.35 | z82+ |
| 849.43 | 849.44 | z5+ |
| 1206.64 | 1206.45 | z8+ |
| 736.34 | 736.35 | z4+ |
D G K D I L I S G S
[M+3H]3+
c92+
c3+
z82+
z7+
c6+
[M+2H]2+ -NH3
[M+H]+ -NH3
c5+
z8+
z5+
c4+
z4+
c7+
z6+
c+19+
c+18+
c+152+
z9+
100
200
300
400
500
600
700
800
900
1000
1100
1200
1300
1400
m/z
Supplemental figure 5 M: ETD mass spectrum of [M+3H]3+ ions of glycopeptide DGKDILISGS modified at Ser343 with glycan Δm389. RT = 12.96min. CV= -40V

## Slide 14
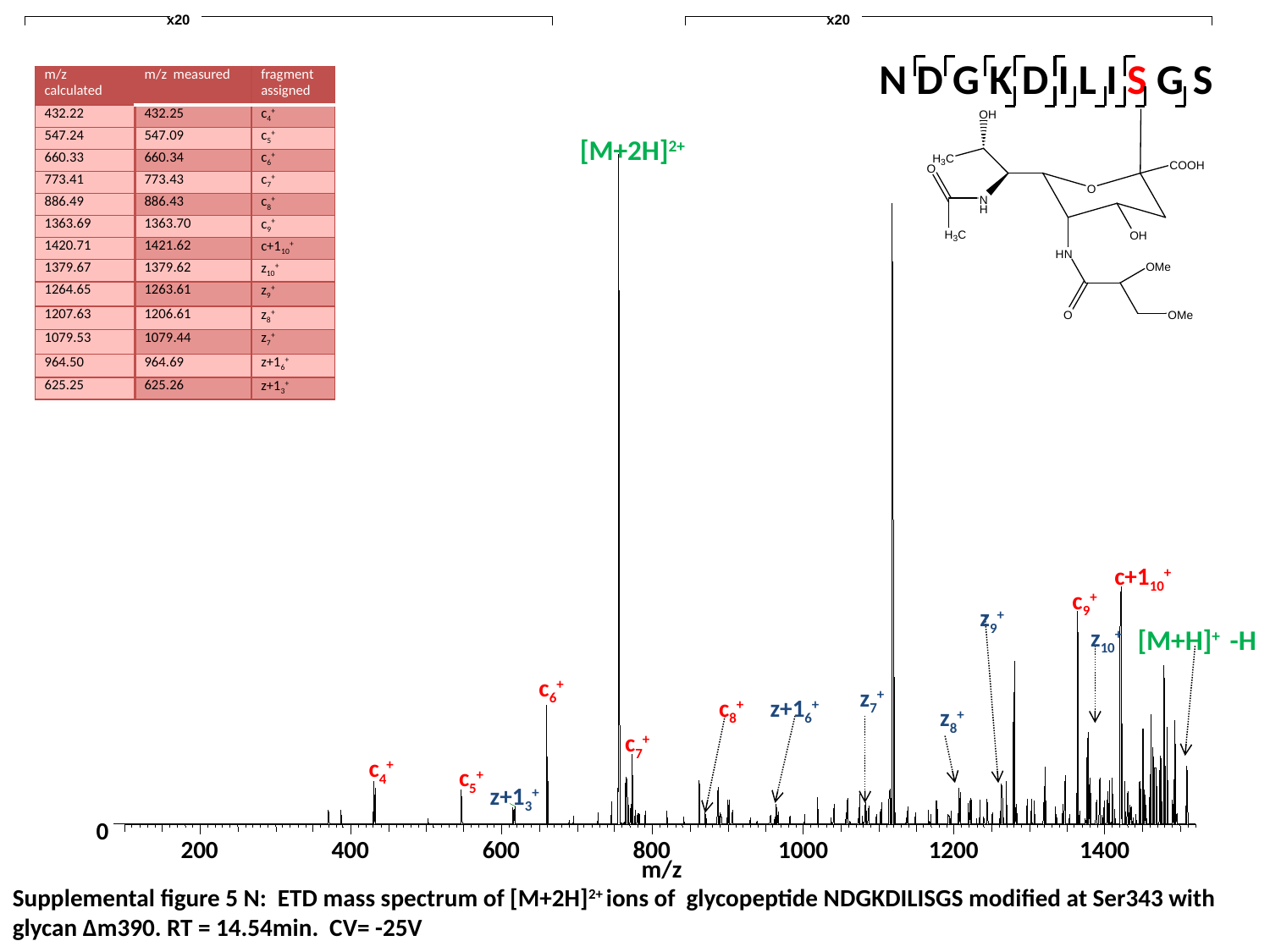

x20
x20
N D G K D I L I S G S
| m/z calculated | m/z measured | fragment assigned |
| --- | --- | --- |
| 432.22 | 432.25 | c4+ |
| 547.24 | 547.09 | c5+ |
| 660.33 | 660.34 | c6+ |
| 773.41 | 773.43 | c7+ |
| 886.49 | 886.43 | c8+ |
| 1363.69 | 1363.70 | c9+ |
| 1420.71 | 1421.62 | c+110+ |
| 1379.67 | 1379.62 | z10+ |
| 1264.65 | 1263.61 | z9+ |
| 1207.63 | 1206.61 | z8+ |
| 1079.53 | 1079.44 | z7+ |
| 964.50 | 964.69 | z+16+ |
| 625.25 | 625.26 | z+13+ |
[M+2H]2+
c+110+
c9+
z9+
z10+
[M+H]+ -H
c6+
z7+
c8+
z+16+
z8+
c7+
c4+
c5+
z+13+
0
200
400
600
800
1000
1200
1400
m/z
Supplemental figure 5 N: ETD mass spectrum of [M+2H]2+ ions of glycopeptide NDGKDILISGS modified at Ser343 with glycan Δm390. RT = 14.54min. CV= -25V

## Slide 15
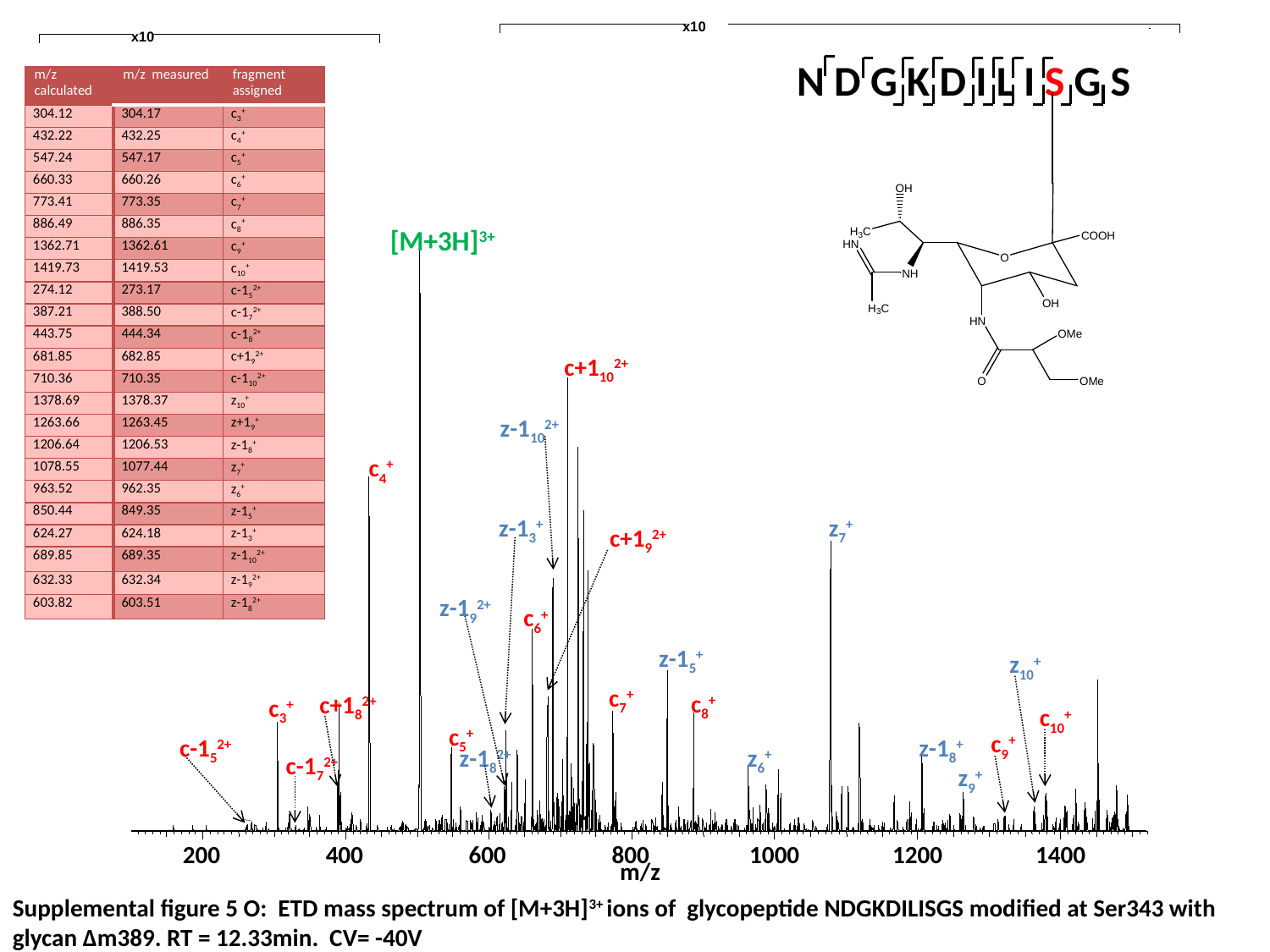

x10
x10
 N D G K D I L I S G S
| m/z calculated | m/z measured | fragment assigned |
| --- | --- | --- |
| 304.12 | 304.17 | c3+ |
| 432.22 | 432.25 | c4+ |
| 547.24 | 547.17 | c5+ |
| 660.33 | 660.26 | c6+ |
| 773.41 | 773.35 | c7+ |
| 886.49 | 886.35 | c8+ |
| 1362.71 | 1362.61 | c9+ |
| 1419.73 | 1419.53 | c10+ |
| 274.12 | 273.17 | c-152+ |
| 387.21 | 388.50 | c-172+ |
| 443.75 | 444.34 | c-182+ |
| 681.85 | 682.85 | c+192+ |
| 710.36 | 710.35 | c-1102+ |
| 1378.69 | 1378.37 | z10+ |
| 1263.66 | 1263.45 | z+19+ |
| 1206.64 | 1206.53 | z-18+ |
| 1078.55 | 1077.44 | z7+ |
| 963.52 | 962.35 | z6+ |
| 850.44 | 849.35 | z-15+ |
| 624.27 | 624.18 | z-13+ |
| 689.85 | 689.35 | z-1102+ |
| 632.33 | 632.34 | z-192+ |
| 603.82 | 603.51 | z-182+ |
[M+3H]3+
c+1102+
z-1102+
c4+
z-13+
z7+
c+192+
z-192+
c6+
z-15+
z10+
c7+
c8+
c+182+
c3+
c10+
c5+
c9+
c-152+
z-18+
z-182+
z6+
c-172+
z9+
200
400
600
800
1000
1200
1400
m/z
Supplemental figure 5 O: ETD mass spectrum of [M+3H]3+ ions of glycopeptide NDGKDILISGS modified at Ser343 with glycan Δm389. RT = 12.33min. CV= -40V

## Slide 16
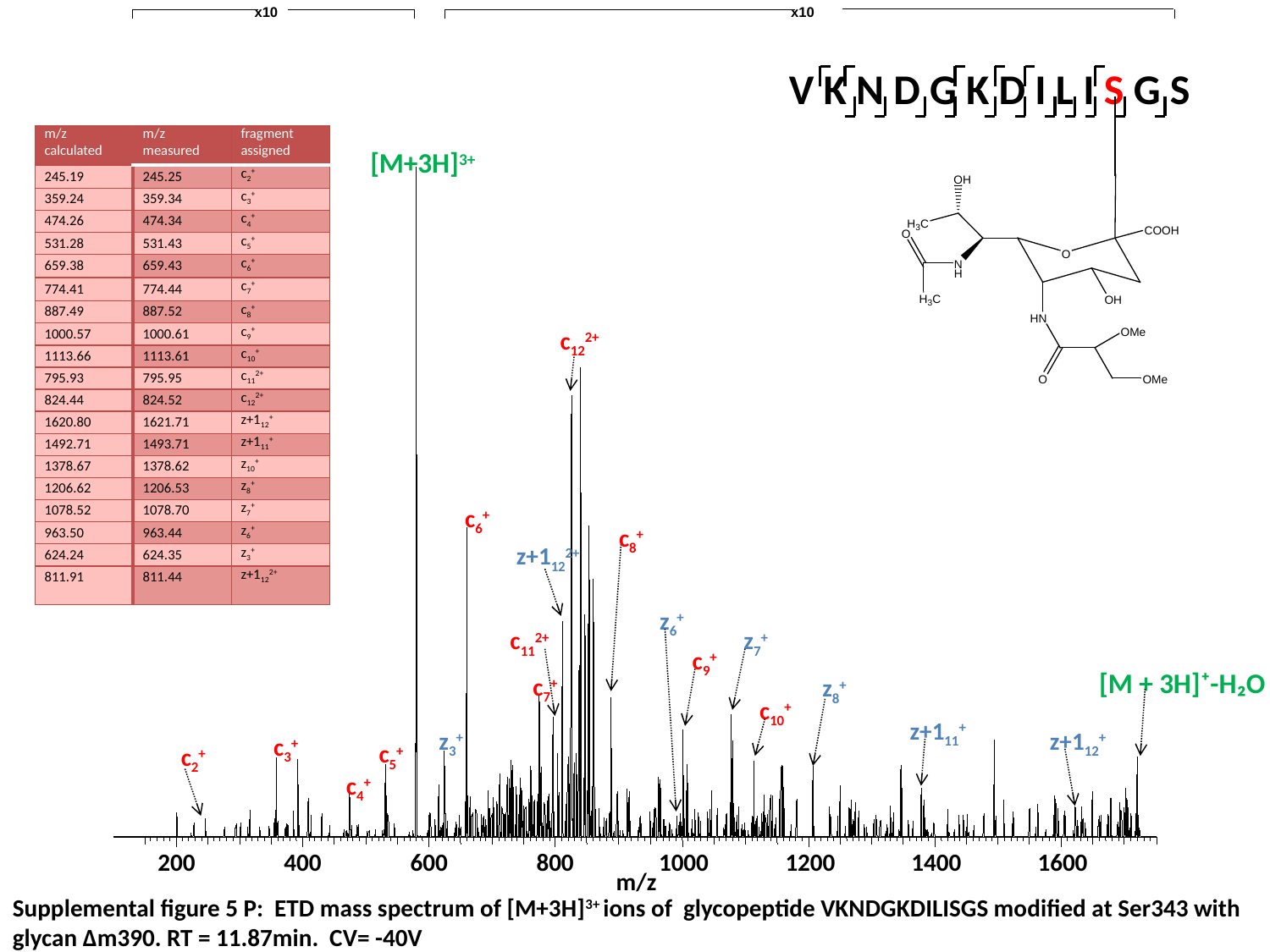

x10
x10
 V K N D G K D I L I S G S
| m/z calculated | m/z measured | fragment assigned |
| --- | --- | --- |
| 245.19 | 245.25 | c2+ |
| 359.24 | 359.34 | c3+ |
| 474.26 | 474.34 | c4+ |
| 531.28 | 531.43 | c5+ |
| 659.38 | 659.43 | c6+ |
| 774.41 | 774.44 | c7+ |
| 887.49 | 887.52 | c8+ |
| 1000.57 | 1000.61 | c9+ |
| 1113.66 | 1113.61 | c10+ |
| 795.93 | 795.95 | c112+ |
| 824.44 | 824.52 | c122+ |
| 1620.80 | 1621.71 | z+112+ |
| 1492.71 | 1493.71 | z+111+ |
| 1378.67 | 1378.62 | z10+ |
| 1206.62 | 1206.53 | z8+ |
| 1078.52 | 1078.70 | z7+ |
| 963.50 | 963.44 | z6+ |
| 624.24 | 624.35 | z3+ |
| 811.91 | 811.44 | z+1122+ |
[M+3H]3+
c122+
c6+
c8+
z+1122+
z6+
c112+
z7+
c9+
[M + 3H]⁺-H₂O
c7+
z8+
c10+
z+111+
z3+
z+112+
c3+
c5+
c2+
c4+
200
400
600
800
1000
1200
1400
1600
m/z
Supplemental figure 5 P: ETD mass spectrum of [M+3H]3+ ions of glycopeptide VKNDGKDILISGS modified at Ser343 with glycan Δm390. RT = 11.87min. CV= -40V

## Slide 17
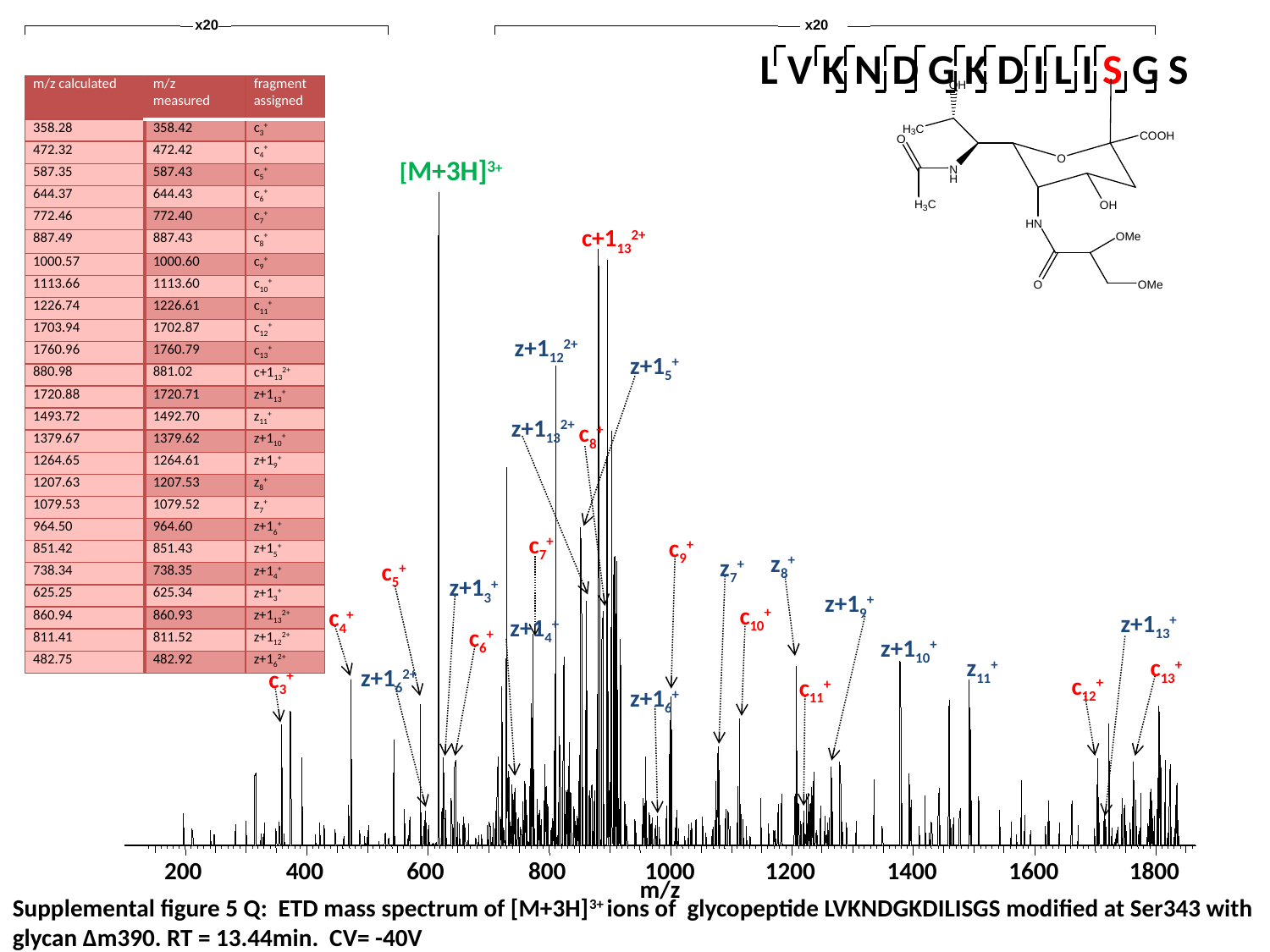

x20
x20
L V K N D G K D I L I S G S
| m/z calculated | m/z measured | fragment assigned |
| --- | --- | --- |
| 358.28 | 358.42 | c3+ |
| 472.32 | 472.42 | c4+ |
| 587.35 | 587.43 | c5+ |
| 644.37 | 644.43 | c6+ |
| 772.46 | 772.40 | c7+ |
| 887.49 | 887.43 | c8+ |
| 1000.57 | 1000.60 | c9+ |
| 1113.66 | 1113.60 | c10+ |
| 1226.74 | 1226.61 | c11+ |
| 1703.94 | 1702.87 | c12+ |
| 1760.96 | 1760.79 | c13+ |
| 880.98 | 881.02 | c+1132+ |
| 1720.88 | 1720.71 | z+113+ |
| 1493.72 | 1492.70 | z11+ |
| 1379.67 | 1379.62 | z+110+ |
| 1264.65 | 1264.61 | z+19+ |
| 1207.63 | 1207.53 | z8+ |
| 1079.53 | 1079.52 | z7+ |
| 964.50 | 964.60 | z+16+ |
| 851.42 | 851.43 | z+15+ |
| 738.34 | 738.35 | z+14+ |
| 625.25 | 625.34 | z+13+ |
| 860.94 | 860.93 | z+1132+ |
| 811.41 | 811.52 | z+1122+ |
| 482.75 | 482.92 | z+162+ |
[M+3H]3+
c+1132+
z+1122+
z+15+
z+1132+
c8+
c7+
c9+
z8+
z7+
c5+
z+13+
z+19+
c10+
c4+
z+113+
z+14+
c6+
z+110+
z11+
c13+
z+162+
c3+
c12+
c11+
z+16+
200
400
600
800
1000
1200
1400
1600
1800
m/z
Supplemental figure 5 Q: ETD mass spectrum of [M+3H]3+ ions of glycopeptide LVKNDGKDILISGS modified at Ser343 with glycan Δm390. RT = 13.44min. CV= -40V

## Slide 18
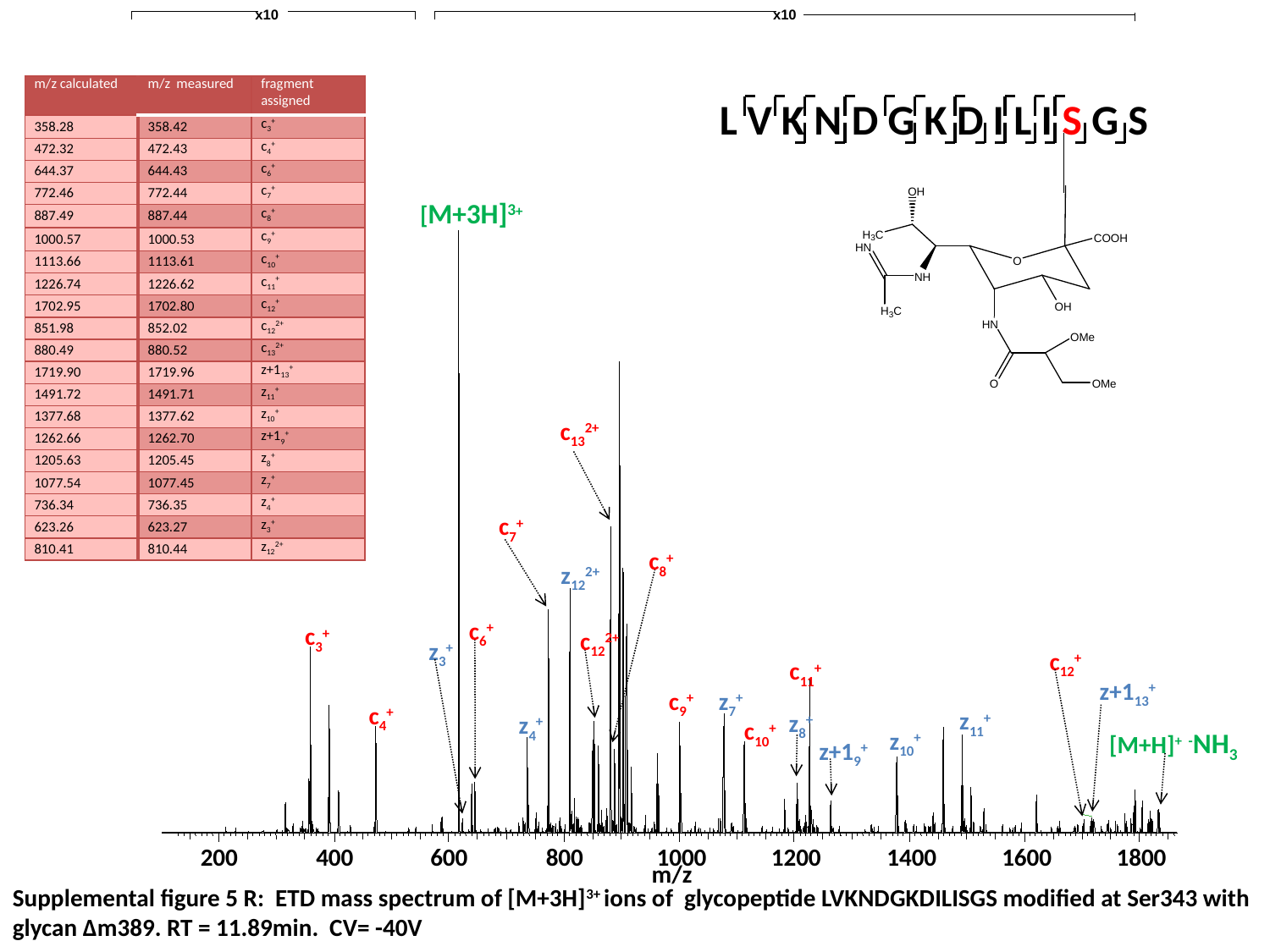

x10
x10
| m/z calculated | m/z measured | fragment assigned |
| --- | --- | --- |
| 358.28 | 358.42 | c3+ |
| 472.32 | 472.43 | c4+ |
| 644.37 | 644.43 | c6+ |
| 772.46 | 772.44 | c7+ |
| 887.49 | 887.44 | c8+ |
| 1000.57 | 1000.53 | c9+ |
| 1113.66 | 1113.61 | c10+ |
| 1226.74 | 1226.62 | c11+ |
| 1702.95 | 1702.80 | c12+ |
| 851.98 | 852.02 | c122+ |
| 880.49 | 880.52 | c132+ |
| 1719.90 | 1719.96 | z+113+ |
| 1491.72 | 1491.71 | z11+ |
| 1377.68 | 1377.62 | z10+ |
| 1262.66 | 1262.70 | z+19+ |
| 1205.63 | 1205.45 | z8+ |
| 1077.54 | 1077.45 | z7+ |
| 736.34 | 736.35 | z4+ |
| 623.26 | 623.27 | z3+ |
| 810.41 | 810.44 | z122+ |
L V K N D G K D I L I S G S
[M+3H]3+
c132+
c7+
c8+
z122+
c6+
c3+
c122+
z3+
c12+
c11+
z+113+
c9+
z7+
c4+
z11+
z8+
z4+
c10+
z10+
[M+H]+ -NH3
z+19+
200
400
600
800
1000
1200
1400
1600
1800
m/z
Supplemental figure 5 R: ETD mass spectrum of [M+3H]3+ ions of glycopeptide LVKNDGKDILISGS modified at Ser343 with glycan Δm389. RT = 11.89min. CV= -40V

## Slide 19
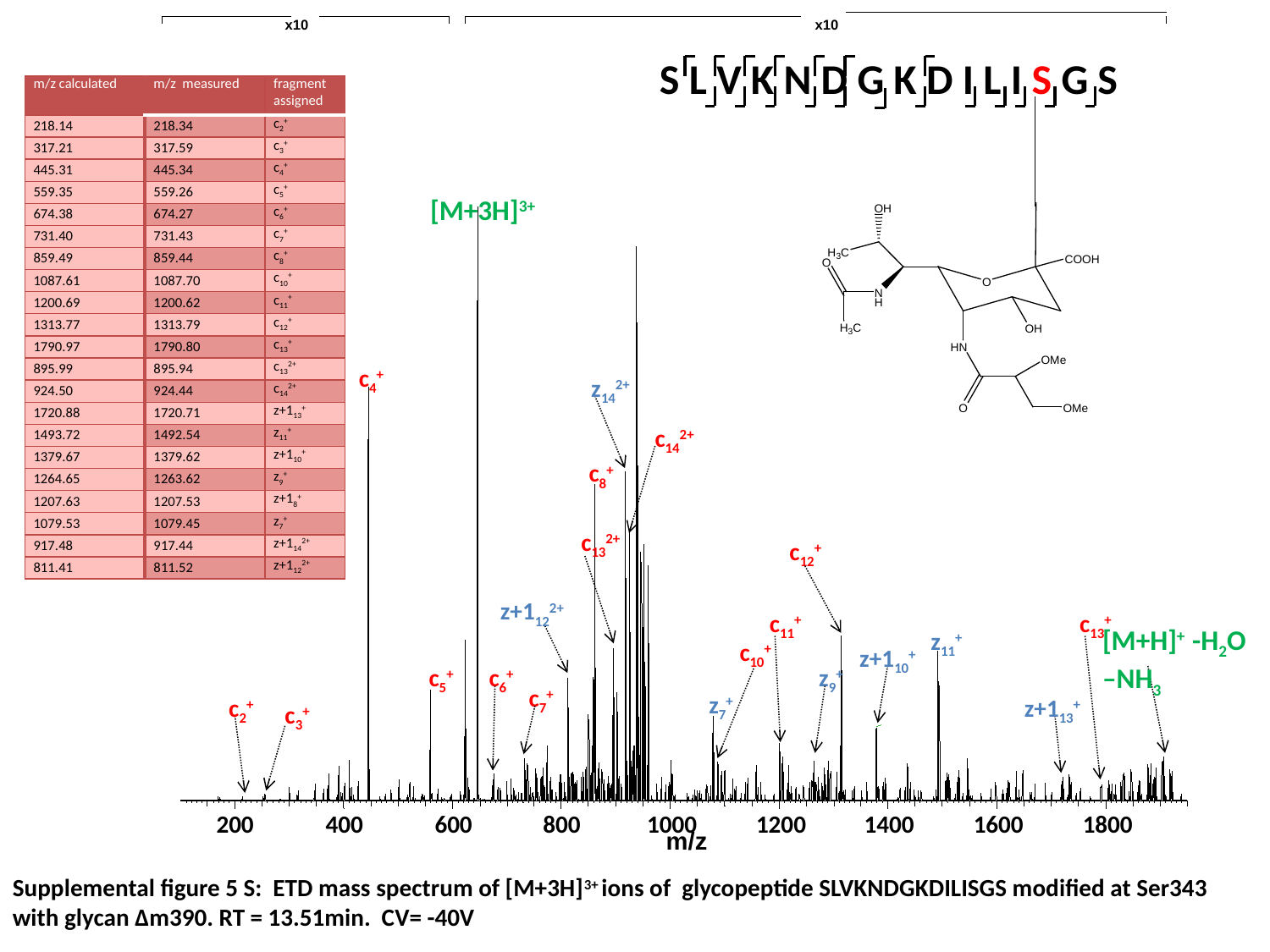

x10
x10
S L V K N D G K D I L I S G S
| m/z calculated | m/z measured | fragment assigned |
| --- | --- | --- |
| 218.14 | 218.34 | c2+ |
| 317.21 | 317.59 | c3+ |
| 445.31 | 445.34 | c4+ |
| 559.35 | 559.26 | c5+ |
| 674.38 | 674.27 | c6+ |
| 731.40 | 731.43 | c7+ |
| 859.49 | 859.44 | c8+ |
| 1087.61 | 1087.70 | c10+ |
| 1200.69 | 1200.62 | c11+ |
| 1313.77 | 1313.79 | c12+ |
| 1790.97 | 1790.80 | c13+ |
| 895.99 | 895.94 | c132+ |
| 924.50 | 924.44 | c142+ |
| 1720.88 | 1720.71 | z+113+ |
| 1493.72 | 1492.54 | z11+ |
| 1379.67 | 1379.62 | z+110+ |
| 1264.65 | 1263.62 | z9+ |
| 1207.63 | 1207.53 | z+18+ |
| 1079.53 | 1079.45 | z7+ |
| 917.48 | 917.44 | z+1142+ |
| 811.41 | 811.52 | z+1122+ |
[M+3H]3+
c4+
z142+
c142+
c8+
c132+
c12+
z+1122+
c11+
c13+
[M+H]+ -H2O –NH3
z11+
c10+
z+110+
c5+
c6+
z9+
c7+
z7+
c2+
z+113+
c3+
200
400
600
800
1000
1200
1400
1600
1800
m/z
Supplemental figure 5 S: ETD mass spectrum of [M+3H]3+ ions of glycopeptide SLVKNDGKDILISGS modified at Ser343 with glycan Δm390. RT = 13.51min. CV= -40V

## Slide 20
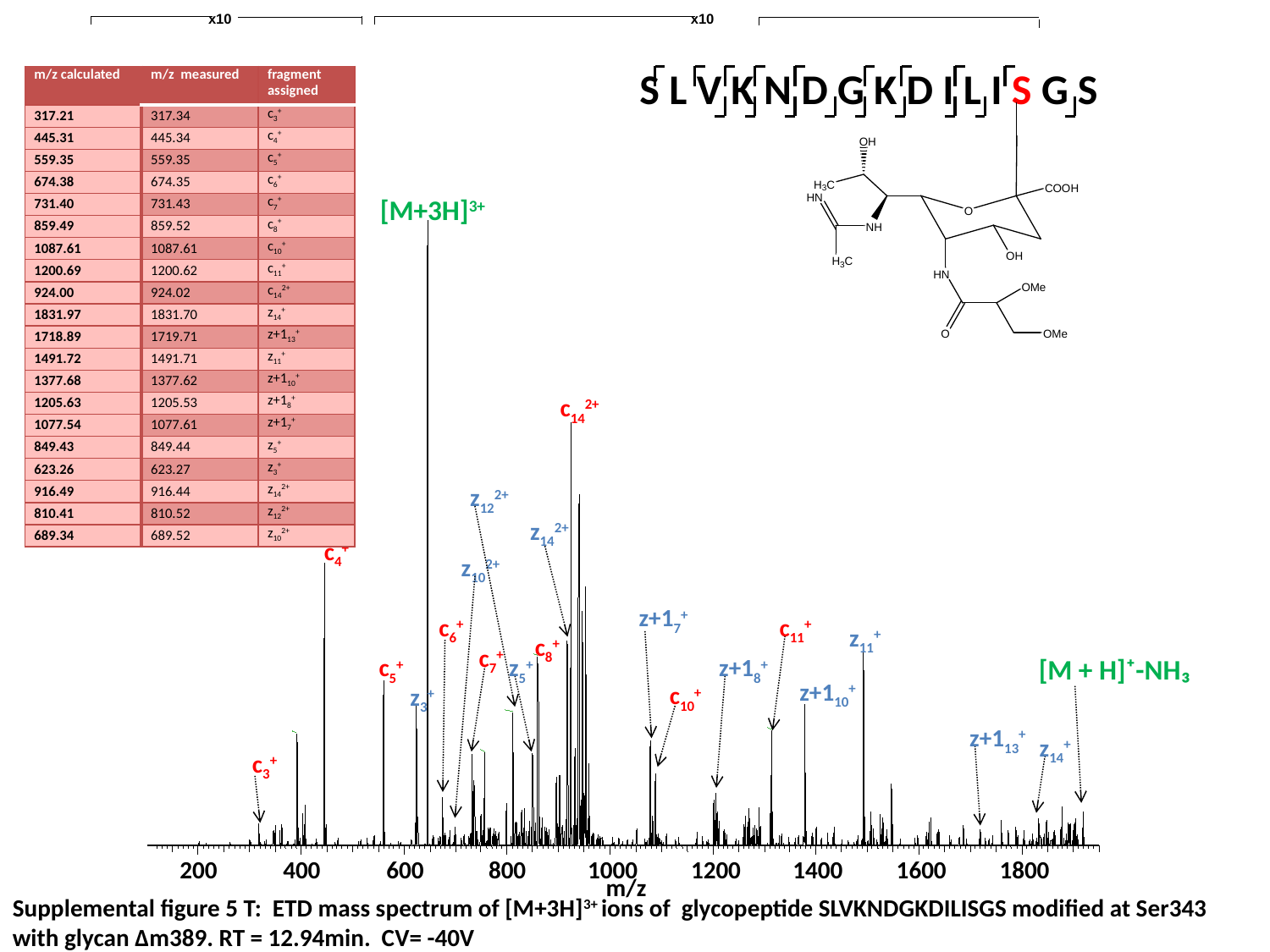

x10
x10
S L V K N D G K D I L I S G S
| m/z calculated | m/z measured | fragment assigned |
| --- | --- | --- |
| 317.21 | 317.34 | c3+ |
| 445.31 | 445.34 | c4+ |
| 559.35 | 559.35 | c5+ |
| 674.38 | 674.35 | c6+ |
| 731.40 | 731.43 | c7+ |
| 859.49 | 859.52 | c8+ |
| 1087.61 | 1087.61 | c10+ |
| 1200.69 | 1200.62 | c11+ |
| 924.00 | 924.02 | c142+ |
| 1831.97 | 1831.70 | z14+ |
| 1718.89 | 1719.71 | z+113+ |
| 1491.72 | 1491.71 | z11+ |
| 1377.68 | 1377.62 | z+110+ |
| 1205.63 | 1205.53 | z+18+ |
| 1077.54 | 1077.61 | z+17+ |
| 849.43 | 849.44 | z5+ |
| 623.26 | 623.27 | z3+ |
| 916.49 | 916.44 | z142+ |
| 810.41 | 810.52 | z122+ |
| 689.34 | 689.52 | z102+ |
[M+3H]3+
c142+
z122+
z142+
c4+
z102+
z+17+
c6+
c11+
z11+
c8+
c7+
c5+
z5+
z+18+
[M + H]⁺-NH₃
z+110+
c10+
z3+
z+113+
z14+
c3+
200
400
600
800
1000
1200
1400
1600
1800
m/z
Supplemental figure 5 T: ETD mass spectrum of [M+3H]3+ ions of glycopeptide SLVKNDGKDILISGS modified at Ser343 with glycan Δm389. RT = 12.94min. CV= -40V

## Slide 21
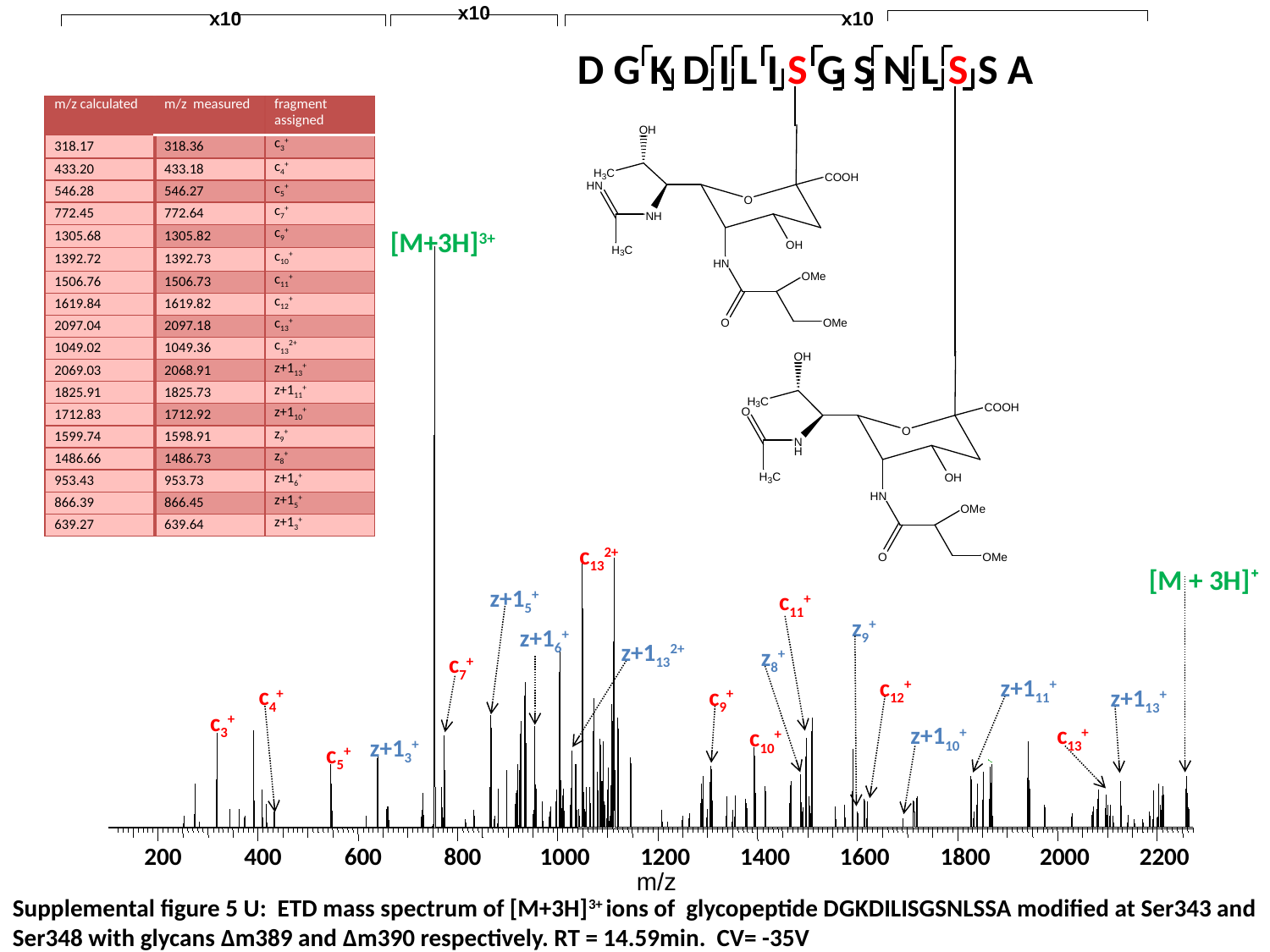

x10
x10
x10
D G K D I L I S G S N L S S A
| m/z calculated | m/z measured | fragment assigned |
| --- | --- | --- |
| 318.17 | 318.36 | c3+ |
| 433.20 | 433.18 | c4+ |
| 546.28 | 546.27 | c5+ |
| 772.45 | 772.64 | c7+ |
| 1305.68 | 1305.82 | c9+ |
| 1392.72 | 1392.73 | c10+ |
| 1506.76 | 1506.73 | c11+ |
| 1619.84 | 1619.82 | c12+ |
| 2097.04 | 2097.18 | c13+ |
| 1049.02 | 1049.36 | c132+ |
| 2069.03 | 2068.91 | z+113+ |
| 1825.91 | 1825.73 | z+111+ |
| 1712.83 | 1712.92 | z+110+ |
| 1599.74 | 1598.91 | z9+ |
| 1486.66 | 1486.73 | z8+ |
| 953.43 | 953.73 | z+16+ |
| 866.39 | 866.45 | z+15+ |
| 639.27 | 639.64 | z+13+ |
[M+3H]3+
c132+
[M + 3H]⁺
z+15+
c11+
z9+
z+16+
z+1132+
z8+
c7+
c12+
z+111+
c4+
c9+
z+113+
c3+
c13+
z+110+
c10+
z+13+
c5+
200
400
600
800
1000
1200
1400
1600
1800
2000
2200
m/z
Supplemental figure 5 U: ETD mass spectrum of [M+3H]3+ ions of glycopeptide DGKDILISGSNLSSA modified at Ser343 and Ser348 with glycans Δm389 and Δm390 respectively. RT = 14.59min. CV= -35V

## Slide 22
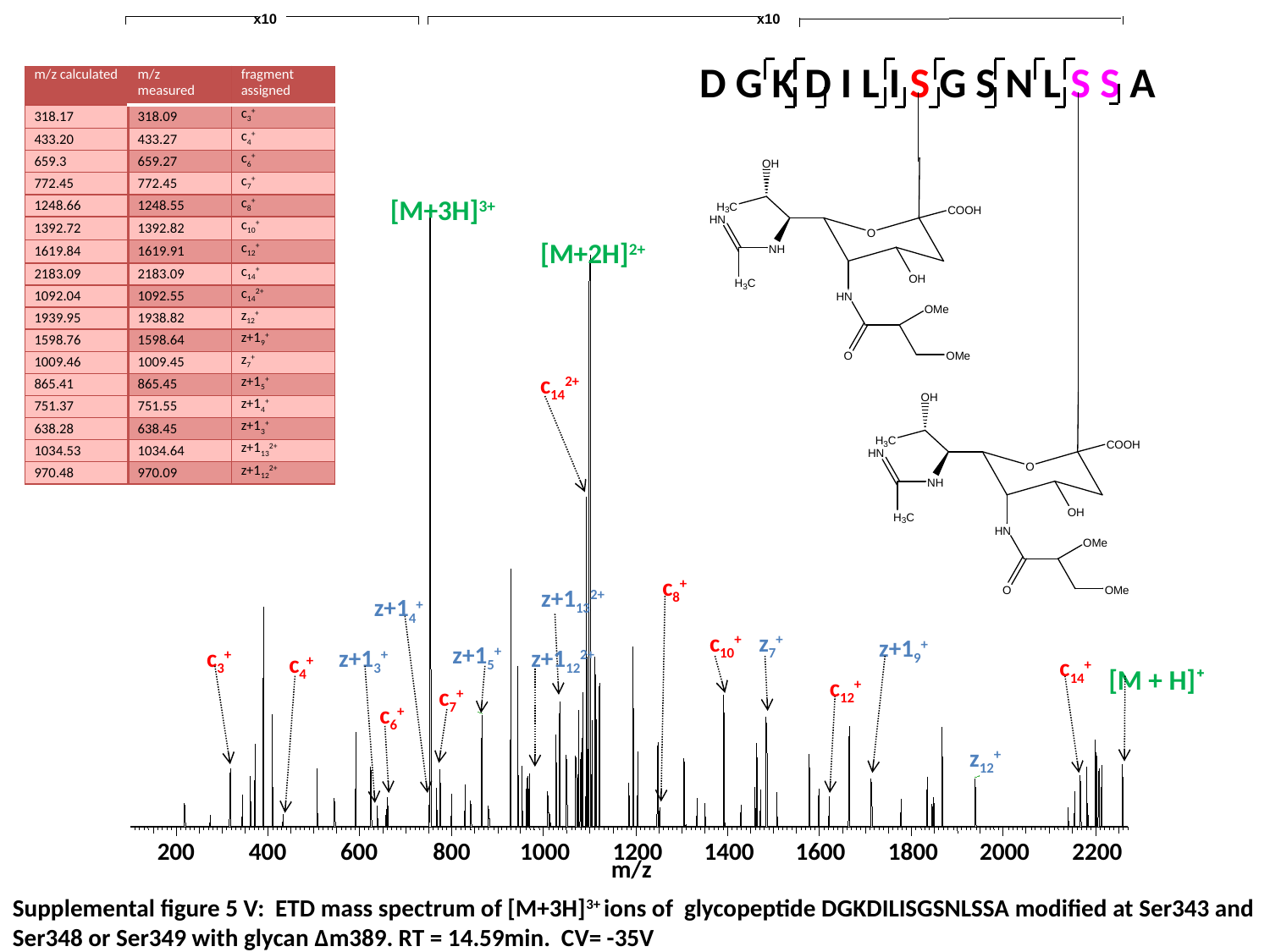

x10
x10
D G K D I L I S G S N L S S A
| m/z calculated | m/z measured | fragment assigned |
| --- | --- | --- |
| 318.17 | 318.09 | c3+ |
| 433.20 | 433.27 | c4+ |
| 659.3 | 659.27 | c6+ |
| 772.45 | 772.45 | c7+ |
| 1248.66 | 1248.55 | c8+ |
| 1392.72 | 1392.82 | c10+ |
| 1619.84 | 1619.91 | c12+ |
| 2183.09 | 2183.09 | c14+ |
| 1092.04 | 1092.55 | c142+ |
| 1939.95 | 1938.82 | z12+ |
| 1598.76 | 1598.64 | z+19+ |
| 1009.46 | 1009.45 | z7+ |
| 865.41 | 865.45 | z+15+ |
| 751.37 | 751.55 | z+14+ |
| 638.28 | 638.45 | z+13+ |
| 1034.53 | 1034.64 | z+1132+ |
| 970.48 | 970.09 | z+1122+ |
[M+3H]3+
[M+2H]2+
c142+
c8+
z+1132+
z+14+
c10+
z7+
z+19+
z+15+
c3+
z+13+
z+1122+
c4+
c14+
[M + H]⁺
c12+
c7+
c6+
z12+
200
400
600
800
1000
1200
1400
1600
1800
2000
2200
m/z
Supplemental figure 5 V: ETD mass spectrum of [M+3H]3+ ions of glycopeptide DGKDILISGSNLSSA modified at Ser343 and Ser348 or Ser349 with glycan Δm389. RT = 14.59min. CV= -35V

## Slide 23
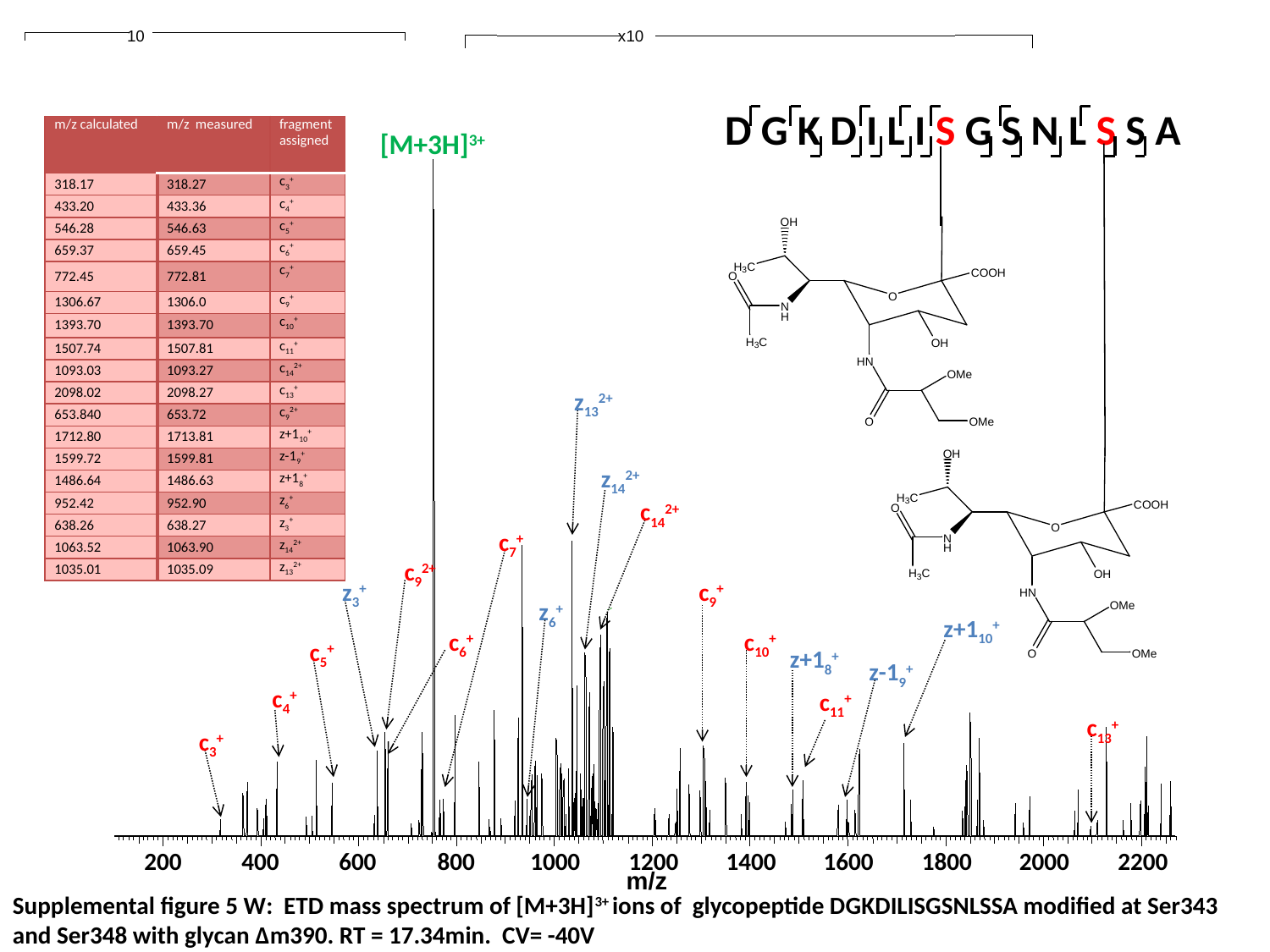

10
x10
D G K D I L I S G S N L S S A
| m/z calculated | m/z measured | fragment assigned |
| --- | --- | --- |
| 318.17 | 318.27 | c3+ |
| 433.20 | 433.36 | c4+ |
| 546.28 | 546.63 | c5+ |
| 659.37 | 659.45 | c6+ |
| 772.45 | 772.81 | c7+ |
| 1306.67 | 1306.0 | c9+ |
| 1393.70 | 1393.70 | c10+ |
| 1507.74 | 1507.81 | c11+ |
| 1093.03 | 1093.27 | c142+ |
| 2098.02 | 2098.27 | c13+ |
| 653.840 | 653.72 | c92+ |
| 1712.80 | 1713.81 | z+110+ |
| 1599.72 | 1599.81 | z-19+ |
| 1486.64 | 1486.63 | z+18+ |
| 952.42 | 952.90 | z6+ |
| 638.26 | 638.27 | z3+ |
| 1063.52 | 1063.90 | z142+ |
| 1035.01 | 1035.09 | z132+ |
[M+3H]3+
z132+
z142+
c142+
c7+
c92+
z3+
c9+
z6+
z+110+
c6+
c10+
c5+
z+18+
z-19+
c4+
c11+
c13+
c3+
200
400
600
800
1000
1200
1400
1600
1800
2000
2200
m/z
Supplemental figure 5 W: ETD mass spectrum of [M+3H]3+ ions of glycopeptide DGKDILISGSNLSSA modified at Ser343 and Ser348 with glycan Δm390. RT = 17.34min. CV= -40V

## Slide 24
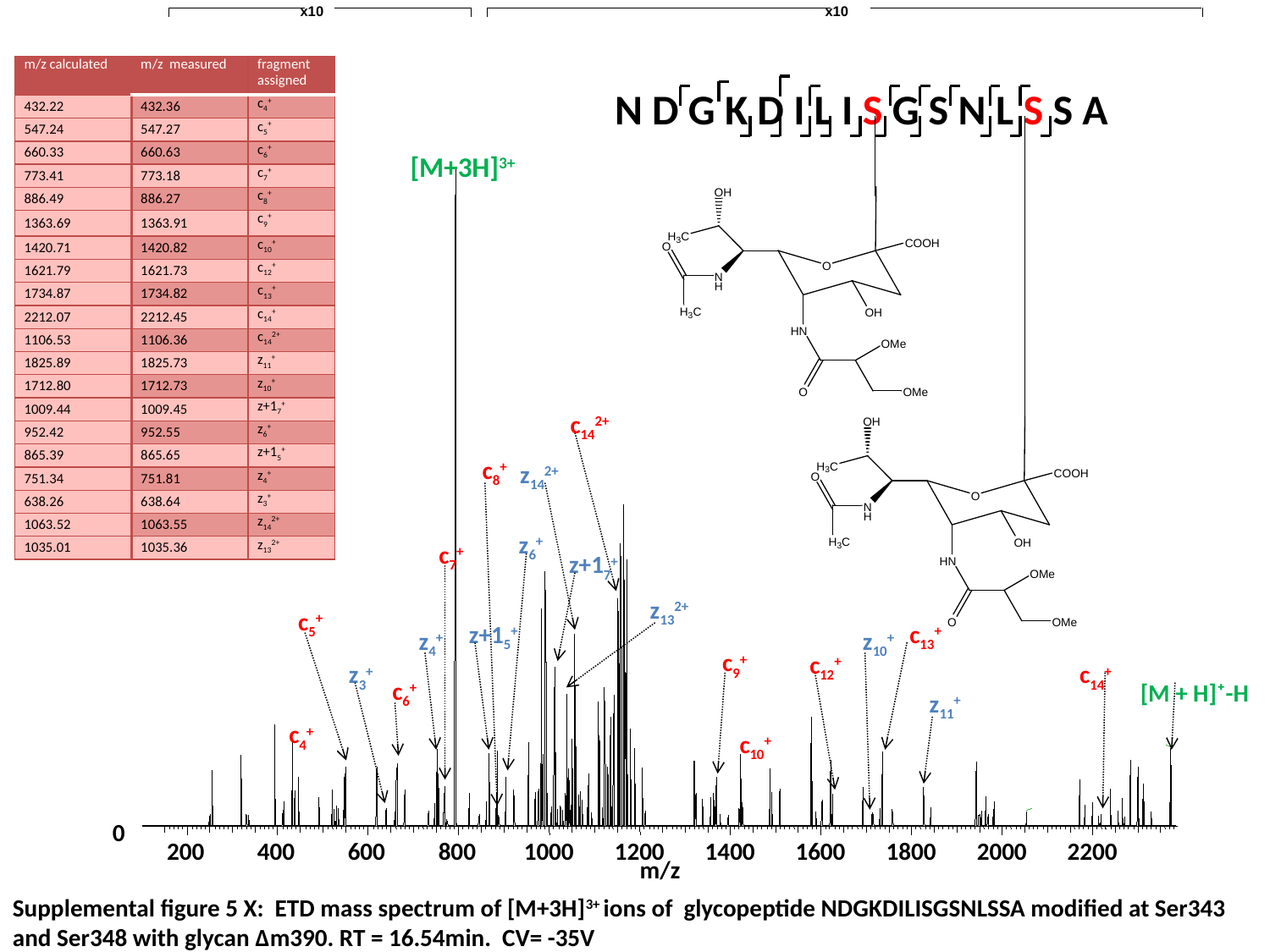

x10
x10
| m/z calculated | m/z measured | fragment assigned |
| --- | --- | --- |
| 432.22 | 432.36 | c4+ |
| 547.24 | 547.27 | c5+ |
| 660.33 | 660.63 | c6+ |
| 773.41 | 773.18 | c7+ |
| 886.49 | 886.27 | c8+ |
| 1363.69 | 1363.91 | c9+ |
| 1420.71 | 1420.82 | c10+ |
| 1621.79 | 1621.73 | c12+ |
| 1734.87 | 1734.82 | c13+ |
| 2212.07 | 2212.45 | c14+ |
| 1106.53 | 1106.36 | c142+ |
| 1825.89 | 1825.73 | z11+ |
| 1712.80 | 1712.73 | z10+ |
| 1009.44 | 1009.45 | z+17+ |
| 952.42 | 952.55 | z6+ |
| 865.39 | 865.65 | z+15+ |
| 751.34 | 751.81 | z4+ |
| 638.26 | 638.64 | z3+ |
| 1063.52 | 1063.55 | z142+ |
| 1035.01 | 1035.36 | z132+ |
N D G K D I L I S G S N L S S A
[M+3H]3+
c142+
c8+
z142+
z6+
c7+
z+17+
z132+
c5+
z+15+
c13+
z4+
z10+
c9+
c12+
z3+
c14+
c6+
[M + H]⁺-H
z11+
c4+
c10+
0
200
400
600
800
1000
1200
1400
1600
1800
2000
2200
m/z
Supplemental figure 5 X: ETD mass spectrum of [M+3H]3+ ions of glycopeptide NDGKDILISGSNLSSA modified at Ser343 and Ser348 with glycan Δm390. RT = 16.54min. CV= -35V

## Slide 25
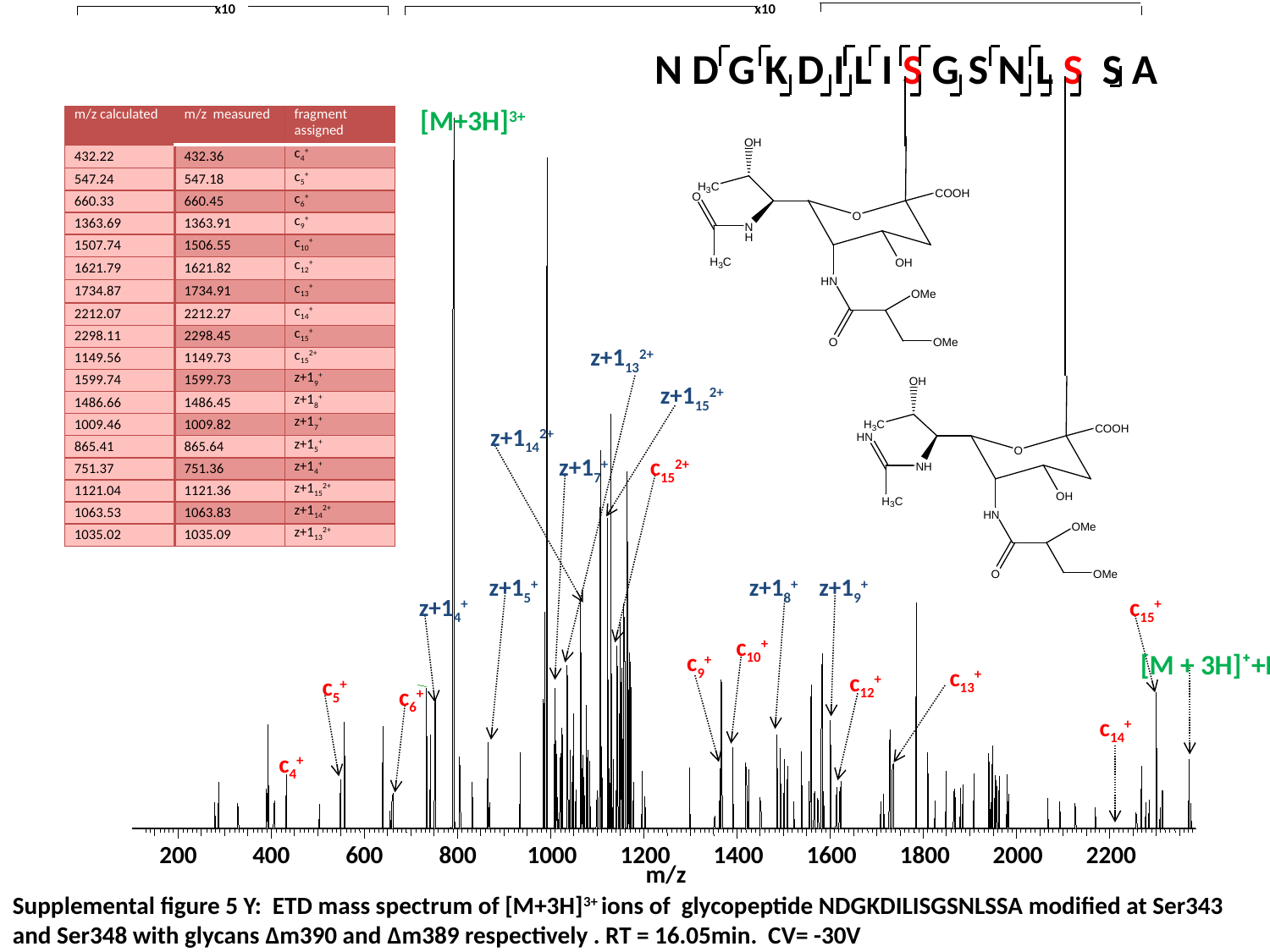

x10
x10
N D G K D I L I S G S N L S S A
[M+3H]3+
| m/z calculated | m/z measured | fragment assigned |
| --- | --- | --- |
| 432.22 | 432.36 | c4+ |
| 547.24 | 547.18 | c5+ |
| 660.33 | 660.45 | c6+ |
| 1363.69 | 1363.91 | c9+ |
| 1507.74 | 1506.55 | c10+ |
| 1621.79 | 1621.82 | c12+ |
| 1734.87 | 1734.91 | c13+ |
| 2212.07 | 2212.27 | c14+ |
| 2298.11 | 2298.45 | c15+ |
| 1149.56 | 1149.73 | c152+ |
| 1599.74 | 1599.73 | z+19+ |
| 1486.66 | 1486.45 | z+18+ |
| 1009.46 | 1009.82 | z+17+ |
| 865.41 | 865.64 | z+15+ |
| 751.37 | 751.36 | z+14+ |
| 1121.04 | 1121.36 | z+1152+ |
| 1063.53 | 1063.83 | z+1142+ |
| 1035.02 | 1035.09 | z+1132+ |
z+1132+
z+1152+
z+1142+
z+17+
c152+
z+15+
z+18+
z+19+
z+14+
c15+
c10+
[M + 3H]⁺+H
c9+
c13+
c12+
c5+
c6+
c14+
c4+
200
400
600
800
1000
1200
1400
1600
1800
2000
2200
m/z
Supplemental figure 5 Y: ETD mass spectrum of [M+3H]3+ ions of glycopeptide NDGKDILISGSNLSSA modified at Ser343 and Ser348 with glycans Δm390 and Δm389 respectively . RT = 16.05min. CV= -30V

## Slide 26
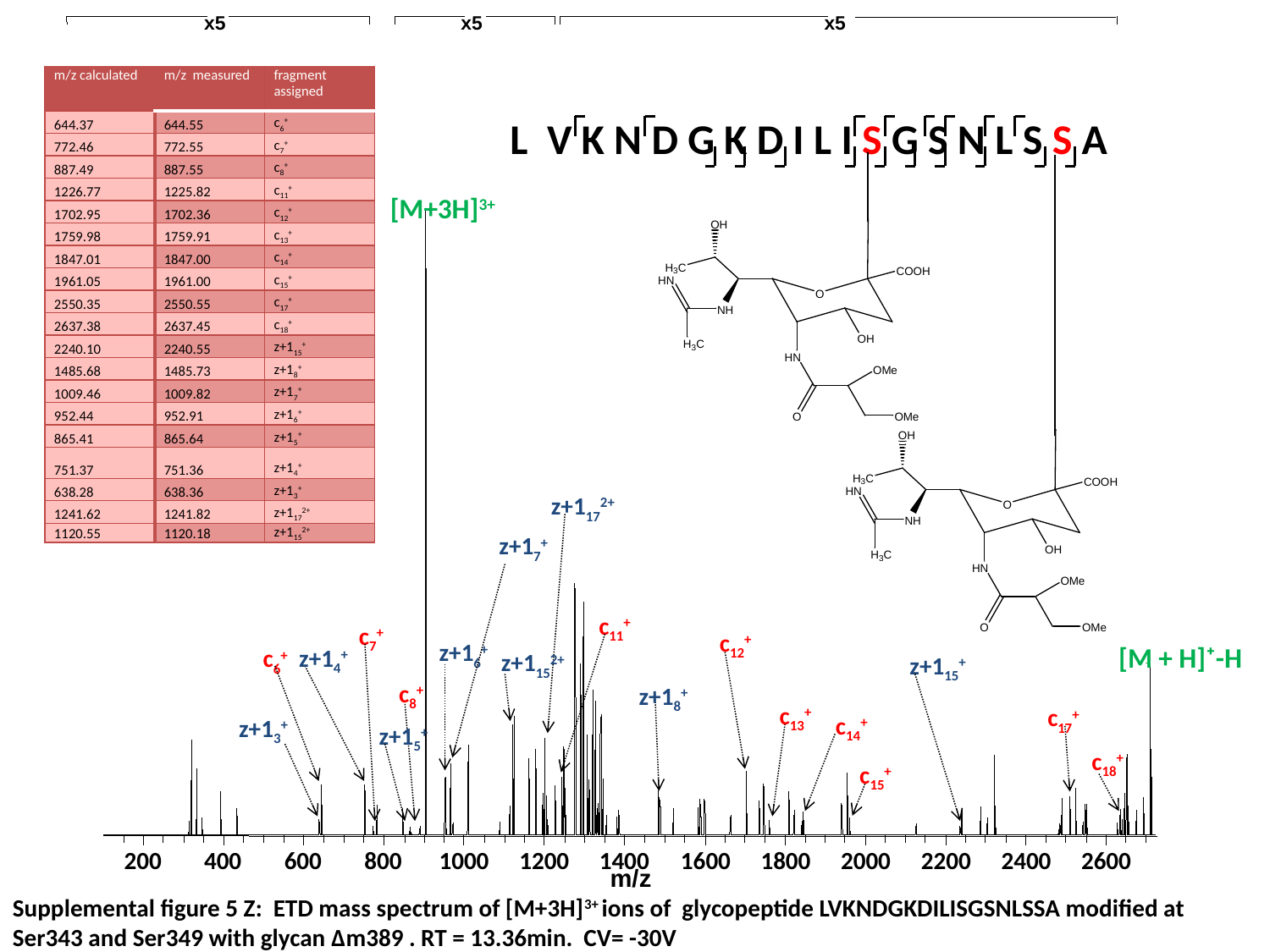

x5
x5
x5
| m/z calculated | m/z measured | fragment assigned |
| --- | --- | --- |
| 644.37 | 644.55 | c6+ |
| 772.46 | 772.55 | c7+ |
| 887.49 | 887.55 | c8+ |
| 1226.77 | 1225.82 | c11+ |
| 1702.95 | 1702.36 | c12+ |
| 1759.98 | 1759.91 | c13+ |
| 1847.01 | 1847.00 | c14+ |
| 1961.05 | 1961.00 | c15+ |
| 2550.35 | 2550.55 | c17+ |
| 2637.38 | 2637.45 | c18+ |
| 2240.10 | 2240.55 | z+115+ |
| 1485.68 | 1485.73 | z+18+ |
| 1009.46 | 1009.82 | z+17+ |
| 952.44 | 952.91 | z+16+ |
| 865.41 | 865.64 | z+15+ |
| 751.37 | 751.36 | z+14+ |
| 638.28 | 638.36 | z+13+ |
| 1241.62 | 1241.82 | z+1172+ |
| 1120.55 | 1120.18 | z+1152+ |
L V K N D G K D I L I S G S N L S S A
[M+3H]3+
z+1172+
z+17+
c11+
c7+
c12+
z+16+
[M + H]⁺-H
z+14+
c6+
z+1152+
z+115+
c8+
z+18+
c13+
c17+
c14+
z+13+
z+15+
c18+
c15+
200
400
600
800
1000
1200
1400
1600
1800
2000
2200
2400
2600
m/z
Supplemental figure 5 Z: ETD mass spectrum of [M+3H]3+ ions of glycopeptide LVKNDGKDILISGSNLSSA modified at Ser343 and Ser349 with glycan Δm389 . RT = 13.36min. CV= -30V

## Slide 27
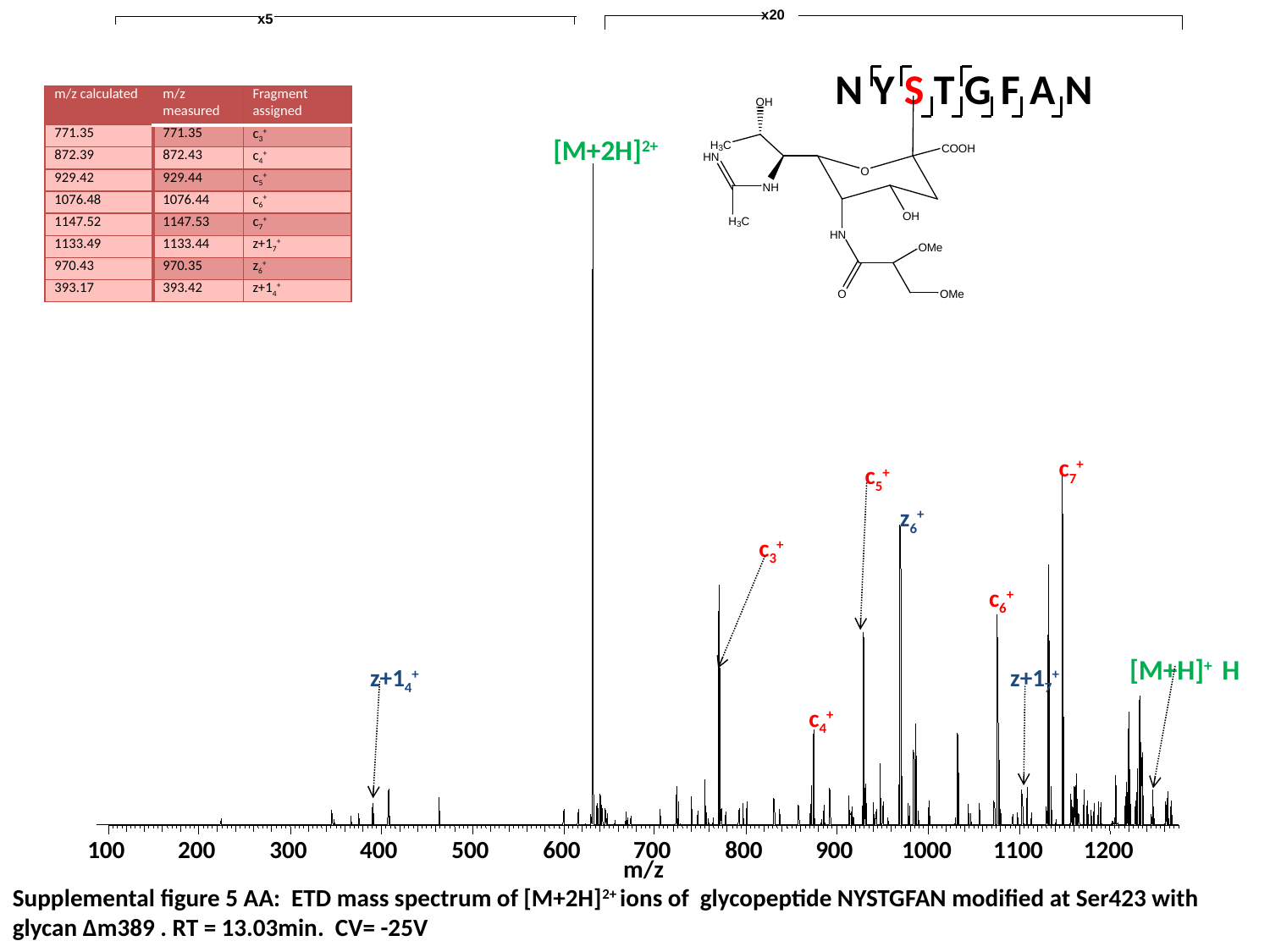

x20
x5
N Y S T G F A N
| m/z calculated | m/z measured | Fragment assigned |
| --- | --- | --- |
| 771.35 | 771.35 | c3+ |
| 872.39 | 872.43 | c4+ |
| 929.42 | 929.44 | c5+ |
| 1076.48 | 1076.44 | c6+ |
| 1147.52 | 1147.53 | c7+ |
| 1133.49 | 1133.44 | z+17+ |
| 970.43 | 970.35 | z6+ |
| 393.17 | 393.42 | z+14+ |
[M+2H]2+
c7+
c5+
z6+
c3+
c6+
[M+H]+ H
z+14+
z+17+
c4+
100
200
300
400
500
600
700
800
900
1000
1100
1200
m/z
Supplemental figure 5 AA: ETD mass spectrum of [M+2H]2+ ions of glycopeptide NYSTGFAN modified at Ser423 with glycan Δm389 . RT = 13.03min. CV= -25V

## Slide 28
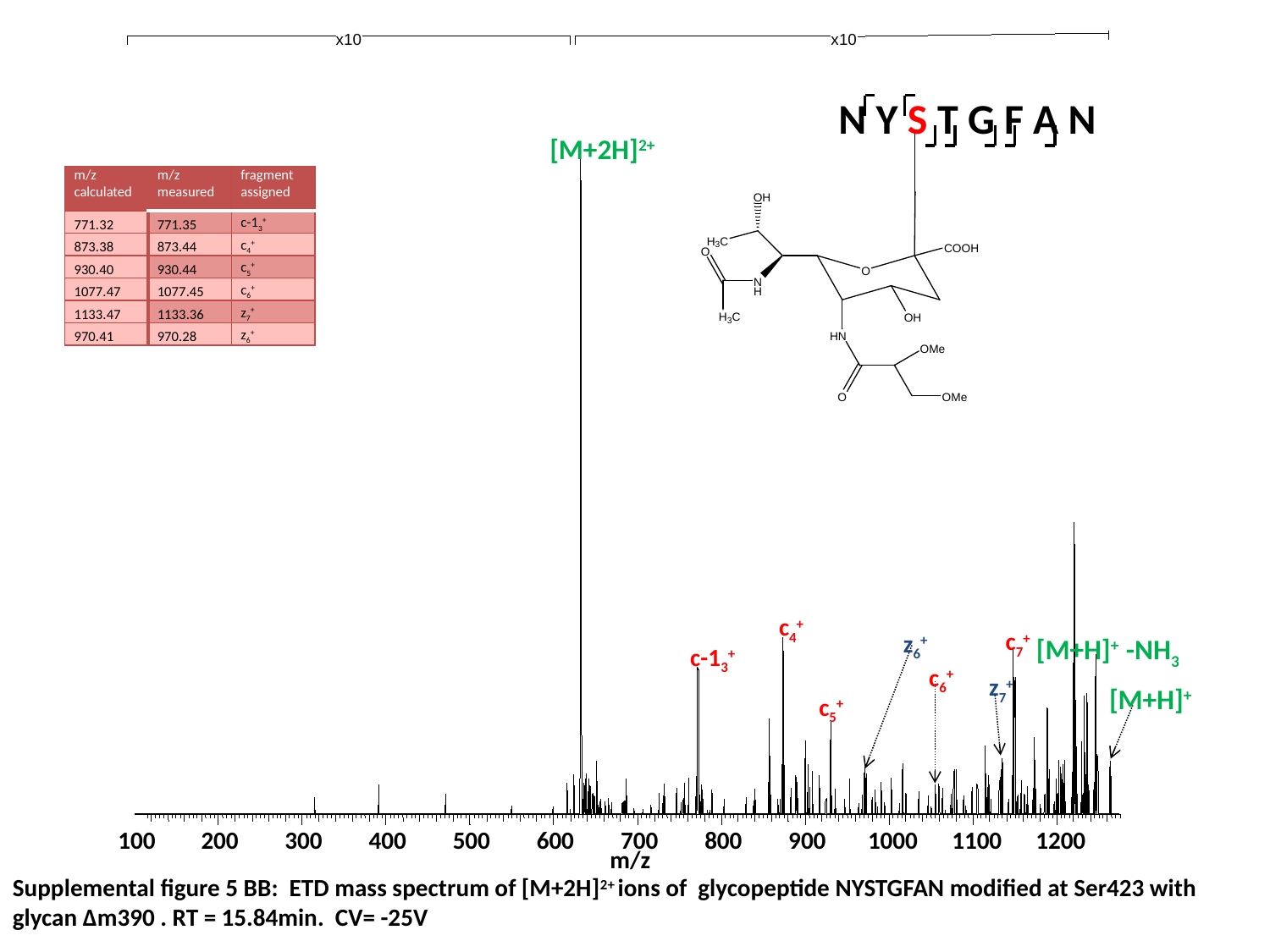

x10
x10
N Y S T G F A N
[M+2H]2+
| m/z calculated | m/z measured | fragment assigned |
| --- | --- | --- |
| 771.32 | 771.35 | c-13+ |
| 873.38 | 873.44 | c4+ |
| 930.40 | 930.44 | c5+ |
| 1077.47 | 1077.45 | c6+ |
| 1133.47 | 1133.36 | z7+ |
| 970.41 | 970.28 | z6+ |
c4+
c7+
z6+
[M+H]+ -NH3
c-13+
c6+
z7+
[M+H]+
c5+
100
200
300
400
500
600
700
800
900
1000
1100
1200
m/z
Supplemental figure 5 BB: ETD mass spectrum of [M+2H]2+ ions of glycopeptide NYSTGFAN modified at Ser423 with glycan Δm390 . RT = 15.84min. CV= -25V

## Slide 29
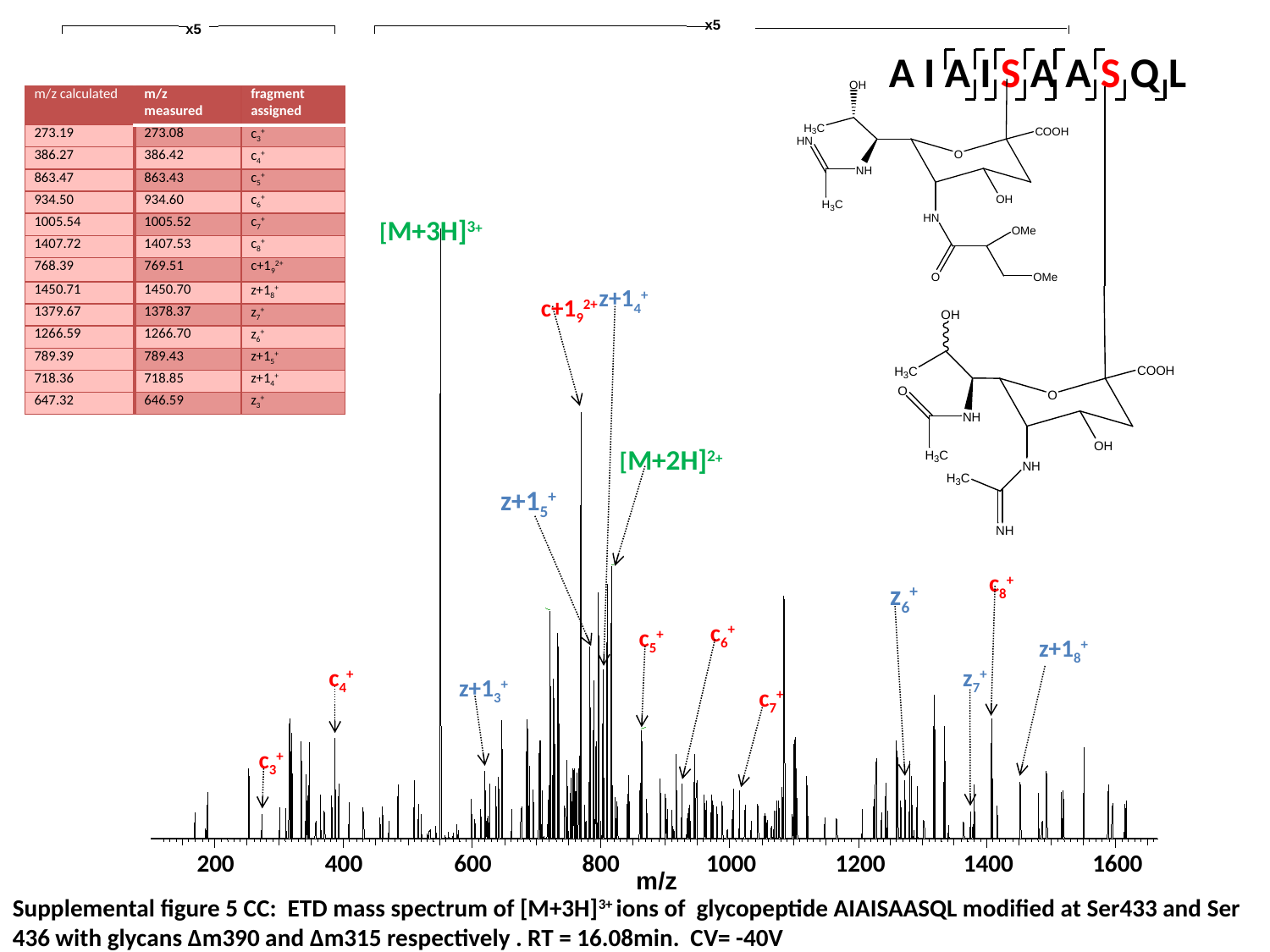

x5
x5
A I A I S A A S Q L
| m/z calculated | m/z measured | fragment assigned |
| --- | --- | --- |
| 273.19 | 273.08 | c3+ |
| 386.27 | 386.42 | c4+ |
| 863.47 | 863.43 | c5+ |
| 934.50 | 934.60 | c6+ |
| 1005.54 | 1005.52 | c7+ |
| 1407.72 | 1407.53 | c8+ |
| 768.39 | 769.51 | c+192+ |
| 1450.71 | 1450.70 | z+18+ |
| 1379.67 | 1378.37 | z7+ |
| 1266.59 | 1266.70 | z6+ |
| 789.39 | 789.43 | z+15+ |
| 718.36 | 718.85 | z+14+ |
| 647.32 | 646.59 | z3+ |
[M+3H]3+
z+14+
c+192+
[M+2H]2+
z+15+
c8+
z6+
c6+
c5+
z+18+
c4+
z7+
z+13+
c7+
c3+
200
400
600
800
1000
1200
1400
1600
m/z
Supplemental figure 5 CC: ETD mass spectrum of [M+3H]3+ ions of glycopeptide AIAISAASQL modified at Ser433 and Ser 436 with glycans Δm390 and Δm315 respectively . RT = 16.08min. CV= -40V

## Slide 30
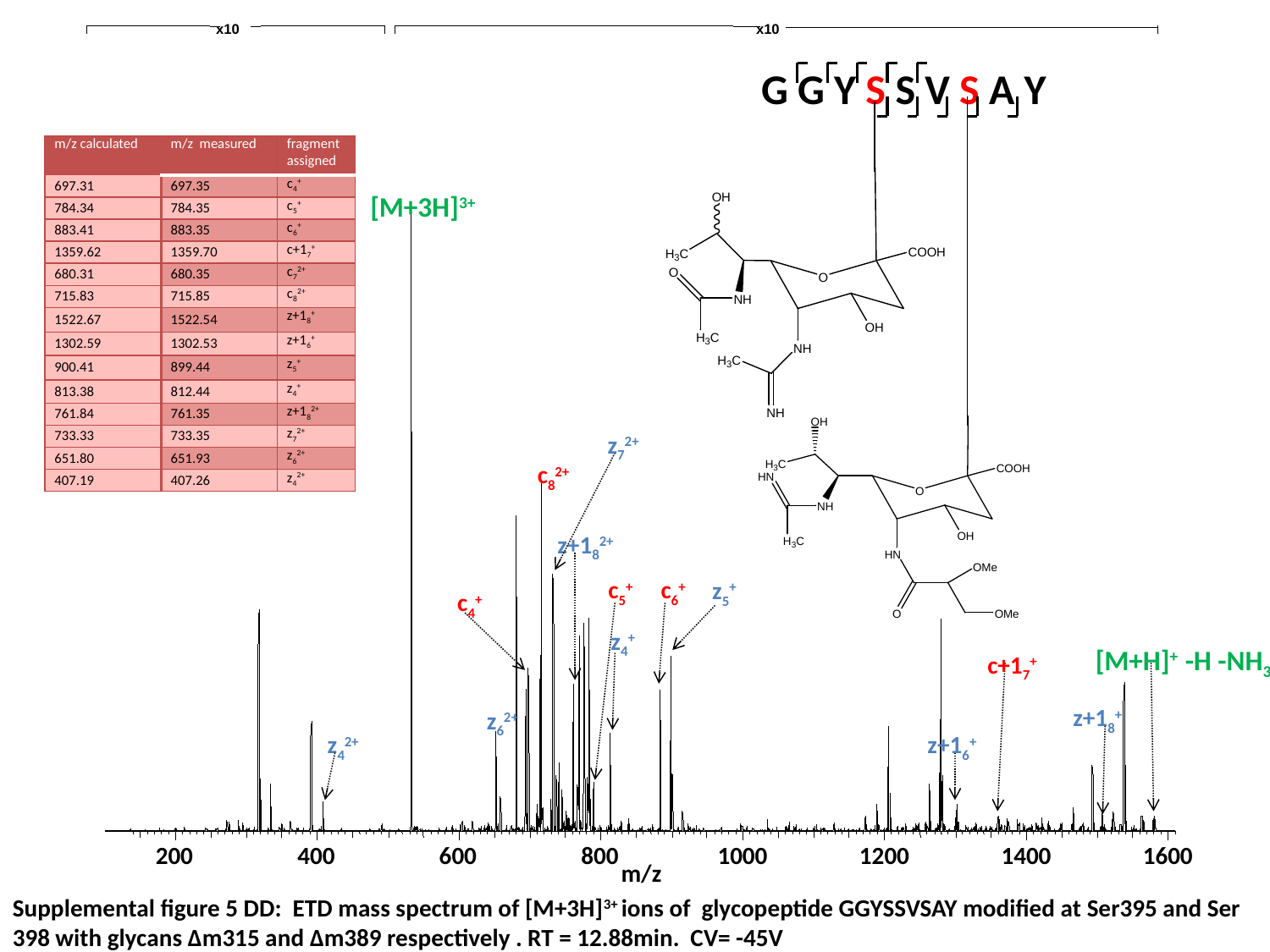

x10
x10
G G Y S S V S A Y
| m/z calculated | m/z measured | fragment assigned |
| --- | --- | --- |
| 697.31 | 697.35 | c4+ |
| 784.34 | 784.35 | c5+ |
| 883.41 | 883.35 | c6+ |
| 1359.62 | 1359.70 | c+17+ |
| 680.31 | 680.35 | c72+ |
| 715.83 | 715.85 | c82+ |
| 1522.67 | 1522.54 | z+18+ |
| 1302.59 | 1302.53 | z+16+ |
| 900.41 | 899.44 | z5+ |
| 813.38 | 812.44 | z4+ |
| 761.84 | 761.35 | z+182+ |
| 733.33 | 733.35 | z72+ |
| 651.80 | 651.93 | z62+ |
| 407.19 | 407.26 | z42+ |
[M+3H]3+
z72+
c82+
z+182+
c5+
c6+
z5+
c4+
z4+
[M+H]+ -H -NH3
c+17+
z+18+
z62+
z42+
z+16+
200
400
600
800
1000
1200
1400
1600
m/z
Supplemental figure 5 DD: ETD mass spectrum of [M+3H]3+ ions of glycopeptide GGYSSVSAY modified at Ser395 and Ser 398 with glycans Δm315 and Δm389 respectively . RT = 12.88min. CV= -45V

## Slide 31
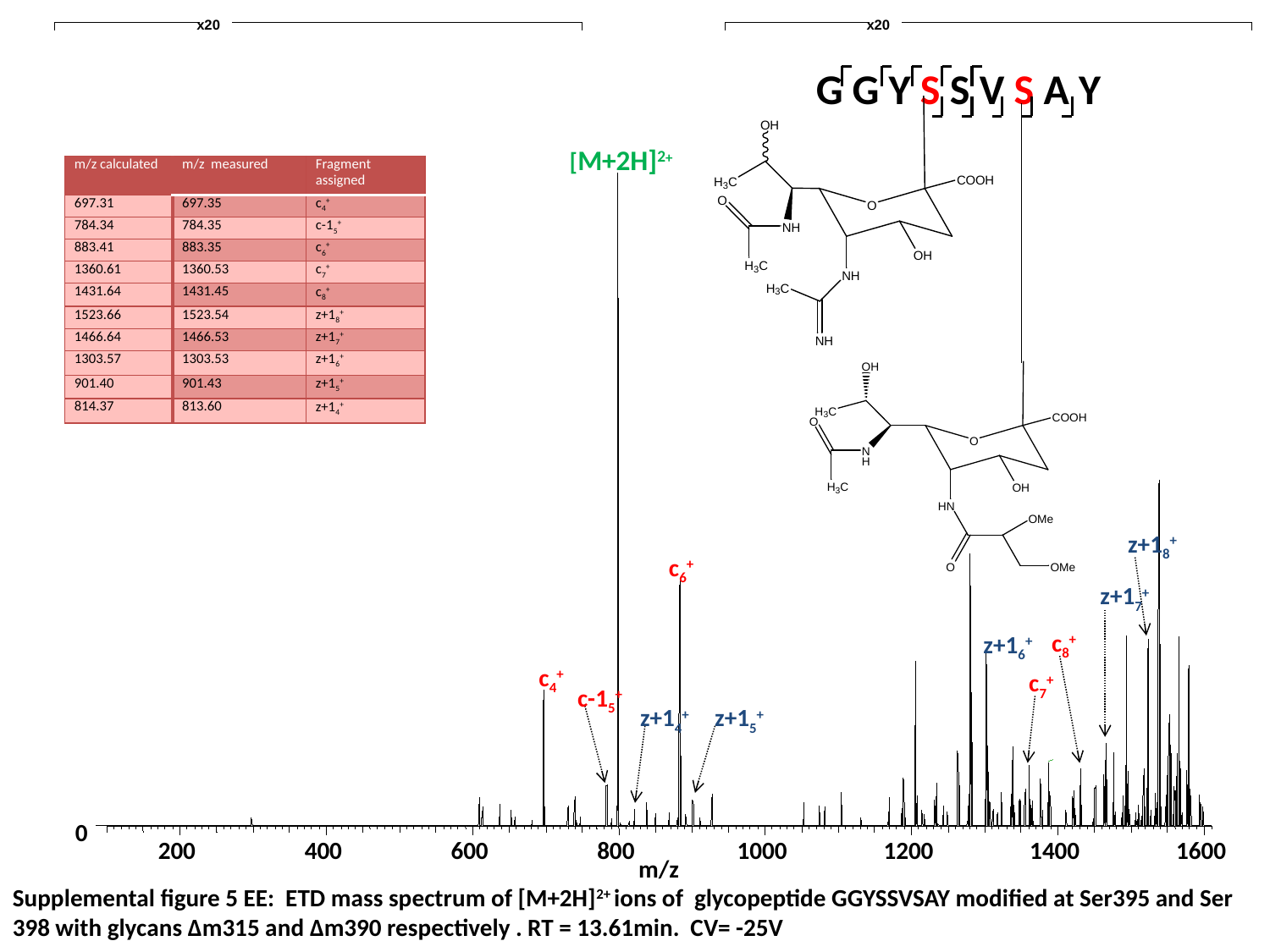

x20
x20
G G Y S S V S A Y
[M+2H]2+
| m/z calculated | m/z measured | Fragment assigned |
| --- | --- | --- |
| 697.31 | 697.35 | c4+ |
| 784.34 | 784.35 | c-15+ |
| 883.41 | 883.35 | c6+ |
| 1360.61 | 1360.53 | c7+ |
| 1431.64 | 1431.45 | c8+ |
| 1523.66 | 1523.54 | z+18+ |
| 1466.64 | 1466.53 | z+17+ |
| 1303.57 | 1303.53 | z+16+ |
| 901.40 | 901.43 | z+15+ |
| 814.37 | 813.60 | z+14+ |
z+18+
c6+
z+17+
c8+
z+16+
c4+
c7+
c-15+
z+14+
z+15+
0
200
400
600
800
1000
1200
1400
1600
m/z
Supplemental figure 5 EE: ETD mass spectrum of [M+2H]2+ ions of glycopeptide GGYSSVSAY modified at Ser395 and Ser 398 with glycans Δm315 and Δm390 respectively . RT = 13.61min. CV= -25V

## Slide 32
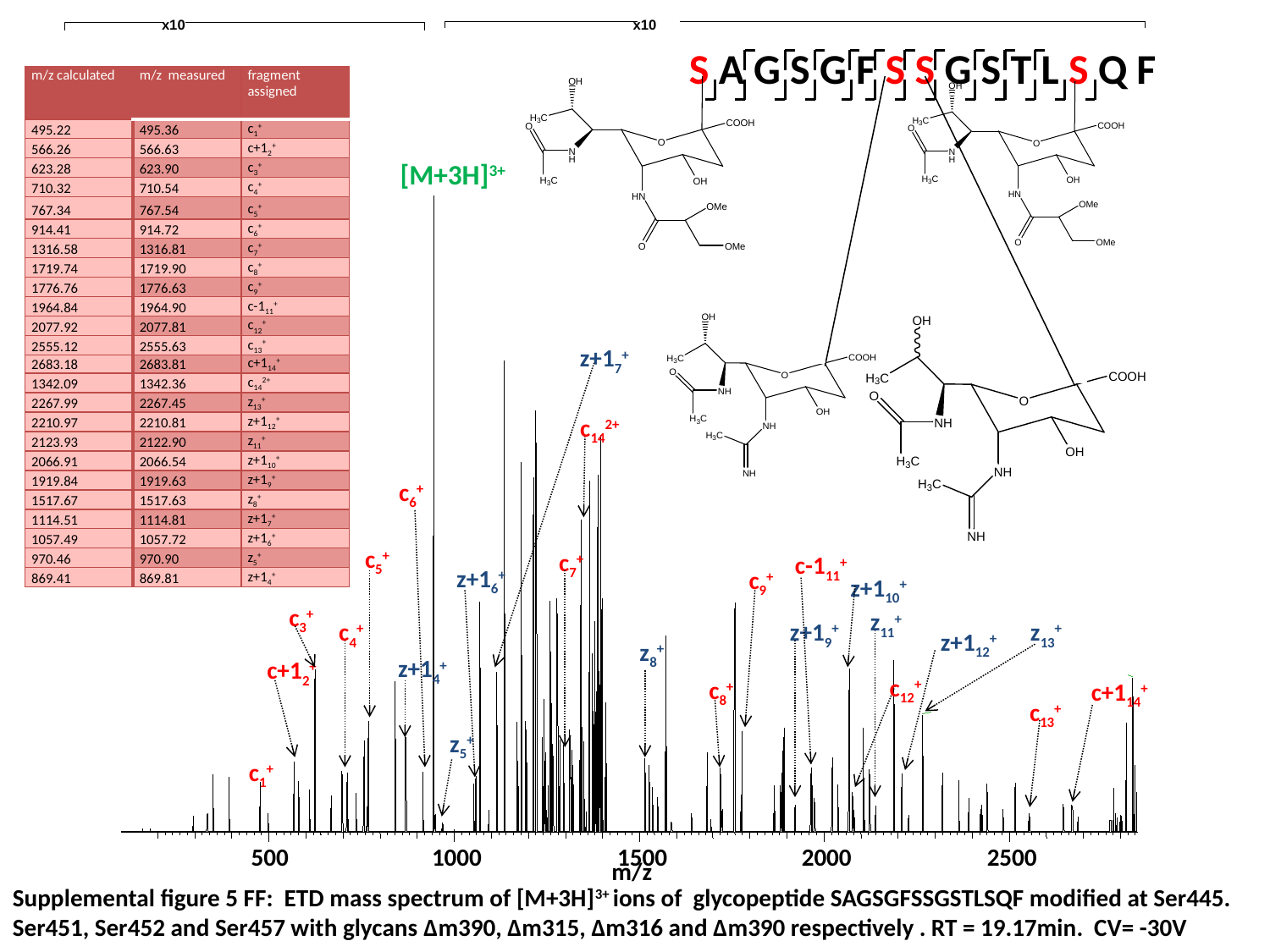

x10
x10
 S A G S G F S S G S T L S Q F
| m/z calculated | m/z measured | fragment assigned |
| --- | --- | --- |
| 495.22 | 495.36 | c1+ |
| 566.26 | 566.63 | c+12+ |
| 623.28 | 623.90 | c3+ |
| 710.32 | 710.54 | c4+ |
| 767.34 | 767.54 | c5+ |
| 914.41 | 914.72 | c6+ |
| 1316.58 | 1316.81 | c7+ |
| 1719.74 | 1719.90 | c8+ |
| 1776.76 | 1776.63 | c9+ |
| 1964.84 | 1964.90 | c-111+ |
| 2077.92 | 2077.81 | c12+ |
| 2555.12 | 2555.63 | c13+ |
| 2683.18 | 2683.81 | c+114+ |
| 1342.09 | 1342.36 | c142+ |
| 2267.99 | 2267.45 | z13+ |
| 2210.97 | 2210.81 | z+112+ |
| 2123.93 | 2122.90 | z11+ |
| 2066.91 | 2066.54 | z+110+ |
| 1919.84 | 1919.63 | z+19+ |
| 1517.67 | 1517.63 | z8+ |
| 1114.51 | 1114.81 | z+17+ |
| 1057.49 | 1057.72 | z+16+ |
| 970.46 | 970.90 | z5+ |
| 869.41 | 869.81 | z+14+ |
[M+3H]3+
z+17+
c142+
c6+
c5+
c7+
c-111+
z+16+
c9+
z+110+
c3+
z11+
c4+
z+19+
z13+
z+112+
z8+
z+14+
c+12+
c12+
c8+
c+114+
c13+
z5+
c1+
500
1000
1500
2000
2500
m/z
Supplemental figure 5 FF: ETD mass spectrum of [M+3H]3+ ions of glycopeptide SAGSGFSSGSTLSQF modified at Ser445. Ser451, Ser452 and Ser457 with glycans Δm390, Δm315, Δm316 and Δm390 respectively . RT = 19.17min. CV= -30V

## Slide 33
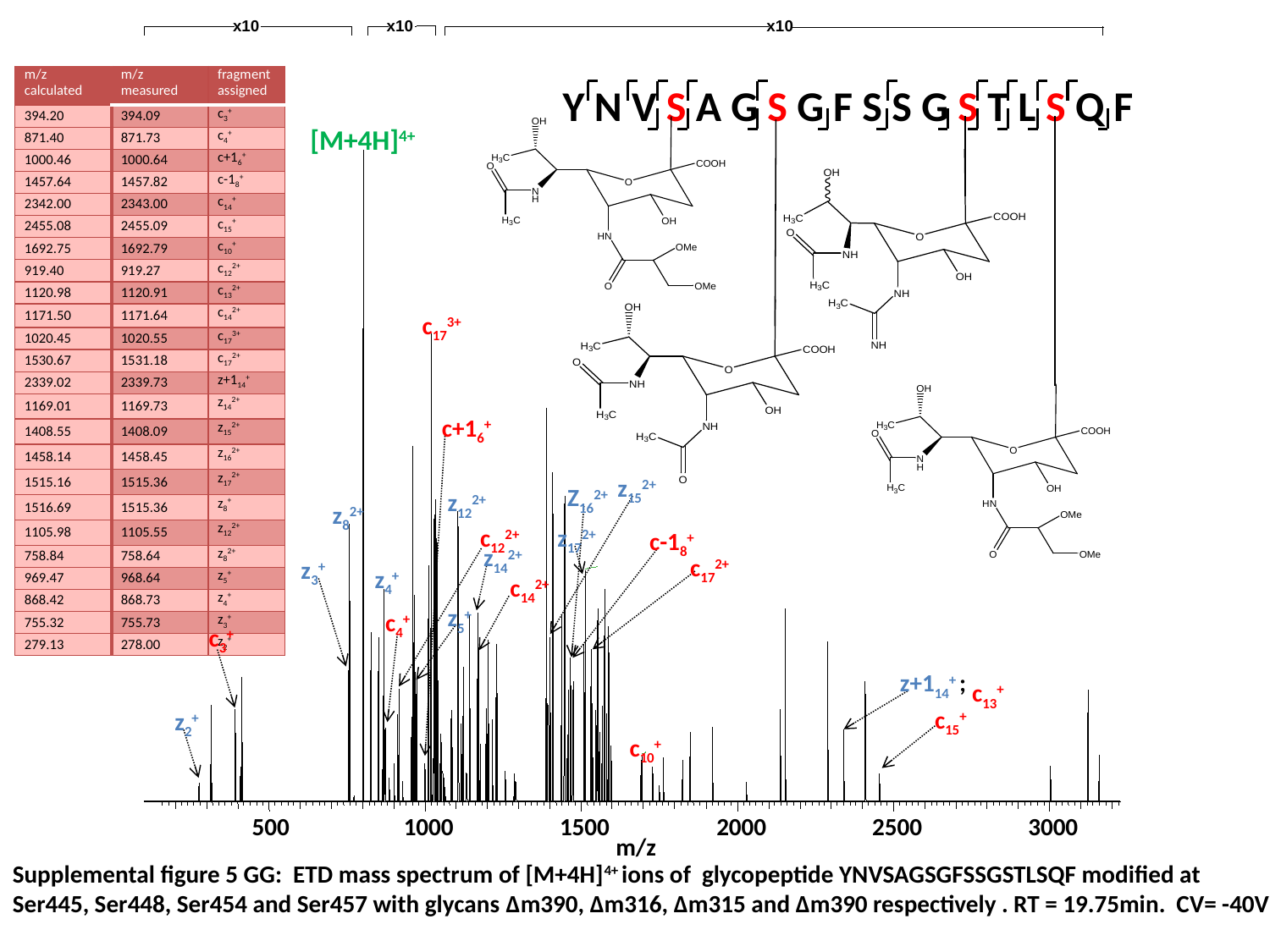

x10
x10
x10
| m/z calculated | m/z measured | fragment assigned |
| --- | --- | --- |
| 394.20 | 394.09 | c3+ |
| 871.40 | 871.73 | c4+ |
| 1000.46 | 1000.64 | c+16+ |
| 1457.64 | 1457.82 | c-18+ |
| 2342.00 | 2343.00 | c14+ |
| 2455.08 | 2455.09 | c15+ |
| 1692.75 | 1692.79 | c10+ |
| 919.40 | 919.27 | c122+ |
| 1120.98 | 1120.91 | c132+ |
| 1171.50 | 1171.64 | c142+ |
| 1020.45 | 1020.55 | c173+ |
| 1530.67 | 1531.18 | c172+ |
| 2339.02 | 2339.73 | z+114+ |
| 1169.01 | 1169.73 | z142+ |
| 1408.55 | 1408.09 | z152+ |
| 1458.14 | 1458.45 | z162+ |
| 1515.16 | 1515.36 | z172+ |
| 1516.69 | 1515.36 | z8+ |
| 1105.98 | 1105.55 | z122+ |
| 758.84 | 758.64 | z82+ |
| 969.47 | 968.64 | z5+ |
| 868.42 | 868.73 | z4+ |
| 755.32 | 755.73 | z3+ |
| 279.13 | 278.00 | z2+ |
Y N V S A G S G F S S G S T L S Q F
[M+4H]4+
c173+
c+16+
z152+
Z162+
z122+
z82+
c122+
z172+
c-18+
z142+
c172+
z3+
z4+
c142+
z5+
c4+
c3+
z+114+ ;
c13+
c15+
z2+
c10+
500
1000
1500
2000
2500
3000
m/z
Supplemental figure 5 GG: ETD mass spectrum of [M+4H]4+ ions of glycopeptide YNVSAGSGFSSGSTLSQF modified at Ser445, Ser448, Ser454 and Ser457 with glycans Δm390, Δm316, Δm315 and Δm390 respectively . RT = 19.75min. CV= -40V

## Slide 34
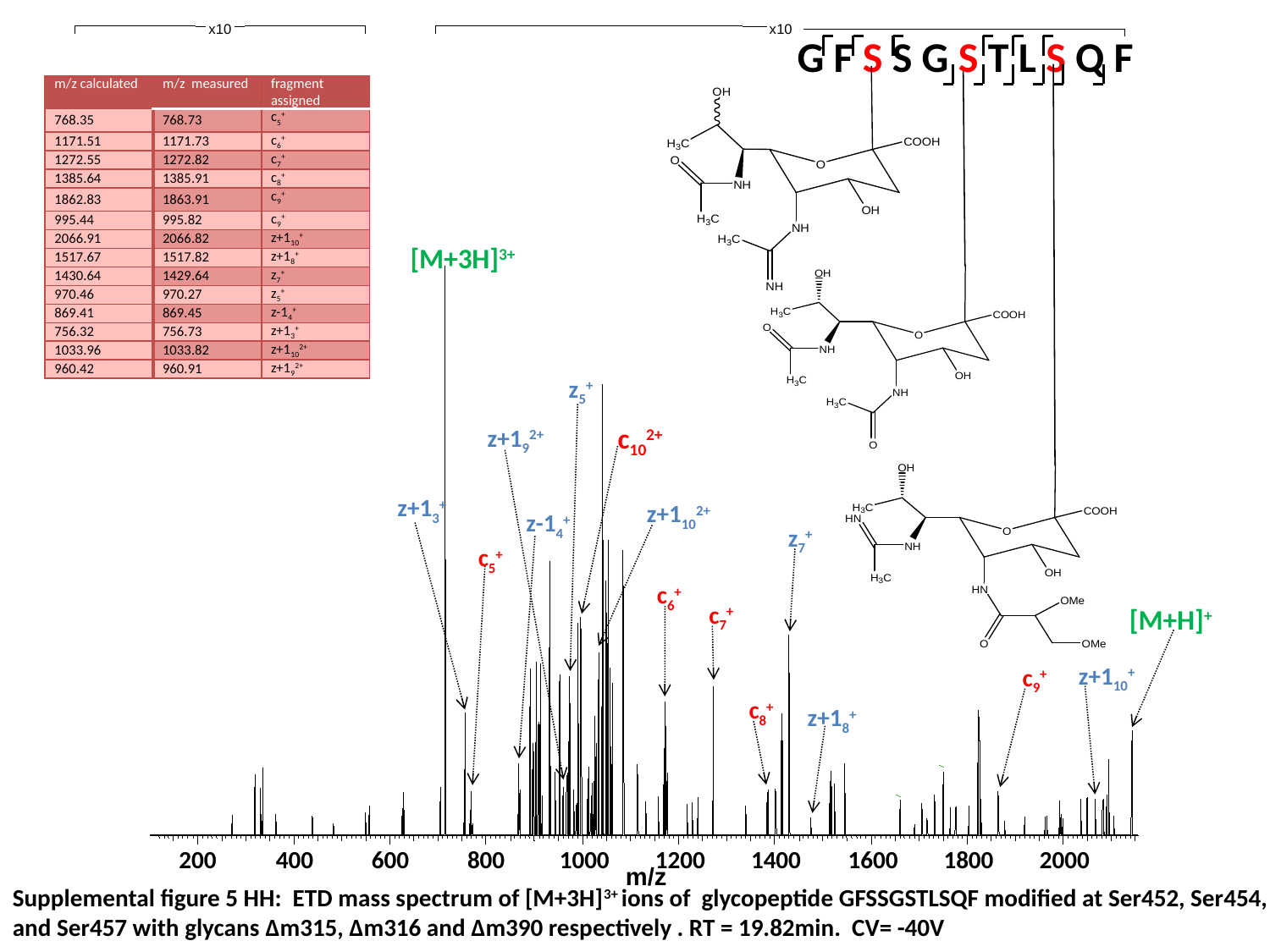

x10
x10
G F S S G S T L S Q F
| m/z calculated | m/z measured | fragment assigned |
| --- | --- | --- |
| 768.35 | 768.73 | c5+ |
| 1171.51 | 1171.73 | c6+ |
| 1272.55 | 1272.82 | c7+ |
| 1385.64 | 1385.91 | c8+ |
| 1862.83 | 1863.91 | c9+ |
| 995.44 | 995.82 | c9+ |
| 2066.91 | 2066.82 | z+110+ |
| 1517.67 | 1517.82 | z+18+ |
| 1430.64 | 1429.64 | z7+ |
| 970.46 | 970.27 | z5+ |
| 869.41 | 869.45 | z-14+ |
| 756.32 | 756.73 | z+13+ |
| 1033.96 | 1033.82 | z+1102+ |
| 960.42 | 960.91 | z+192+ |
[M+3H]3+
z5+
c102+
z+192+
z+13+
z+1102+
z-14+
z7+
c5+
c6+
c7+
[M+H]+
z+110+
c9+
c8+
z+18+
200
400
600
800
1000
1200
1400
1600
1800
2000
m/z
Supplemental figure 5 HH: ETD mass spectrum of [M+3H]3+ ions of glycopeptide GFSSGSTLSQF modified at Ser452, Ser454, and Ser457 with glycans Δm315, Δm316 and Δm390 respectively . RT = 19.82min. CV= -40V

## Slide 35
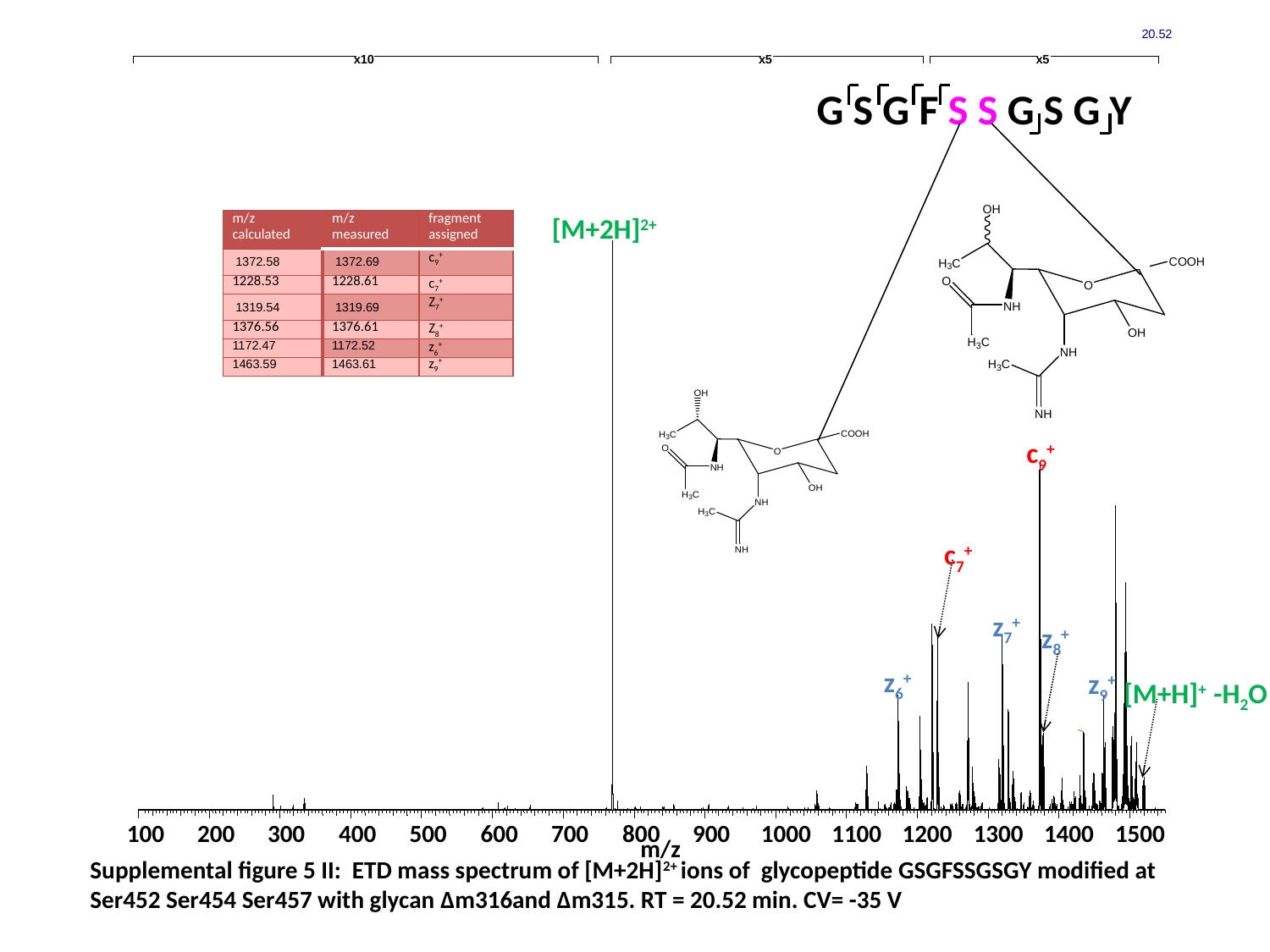

[M+3H]3+
20.52
x10
x5
x5
G S G F S S G S G Y
[M+2H]2+
| m/z calculated | m/z measured | fragment assigned |
| --- | --- | --- |
| 1372.58 | 1372.69 | c9+ |
| 1228.53 | 1228.61 | c7+ |
| 1319.54 | 1319.69 | Z7+ |
| 1376.56 | 1376.61 | Z8+ |
| 1172.47 | 1172.52 | z6+ |
| 1463.59 | 1463.61 | z9+ |
c9+
c7+
z7+
z8+
z6+
z9+
[M+H]+ -H2O
100
200
300
400
500
600
700
800
900
1000
1100
1200
1300
1400
1500
m/z
Supplemental figure 5 II: ETD mass spectrum of [M+2H]2+ ions of glycopeptide GSGFSSGSGY modified at Ser452 Ser454 Ser457 with glycan Δm316and Δm315. RT = 20.52 min. CV= -35 V

## Slide 36
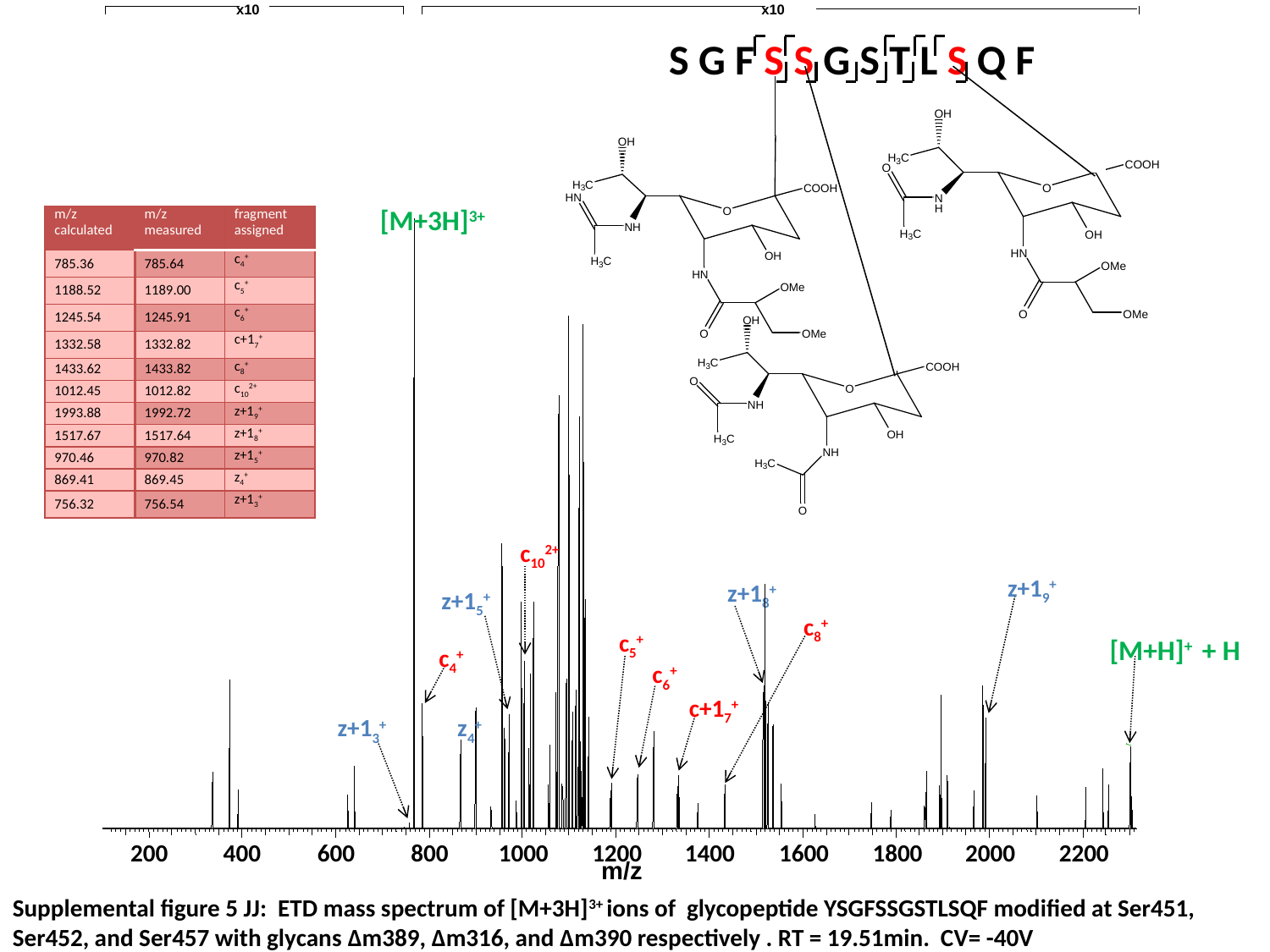

x10
x10
S G F S S G S T L S Q F
[M+3H]3+
| m/z calculated | m/z measured | fragment assigned |
| --- | --- | --- |
| 785.36 | 785.64 | c4+ |
| 1188.52 | 1189.00 | c5+ |
| 1245.54 | 1245.91 | c6+ |
| 1332.58 | 1332.82 | c+17+ |
| 1433.62 | 1433.82 | c8+ |
| 1012.45 | 1012.82 | c102+ |
| 1993.88 | 1992.72 | z+19+ |
| 1517.67 | 1517.64 | z+18+ |
| 970.46 | 970.82 | z+15+ |
| 869.41 | 869.45 | z4+ |
| 756.32 | 756.54 | z+13+ |
c102+
z+19+
z+18+
z+15+
c8+
c5+
[M+H]+ + H
c4+
c6+
c+17+
z+13+
z4+
200
400
600
800
1000
1200
1400
1600
1800
2000
2200
m/z
Supplemental figure 5 JJ: ETD mass spectrum of [M+3H]3+ ions of glycopeptide YSGFSSGSTLSQF modified at Ser451, Ser452, and Ser457 with glycans Δm389, Δm316, and Δm390 respectively . RT = 19.51min. CV= -40V

## Slide 37
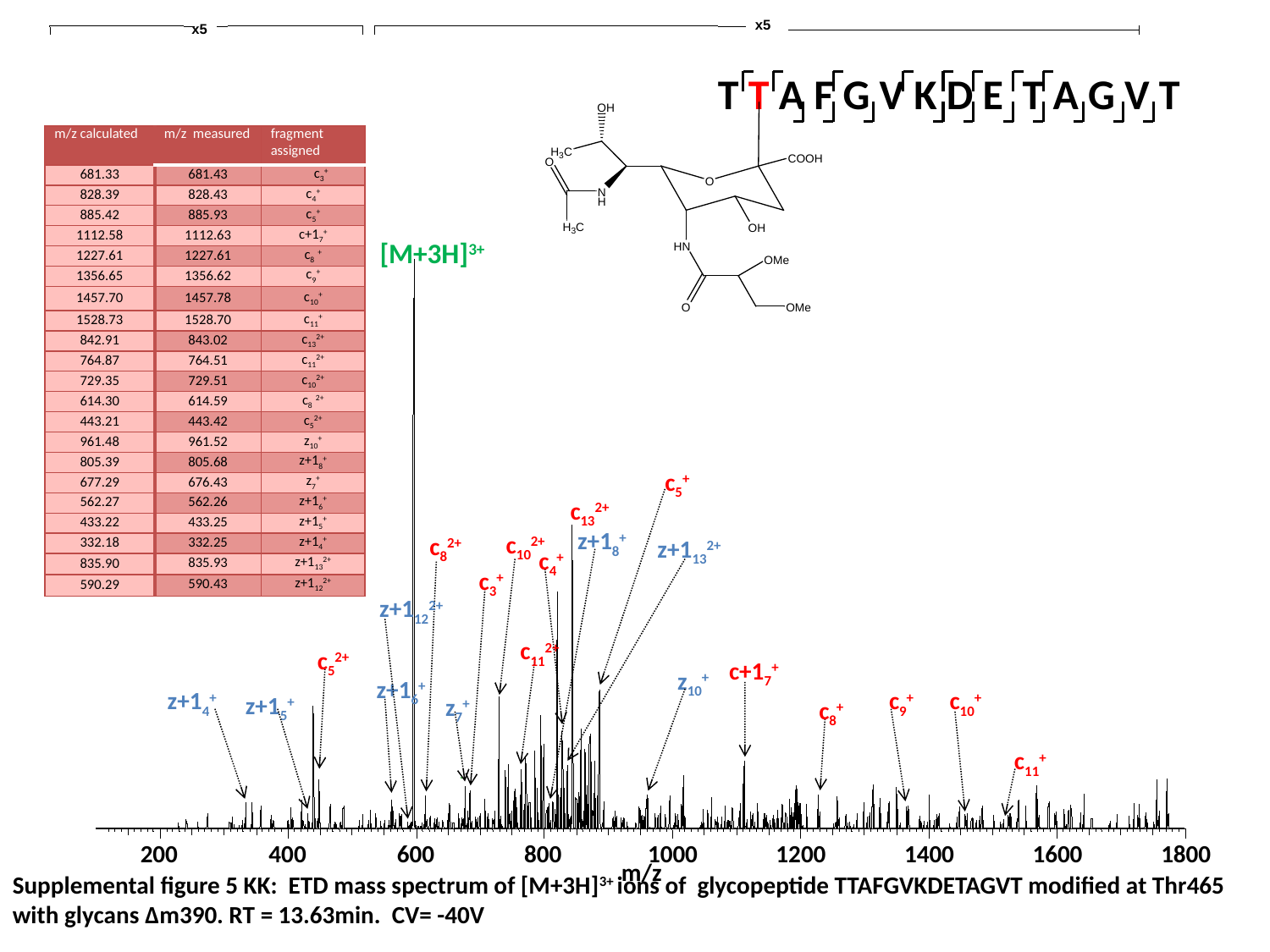

x5
x5
T T A F G V K D E T A G V T
| m/z calculated | m/z measured | fragment assigned |
| --- | --- | --- |
| 681.33 | 681.43 | c3+ |
| 828.39 | 828.43 | c4+ |
| 885.42 | 885.93 | c5+ |
| 1112.58 | 1112.63 | c+17+ |
| 1227.61 | 1227.61 | c8 + |
| 1356.65 | 1356.62 | c9+ |
| 1457.70 | 1457.78 | c10+ |
| 1528.73 | 1528.70 | c11+ |
| 842.91 | 843.02 | c132+ |
| 764.87 | 764.51 | c112+ |
| 729.35 | 729.51 | c102+ |
| 614.30 | 614.59 | c8 2+ |
| 443.21 | 443.42 | c52+ |
| 961.48 | 961.52 | z10+ |
| 805.39 | 805.68 | z+18+ |
| 677.29 | 676.43 | z7+ |
| 562.27 | 562.26 | z+16+ |
| 433.22 | 433.25 | z+15+ |
| 332.18 | 332.25 | z+14+ |
| 835.90 | 835.93 | z+1132+ |
| 590.29 | 590.43 | z+1122+ |
[M+3H]3+
c5+
c132+
z+18+
c102+
c82+
z+1132+
c4+
c3+
z+1122+
c112+
c52+
c+17+
z10+
z+16+
z+14+
c9+
c10+
z+15+
z7+
c8+
c11+
200
400
600
800
1000
1200
1400
1600
1800
m/z
Supplemental figure 5 KK: ETD mass spectrum of [M+3H]3+ ions of glycopeptide TTAFGVKDETAGVT modified at Thr465 with glycans Δm390. RT = 13.63min. CV= -40V
