# Supplementary material for: Comprehensive mapping of O‐glycosylation in flagellin from Campylobacter jejuni 11168: A multienzyme differential ion mobility mass spectrometry approach
Source: Proteomics. 2015 Jun 15;15(16):2733–45. doi: 10.1002/pmic.201400533 (PMC4975691; doi:10.1002/pmic.201400533)
Supplement: Supplementary file 1 — Figure S1. SDS‐PAGE analysis of purified Campylobacter jejuni flagellin protein. 10% SDS‐PAGE gel, stained with Coomassie blue. Lane 1 – MW markers. Lane 2 – cell suspension from C. jejuni strain 11168 culture, Lane 3 – purified flagellin protein Figure S2. Figure S3. Figure S4. Figure S5. Supplemental Table 1: Non‐glycopeptides identified from tryptic digest of flagellin following ETD MS/MS (with and without FAIMS). (Note that where peptides were identified from both replicates, m/zmeas values are given for replicate#1). Supplemental Table 2: Non‐glycopeptides identified from proteinase K digest of flagellin following ETD MS/MS (without FAIMS). (Note that where peptides were identified from both replicates, m/zmeas values are given for replicate#2). Supplemental Table 3: Non‐glycopeptides identified from proteinase K digest of flagellin following ETD MS/MS (with FAIMS). (Note that where peptides were identified from both replicates, m/zmeas values are given for replicate#2). Comprehensive mapping of O‐glycosylation in flagellin from Campylobacter jejuni 11168: A multi‐enzyme differential ion mobility mass spectrometry approach [file PMIC-15-2733-s001.zip › pmic201400533-sup-0007-Tables 1-3.docx]

**Supplemental Table 1:** Non-glycopeptides identified from tryptic digest of flagellin following ETD MS/MS (with and without FAIMS). (Note that where peptides were identified from both replicates, m/z_meas_ values are given for replicate#1).

| Peptide sequence | Modification | Site | Charge state | m/z_meas_ | m/z_calc_ | Δ ppm | CV | #1 | #2 |
| --- | --- | --- | --- | --- | --- | --- | --- | --- | --- |
| *Trypsin proteolysis and LC MS/MS* | | | | | | | | | |
| ADIGSVQNQVTSTINNITVTQVNVK | n/a | n/a | 3+ | 881.7987 | 881.8032 | 0.94 | n/a | ✓ | ✓ |
| AGATSDTFAINGVK | n/a | n/a | 2+ | 676.3444 | 676.3462 | -2.7 | n/a | ✓ | ✓ |
| AmDEQLK | Oxidation | M82 | 2+ | 425.7032 | 425.7024 | 1.89 | n/a | ✓ | ✓ |
| ANILAQSGSYAMAQANSVQQNVLR | n/a | n/a | 3+ | 850.7593 | 850.7625 | -3.9 | n/a | ✓ |  |
| ANILAQSGSYAmAQANSVQQNVLR | Oxidation | M557 | 3+ | 850.7593 | 850.7625 | -3.82 | n/a | ✓ |  |
| ATFTVETR | n/a | n/a | 2+ | 462.7434 | 462.7429 | 0.99 | n/a | ✓ | ✓ |
| ATIGATQSSK | n/a | n/a | 2+ | 482.26 | 482.2589 | 2.11 | n/a | ✓ | ✓ |
| ATQAAQDGQSLK | n/a | n/a | 2+ | 609.3108 | 609.3097 | 108 | n/a | ✓ | ✓ |
| DETAGVTTLK | n/a | n/a | 2+ | 517.7709 | 517.7719 | -1.9 | n/a | ✓ | ✓ |
| DGDANGALVAAINSVK | n/a | n/a | 2+ | 757.8917 | 757.8941 | -3.2 | n/a | ✓ | ✓ |
| DTTGVEASIDANGQLLLTSR | n/a | n/a | 3+ | 687.684 | 687.6868 | -4.1 | n/a | ✓ | ✓ |
| DVDFAAESANYSK | n/a | n/a | 3+ | 472.8793 | 472.8808 | -3.2 | n/a | ✓ | ✓ |
| ESKGQIDANIADAMGFGSANK | n/a | n/a | 3+ | 708.6696 | 708.6724 | -4.06 | n/a | ✓ | ✓ |
| GAMAVMDIAETAITNLDQIR | n/a | n/a | 3+ | 711.6949 | 711.6942 | 0.99 | n/a | ✓ | ✓ |
| GAmAVMDIAETAITNLDQIR | Oxidation | M483 | 3+ | 717.0266 | 717.0258 | -4.6 | n/a | ✓ | ✓ |
| GAMAVmDIAETAITNLDQIR | Oxidation | M486 | 3+ | 717.027 | 717.0258 | 1.71 | n/a | ✓ | ✓ |
| GAmAVmDIAETAITNLDQIR | Oxidation | M483,M486 | 3+ | 722.3583 | 722.3574 | 1.22 | n/a | ✓ | ✓ |
| GIKIDGNIGGGAFINADMKENYGR | n/a | n/a | 4+ | 628.3148 | 628.3169 | -3.22 | n/a | ✓ | ✓ |
| GQIDANIADAMGFGSANK | n/a | n/a | 3+ | 593.9467 | 593.9492 | -4.2 | n/a | ✓ | ✓ |
| GQIDANIADAmGFGSANK | Oxidation | M380 | 2+ | 898.4175 | 898.4177 | -0.13 | n/a | ✓ | ✓ |
| IDGNIGGGAFINADMK | n/a | n/a | 2+ | 796.891 | 796.8906 | 0.56 | n/a | ✓ | ✓ |
| IDGNIGGGAFINADmK | Oxidation | M323 | 2+ | 804.8846 | 804.888 | -4.3 | n/a | ✓ | ✓ |
| IDGNIGGGAFINADMKENYGR | n/a | n/a | 3+ | 738.0169 | 738.0199 | -4.19 | n/a | ✓ | ✓ |
| IDGNIGGGAFINADmKENYGR | Oxidation | M323 | 3+ | 743.3488 | 743.3516 | -3.72 | n/a | ✓ | ✓ |
| INSAADDASGMAIADSLR | n/a | n/a | 2+ | 889.4234 | 889.4229 | 0.51 | n/a | ✓ | ✓ |
| INSAADDASGmAIADSLR | Oxidation | M48 | 3+ | 598.6138 | 593.2844 | -3.7 | n/a | ✓ | ✓ |
| INTNVAALNAK | n/a | n/a | 2+ | 564.8217 | 564.8222 | -1.03 | n/a | ✓ | ✓ |
| LMEELDNIANTTSFNGK | n/a | n/a | 3+ | 632.9691 | 632.9718 | -4.3 | n/a | ✓ | ✓ |
| LmMEELDNIANTTSFNGK | Oxidation | M120 | 2+ | 956.9527 | 956.9515 | 1.22 | n/a | ✓ | ✓ |
| NYNGIDDFQFQK | n/a | n/a | 2+ | 744.8381 | 744.8413 | -4.3 | n/a | ✓ | ✓ |
| QLLSGNFINQEFQIGASSNQTVK | n/a | n/a | 3+ | 841.7683 | 841.7662 | 2.5 | n/a | ✓ | ✓ |
| SLDASLSR | n/a | n/a | 2+ | 424.7279 | 424.7272 | 1.5 | n/a | ✓ | ✓ |
| SQANTLGQAISNGNDALGILQTADK | n/a | n/a | 3+ | 834.0895 | 834.0928 | -4 | n/a | ✓ | ✓ |
| TMLQADINR | n/a | n/a | 2+ | 531.2744 | 531.2741 | 0.54 | n/a | ✓ | ✓ |
| TTAFGVKDETAGVTTLK | n/a | n/a | 3+ | 580.3096 | 580.3123 | -4.54 | n/a | ✓ | ✓ |
| VDYKDGDANGALVAAINSVK | n/a | n/a | 3+ | 674.0137 | 674.0164 | -3.97 | n/a | ✓ |  |
| *Trypsin proteolysis and LC FAIMS MS/MS* | | | | | | | | | |
| AAESQIR | n/a | n/a | 2+ | 387.7085 | 387.7089 | -0.99 | -45 |  | ✓ |
| AAESQIRDVDFAAESANYSK | n/a | n/a | 3+ | 724.679 | 724.6783 | 0.85 | -30 | ✓ |  |
| ADIGSVQNQVTSTINNITVTQVNVK | n/a | n/a | 3+ | 881.8024 | 881.8031 | -0.9 | -25 | ✓ | ✓ |
| AGATSDTFAINGVK | n/a | n/a | 3+ | 451.2335 | 451.2333 | 0.4 | -50 | ✓ | ✓ |
| AMDEQLK | n/a | n/a | 2+ | 417.7049 | 417.705 | -0.14 | -45 | ✓ |  |
| AmDEQLK | Oxidation | M82 | 3+ | 425.7014 | 425.7024 | -2.3 | -40 | ✓ | ✓ |
| AMDEQLKILDTIK | n/a | n/a | 3+ | 506.6137 | 506.613 | 1.4 | -40 | ✓ |  |
| AmDEQLKILDTIK | Oxidation | M82 | 3+ | 511.9456 | 511.9446 | 2 | -40 | ✓ |  |
| ANADLNSK | n/a | n/a | 2+ | 416.7105 | 416.7116 | -2.7 | -45 |  | ✓ |
| ANILAQSGSYAMAQANSVQQNVLR | n/a | n/a | 3+ | 845.4299 | 845.4309 | -1.2 | -25 | ✓ |  |
| ANILAQSGSYAmAQANSVQQNVLR | Oxidation | M557 | 3+ | 850.7614 | 850.7625 | -1.31 | -25 | ✓ |  |
| ATFTVETR | n/a | n/a | 3+ | 462.7428 | 462.7429 | -0.2 | -40 | ✓ | ✓ |
| ATIGATQSSK | n/a | n/a | 2+ | 482.2586 | 482.2589 | -0.6 | -25 | ✓ | ✓ |
| ATQAAQDGQSLK | n/a | n/a | 3+ | 406.5426 | 406.5422 | 0.94 | -50 | ✓ | ✓ |
| ATQAAQDGQSLKTR | n/a | n/a | 2+ | 737.8826 | 737.8841 | -2 | -20 | ✓ |  |
| DETAGVTTLK | n/a | n/a | 2+ | 517.7722 | 517.7719 | 0.6 | -35 | ✓ | ✓ |
| DGDANGALVAAINSVK | n/a | n/a | 3+ | 505.5977 | 505.5985 | -1.6 | -30 | ✓ | ✓ |
| DGDANGALVAAINSVKDTTGVEASIDANGQLLLTSR | n/a | n/a | 3+ | 1186.2723 | 1186.275 | -1.87 | -20 | ✓ |  |
| DTTGVEASIDANGQLLLTSR | n/a | n/a | 3+ | 687.6876 | 687.6868 | 1.2 | -35 | ✓ |  |
| DVDFAAESANYSK | n/a | n/a | 3+ | 472.8809 | 472.8808 | 0.2 | -50 | ✓ | ✓ |
| ESKGQIDANIADAMGFGSANK | n/a | n/a | 3+ | 708.6728 | 708.6724 | 0.51 | -40 | ✓ |  |
| ESKGQIDANIADAmGFGSANK | Oxidation | M380 | 3+ | 714.0048 | 714.004 | 1.1 | -40 | ✓ |  |
| GAMAVMDIAETAITNLDQIR | n/a | n/a | 3+ | 711.6933 | 711.6942 | -1.24 | -35 | ✓ | ✓ |
| GAmAVMDIAETAITNLDQIR | Oxidation | M483 | 4+ | 538.022 | 538.0211 | 1.7 | -35 | ✓ | ✓ |
| GAMAVmDIAETAITNLDQIR | Oxidation | M486 | 3+ | 717.0264 | 717.0258 | 0.8 | -35 | ✓ | ✓ |
| GAmAVmDIAETAITNLDQIR | Oxidation | M483, M486 | 3+ | 722.3567 | 722.3574 | -1.1 | -25 | ✓ | ✓ |
| GIKIDGNIGGGAFINADMKENYGR | n/a | n/a | 3+ | 837.4203 | 837.4201 | 0.2 | -35 | ✓ |  |
| GIKIDGNIGGGAFINADmKENYGR | Oxidation | M323 | 3+ | 842.7525 | 842.7518 | 0.9 | -30 | ✓ |  |
| GQIDANIADAMGFGSANK | n/a | n/a | 3+ | 593.948 | 593.9492 | -2.07 | -40 | ✓ | ✓ |
| GQIDANIADAmGFGSANK | Oxidation | M380 | 2+ | 898.4161 | 898.4176 | -1.7 | -45 | ✓ | ✓ |
| IDGNIGGGAFINADMK | n/a | n/a | 3+ | 536.9285 | 536.9277 | 1.5 | -40 | ✓ | ✓ |
| IDGNIGGGAFINADmK | Oxidation | M323 | 2+ | 804.8868 | 804.888 | -1.52 | -25 | ✓ | ✓ |
| IDGNIGGGAFINADMKENYGR | n/a | n/a | 3+ | 738.02 | 738.02 | 0.03 | -45 | ✓ |  |
| IDGNIGGGAFINADMKENYGR | Oxidation | M323 | 3+ | 743.3515 | 743.3515 | 0.8 | -40 | ✓ |  |
| IGKVDYKDGDANGALVAAINSVK | n/a | n/a | 3+ | 773.4177 | 773.4165 | 1.6 | -30 | ✓ |  |
| ILDTIKTK | n/a | n/a | 2+ | 466.2948 | 466.2948 | 0.09 | -30 | ✓ |  |
| INSAADDASGMAIADSLR | n/a | n/a | 3+ | 593.2853 | 593.2844 | 1.5 | -40 | ✓ | ✓ |
| INSAADDASGmAIADSLR | Oxidation | M48 | 3+ | 598.6157 | 598.616 | -2.5 | -30 | ✓ | ✓ |
| INTNVAALNAK | n/a | n/a | 2+ | 564.8203 | 564.8222 | -3.4 | -30 | ✓ | ✓ |
| LmEELDNIANTTSFNGK | Oxidation | M120 | 3+ | 638.3042 | 638.3034 | 1.3 | -50 | ✓ | ✓ |
| LMELDNIANTTSFNGK | n/a | n/a | 3+ | 632.9714 | 632.9718 | -0.58 | -45 | ✓ | ✓ |
| NYNGIDDFQFQK | n/a | n/a | 3+ | 496.8975 | 496.8966 | 1.8 | -45 | ✓ | ✓ |
| QLLSGNFINQEFQIGASSNQTVK | n/a | n/a | 3+ | 841.7679 | 841.7661 | 2.1 | -30 | ✓ |  |
| SLDASLSR | n/a | n/a | 2+ | 424.7263 | 424.7272 | -2.3 | -40 | ✓ | ✓ |
| SQANTLGQAISNGNDALGILQTADK | n/a | n/a | 4+ | 625.8229 | 625.8214 | 2.4 | -50 | ✓ | ✓ |
| SQANTLGQAISNGNDALGILQTADKAMDEQLK | n/a | n/a | 3+ | 1105.88750 | 1105.89 | -0.27 | -25 | ✓ |  |
| SQANTLGQAISNGNDALGILQTADKAmDEQLK | Oxidation | M82 | 5+ | 667.13570 | 667.13 | 1.71 | -45 | ✓ |  |
| SQANTLGQAISNGNDALGILQTADKAMDEQLKILDTIK | n/a | n/a | 4+ | 1000.52310 | 1000.52 | -0.01 | -25 | ✓ |  |
| SQANTLGQAISNGNDALGILQTADKAmDEQLKILDTIK | Oxidation | M82 | 4+ | 1004.52190 | 1004.52 | 0.04 | -20 | ✓ |  |
| TKATQAAQDGQSLK | n/a | n/a | 2+ | 482.9237 | 482.9231 | 1.2 | -50 | ✓ |  |
| TMLQADINR | n/a | n/a | 2+ | 531.2727 | 531.274 | -2.6 | -35 | ✓ | ✓ |
| TmLQADINR | Oxidation | M111 | 2+ | 539.2713 | 539.2715 | -0.42 | -30 | ✓ | ✓ |
| TMLQADINRLMEELDNIANTTSFNGK | n/a | n/a | 3+ | 980.4786 | 980.4794 | -0.86 | -25 | ✓ |  |
| TmLQADINRLMEELDNIANTTSFNGK | Oxidation | M111 | 3+ | 985.8121 | 985.8111 | 1.03 | -30 | ✓ |  |
| TMLQADINRLmEELDNIANTTSFNGK | Oxidation | M120 | 3+ | 985.801 | 985.8111 | -1.14 | -25 | ✓ |  |
| TmLQADINRLmEELDNIANTTSFNGK | Oxidation | M111,M120 | 3+ | 991.143 | 991.1427 | 0.25 | -25 | ✓ |  |
| TTAFGVKDETAGVTTLK | n/a | n/a | 2+ | 869.9631 | 869.9647 | -1.87 | -20 | ✓ |  |
| VDYKDGDANGALVAAINSVK | n/a | n/a | 2+ | 1010.519 | 1010.521 | -1.91 | -20 | ✓ |  |
| VDYKDGDANGALVAAINSVKDTTGVEASIDANGQLLLTSR | n/a | n/a | 4+ | 1016.2711 | 1016.271 | -0.03 | -25 | ✓ |  |

**Supplemental Table 2:** Non-glycopeptides identified from proteinase K digest of flagellin following ETD MS/MS (without FAIMS). (Note that where peptides were identified from both replicates, m/z_meas_ values are given for replicate#2).

| Peptide sequence | Modification | Site | Charge state | m/z_meas_ | m/z_calc_ | Δ ppm | CV | #1 | #2 |
| --- | --- | --- | --- | --- | --- | --- | --- | --- | --- |
|  | | | | | | | | | |
| ADMKENYGRL | n/a | n/a | 2+ | 598.7851 | 598.7901 | -8.3 | n/a |  |  |
| ITNLDQIRAD | n/a | n/a | 2+ | 579.8038 | 579.8093 | -9.5 | n/a |  |  |
| KIDGNIGGGAF | n/a | n/a | 2+ | 524.7698 | 524.7747 | -9.3 | n/a |  |  |
| RLMEELDNIAN | n/a | n/a | 2+ | 659.3208 | 659.3270 | -9.5 | n/a |  |  |

**Supplemental** **Table 3:** Non-glycopeptides identified from proteinase K digest of flagellin following ETD MS/MS (with FAIMS). (Note that where peptides were identified from both replicates, m/z_meas_ values are given for replicate#2).

| Peptide sequence | Glycan(s) | Site | Charge state | m/z_meas_ | m/z_calc_ | Δ ppm | CV | #1 | #2 |
| --- | --- | --- | --- | --- | --- | --- | --- | --- | --- |
| *Proteinase K proteolysis and LC FAIMS MS/MS* | | | | | | | |  |  |
| ADMKENYGRL | n/a | n/a | 3+ | 399.5269 | 399.5291 | -5.5 | -50 |  |  |
| DKAMDEQLKI | n/a | n/a | 3+ | 435.2333 | 435.2357 | -5.5 | -45 |  |  |
| GALADEINKN | n/a | n/a | 2+ | 522.7668 | 522.7696 | -5.4 | -30 | ⬝ |  |
| GNDALGIL | n/a | n/a | 2+ | 386.7114 | 386.7136 | -5.7 | -35 | ⬝ |  |
| GSANKGVVL | n/a | n/a | 2+ | 422.7457 | 422.748 | -5.4 | -30 | ⬝ |  |
| GVKDETAGVT | n/a | n/a | 2+ | 488.7508 | 488.751 | -0.32 | -35 |  |  |
| INADMKENYGRL | n/a | n/a | 3+ | 475.2352 | 475.2381 | -6 | -55 | ⬝ |  |
| IRDVDFAAE | n/a | n/a | 3+ | 518.2558 | 518.2589 | -6.08 | -35 | ⬝ |  |
| ITNLDQIR | n/a | n/a | 2+ | 486.7749 | 486.7773 | -4.86 | -40 | ⬝ |  |
| ITNLDQIRA | n/a | n/a | 2+ | 522.293 | 522.2958 | -5.4 | -30 | ⬝ |  |
| ITNLDQIRAD | n/a | n/a | 2+ | 579.8059 | 579.8093 | -5.9 | -35 | ⬝ |  |
| ITNLDQIRADIG | n/a | n/a | 2+ | 664.8593 | 664.8621 | -4.2 | -25 | ⬝ |  |
| KIDGNIGGGAF | n/a | n/a | 2+ | 524.772 | 524.7747 | -5.1 | -35 | ⬝ |  |
| LDQIRADIG | n/a | n/a | 2+ | 500.772 | 500.7747 | -5.4 | -30 | ⬝ |  |
| LKNYNGIDDF | n/a | n/a | 2+ | 599.788 | 599.7906 | -4.3 | -35 | ⬝ |  |
| MDEQLKIL | n/a | n/a | 2+ | 495.2679 | 495.4705 | -5.09 | -30 | ⬝ |  |
| MEELDNIA | n/a | n/a | 2+ | 467.7105 | 467.713 | -5.22 | -45 | ⬝ |  |
| MEELDNIAN | n/a | n/a | 2+ | 524.7318 | 524.7344 | -5.14 | -35 | ⬝ |  |
| MEELDNIANTT | n/a | n/a | 2+ | 625.7792 | 625.7821 | -4.6 | -30 | ⬝ |  |
| NLDQIRA | n/a | n/a | 2+ | 415.228 | 4215.23 | -4.78 | -40 | ⬝ |  |
| NLDQIRADIG | n/a | n/a | 2+ | 557.7936 | 557.7962 | -4.64 | -25 | ⬝ |  |
| NYNGIDDF | n/a | n/a | 2+ | 479.1989 | 479.2011 | -4.6 | -40 | ⬝ |  |
| REGRGIKIDGNIGGGAF | n/a | n/a | 3+ | 572.9731 | 572.9762 | -5.4 | -40 | ⬝ |  |
| RLMEEL | n/a | n/a | 2+ | 395.708 | 395.71 | -5.14 | -40 | ⬝ |  |
| RLMEELDNIA | n/a | n/a | 2+ | 602.303 | 602.3056 | -4.31 | -25 | ⬝ |  |
| RLmEELDNIA | Oxidation | M120 | 2+ | 610.3005 | 610.303 | -4.19 | -25 | ⬝ |  |
| RLMEELDNIAN | n/a | n/a | 2+ | 659.3242 | 659.327 | -4.28 | -25 | ⬝ |  |
| RLmEELDNIAN | Oxidation | M120 | 2+ | 667.3215 | 667.3245 | -4.5 | -25 | ⬝ |  |
| RLMEELDNIANTT | n/a | n/a | 2+ | 768.3691 | 768.3721 | -3.9 | -25 |  |  |
| SREGRGIKIDGNIGGGA | n/a | n/a | 3+ | 552.9597 | 552.9641 | -8 | -50 | ⬝ |  |
| SREGRGIKIDGNIGGGAF | n/a | n/a | 3+ | 601.9827 | 601.9869 | -7 | -55 | ⬝ |  |
